# Comparing potassium-competitive acid blocker-based therapies for *Helicobacter pylori* infection: A Bayesian network meta-analysis of 77 randomised controlled trials

**DOI:** 10.3389/fphys.2026.1843686

**Published:** 2026-07-07

**Authors:** Changmin Mi, Kai Zhou, Yanyan Shi, Kailun Liang, Fang Zhou, Zhiqiang Song

**Affiliations:** 1 Department of Gastroenterology, Peking University Third Hospital, Beijing, China; 2 Research Center of Clinical Epidemiology, Peking University Third Hospital, Beijing, China; 3 Takeda Pharmaceutical Company, Shanghai, China

**Keywords:** eradication rate, *Helicobacter pylori*, network meta-analysis, potassium-competitive acid blockers, vonoprazan

## Abstract

**Background:**

Eradication rates for *Helicobacter pylori* (*H. pylori*) with proton pump inhibitor (PPI)-based therapy have declined due to rising resistance and pharmacokinetic limitations. Several potassium-competitive acid blockers (PCABs) are now approved for *H. pylori* eradication. However, direct comparisons among PCABs-based regimens are limited.

**Aim:**

To evaluate the efficacy, safety and compliance of PCAB-based regimens for *H. pylori* eradication.

**Methods:**

Randomised controlled trials in adults with *H. pylori* infection, evaluating PCAB-based dual, triple, or bismuth quadruple regimens or comparing PCAB- with PPI-based regimens, were included. The primary outcome was eradication rate; secondary outcomes were compliance and adverse events. Proportional meta-analysis and Bayesian network meta-analysis (NMA) with a random-effects model were conducted to compare efficacy and safety. Pooled estimates were expressed in odd ratio (OR) and corresponding 95% confidence intervals (CIs). Ranking used surface under the cumulative ranking curve (SUCRA). Subgroup analyses were conducted to explore potential sources of heterogeneity.

**Results:**

Seventy-seven RCTs involving 19,950 patients were included. Vonoprazan (VPZ)-based regimens showed the highest eradication rates in proportional meta-analysis (88.15–91.94%), while tegoprazan (TPZ)- and keverprazan (KEV)-based regimens demonstrated more variable performance. NMA demonstrated a significantly higher eradication rate in patients treated with VPZ-based quadruple regimen compared with VPZ-based dual (OR = 1.47, 95% CI 1.05–2.07) and TPZ-based triple (OR = 3.54, 95% CI 1.21–10.65) regimen. SUCRA ranking identified VPZ-based quadruple as the highest-ranked regimen (93.78%), followed by VPZ-based triple (70.55%) and TPZ-based quadruple (66.22%) regimen, indicating relative treatment ranking probabilities. Compliance rates were comparable across regimens with no significant differences, while VPZ- and TPZ-based dual therapies showed better safety profiles with lower adverse events than quadruple therapies (OR = 0.39–0.42).

**Conclusions:**

VPZ-based quadruple therapy demonstrated the highest eradication efficacy among PCAB-based regimens, with significantly superior performance compared with selected dual and triple therapies. VPZ-based dual therapy appears to have a better balance of efficacy and safety profile. However, as 76 of 77 included trials were conducted in Asian populations, these findings should be interpreted with caution when extrapolating to non-Asian settings.

**Systematic Review Registration:**

https://www.crd.york.ac.uk/PROSPERO/home ID= CRD420251125759 identifier CRD420251125759.

## 1 Introduction

Helicobacter pylori (H. pylori) is a gram-negative bacterium that colonises the gastric mucosa and plays a key role in gastric carcinogenesis, particularly non-cardia adenocarcinoma. Chronic infection can trigger a sequence of histological changes, including gastritis, atrophy, intestinal metaplasia, and dysplasia (Aumpan et al., 2023). A 2025 World Health Organization report indicates that H. pylori affects approximately 44% of adults and one-third of children globally (Morgan et al., 2025), and it was classified as a human carcinogen by the National Toxicology Program in 2021 (National Toxicology Program (NTP), 2021).

Eradication of H. pylori is recommended by international guidelines as a primary strategy to prevent gastric cancer (Chey et al., 2024; Patel et al., 2024). However, conventional proton pump inhibitor (PPI)-based regimens have declining efficacy, largely due to increasing antibiotic resistance and pharmacokinetic limitations. Current real-world studies indicate that the eradication rate of these regimens has fallen to below 80% (Tai et al., 2019; Kim et al., 2020).

Potassium-competitive acid blockers (PCABs) have emerged as a promising alternative to PPIs. These agents offer rapid and potent acid suppression that maintains consistent efficacy irrespective of CYP2C19 genotype (Aumpan et al., 2023; Elsabaawy et al., 2024; Kanu and Soldera, 2024). Vonoprazan (VPZ) was the first PCAB approved for H. pylori eradication. Following its 2014 launch in Japan, VPZ is now available in the United States and several Asian countries, including South Korea, Singapore, and China. In a recent multicentre randomised trial, VPZ maintained intragastric pH > 6 for 75.3% of the first day (median pH 7.4). By day 7, this increased to over 97.2% of the time, with a median intragastric pH value of 7.8 (Song et al., 2025b). It has achieved >90% eradication rates even in clarithromycin-resistant cases (Tungtrongchitr et al., 2024). Following the introduction of VPZ, tegoprazan (TPZ) and keverprazan (KEV) have also been introduced in several regions and have demonstrated comparable efficacy to PPIs in studies from China, although global evidence remains limited (Lee et al., 2019; Choi et al., 2022; Tan et al., 2023). Other PCABs, such as revaprazan and fexuprazan, possess distinct pharmacokinetic profiles and currently remain at early stages of clinical development or use (Han et al., 2019; Sunwoo et al., 2020; Kim et al., 2023).

Recent international guidelines, including the 2024 American College of Gastroenterology and American Gastroenterological Association guidelines, recognise PCABs as a viable alternative to PPIs for H. pylori eradication (Chey et al., 2024; Patel et al., 2024). While prior meta-analyses have compared PCABs with PPIs, no network meta-analysis (NMA) has yet simultaneously evaluated the dual, triple, and quadruple regimens incorporating VPZ, TPZ, and KEV. As a result, the relative efficacy and safety among different PCABs remain unclear (Shah et al., 2023; Ouyang et al., 2024). Moreover, existing research has not yet fully integrated emerging data on newer agents like KEV (Zhou et al., 2023). To address these gaps, this systematic review and NMA aims to compare the efficacy and safety of various PCAB-based regimens for H. pylori eradication. This evidence may help clinicians determine not only whether to use a PCAB-based regimen, but also which PCAB and treatment strategy may be most appropriate for individual patients.

## 2 Materials and methods

This study followed the Preferred Reporting Items for Systematic Reviews and Meta-analyses 2015 NMA Checklist (Hutton et al., 2015). The protocol was registered on PROSPERO (CRD420251125759).

### 2.1 Data sources and literature search

An electronic database search of PubMed, Embase, Cochrane Library, Web of Science, China Biology Medicine, China National Knowledge Infrastructure, Wanfang Data, and the VIP Database was conducted from inception to April 2025 using the keywords: Helicobacter infections, Potassium competitive acid blocker, and their synonyms. We searched without language restriction but limited inclusion to randomised controlled trials (RCTs). The search strategy is provided in **Supplementary Material 1 Search strategy**.

### 2.2 Study selection

We included RCTs that enrolled adults (≥18 years) with *H. pylori* confirmed by rapid urease test, 13C- or 14C-urea-breath test, faecal monoclonal antigen test, histology, culture identification, specific gene test, or other validated molecular methods. Studies relying solely on serology were excluded. Eligible interventions comprised dual, triple, or bismuth quadruple regimens based on PCABs, including VPZ, TPZ, KEV, and other PCABs regardless duration, frequency, and dosage. Trials comparing PCAB- with PPI-based regimens were also included.

Two reviewers independently screened studies for eligibility. Any disagreements were resolved through discussion with a third reviewer. The primary outcome was *H. pylori* eradication, defined as the absence of infection at least four weeks after treatment completion, as assessed using the follow-up methods reported in each trial. Secondary outcomes were the overall incidence of adverse events (AEs), defined as the proportion of patients experiencing any AEs, treatment discontinuation due to AEs, and treatment compliance, defined as the proportion of patients who received at least 80% of the prescribed medications.

### 2.3 Data extraction

Two reviewers extracted the following data from each study into a pre-defined data extraction form: trial characteristics (first author, publication year, country and region, and trial or study name), participant characteristics (sample size, method of detecting *H. pylori* infection, patient age, comorbidities, treatment history), intervention information (drug names, treatment duration), and outcome information (outcome name, definition, measurement time points). Any disagreements were resolved by discussion with a third reviewer. Where multiple follow-up assessments were reported, data from the longest available follow-up were extracted.

### 2.4 Data synthesis and analysis

A proportional meta-analysis was first performed to obtain pooled eradication rates for each treatment regimen with corresponding 95% confidence intervals (CIs). A Bayesian NMA was then conducted to compare the efficacy and safety of the different regimens. As all outcomes were dichotomous, effect sizes were expressed as odds ratios (ORs) with 95% CIs. The analyses were implemented in R software (version 4.3.1) using a random-effects model. Missing data were handled using the available-case data as reported in each individual trial. When available, intention-to-treat (ITT) populations were preferentially used. No additional imputation for missing outcome data was performed. Studies were included if sufficient data for effect size estimation were available.

Network plots were constructed to illustrate the evidence structure. In these plots, each node represented a treatment, with the node size proportional to the number of participants assigned to that regimen. Edges between nodes indicated available direct comparisons, with the line thickness reflecting the relative weight of evidence. The treatment ranking was estimated using the surface under the cumulative ranking curve (SUCRA). SUCRA values range from 0% to 100%, with higher values indicating a greater probability of being the most effective regimen.

Heterogeneity among studies was assessed by Cochran’s Q test (*χ*²) and the *I*^2^ statistic. A *p*-value of 0.05 or less for the *χ*^2^ test or *I*^2^ greater than 50% indicates substantial heterogeneity (Deeks et al., 2019). Pooled NMA results were presented as forest plots, and league tables were constructed to facilitate pairwise comparisons across all interventions. The assumption of transitivity was appraised by evaluating the comparability of study and patient characteristics across treatment comparisons, including baseline demographics, common control groups, and outcome definitions (Jansen et al., 2011). Clinical and methodological variables were reviewed to ensure reasonable similarity across the network. Consistency was assessed at both global and local levels. Globally, inconsistency was evaluated using a design-by-treatment interaction model, which yields a *χ*^2^ test statistic (*p* < 0.05 indicating significant inconsistency). Locally, a node-splitting approach was applied to compare direct and indirect evidence for specific treatment comparisons.

Sensitivity analyses were performed using fixed-effects models for all outcomes to assess the robustness of the results. In addition, sensitivity analyses excluding studies at high risk of bias were conducted for the overall analysis to evaluate the impact of study quality on the pooled estimates.

Publication bias was assessed when ≥10 studies were available for a direct comparison. Funnel plots were visually inspected, and Egger’s test was used to evaluate small-study effects, with *p* < 0.05 considered statistically significant.

Pre-specified subgroup analyses were conducted by treatment duration (7, 10, or 14 days), type of PCAB regimen (dual, triple, or bismuth quadruple), and treatment history (treatment-naive vs. previously treated). Additional analyses focused on Asian populations, particularly treatment-naive patients in China receiving 14-day regimens, reflecting the high prevalence of antibiotic resistance and the common recommendation of extended regimens in this region. When quantitative synthesis was not feasible, a descriptive analysis was performed.

### 2.5 Quality assessment

The quality of the included studies was assessed independently by two reviewers using the Cochrane risk-of-bias tool for randomised trials (Higgins et al., 2011). Any disagreements were resolved by discussion with a third reviewer.

## 3 Results

### 3.1 Results of study selection

**Figure 1:**
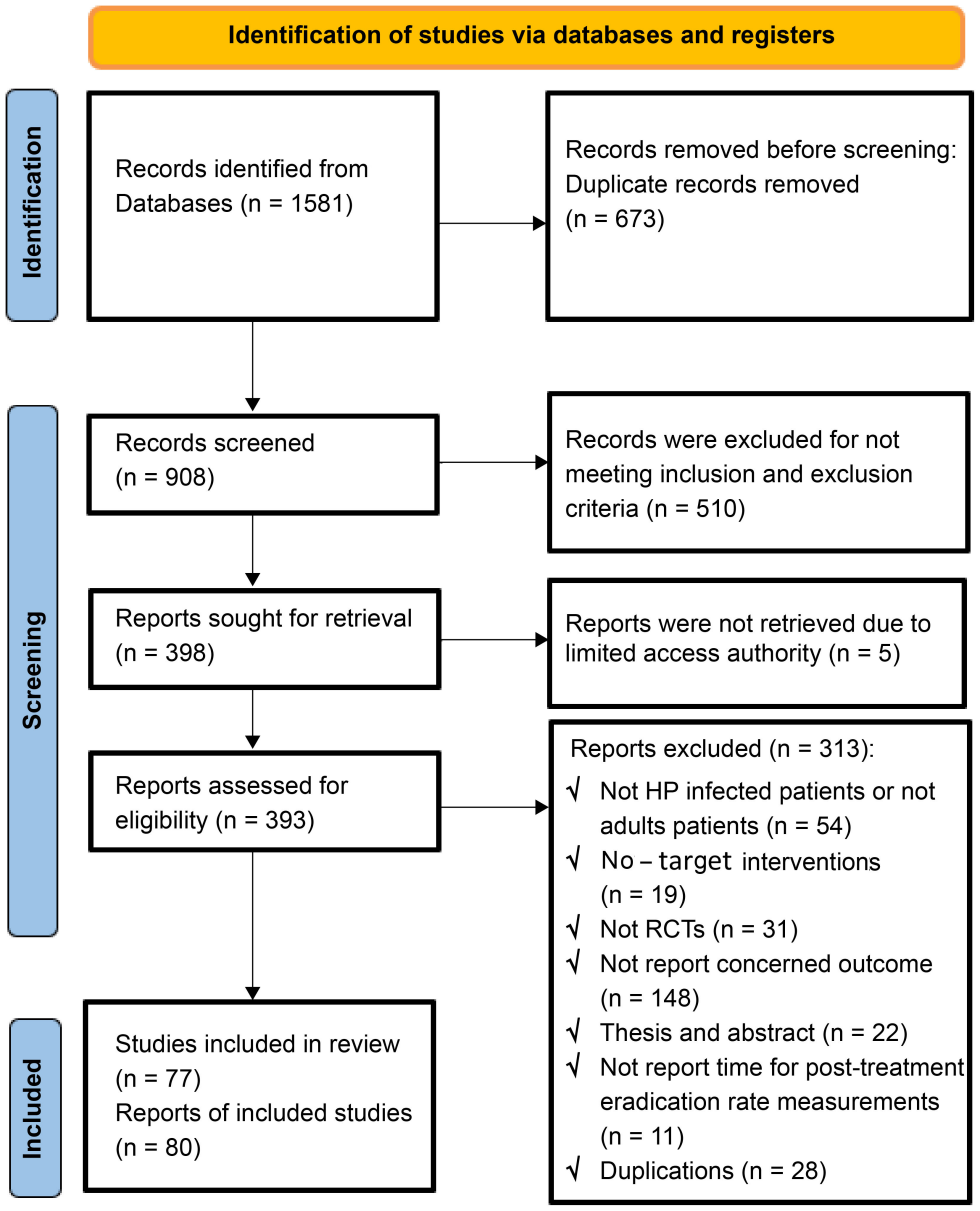
PRISMA flow diagram.

Our systematic search identified 1,581 articles, including 908 unique reports. After excluding reports on the basis of titles and abstracts, 393 full-text articles were retrieved for assessment. Among them, 77 RCTs (80 reports) (Murakami et al., 2016; Maruyama et al., 2017; Sue et al., 2018; Nct, 2019a; Nct, 2019b; Sue et al., 2019; Suzuki et al., 2020; Bunchorntavakul and Buranathawornsom, 2021; Chen, 2021; Suzuki et al., 2021a; Suzuki et al., 2021b; Ang et al., 2022; Chen et al., 2022; Chey et al., 2022; Choi et al., 2022; Deng et al., 2022; Huang and Lin, 2022; Huh et al., 2022; Kim et al., 2022; Lin et al., 2022; Wang et al., 2022; Wei et al., 2022; Zuberi et al., 2022; Duan et al., 2023; Han et al., 2023; Iqbal et al., 2023; Lai, 2023; Li GZ. et al., 2023; Li J. et al., 2023; Lu LF. et al., 2023; Lu ZX. et al., 2023; Miao et al., 2023; Pan et al., 2023; Peng et al., 2023; Qian et al., 2023; Ran et al., 2023; Ratana-Amornpin et al., 2023; Tang et al., 2023; Wang T. et al., 2023; Wang XL. et al., 2023; Xiong et al., 2023; Yan et al., 2023; Zhang et al., 2023; Zhong and Cao, 2023; Chen et al., 2024; Cheung et al., 2024; Duan, 2024; Gao, 2024; Gao et al., 2024; Guo et al., 2024; Huang et al., 2024; Hu et al., 2024; Jiang et al., 2024; Jin et al., 2024; Kong et al., 2024; Li HT. et al., 2024; Li MF. et al., 2024; Lin and Liang, 2024; Lin and Wan, 2024; Lin et al., 2024; Liu GH. et al., 2024; Liu HN. et al., 2024; Shang et al., 2024; Shen et al., 2024; Tan et al., 2024; Wang HT. et al., 2024; Wang SL. et al., 2024; Wang X. et al., 2024; Waqar et al., 2024; Xu et al., 2024; Yan et al., 2024; Yang et al., 2024; Yuan et al., 2024; Zhou BG. et al., 2024; Cai et al., 2025; Li et al., 2025; Song et al., 2025a; Wu and Yu, 2025; Xiao et al., 2025; Yan et al., 2025) met the inclusion criteria in the NMA, comprising a total of 19,950 patients (**Figure 1**).

### 3.2 Characteristics of studies and patients

A total of 77 RCTs involving 19,950 patients published between 2016 and 2025 were included (**Supplementary Table 1**). Publication activity increased notably in 2023 (24 RCTs) and 2024 (27 RCTs). The mean age of participants ranged from 26.2 to 73.6 years. The most frequently compared treatments were VPZ-based dual therapy vs. PPI-based quadruple therapy (31 RCTs), followed by VPZ-based quadruple therapy vs. PPI-based quadruple therapy (15 RCTs) and VPZ-based triple therapy vs. PPI-based triple therapy (9 RCTs). Other trials evaluated VPZ-based dual therapy against VPZ-based triple or quadruple therapy, TPZ-based therapies vs. PPI- or TPZ-based comparators, and variations in treatment dose or duration. Among the included studies, *H. pylori* eradication was primarily assessed using urea breath tests (13C- or 14C-UBT) in 72 studies. Three studies used stool antigen tests (HpSA) to define eradication. One study assessed eradication using a combination of endoscopy with 14C-UBT, and one study used either HpSA or 13C-UBT according to its protocol.

### 3.3 Quality assessment of included studies

**Figure 2:**
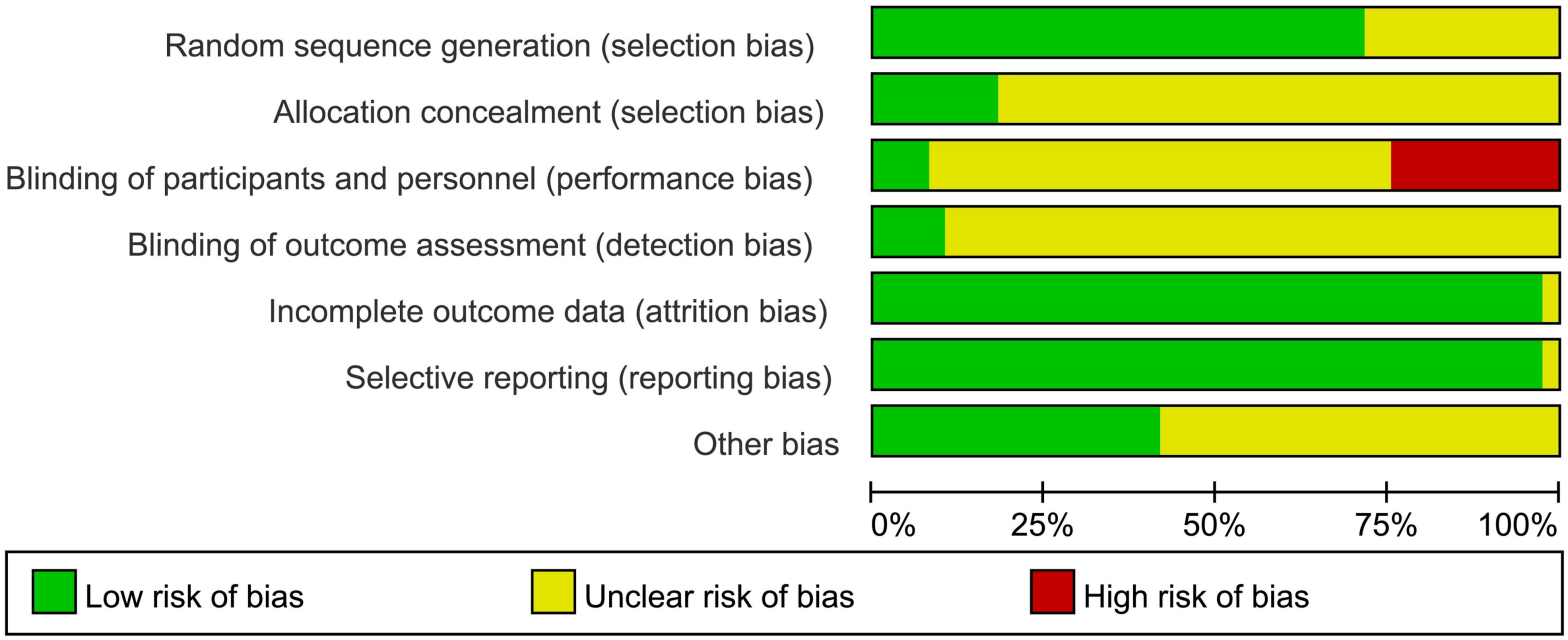
Risk of bias assessment results.

**Figure 2** summarises the methodological quality of the included RCTs, with detailed assessments provided in **Supplementary Figure 1**, **Supplementary Table 2**. Randomisation was adequate in 74% of trials, while allocation concealment was clearly reported in 19%. Blinding was inconsistently applied, and most trials had unclear risk for outcome assessment. Selective reporting, attrition, and other biases were generally low or unclear.

### 3.4 Eradication rate from proportional meta-analysis

For TPZ-based regimens, eradication was 88.45% with dual therapy (95% CI: 81.60–95.30%, 4 RCTs with 616 patients), 62.86% with triple therapy (95% CI: 55.24–70.03%, 1 RCT with 175 patients), and 86.84% with quadruple therapy (95% CI: 73.51–100.00%, 3 RCTs with 279 patients). For VPZ-based regimens, eradication was 88.15% with dual therapy (95% CI: 86.03–90.12%, 44 RCTs with 6,170 patients), 88.73% with triple therapy (95% CI: 84.49–91.92%, 16 RCTs with 1949 patients), and 91.94% with quadruple therapy (95% CI: 89.23–94.66%, 19 RCTs with 1,941 patients). Additionally, KEV-based quadruple therapy achieved 87.80% (95% CI: 83.45–91.36%) in a single RCT (287 patients). Overall, eradication rates ranked highest with VPZ quadruple therapy (91.94%) and lowest with TPZ triple therapy (62.86%). PPI-based regimens achieved modest eradication rates, with 81.83% for dual therapy, 76.02% for triple therapy, and 83.58% for quadruple therapy (**Supplementary Figures 2**-**9**). Fixed-effects sensitivity showed a generally similar pattern with narrower CIs (**Supplementary Figures 10**-**17**).

### 3.5 Eradication rates of various regimens

**Figure 3:**
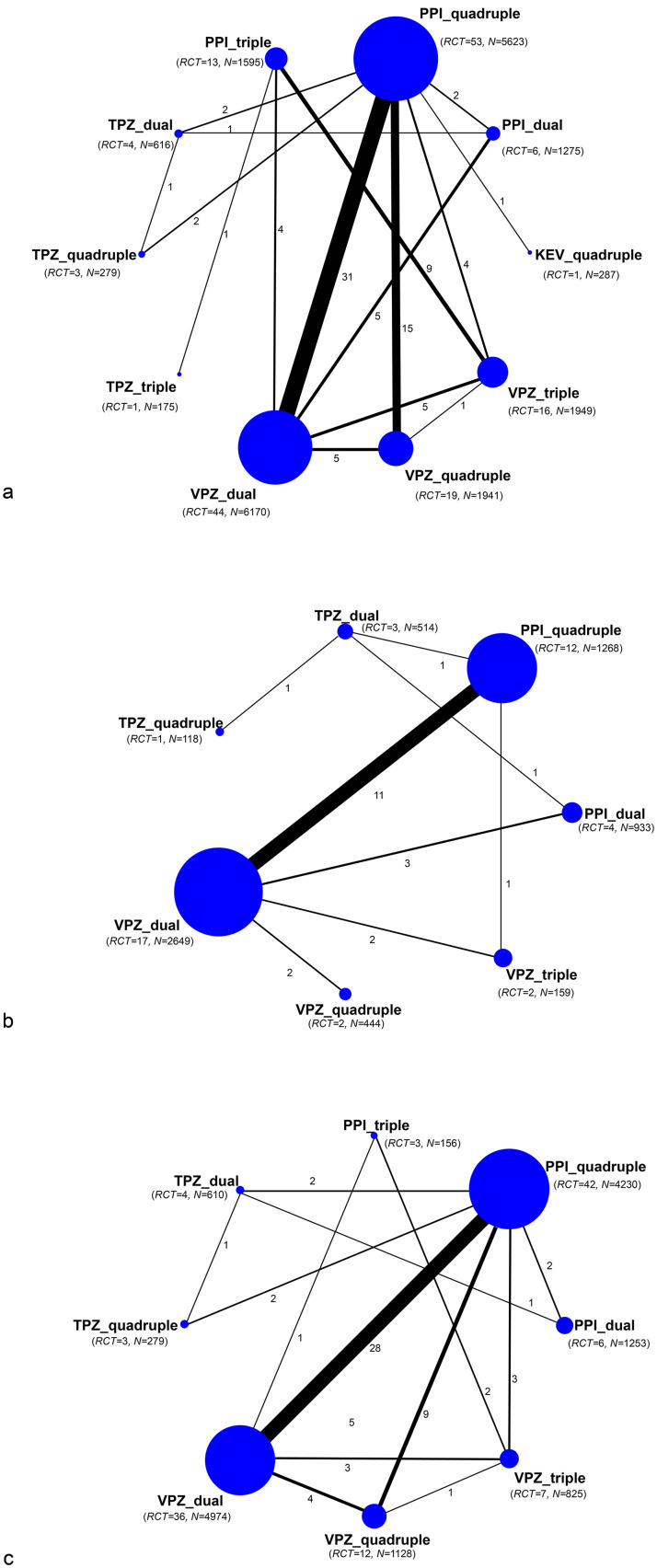
Network maps of comparisons of eradication treatments: **(A)** eradication rate, **(B)** compliance rate, and **(C)** adverse event rate.

A total of 77 RCTs (19,950 patients) involving 10 regimens reporting the *H. pylori* eradication rate were included, with 45 treatment comparisons (16 direct and 29 indirect, **Figure 3A**). For VPZ-based regimens, quadruple therapy resulted in a significantly higher eradication rate than dual therapy (OR = 1.47, 95% CI: 1.05–2.07). In addition, VPZ-based quadruple reported a higher eradication rate than TPZ-based triple (OR = 3.54, 95% CI: 1.21–10.65). We found no significant differences among other PCAB regimens (**Table 1**).

**Table 1:** League matrix of comparative efficacies of various regimens based on eradication rate.

**TPZ_dual**	1.02 (0.54, 1.92)	0.68 (0.35, 1.32)	0.42 (0.13, 1.4)	1.08 (0.52, 2.18)	0.38 (0.18, 0.81)	0.92 (0.29, 2.81)	1.04 (0.48, 2.28)	1.5 (0.76, 2.99)	0.6 (0.32, 1.09)
0.98 (0.52, 1.84)	**VPZ_dual**	0.66 (0.44, 1)	0.41 (0.15, 1.16)	1.05 (0.72, 1.51)	0.38 (0.24, 0.57)	0.89 (0.33, 2.35)	1.01 (0.48, 2.21)	1.47 (1.05, 2.07)	0.58 (0.47, 0.71)
1.47 (0.76, 2.86)	**1.51 (1, 2.26)**	**PPI_dual**	0.62 (0.2, 1.88)	1.58 (0.91, 2.71)	0.56 (0.31, 1)	1.34 (0.46, 3.81)	1.52 (0.67, 3.54)	2.2 (1.33, 3.73)	0.88 (0.57, 1.34)
2.36 (0.71, 7.97)	2.42 (0.86, 6.87)	1.6 (0.53, 4.95)	**TPZ_triple**	2.55 (0.91, 7.11)	0.91 (0.35, 2.35)	2.15 (0.52, 8.86)	2.45 (0.69, 8.96)	3.54 (1.21, 10.65)	1.41 (0.49, 4.01)
0.93 (0.46, 1.91)	0.95 (0.66, 1.38)	0.63 (0.37, 1.09)	0.39 (0.14, 1.09)	**VPZ_triple**	0.36 (0.24, 0.52)	0.85 (0.3, 2.36)	0.96 (0.43, 2.27)	1.39 (0.88, 2.26)	0.55 (0.37, 0.81)
**2.6 (1.24, 5.57)**	**2.66 (1.76, 4.12)**	**1.77 (1, 3.2)**	1.1 (0.43, 2.86)	**2.8 (1.93, 4.09)**	**PPI_triple**	2.38 (0.83, 6.8)	2.7 (1.16, 6.54)	3.9 (2.32, 6.73)	1.55 (0.99, 2.43)
1.09 (0.36, 3.49)	1.12 (0.43, 3.03)	0.74 (0.26, 2.16)	0.47 (0.11, 1.92)	1.18 (0.42, 3.34)	0.42 (0.15, 1.21)	**KEV_quadruple**	1.13 (0.34, 3.94)	1.63 (0.61, 4.6)	0.65 (0.25, 1.72)
0.96 (0.44, 2.09)	0.99 (0.45, 2.09)	0.66 (0.28, 1.49)	0.41 (0.11, 1.44)	1.04 (0.44, 2.34)	**0.37 (0.15, 0.86)**	0.88 (0.25, 2.91)	**TPZ_quadruple**	1.45 (0.65, 3.2)	0.57 (0.27, 1.19)
0.67 (0.33, 1.31)	**0.68 (0.48, 0.95)**	0.45 (0.27, 0.75)	**0.28 (0.09, 0.83)**	0.72 (0.44, 1.14)#	**0.26 (0.15, 0.43)**	0.61 (0.22, 1.64)	0.69 (0.31, 1.54)	**VPZ_quadruple**	0.4 (0.29, 0.54)
1.68 (0.92, 3.15)	**1.72 (1.41, 2.13)**	1.14 (0.75, 1.77)	0.71 (0.25, 2.04)	**1.81 (1.23, 2.67)**	0.65 (0.41, 1.01)	1.54 (0.58, 4.02)	1.74 (0.84, 3.73)	**2.52 (1.86, 3.5)**	**PPI_quadruple**

Odds ratio (95% confidence interval) reported. Statistically significant data presented in bold.

*TPZ_dual* Tegoprazan combined with one antibiotic; *VPZ_dual* Vonoprazan combined with one antibiotic; *PPI_dual* Proton pump inhibitor combined with one antibiotic; *TPZ_triple* Tegoprazan combined with two antibiotics; *VPZ_triple* Vonoprazan combined with two antibiotics; *PPI_triple* Proton pump inhibitor combined with two antibiotics; *KEV_quadruple* Keverprazan combined with two antibiotics and bismuth; *TPZ_quadruple* Tegoprazan combined with two antibiotics and bismuth; *VPZ_quadruple* Vonoprazan combined with two antibiotics and bismuth; *PPI_quadruple* Proton pump inhibitor combined with two antibiotics and bismuthver.

# local inconsistency.

**Figure 4:**
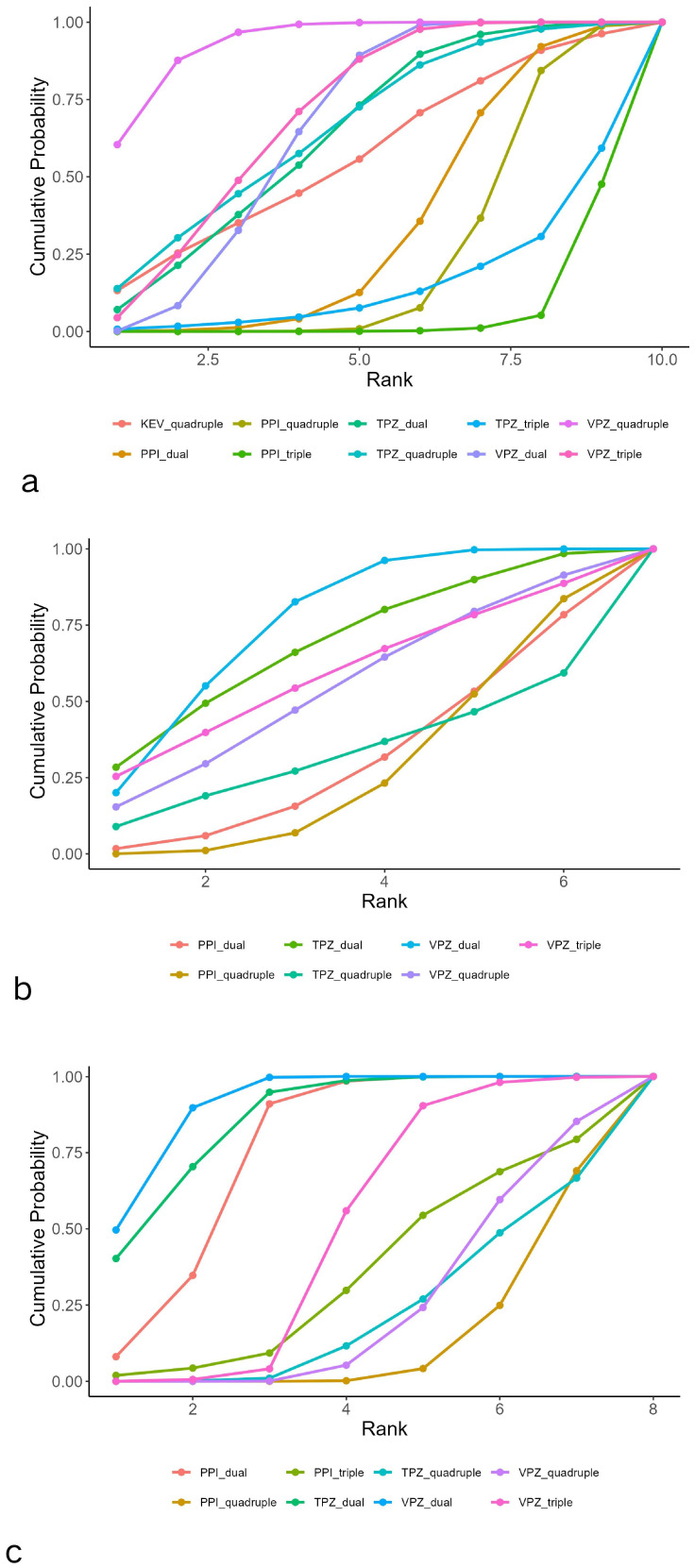
Cumulative rank probability plots of **(A)** eradication rate, **(B)** compliance rate, and **(C)** adverse event rate.

Rankograms and SUCRA values (**Figure 4A**) indicated that VPZ-based quadruple (93.78%) exhibited the most effective eradication, followed by VPZ-based triple (70.55%) and TPZ-based quadruple (66.22%), VPZ-based dual (66.03%), TPZ-based dual (64.16%), KEV-based quadruple (57.02%), PPI-based dual (35.06%) and quadruple (25.39%), TPZ-based triple (15.75%), and PPI-based triple therapy (6.03%). Notably, despite these differences in ranking probabilities, the lack of statistically significant differences among most pairwise comparisons suggests that these PCAB regimens may have comparable efficacy. Fixed-effects sensitivity analysis showed a broadly similar overall pattern, although some additional comparisons (e.g., among TPZ-based regimens) reached statistical significance, with slightly altered effect estimates and narrower CIs (**Supplementary Figure 18**, **Supplementary Table 3**).

### 3.6 Eradication rates of dual, triple, and quadruple PCAB-based regimens

Subgroup analyses across different PCAB-based regimens showed no statistically significant differences in eradication rates among regimens in dual (VPZ vs. TPZ), triple (VPZ vs. TPZ), or quadruple therapy (VPZ, TPZ, and KEV) (**Supplementary Figures 19**, **20**, **Supplementary Tables 4**-**6**). Rankograms and SUCRA values suggested that VPZ-based regimens had higher probabilities of being among the more effective treatments across dual, triple, and quadruple therapies. These findings should be interpreted with caution, as the absence of statistically significant differences in pairwise comparisons limits the reliability of the ranking results. Fixed-effects sensitivity analysis showed a broadly similar overall pattern, although the results showed a statistically significant advantage of VPZ-based triple therapy over TPZ-based triple therapy (**Supplementary Figure 21**, **Supplementary Tables 7**-**9**).

### 3.7 Eradication rates in treatment-naive and previously treated patients

In treatment-naive patients, reported in 51 RCTs with 15,931 patients (**Supplementary Figure 22A**), VPZ-based regimens consistently outperformed TPZ-based regimens. Among the VPZ-based regimens, quadruple (OR = 1.71) achieved higher eradication rates than dual. Compared with TPZ-based regimens, VPZ-quadruple outperformed TPZ-triple (OR = 3.31) and TPZ-quadruple (OR = 2.43) (**Supplementary Table 10**). SUCRA ranking (**Supplementary Figure 23A**) placed VPZ-based quadruple highest, followed by VPZ-based triple and VPZ-based dual. In previously treated patients, we found no significant differences between VPZ-based dual and quadruple regimens (6 RCTs with 566 patients, **Supplementary Figure 22B**, **Supplementary Table 11**). Ranking analyses (**Supplementary Figure 23B**) indicated VPZ-based quadruple as the top regimen, followed by VPZ-based dual, though pairwise comparison did not show any significant differences. Fixed-effects sensitivity analysis showed a broadly similar overall pattern, although more pairwise comparisons reached statistical significance, particularly among VPZ-based regimens in treatment-naive patients (**Supplementary Figure 24**, **Supplementary Tables 12**, **13**).

### 3.8 Eradication rates of 7-day, 10-day, and 14-day PCAB-based regimens

For 14-day regimens, a total of 53 RCTs involving 14,764 patients were included (**Supplementary Figure 25C**). Within VPZ-based regimens, quadruple therapy achieved a significantly higher eradication rate than dual therapy (OR = 1.43), while we found no significant differences between quadruple and triple, or between dual and triple (**Supplementary Table 14**). SUCRA (**Supplementary Figure 26C**) ranked VPZ-based quadruple highest, followed by VPZ-based dual and triple.

Shorter regimens were less frequently studied (**Supplementary Figures 25A, B**). Seven-day and 10-day data were reported in 7 RCTs (1,869 patients) and 2 RCTs (with 525 patients), respectively. We found no significant differences between PCAB-regimens in 7 or 10 days (**Supplementary Tables 14**, **15**). For 7-day regimens, SUCRA ranked VPZ-based triple highest, followed by VPZ-based dual and TPZ-based triple (**Supplementary Figure 26A**). Fixed-effects sensitivity analysis showed a similar overall pattern, although some additional differences emerged in shorter-duration regimens (**Supplementary Figure 27**, **Supplementary Tables 17**-**19**).

### 3.9 Eradication rates of PCAB-based regimens in different regions

Among the 77 included RCTs, 76 RCTs with 19,255 patients were conducted in Asia, resulting in nearly identical subgroup estimates to those in the overall analysis (**Supplementary Figure 28**). VPZ-based quadruple outperformed VPZ-based dual (OR = 1.46) and TPZ-based triple (OR = 4.14) (**Supplementary Table 20**). Ranking analysis placed VPZ-based quadruple highest, followed by VPZ-based dual and triple (**Supplementary Figure 30**).

Focusing on treatment-naive Chinese patients receiving 14-day regimens (**Supplementary Figure 29**), data from 13 RCTs with 9,139 patients (**Supplementary Table 21**) showed no significant differences between various PCAB-based regimens. Ranking analysis (SUCRA) placed VPZ-based quadruple highest (**Supplementary Figure 31**), followed by VPZ-based triple and dual, KEV-based quadruple, TPZ-based dual and quadruple, PPI-based dual, quadruple and triple. Fixed-effects sensitivity analysis showed a broadly similar overall pattern, although additional statistically significant differences emerged in regional subgroup analyses, particularly among treatment-naive Chinese patients (**Supplementary Figures 32**, **33**, **Supplementary Tables 22**, **23**).

### 3.10 Compliance rates of various regimens

Of the 77 included RCTs, only 20 RCTs including 6,085 patients reported compliance outcomes and were therefore included in the compliance network, indicating that approximately three-quarters of trials did not provide compliance data (**Figure 3B**). We found no significant differences between VPZ-based and TPZ-based regimens (**Supplementary Table 24**). Ranking by SUCRA indicated that VPZ-based dual had the highest adherence, followed by TPZ-based dual, VPZ-based triple, and VPZ-based quadruple (**Figure 4B**). However, these ranking results should be interpreted with caution due to the lack of significant differences among pairwise comparisons. Fixed-effects sensitivity analysis showed a similar overall pattern (**Supplementary Figure 34**, **Supplementary Table 25**).

### 3.11 Safety of various regimens

Fifty-four RCTs involving 13,455 patients were included in the NMA of total AEs (**Figure 3C**). VPZ-based and TPZ-based dual was associated with lower AEs than VPZ-based quadruple (OR = 0.41 and 0.42), and TPZ-based quadruple regimens (OR = 0.39 and 0.41, **Supplementary Table 26**). In addition, VPZ-based dual showed lower incidence of AEs than VPZ-based triple regimens (OR = 0.55). SUCRA rankings confirmed VPZ-based dual as the safest regimen, followed by TPZ-based dual (**Figure 4C**). Fixed-effects sensitivity analysis showed a similar overall pattern, although some additional differences emerged in TPZ-based dual and VPZ-based triple regimens (**Supplementary Figure 35**, **Supplementary Table 27**).

Regarding treatment discontinuation due to AEs, 21 RCTs with 7,238 patients were included (**Supplementary Figure 36**). We found no significant differences among regimens (**Supplementary Table 28**); details of the SUCRA analysis are provided in **Supplementary Figure 37**. Fixed-effect sensitivity analysis showed a similar pattern (**Supplementary Figure 38**, **Supplementary Table 29**).

### 3.12 Publication bias and consistency tests

Funnel plots showed asymmetry across several analyses, including overall, dual, triple, and quadruple therapy, treatment-naive patients, 14-day regimens, Asian populations, treatment-naive Chinese patients receiving 14-day therapy, and AEs, suggesting potential small-study effects (**Supplementary Figures 39**-**49**). Most comparisons showed no evidence of inconsistency. We identified significant inconsistency in the comparison between VPZ-based triple and quadruple therapy in the overall analysis, as well as in the treatment-naive and 14-day subgroups (*p* < 0.05, **Supplementary Figures 50**-**58**).

### 3.13 Heterogeneity and sensitivity analysis excluding high-risk RCTs

Overall heterogeneity across the network was moderate. Among PCAB-based regimens, heterogeneity ranged from moderate to substantial in several comparisons, including VPZ-based triple vs. quadruple therapy in the overall analysis (*I²* = 52.6%) and in 14-day regimens (*I²* = 67.4%), as well as VPZ-based quadruple vs. dual (*I²* = 53.2%) and triple vs. dual (*I²* = 54.2%) in compliance outcomes (**Supplementary Figures 59**-**69**). A random-effects model was applied to account for between-study variability.

Sensitivity analysis excluding studies with high risk of bias showed a broadly similar overall pattern. However, some pairwise comparisons changed, with an additional significant difference showing between TPZ-based dual and triple therapy, and the previously observed difference between VPZ-based quadruple and dual therapy no longer remaining significant (**Supplementary Figure 70**, **Supplementary Table 30**).

## 4 Discussion

This NMA synthesising 77 RCTs provides one of the most comprehensive and up-to-date comparative evaluations of the efficacy, safety, and compliance of TPZ-, VPZ-, KEV-, and PPI-based regimens for *H. pylori* eradication. Previous meta-analyses have examined this topic but with important limitations. The NMA published in 2024 primarily focused on comparisons between PCABs and PPIs in first-line therapy, without systematically differentiating between dual, triple, and quadruple regimens or including emerging PCABs such as TPZ and KEV (Ouyang et al., 2024). Similarly, the meta-analysis published in 2023 mainly performed pairwise comparisons of PCAB vs. PPI-based therapies and did not incorporate a full network structure to allow indirect comparisons across multiple regimen types (Shah et al., 2023). In contrast, the present study integrates a broader range of treatment strategies, including multiple PCAB agents and regimen combinations, and applies an NMA framework to enable comprehensive comparisons across regimens, treatment durations, and clinical settings. Overall, VPZ-based regimens, particularly quadruple therapy, achieved the highest eradication rates, while the PPI-based triple regimen was consistently the least effective. The VPZ-based dual regimen showed advantages in compliance and safety, suggesting it may provide a balance between efficacy and tolerability in selected patients. Subgroup analyses by regimen type, treatment history, treatment duration, and geography confirmed the robustness of the findings. The China-specific subgroup analysis was largely driven by the availability of data from Chinese studies, where treatment-naive patients and 14-day regimens were most frequently reported, reflecting real-world prescribing patterns rather than a pre-specified selective analytical focus.

Our findings demonstrate that PCAB-based regimens consistently achieved superior *H. pylori* eradication compared with PPI-based therapies, supporting the shift in guideline recommendations towards PCAB as the preferred acid-suppressive backbone (Fallone et al., 2016; Malfertheiner et al., 2022; Chey et al., 2024). Among the PCABs, VPZ-based regimens showed the most consistent and robust efficacy for *H. pylori* eradication across dual (87.25%), triple (88.60%), and quadruple (89.82%) regimens, regardless of treatment duration or geographic setting. In treatment-naïve Chinese populations receiving 14-day regimens, our network meta-analysis did not identify statistically significant differences among PCAB-based therapies in direct comparisons, although ranking probabilities suggested a consistent tendency favouring VPZ-containing regimens. This trend remained generally stable across sensitivity and subgroup analyses despite residual heterogeneity. A recent prospective multicenter RCT conducted in China further supported this observation, showing higher eradication rates with 14-day VPZ-based dual therapy than with TPZ-based dual therapy in a similar patient population and treatment setting (89.3% vs. 76.1%, *p* < 0.001) *(*Zhou et al., 2026). The observed tendency favouring VPZ-based regimens may be related to the more rapid and sustained acid suppression provided by VPZ, which enhances antibiotic activity and stability in the gastric environment (Hori et al., 2011; Aumpan et al., 2023; Elsabaawy et al., 2024; Kanu and Soldera, 2024; Song et al., 2025b).

As novel PCABs, TPZ and KEV similarly exhibit rapid absorption and potent acid suppression (Hu et al., 2022; Tan et al., 2023; Zhou et al., 2023). Our findings suggest that their eradication efficacy may be slightly lower than that of VPZ. This difference may be attributed to their shorter plasma half-life (approximately 3–6 hours) compared with VPZ (7–9 hours), suboptimal dosing in clinical studies, and potential variations in pharmacodynamic potency against the H^+^/K^+^-ATPase (Echizen, 2016; Choi, 2023; Zhou et al., 2023). Although most comparisons were consistent, significant inconsistency was observed between VPZ-based triple and quadruple therapies in the overall analysis and in certain subgroups (treatment-naive and 14-day regimens). This may reflect clinical and methodological heterogeneity across studies, including variations in antibiotic combinations, treatment duration, and patient characteristics. Therefore, the comparative efficacy between these regimens should be interpreted with caution, and the ranking results may be less reliable for these specific comparisons.

Our findings showed that the VPZ-based dual regimen (typically VPZ + amoxicillin) is a particularly attractive option, combining high eradication rates (87.25%) with favourable safety, tolerability, and compliance. Its simplified dosing and lower pill burden reduce gastrointestinal side effects and enhance adherence, which may explain why the VPZ-based dual regimen is increasingly included in international guidelines as an empirical option, particularly in settings where antibiotic susceptibility testing is unavailable (Malfertheiner et al., 2022; Chey et al., 2024; Patel et al., 2024). Conversely, the VPZ-based quadruple therapy demonstrates very high efficacy and is listed as an alternative in Chinese guidelines (Zhou et al., 2022), particularly in regions with high dual resistance to clarithromycin and metronidazole (Tungtrongchitr et al., 2024). However, quadruple regimens are often associated with reduced adherence due to regimen complexity, bismuth-related AEs, and the burden of multiple antibiotics (Mesquita et al., 2022; Reum Choe et al., 2024; Zhou J. et al., 2024). In the present study, AEs were primarily analysed as a composite outcome, and detailed classification was limited by heterogeneous reporting across trials. Although treatment discontinuation due to AEs did not differ significantly among regimens, safety findings should still be interpreted with caution given study heterogeneity. Moreover, the limited number of standardised RCTs and the heterogeneity across available studies underscore the need for further validation in large, well-designed RCTs. Notably, the advantage of VPZ appears most pronounced in clarithromycin-containing regimens, where acid stability is critical for antimicrobial activity. In contrast, in salvage settings where clarithromycin is replaced by metronidazole or sitafloxacin, the incremental advantage of VPZ over PPIs is less consistent or statistically non-significant (Benito et al., 2024). This suggests that while VPZ-based therapy is clearly preferred for first-line treatment, its role in rescue therapy requires further clarification. However, emerging evidence from non-Asian settings may help address this gap. A recent prospective multicentre cohort study in Egypt evaluated a 14-day VPZ–amoxicillin–metronidazole triple regimen in patients with confirmed dual resistance to clarithromycin and levofloxacin who had failed prior eradication therapy (Semeya et al., 2026). Despite high local metronidazole resistance (>85%), the per-protocol eradication rate reached 89.5%, closely aligning with the pooled estimate for VPZ-based triple therapy in the present NMA (88.73%). This finding provides important support for the effectiveness of VPZ-based regimens in high-resistance salvage settings beyond Asia.

The strengths of this study include a large sample size, comprehensive NMA methodology incorporating both direct and indirect comparisons, and extensive subgroup analyses by treatment duration, history, and population. However, several limitations should be acknowledged. First, although all included studies were RCTs, clinical and methodological heterogeneity across trials, including differences in antibiotic combinations, treatment protocols, outcome assessment methods for *H. pylori* eradication, and study design characteristics and quality, may have influenced the robustness of indirect comparisons. Second, the discrepancy between SUCRA-based rankings and the lack of statistically significant differences in most pairwise comparisons highlights the need for cautious interpretation of treatment hierarchies in the NMA. In addition, sample size was limited for certain interventions. Notably, evidence for KEV and TPZ was derived from a single RCT including 287 patients and a limited number of trials, respectively. Some subgroup analyses, such as the 10-day treatment duration subgroup, were based on only two trials. These findings should therefore be interpreted with caution. Third, potential sources of bias should be considered, including publication bias, heterogeneity in regional antibiotic resistance, and inconsistencies identified in node-splitting analyses. Additionally, the compliance network was based on a limited subset of studies, due to the requirement for a uniform definition (≥80% medication adherence). This may have reduced the number of eligible studies and the network size, potentially affecting the precision and robustness of the compliance-related estimates. Furthermore, pooling different PPIs into a single node despite known differences in their pharmacokinetic and pharmacodynamic profiles may have introduced additional heterogeneity and potentially masked differences between individual agents. Finally, most of the available RCTs (76/77) were conducted in Asia, which limits the applicability of the results to Western populations. Resistance patterns differ between regions: metronidazole resistance is generally lower in Western countries, but clarithromycin resistance remains common (Savoldi et al., 2018; Nyssen et al., 2022), which may potentially influence the relative benefit of VPZ-based regimens. Importantly, a substantial proportion of the included studies originated from China, particularly in the VPZ-based dual therapy arm. Therefore, observed differences in treatment effects may partly reflect regional antibiotic resistance patterns, prescribing practices, and healthcare context rather than a universally generalisable effects. As a result, caution is warranted when extrapolating these findings to non-Asian populations. Future studies in Western settings are urgently needed to validate these results, inform global guideline development, and optimise individualised therapy across diverse resistance landscapes.

These findings support VPZ-based regimens, particularly 14-day quadruple therapy, as effective options for *H. pylori* eradication in Asia, including China. VPZ-based dual therapy may be considered in patients where adherence or safety is a concern. PPI-based triple therapy appears to be less effective and may be less suitable as a first-line option in this setting. Treatment history should be taken into account when selecting eradication regiments, with VPZ-based regimens favoured even in previously treated patients.

## 5 Conclusion

The VPZ-based quadruple regimen was associated with the highest eradication rates for *H. pylori*, particularly in Asian populations and treatment-naive Chinese patients receiving 14-day regimens. The VPZ-based dual regimen appeared to offer a favourable balance of efficacy, safety and compliance. However, these findings should be interpreted as evidence from RCTs and indirect comparisons, rather than definitive clinical superiority. Future research should focus on evaluating PCAB-based regimens in non-Asian populations, assessing their role in second-line and salvage therapy, and further optimising treatment strategies in the context of diverse antibiotic resistance patterns across regions.

## Data Availability

Publicly available datasets were analyzed in this study. Data were obtained from published RCTs (listed in references). No public repository or accession number is available.
