# Supplementary material for: Comparing potassium-competitive acid blocker-based therapies for Helicobacter pylori infection: A Bayesian network meta-analysis of 77 randomised controlled trials
Source: Front Physiol. 2026 Jul 7;17:1843686. doi: 10.3389/fphys.2026.1843686 (PMC13384841; doi:10.3389/fphys.2026.1843686)
Supplement: Supplementary file 1 [file DataSheet1.docx]

**Appendix**

Table of Contents

[Appendix 1 Search strategy 2](#_Toc9017)

[Appendix 2 Characteristics and quality assessment 6](#_Toc24077)

[Appendix 3 Proportional meta-analysis results-Random 19](#_Toc13461)

[Appendix 4 Proportional meta-analysis results-Fix 25](#_Toc27299)

[Appendix 5 Eradication rates for various PCAB-based regimens-Fixed 30](#_Toc28539)

[Appendix 6 Subgroup analysis of eradication rates for different PCAB-based regimens-Random 32](#_Toc12313)

[Appendix 7 Subgroup analysis of eradication rates for different PCAB-based regimens-Fix 36](#_Toc29756)

[Appendix 8 Subgroup analysis of eradication rates in patients with different treatment histories-Random 39](#_Toc31626)

[Appendix 9 Subgroup analysis of eradication rates in patients with different treatment histories-Fix 42](#_Toc31084)

[Appendix 10 Subgroup analysis of eradication rates for various treatment durations-Random 44](#_Toc22420)

[Appendix 11 Subgroup analysis of eradication rates for various treatment durations-Fix 48](#_Toc15393)

[Appendix 12 Subgroup analysis of eradication rates in different regions-Random 51](#_Toc15902)

[Appendix 13 Subgroup analysis of eradication rates in different regions-Fix 57](#_Toc25384)

[Appendix 14 Compliance rate of various regimens-Random 60](#_Toc1341)

[Appendix 15 Compliance rate of various regimens-Fix 60](#_Toc29907)

[Appendix 16 Adverse events of various regimens-Random 61](#_Toc18499)

[Appendix 17 Adverse events of various regimens-Fix 62](#_Toc15441)

[Appendix 18 Treatment discontinuation due to adverse events of various regimens - Random 63](#_Toc22074)

[Appendix 19 Treatment discontinuation due to adverse events of various regimens -Fix 65](#_Toc8881)

[Appendix 20 Funnel plots-Random 66](#_Toc14691)

[Appendix 21 Inconsistency (Node-splitting) results-Random 76](#_Toc24825)

[Appendix 22 Heterogeneity test-Random 87](#_Toc18280)

[Appendix 23 Sensitivity analysis in the overall population 119](#_Toc23543)

# **Appendix 1 Search strategy**

- PubMed

#1. "Helicobacter Infections"[Mesh] OR "Helicobacter pylori"[Mesh] OR Helicobacter[tw] OR Campylobacter[tw] OR "H. pylori infection*"[tw]

#2. potassium competitive acid blocker*[tw] OR Potassium ion competitive acid blocker*[tw] OR "P-CABs"[tw] OR PCAB[tw] OR "P-CAB"[tw] OR "1-(5-(2-fluorophenyl)-1-(pyridin-3-ylsulfonyl)-1H-pyrrol-3-yl)-N-methylmethanamine" [Supplementary Concept] OR Vonoprazan[tw] OR "TAK 438"[tw] OR TAK438[tw] OR Vorapaxar[tw] OR vonosap[tw] OR takecab[tw] OR vocinti[tw] OR vonopion[tw] OR vonosap[tw] OR "tegoprazan" [Supplementary Concept] OR tegoprazan[tw] OR Keverprazan[tw] OR "fexuprazan" [Supplementary Concept] OR fexuprazan[tw] OR abeprazan[tw] OR "dwp 14012"[tw] OR "YH 1885" [Supplementary Concept] OR "YH 1885"[tw] OR Revaprazan[tw] OR Xinoprazan[tw] OR Konoprazan[tw] OR Zastaprazan[tw] OR "linaprazan" [Supplementary Concept] OR Linaprazan[tw]

#3. #1 AND #2

#4. ("controlled clinical trial"[pt] OR "Controlled Clinical Trials as Topic"[MeSH] OR "Random Allocation"[MeSH] OR "Double-Blind Method"[MeSH] OR "single-blind method"[MeSH] OR "Control Groups"[MeSH] OR "cross-over studies"[MeSH] OR random*[tiab] OR placebo[tiab] OR trial[tiab] OR groups[tiab] OR crossover[tiab] OR cross-over[tiab] OR single blind*[tiab] OR double blind*[tiab] OR triple blind*[tiab] OR Factorial design*[tiab]) NOT ("Animals"[Mesh] NOT ("Humans"[Mesh] AND "Animals"[Mesh]))

#5. #3 and #4

- EMBASE

#1. 'Helicobacter infection'/exp OR 'Helicobacter pylori'/exp OR (Helicobacter OR Campylobacter OR "H. pylori infection*"):ab,ti,kw

#2. 'potassium competitive acid blocker'/exp OR 'vonoprazan'/exp OR 'tegoprazan'/exp OR 'fexuprazan'/exp OR 'revaprazan'/exp OR 'zastaprazan'/exp OR 'linaprazan'/exp OR ((potassium NEAR/3 competitive NEAR/3 "acid blocker*") OR "P-CABs" OR PCAB OR "P-CAB" OR Vonoprazan OR "TAK 438" OR TAK438 OR Vorapaxar OR vonosap OR takecab OR vocinti OR vonopion OR vonosap OR tegoprazan OR Keverprazan OR fexuprazan OR abeprazan OR "dwp 14012" OR "YH 1885" OR Revaprazan OR Xinoprazan OR Konoprazan OR Zastaprazan OR Linaprazan):ab,ti,kw

#3. #1 AND #2

#4. ('controlled clinical trial'/exp OR 'Controlled Clinical Trial (Topic)'/exp OR 'double blind procedure'/de OR 'control group'/de OR 'crossover procedure'/de OR 'single blind procedure'/de OR 'triple blind procedure'/de OR 'placebo'/de OR 'randomization'/exp OR (random* OR trial OR groups OR placebo* OR crossover OR "cross-over" OR "Factorial design*" OR ((Doubl* OR Singl* OR tripl*) NEAR/2 Blind*)):ab,ti,kw) NOT (('nonhuman'/exp OR 'animal'/exp) NOT 'human'/exp)

#5. #3 and #4

- WOS

#1. TS=(Helicobacter OR Campylobacter OR "H. pylori infection*")

#2. TS=((potassium NEAR/3 competitive NEAR/3 "acid blocker*") OR "P-CABs" OR PCAB OR "P-CAB" OR Vonoprazan OR "TAK 438" OR TAK438 OR Vorapaxar OR vonosap OR takecab OR vocinti OR vonopion OR vonosap OR tegoprazan OR Keverprazan OR fexuprazan OR abeprazan OR "dwp 14012" OR "YH 1885" OR Revaprazan OR Xinoprazan OR Konoprazan OR Zastaprazan OR Linaprazan)

#3. #1 AND #2

#4. (TS=(((controlled OR Random*) NEAR/5 (Trial OR study)) OR "Random Allocation*" OR Randomization* OR "Double Blind" OR "single blind" OR "Control Group*" OR "Controlled Group*" OR "cross over" OR placebo* OR crossover OR random* OR "Factorial design*" OR ((Doubl* OR Singl* OR tripl*) NEAR/2 Blind*)) OR TI=("trial" OR "RCT" OR "groups" OR "group"))

#5. #3 and #4

- Cochrane

#1 MeSH descriptor: [Helicobacter Infections] explode all trees

#2 MeSH descriptor: [Helicobacter pylori] explode all trees

#3 (Helicobacter OR Campylobacter OR "H. pylori infection*"):ti,ab,kw

#4 #1 or #2 or #3

#5 ((potassium NEAR/3 competitive NEAR/3 "acid blocker*") OR "P-CABs" OR PCAB OR "P-CAB" OR Vonoprazan OR "TAK 438" OR TAK438 OR Vorapaxar OR vonosap OR takecab OR vocinti OR vonopion OR vonosap OR tegoprazan OR Keverprazan OR fexuprazan OR abeprazan OR "dwp 14012" OR "YH 1885" OR Revaprazan OR Xinoprazan OR Konoprazan OR Zastaprazan OR Linaprazan):ti,ab,kw

#6 #4 and #5

- CNKI(期刊、学位、会议，中英文扩展：是，中文)

(SU%=(幽门螺杆菌+幽门螺旋杆菌+幽门螺旋菌)*感染+hp感染+HP阳性+螺旋菌阳性+螺杆菌阳性+螺旋菌感染+螺杆菌感染 OR TKA % (幽门螺杆菌+幽门螺旋杆菌+幽门螺旋菌)*感染+hp感染+HP阳性+螺旋菌阳性+螺杆菌阳性+螺旋菌感染+螺杆菌感染) AND (SU%= 钾竞争性酸阻滞剂+钾离子竞争性酸阻滞剂+钾竞争性酸抑制剂+钾离子竞争性酸抑制剂+"P-CAB"+"P-CABs"+vonoprazan +伏诺拉生+沃克+Vocinti+ "TAK-438"+富马酸伏诺拉生片+富马酸伏诺拉生+伏诺拉生片+替戈拉生+Tegoprazan+特戈拉赞+凯普拉生+Keverprazan+克维拉赞+非苏拉生+Fexuprazan+非苏拉赞+瑞伐拉赞+Revaprazan+ 信诺拉生+Xinoprazan +柯诺拉赞+Konoprazan OR TKA % 钾竞争性酸阻滞剂+钾离子竞争性酸阻滞剂+钾竞争性酸抑制剂+钾离子竞争性酸抑制剂+"P-CAB"+"P-CABs"+vonoprazan +伏诺拉生+沃克+Vocinti+ "TAK-438"+富马酸伏诺拉生片+富马酸伏诺拉生+伏诺拉生片+替戈拉生+Tegoprazan+特戈拉赞+凯普拉生+Keverprazan+克维拉赞+非苏拉生+Fexuprazan+非苏拉赞+瑞伐拉赞+Revaprazan+ 信诺拉生+Xinoprazan +柯诺拉赞+Konoprazan) and (SU%=随机+盲法+双盲+单盲+三盲+交叉+RCT OR TKA=随机+盲法+双盲+单盲+三盲+交叉+RCT)

- 万方(期刊、学位、会议)

((主题:("幽门螺杆菌" OR "幽门螺旋杆菌" OR "幽门螺旋菌") and 主题:("感染")) or 主题:("hp感染" OR "HP阳性" OR "螺旋菌阳性" OR "螺杆菌阳性" OR "螺旋菌感染" OR "螺杆菌感染")) and 主题:("钾竞争性酸阻滞剂" OR "钾离子竞争性酸阻滞剂" OR "钾竞争性酸抑制剂" OR "钾离子竞争性酸抑制剂" OR "P-CAB" OR "P-CABs" OR "vonoprazan" OR "伏诺拉生" OR "沃克" OR "Vocinti" OR "TAK-438" OR "替戈拉生" OR "Tegoprazan" OR "特戈拉赞" OR "凯普拉生" OR "Keverprazan" OR "克维拉赞" OR "非苏拉生" OR "Fexuprazan" OR "非苏拉赞" OR "瑞伐拉赞" OR "Revaprazan" OR "信诺拉生" OR "Xinoprazan" OR "柯诺拉赞" OR "Konoprazan") and 主题:("随机" OR "盲法" OR "双盲" OR "单盲" OR "三盲" OR "交叉" OR "RCT")

- CBM

((( "幽门螺杆菌"[常用字段:智能] OR "幽门螺旋杆菌"[常用字段:智能] OR "幽门螺旋菌"[常用字段:智能]) AND "感染"[常用字段:智能]) OR ( "hp感染"[常用字段:智能] OR "HP阳性"[常用字段:智能] OR "螺旋菌阳性"[常用字段:智能] OR "螺杆菌阳性"[常用字段:智能] OR "螺旋菌感染"[常用字段:智能] OR "螺杆菌感染"[常用字段:智能])) AND ("钾竞争性酸阻滞剂"[常用字段:智能] OR "钾离子竞争性酸阻滞剂"[常用字段:智能] OR "钾竞争性酸抑制剂"[常用字段:智能] OR "钾离子竞争性酸抑制剂"[常用字段:智能] OR "P-CAB"[常用字段:智能] OR "P-CABs"[常用字段:智能] OR "vonoprazan"[常用字段:智能] OR "伏诺拉生"[常用字段:智能] OR "沃克"[常用字段:智能] OR "Vocinti"[常用字段:智能] OR "TAK-438"[常用字段:智能] OR "富马酸伏诺拉生片"[常用字段:智能] OR "富马酸伏诺拉生"[常用字段:智能] OR "伏诺拉生片"[常用字段:智能] OR "替戈拉生"[常用字段:智能] OR "Tegoprazan"[常用字段:智能] OR "特戈拉赞"[常用字段:智能] OR "凯普拉生"[常用字段:智能] OR "Keverprazan"[常用字段:智能] OR "克维拉赞"[常用字段:智能] OR "非苏拉生"[常用字段:智能] OR "Fexuprazan"[常用字段:智能] OR "非苏拉赞"[常用字段:智能] OR "瑞伐拉赞"[常用字段:智能] OR "Revaprazan"[常用字段:智能] OR "信诺拉生"[常用字段:智能] OR "Xinoprazan"[常用字段:智能] OR "柯诺拉赞"[常用字段:智能] OR "Konoprazan"[常用字段:智能]) AND ("随机对照试验"[不加权:扩展] OR "随机对照试验(主题)"[不加权:扩展] OR "非随机对照试验(主题)"[不加权:扩展] OR "随机"[常用字段:智能] OR "盲法"[常用字段:智能] OR "双盲"[常用字段:智能] OR "单盲"[常用字段:智能] OR "三盲"[常用字段:智能] OR "交叉"[常用字段:智能] OR "RCT"[常用字段:智能])

- VIP

(U=(幽门螺杆菌+幽门螺旋杆菌+幽门螺旋菌)*感染+hp感染+HP阳性+螺旋菌阳性+螺杆菌阳性+螺旋菌感染+螺杆菌感染) AND (U= 钾竞争性酸阻滞剂+钾离子竞争性酸阻滞剂+钾竞争性酸抑制剂+钾离子竞争性酸抑制剂+伏诺拉生+沃克+替戈拉生+特戈拉赞+凯普拉生+克维拉赞+非苏拉生+非苏拉赞+瑞伐拉赞+信诺拉生+柯诺拉赞) AND (U=随机+盲法+双盲+单盲+三盲+交叉+RCT)

# Appendix 2 Characteristics and quality assessment

## Table S1 Characteristics of studies

| Study ID | Sample size | Treatment naïve or experienced | Diagnostic tool | Mean Age (years) | Intervention regimen | Outcome detection | Follow up period | Eradication rate |
| --- | --- | --- | --- | --- | --- | --- | --- | --- |
|  |  |  |  |  |  |  |  |  |
| Amornpin 2023[25] | 100 | Treatment naïve | RUT, Histological identification of H. pylori | 14-day VPZ_dual: 50.4 ± 13.5; 14-day VPZ_triple therapy: 54.1 ± 12.8; 7-day VPZ_triple therapy: 56.6 ± 10.8; 14-day VPZ_quadruple: 55.5 ± 14.2 | 14-day VPZ_dual vs.  14 day VPZ_triple vs.  7 day VPZ_triple vs.  14-day VPZ_quadruple | 13C-UBT | 4 weeks | 14/21 vs. 16/27 vs. 24/26 vs. 25/26 |
| Ang 2022[26] | 244 | Treatment naïve | 13-UBT or a positive rapid urease test on gastric biopsies | VPZ_triple group: 51.5±14.7;  PPI_triple group: 52.0±14.6 | 7 day VPZ_triple vs.  7 day PPI_triple vs.  14 day VPZ_triple vs.  14 day PPI_triple | 13C-UBT | 4 weeks | 104/119 vs. 10/14 vs. 110/125 vs. 13/17 |
| Bunchorntavakul 2021[27] | 122 | Treatment naïve | RUT, histology obtained by endoscopy with biopsy samples | VPZ_triple: 54.21 ± 12.3; PPI_triple: 56.79 ± 13.25 | VPZ_triple vs.  PPI_triple | 14C-UBT | 6-8 weeks | 59/61 vs. 54/60 |
| Cai 2025[28] | 112 | Not reported | 13C-UBT | TPZ_quadruple: 42.61±4.37;  PPI_quadruple: 42.58±4.34 | TPZ_quadruple vs. PPI_quadruple | 13C-UBT | 4 weeks | 55/56 vs. 47/56 |
| Chen 2021[29] | 100 | Not reported | The diagnosis was confirmed by electronic gastroscopy and gastric mucosal biopsy | VPZ_dual: 42.9±8.1;  PPI_triple: 44.1±8.7 | VPZ_dual vs.  PPI_triple | 13C-UBT | 6 weeks | 50/53 vs. 37/47 |
| Chen 2022[30] | 126 | Treatment naïve | 14C-UBT | VPZ_dual: 41.52 ± 11.34； PPI_quadruple: 40.67 ± 12.41 | VPZ_dual vs. PPI_quadruple | 14C-UBT | 4 weeks | 53/63 vs. 60/63 |
| Chen 2024[31] | 135 | Not reported | 13C-UBT | VPZ_dual: 44.87 ± 2.00；  VPZ_quadruple: 42.44 ± 1.83；PPI_quadruple: 40.78 ± 1.49 | VPZ_dualvs.VPZ_quadrupe vs.PPI_quadruple | 13C-UBT | 4 weeks | 38/45 vs. 38/45 vs. 38/45 |
| Cheung 2024[32] | 298 | Treatment naïve | 13C-UBT | VPZ_dual: 35.7±8.4；  VPZ_triple: 36.3±9.4；  PPI_quadruple: 35.0±7.4 | VPZ_dual vs.VPZ_triple vs.PPI_quadruple | 13C-UBT | 4-6 weeks | 96/100 vs. 94/98 vs. 92/100 |
| Chey 2022[33,34] | 992 | Treatment naïve | 13C-UBT | VPZ_dual: 51.8 ±13.6; VPZ_triple: 50.6±13.9; PPI_triple : 51.8±13.5 | VPZ_dual vs. VPZ_triple vs. PPI_triple | 13C-UBT | 4 weeks | 208/265 vs. 222/262 vs. 201/255 |
| Choi2022[35] | 350 | Treatment naïve | 13C-UBT | TPZ_triple: 54.71±11.24;  PPI_triple: 53.19±10.88 | TPZ_triple vs.PPI_triple | 13C-UBT | 4-8 weeks | 110/175 vs. 106/175 |
| Deng 2022[36] | 222 | Treatment naïve | 13C-UBT | VPZ_quadruple: 44.88±12.44; PPI_quadruple: 47.70±14.03 | VPZ_quadruple vs. PPI_quadruple | 13C-UBT | 4 weeks | 97/113 vs. 82/119 |
| Duan 2023[37] | 177 | Treatment naïve | 13C-UBT,14C-UBT, Endoscopic biopsy with histopathological examination | VPZ_dual: 42.5±11.5；  PPI_dual: 46.7±13.8;  PPI_quadruple: 47.8±15.3 | VPZ_dual vs.PPI_dual vs.PPI_quadruple | 13C-UBT or 14C-UBT | 4 weeks | 49/58 vs. 50/62 vs. 48/57 |
| Duan 2024[38] | 100 | Not reported | 13C-UBT, 14C-UBT | Total: 34.27±5.02 | VPZ_quadruple vs.PPI_quadruple | UBT | 4 weeks | 48/50 vs. 37/50 |
| Gao 2024a[39] | 300 | Treatment naïve | 13C-UBT | VPZ_dual: 46.6±12.9；  PPI_quadruple: 47.4±12.8 | VPZ_dual vs.PPI_quadruple | 13C-UBT | 6 weeks | 138/150 vs. 134/150 |
| Gao 2024b[40] | 200 | Treatment naïve | RUT, 13C-UBT,14C-UBT，HpSA | VPZ_quadruple: 37.36±5.42;  PPI_quadruple: 37.22±5.23 | VPZ_quadruple vs.PPI_quadruple | 13C-UBT | 4 weeks | 98/100 vs. 90/100 |
| Guo 2024[41] | 1120 | Treatment naïve | 13C-UBT | VPZ_dual:44.3±5.7;  PPI_dual:45.2±4.6;  PPI_quadruple:44.8±5.1 | VPZ_dual vs.PPI_dual vs. PPI_quadruple | 13C-UBT | 4 weeks | 476/560 vs. 231/280 vs. 236/280 |
| Han 2023[42] | 690 | Treatment naïve | 13C-UBT or 14C-UBT or immuno-histochemical staining of biopsy samples | VPZ_dual: 42.5±12.5;  VPZ_triple: 41.8±12.3 | VPZ_dual vs. VPZ_dual | 13C-UBT or 4C-UBT | 4 weeks | 308/345 vs. 293/345 |
| Hu 2023[43] | 190 | Treatment naïve | UBT, Histology examination | VPZ_dual:41.9 ± 13.3;  VPZ_quadruple:38.4 ± 10.2 | VPZ_dual vs.VPZ_quadruple | 13C-UBT or fecal antigen test | 4-12 weeks | 83/95 vs. 88/95 |
| Huang 2022[44] | 120 | Not reported | 14C-UBT | PPI_quadruple: 50.82 ± 4.83; VPZ_quadruple: 51.36 ± 3.94 | VPZ_quadruple vs. PPI_quadruple | 14C-UBT | 4 weeks | 39/40 vs. 32/40 |
| Huang 2024[45] | 140 | Treatment naïve | 13C-UBT,14C-UBT，rapid urease test | 10-day VPZ_dual:45.52±11.26；  14-day VPZ_dual: 45.69 ± 12.39；PPI_quadruple: 43.84 ± 14.26 | VPZ_dual vs.PPI_quadruple | 13C-UBT or 14C-UBT | 6 weeks | 96/103 vs. 94/102 vs. 81/101 |
| IQBAL 2023[46] | 400 | Not reported | Fecal antigen test | VPZ_dual:37.78 ± 10.84; PPI_triple :32.23 ± 15.53 | VPZ_dual vs. PPI_triple | fecal antigen test | 4 weeks | 178/200 vs. 139/200 |
| Jiang 2024[47] | 400 | Treatment naïve | 14C-UBT | VPZ_dual: 43.7 ± 15.1；  PPI_quadruple: 44.5 ± 15.0 | VPZ_dual vs.PPI_quadruple | 14C-UBT | 4 weeks | 188/200 vs. 174/200 |
| Jin 2024[48] | 240 | Treatment naïve | 13C-UBT | 7-day VPZ_dual: 42.35±11.90;  14-day VPZ_dual:41.91±10.24; PPI_quadruple:41. 13 ± 11. 91 | VPZ_dual vs.PPI_quadruple | 13C-UBT or HpSA | 4 weeks | 61/80 vs.67/80 vs. 64/80 |
| Ki 2022[49] | 30 | Not reported | UBT | VPZ_quadruple: 32.8 ± 6.9;  PPI_quadruple: 33.3 ± 8.6 | VPZ_quadruple vs. PPI_quadruple | 13C-UBT | 4 weeks | 12/15 vs. 14/15 |
| Kim 2023[50] | 217 | Treatment naïve | RUT, Histological ex-amination | TPZ_quadruple : 58.0±11.3;  PPI_quadruple: 57.9±10.0 | TPZ_quadruple vs.PPI_quadruple | 13C-UBT | 4 weeks | 84/105 vs. 82/106 |
| Kong 2024[51] | 368 | Treatment naïve | Pathological histological examination, RUT, 13C-UBT, 14C-UBT | TPZ_dual:45 (36–54);  PPI_dual: 42 (33–55) | TPZ_dual vs.PPI_dual | 13C-UBT | 6 weeks | 158/184 vs. 155/184 |
| Lai 2023[52] | 120 | Not reported | 13C-UBT | PPI_quadruple : 48.72±9.31; VPZ_dual: 49.44±9.50 | PPI_quadruple vs. VPZ_dual | 13C-UBT | 4 weeks | 50/60 vs. 57/60 |
| Li 2023a[53] | 130 | Treatment naïve | 13C-UBT | Not reported | VPZ_dual vs. VPZ_quadruple | 13C-UBT | 4 weeks | 57/65 vs. 58/65 |
| Li 2023b[54] | 224 | Treatment naïve | 13C-UBT or 14C-UBT | VPZ_dual: 45.85±13.97;  PPI_quadruple: 42.67+12.61 | VPZ_dual vs. PPI_quadruple | UBT | 4 weeks | 58/75 vs. 59/75 |
| Li 2024a[55] | 100 | Not reported | 14C-UBT | VPZ_quadruple: 49.67±5.47;  PPI_quadruple: 48.41±5.28 | VPZ_quadruple vs.PPI_quadruple | 14C-UBT | 8 weeks | 45/50 vs. 37/50 |
| Li 2024b[56] | 200 | Not reported | 13C-UBT,14C-UBT | TPZ_dual: 42.5±1.4;  PPI_quadruple: 42.3±1.5 | TPZ_dual vs.PPI_quadruple | 14C-UBT | 4 weeks | 97/100 vs. 88/100 |
| Li 2025[57] | 700 | Treatment naïve | 13C-UBT,14C-UBT | VPZ_dual: 38 (33–51);  VPZ_quadruple: 39 (30.8–53.0) | VPZ_dual vs.VPZ_quadruple | 13C-UBT or 14C-UBT | 4 weeks | 266/350 VS. 302/350 |
| Lin 2022[58] | 230 | Treatment naïve | 13C-UBT or 14C-UBT | Not reported | High dose VPZ_dual vs. low dose VPZ_dual vs. VPZ_triple | UBT | 4 weeks | 54/85 vs. 49/84 vs. 37/61 |
| Lin 2024a[59] | 321 | Not reported | 13C-UBT,14C-UBT | TPZ_dual: 41.50±12.67；  PPI_quadruple: 41.22±13.47 | TPZ_dual vs.PPI_quadruple | 13C-UBT or 14C-UBT | 6 weeks | 184/214 vs. 91/107 |
| Lin 2024b[60] | 118 | Treatment naïve | 14C-UBT | VPZ_dual: 43.5±9.2;  PPI_quadruple: 44.1±8.8 | VPZ_dual vs.PPI_quadruple | 14C-UBT | 4 weeks | 50/54 vs. 45/56 |
| Lin 2024c[61] | 110 | Treatment naïve | 13C-UBT | VPZ_dual: 45.0±11.6;  PPI_quadruple: 47.9±9.6 | VPZ_dual vs.PPI_quadruple | 13C-UBT | 4-6 weeks | 48/55 vs. 44/55 |
| Liu 2024a[62] | 236 | Treatment naïve | 13C-UBT | TPZ_dual: 26.2±9.6;  TPZ_quadruple: 27.3±8.6 | TPZ_dual vs.TPZ_quadruple | 13C-UBT | 4-6 weeks | 99/118 vs. 96/118 |
| Liu 2024b[63] | 189 | Treatment naïve | 14C-UBT | VPZ_dual: 44.6±8.7;  PPI_quadruple: 43.8±9.3 | VPZ_dual vs.PPI_quadruple | 14C-UBT | 4-6 weeks | 91/100 vs. 79/100 |
| Lu 2023a[64] | 234 | Treatment naïve | Histochemical staining, tissue culture, 14C- UBT, 13C-UBT | 10 day VPZ_quadruple: 37.14 ±19.65; 14 day VPZ_quadruple: 35.88 ±11.51; PPI_quadruple: 36.54 ±10.96; | 10 day VPZ_quadruple vs. 14 day VPZ_quadruple vs. PPI_quadruple | 13C­UBT | 6-8 weeks | 75/78 vs. 74/78 vs. 73/78 |
| Lu 2023b[65] | 60 | Not reported | 13C-UBT or 14C-UBT | VPZ_dual: 45.36±5.74 ;  PPI_quadruple: 45.46±5.62 | VPZ_dual vs. PPI_quadruple | 14C-UBT | 12 weeks | 31/30 vs. 21/30 |
| Maruyama 2017[66] | 141 | Treatment naïve | 13C-UBT | Not reported | VPZ_triple vs. PPI_triple | 13C-UBT | 8 weeks | 69/72 vs. 48/69 |
| Miao 2023[67] | 44 | Not reported | 13C-UBT | VPZ_quadruple: 34.5; PPI_quadruple: 31.6 | VPZ_quadruple vs. PPI_quadruple | 13C-UBT | 4 weeks | 20/20 vs. 17/18 |
| Murakami 2016[68] | 650 | Treatment naïve | RUT, culture, 13C-UBT and/or the stool H pylori antigen test. | VPZ_triple group: 55.2±12.3； PPI_triple group: 53.9±12.9 | VPZ_triple vs. PPI--triple | 13C-UBT | 4 weeks | 300/324 vs. 243/320 |
| NCT04198363[69] | 510 | Not reported | 13C-UBT | Vonoprazan 20 mg: 39.5±12.29;  Esomeprazole 20 mg: 38.3±12.13; | VPZ_quadruple vs.PPI_quadruple | 13C-UBT | 4 weeks | 210/242 vs. 208/240 |
| Pan 2023[70] | 1085 | Treatment naïve | 13C-UBT, Gastroscopic pathological examination | VPZ_dual: 42.5±8.3;  PPI_quadruple: 44.2±9.5 | VPZ_dual vs. PPI_quadruple | 13C-UBT | 4 weeks | 498/537 vs. 447/548 |
| Peng 2023[71] | 316 | Treatment naïve | 13C-UBT or pathological examination | VPZ_dual: 40.1±12.1;  PPI_triple: 41.9±12.6； | VPZ_dual vs. PPI_quadruple | 13C-UBT | 4 weeks | 142/158 vs. 128/158 |
| Qian 2023[72] | 375 | Treatment naïve | 13C-UBT, RUT, Histopathology; | PPI_quadruple: 43.30 ± 13.93; VPZ_dual: 42.70± 12.96; | high-dose VPZ_dual vs. low dose VPZ_dual vs. PPI_quadruple | 13C-UBT | 4 weeks | 110/125 vs. 217/250 |
| Ran 2023[73] | 240 | Treatment naïve | 13C-UBT | PPI_quadruple:4 1.1 3±11.91;  14 day VPZ_quadruple:41.91±10.24;  7 day VPZ_quadruple: 42.3 5±11.90 | PPI_quadruple vs.  14 day VPZ_quadruple vs.  7 day VPZ_quadruple | 13C-UBT | 4-6 weeks | 64/80 vs. 67/80 vs. 61/80 |
| Shang 2024[74] | 150 | Treatment naïve | 13C-UBT | VPZ_dual: 47.06±12.63;  PPI_quadruple: 45.15±14.25 | VPZ_dual vs.PPI_quadruple | 13C-UBT | 4 weeks | 64/75 vs. 57/75 |
| Shen 2024[75] | 80 | Treatment naïve | EGD; Histopathological examination | VPZ_triple : 42.20±4.30;  PPI_triple: 43.30±4.05 | VPZ_triple vs.PPI_triple | 13C-UBT | 4 weeks | 37/40 vs. 28/40 |
| Song 2025[76] | 510 | Mix | 13C-UBT | VPZ_quadruple: 39.5±12.3；  PPI_quadruple: 38.3±12.1 | VPZ_quadruple vs.  PPI_quadruple | 13C-UBT | 4 weeks | 219/256 vs. 217/254 |
| Sue 2018[77] | 147 | Treatment naïve | Detection of anti-H. pylori IgG antibodies; the rapid urease test，bacterial culture， pathology (histology); or the UBT | CAM-susceptible: VPZ_triple : 64.3 ± 12.3; PPI_triple :61.9 ± 13.3 CAM-resistant: VPZ_triple:64.0 ± 11.4 | VPZ_triple vs. PPI_triple vs. VPZ_triple | UBT | 8 weeks | 48/55 vs. 39/51 vs. 34/41 |
| Sue 2019[78] | 63 | Treatment relapsed | UBT, H. pylori stool antigen test, detection of anti-H. pylori IgG, rapid urease test or H. pylori culture | VPZ_triple : 62.4 ± 14.1; PPI_triple: 64.0 ± 12.3; | VPZ_triple vs. PPI_triple | UBT | 4 weeks | 25/33 vs. 16/30 |
| Suzuki 2020[79-81] | 335 | Treatment naïve | Biopsy of the gastric mucosa， The culture was considered positive if one or more colonies showed Gram negativity, urease, oxidase, catalase, and spiral or curved rods in morphology | VPZ_dual: 61.2±11.5;  VPZ_triple : 61.3±10.4 | VPZ_dual vs. VPZ_triple | 13C-UBT | 4 weeks | 142/168 vs. 149/167 |
| Tan 2024[82] | 576 | Treatment naïve | 13C-UBT | KEV_quadruple : 39.3 ± 11.3；PPI_quadruple: 40.6 ± 12.4 | KEV_quadruple vs.PPI_quadruple | 13C-UBT | 4 weeks | 252/287 vs. 236/286 |
| Tang 2023[83] | 110 | Treatment naïve | 13C-UBT | VPZ_quadruple: 50.3±12.0;  PPI_quadruple: 48.2±11.4 | VPZ_quadruple vs.PPI_quadruple | 13C-UBT | 4 weeks | 52/54 vs. 46/56 |
| Wang 2022[84] | 84 | Not reported | 14C-UBT | PPI_quadruple: 39.67 ±7.74； VPZ_triple : 42.56±5.88 | VPZ_triple vs. PPI_quadruple | Electronic gastroscopy and "C" breath test were negative | 4 weeks | 39/42 vs. 36/42 |
| Wang 2023a[85] | 120 | Not reported | 13C-UBT | PPI -quadruple group : 73.6±5.9； VPZ_dual: 72.5±5.8 | VPZ_dual vs. PPI_quadruple | 13C-UBT | 4-8 weeks | 53/60 vs. 44/60 |
| Wang 2023b[86] | 151 | Treatment naïve | 13C-UBT | Total: 44.3±9.8 | VPZ_dual vs. PPI_quadruple | 13C-UBT | 4–6weeks | 70/74 vs. 67/77 |
| Wang 2024a[87] | 243 | Mix | Positive by rapid urease test , 13C-UBT,14C-UBT,Hp antigen test | VPZ_dual: 43.04±12.27;  PPI_dual group: 41.09±12.20 | VPZ_dual vs.PPI_dual | 13C-UBT or 14C-UBT | 4 weeks | 103/122 vs. 96/121 |
| Wang 2024b[88] | 120 | Treatment experienced | 13C-UBT | VPZ_dual: 42.80±12.69;  PPI_quadruple: 38.85±13.04 | VPZ_dual vs.PPI_quadruple | 13C-UBT | 4 weeks | 54/60 vs. 51/60 |
| Wang 2024c[89] | 216 | Treatment naïve | 13C-UBT | VPZ_triple: 49.04±10.58;  PPI_quadruple: 47.81±10.33 | VPZ_triple vs.  PPI_quadruple | 13C-UBT | 4 weeks | 98/105 vs. 95/101 |
| Waqar 2023[90] | 122 | Treatment naïve | Positive for H. pylori by stool antigen test | VPZ_triple : 40.98±12.13；  PPI_triple: 38.40±12.25 | VPZ_triple vs.PPI_triple | Fecal antigen test | 4 weeks | 58/61 vs. 57/61 |
| Wei 2022[91] | 130 | Treatment relapsed | 13C-UBT | VPZ_dual: 40.6±12.8; PPI_triple: 39.6±10.2 | VPZ_dual vs. PPI_triple | 13C-UBT | 4 weeks | 56/65 vs. 52/65 |
| Wu 2025[92] | 100 | Treatment naïve | 13C-UBT,14C-UBT | VPZ_dual: 42.28±5.51;  PPI_quadruple: 41.37±5.68 | VPZ_dual vs.PPI_quadruple | 14C-UBT | 4 weeks | 47/50 vs. 38/50 |
| Xiao 2025[93] | 200 | Treatment naïve | 13C-UBT | VPZ_dual: 52.36±11.34;  PPI_quadruple: 3.95±12.93 | VPZ_dual vs.PPI_quadruple | 13C-UBT | 4 weeks | 90/100 vs. 79/100 |
| Xiong 2023[94] | 85 | Treatment relapsed | 13C-UBT | VPZ_dual: 28.2±7.2;  PPI_quadruple: 27.7±6.6 | VPZ_dual vs. PPI_quadruple | 13C-UBT | 4 weeks | 32/42 vs. 31/43 |
| Xu 2024[95] | 63 | Not reported | 13C-UBT | VPZ_dual: 58.44土5.28;  PPI_quadruple: 58.35士5.09 | VPZ_dual vs.PPI_quadruple | 13C-UBT | 4-6 weeks | 33/33 vs. 23/30 |
| Yan 2023[96] | 314 | Treatment naïve | UBT, Histology examination, or positive bacterial culture | VPZ_dual: 38.10 ± 12.37; PPI_quadruple: 38.64 ± 13.60; | VPZ_dual vs. PPI_quadruple | 13C-UBT | 4 weeks | 135/157 vs. 140/157 |
| Yan 2024[97] | 314 | Treatment naïve | UBT, Histology examination, and positive bacterial culture. | VPZ_dual: 38.10±12.37；  PPI_quadruple: 38.64±13.60 | VPZ_dual vs.PPI_quadruple | 13C-UBT | 4-12 weeks | 135/157 vs. 140/157 |
| Yan 2025[98] | 80 | Treatment experienced | 14C-UBT,Esophagogastroduodenoscopy (EGD) | VPZ_dual: 32.6±2.4;  PPI_quadruple: 32.5±2.6 | VPZ_dual vs.PPI_quadruple | 14C-UBT | 4 weeks | 38/40 vs. 32/40 |
| Yang 2024[13] | 210 | Treatment naïve | 14C-UBT | VPZ_dual: 43.24±13.28;  PPI_quadruple: 43.43±12.13 | VPZ_dual vs.PPI_quadruple | 14C-UBT | 4-6 weeks | 95/106 vs. 92/104 |
| Yuan 2024[99] | 92 | Refractory | 14C-UBT | VPZ_quadruple: 38.6±9.5;  PPI_quadruple: 39.4±10.0 | VPZ_quadruple vs.PPI_quadruple | 13C-UBT | 4 weeks | 41/47 vs. 26/45 |
| Zhang 2023[100] | 640 | Treatment naïve | 13C-UBT or 14C-UBT | VPZ_triple : 42.07 ± 13.06;  PPI_quadruple: 42.44 ± 14.37 | VPZ_triple vs. PPI_quadruple | 14C-UBT | 5 weeks | 281/320 vs. 248/320 |
| Zhong 2023[101] | 120 | Mix | Pathological examination by gastroscopy or 14C-UBT | Total: 37.89±5.38 | VPZ_quadruple vs. PPI_quadruple | 14C-UBT | 4-6 weeks | 58/60 vs. 51/60 |
| Zhou 2024[102] | 570 | Treatment naïve | 13C-UBT,14C-UBT | VPZ_dual: 46.87 ± 12.06；  PPI_dual: 47.66 ± 11.92 | VPZ_dual vs.PPI_dual | 13C-UBT or 14C-UBT | 4-8 weeks | 245/287 vs. 217/283 |
| Zuberi 2022[103] | 179 | Not reported | Helicobacter Pylori Stool Antigen; Histopathology on Giemsa Stain | PPI_triple: 40.2 ±9.8;  VPZ_dual: 41.4 ±10.6 | PPI_triple vs. VPZ_dual e | Hp antigen test | 4 weeks | 86/92 vs. 73/87 |

Notes: EGD, Esophagogastroduodenoscopy; KEV, Keverprazan; PPI, Proton Pump Inhibitor; TPZ, Tegoprazan; RUT, Rapid urease test; UBT, Urea breath test; VPZ, vonoprazan.

## Table S2 Quality assessment results

| **Study ID (Author + Year)** | **RANDOMISATION** | **ALLOCATION CONCEALMENT** | **BLINDING OF PARTICIPANTS AND INVESTIGATORS** | **BLINDING OF OUTCOME ASSESSMENT** | **SELECTIVE REPORT OF OUTCOMES** | **STUDY ATTRITION** | **OTHER** |
| --- | --- | --- | --- | --- | --- | --- | --- |
| Amornpin 2023[25] | Low | Low | High | Unclear | Low | Low | Unclear |
| Ang 2022[26] | Low | Low | High | Unclear | Low | Low | Low |
| Bunchorntavakul 2021[27] | Unclear | Unclear | Unclear | Unclear | Low | Low | Unclear |
| Cai 2025[28] | Low | Unclear | Unclear | Unclear | Low | Low | Unclear |
| Chen 2021[29] | Unclear | Unclear | Unclear | Unclear | Low | Low | Unclear |
| Chen 2022[30] | Low | Unclear | Unclear | Unclear | Low | Low | Unclear |
| Chen 2024[31] | Low | Low | High | Low | Low | Low | Low |
| Cheung 2024[32] | Low | Low | Unclear | Unclear | Low | Low | Low |
| Chey 2022[33,34] | Low | Unclear | Unclear | Unclear | Low | Low | Unclear |
| Choi2022[35] | Low | Low | Low | Unclear | Low | Low | Low |
| Deng 2022[36] | Low | Unclear | Unclear | Unclear | Low | Low | Low |
| Duan 2023[37] | Low | Unclear | Unclear | Unclear | Low | Low | Unclear |
| Duan 2024[38] | Unclear | Unclear | Unclear | Unclear | Low | Low | Unclear |
| Gao 2024a[39] | Low | Low | High | Low | Low | Low | Low |
| Gao 2024b[40] | Low | Unclear | Unclear | Unclear | Low | Low | Unclear |
| Guo 2024[41] | Low | Unclear | Unclear | Unclear | Low | Low | Low |
| Han 2023[42] | Low | Unclear | Unclear | Unclear | Low | Low | Low |
| Hu 2023[43] | Low | Unclear | High | Unclear | Low | Low | Low |
| Huang 2022[44] | Unclear | Unclear | Unclear | Unclear | Low | Low | Low |
| Huang 2024[45] | Low | Unclear | High | Unclear | Low | Low | Low |
| IQBAL 2023[46] | Unclear | Unclear | Unclear | Unclear | Low | Low | Unclear |
| Jiang 2024[47] | Low | Unclear | Unclear | Unclear | Low | Low | Low |
| Jin 2024[48] | Low | Unclear | Unclear | Unclear | Low | Low | Unclear |
| Ki 2022[49] | Unclear | Unclear | Low | Unclear | Low | Low | Unclear |
| Kim 2023[50] | Low | Low | Low | Unclear | Low | Low | Unclear |
| Kong 2024[51] | Low | Low | High | Unclear | Low | Low | Low |
| Lai 2023[52] | Low | Unclear | Unclear | Unclear | Low | Low | Unclear |
| Li 2023a[53] | Low | Unclear | Unclear | Unclear | Low | Low | Unclear |
| Li 2023b[54] | Low | Unclear | Unclear | Unclear | Low | Low | Low |
| Li 2024a[55] | Low | Unclear | Unclear | Unclear | Low | Low | Unclear |
| Li 2024b[56] | Low | Unclear | Unclear | Unclear | Low | Low | Unclear |
| Li 2025[57] | Low | Unclear | High | Unclear | Low | Low | Low |
| Lin 2022[58] | Low | Unclear | High | Low | Low | Low | Low |
| Lin 2024a[59] | Low | Low | High | Low | Low | Low | Low |
| Lin 2024b[60] | Unclear | Unclear | Unclear | Unclear | Low | Low | Unclear |
| Lin 2024c[61] | Low | Unclear | Unclear | Unclear | Low | Low | Unclear |
| Liu 2024a[62] | Low | Unclear | High | Unclear | Low | Low | Low |
| Liu 2024b[63] | Low | Unclear | Unclear | Unclear | Low | Low | Unclear |
| Lu 2023a[64] | Low | Unclear | High | Low | Low | Low | Low |
| Lu 2023b[65] | Unclear | Unclear | Unclear | Unclear | Low | Low | Unclear |
| Maruyama 2017[66] | Unclear | Unclear | High | Unclear | Low | Low | Low |
| Miao 2023[67] | Unclear | Unclear | Unclear | Unclear | Low | Low | Unclear |
| Murakami 2016[68] | Low | Unclear | Low | Unclear | Low | Low | Unclear |
| NCT04198363[69] | Unclear | Unclear | Unclear | Unclear | Low | Low | Low |
| Pan 2023[70] | Unclear | Unclear | Unclear | Unclear | Low | Low | Unclear |
| Peng 2023[71] | Low | Low | Unclear | Unclear | Low | Low | Low |
| Qian 2023[72] | Low | Unclear | Unclear | Unclear | Low | Low | Low |
| Ran 2023[73] | Low | Unclear | Unclear | Unclear | Low | Low | Unclear |
| Shang 2024[74] | Low | Unclear | Unclear | Unclear | Low | Low | Unclear |
| Shen 2024[75] | Low | Unclear | Unclear | Unclear | Low | Low | Unclear |
| Song 2025[76] | Low | Low | Low | Unclear | Low | Low | Unclear |
| Sue 2018[77] | Low | Unclear | High | Unclear | Low | Low | Low |
| Sue 2019[78] | Low | Unclear | High | Low | Low | Low | Low |
| Suzuki 2020[79-81] | Low | Unclear | High | Unclear | Low | Low | Unclear |
| Tan 2024[82] | Low | Low | Low | Low | Low | Low | Low |
| Tang 2023[83] | Unclear | Unclear | Unclear | Unclear | Low | Low | Unclear |
| Wang 2022[84] | Unclear | Unclear | Unclear | Unclear | Low | Low | Unclear |
| Wang 2023a[85] | Unclear | Unclear | Unclear | Unclear | Low | Low | Unclear |
| Wang 2023b[86] | Low | Low | Unclear | Unclear | Low | Low | Low |
| Wang 2024a[87] | Low | Unclear | Unclear | Unclear | Low | Low | Unclear |
| Wang 2024b[88] | Low | Unclear | Unclear | Unclear | Low | Low | Unclear |
| Wang 2024c[89] | Unclear | Unclear | Unclear | Unclear | Low | Low | Unclear |
| Waqar 2023[90] | Low | Unclear | High | Unclear | Low | Low | Low |
| Wei 2022[91] | Low | Unclear | Unclear | Unclear | Low | Low | Unclear |
| Wu 2025[92] | Low | Unclear | Unclear | Unclear | Low | Low | Unclear |
| Xiao 2025[93] | Low | Unclear | Unclear | Unclear | Low | Low | Unclear |
| Xiong 2023[94] | Low | Unclear | Unclear | Unclear | Low | Low | Unclear |
| Xu 2024[95] | Unclear | Unclear | Unclear | Unclear | Low | Low | Unclear |
| Yan 2023[96] | Low | Unclear | High | Unclear | Low | Low | Low |
| Yan 2024[97] | Low | Low | High | Unclear | Low | Low | Low |
| Yan 2025[98] | Unclear | Unclear | Unclear | Unclear | Low | Low | Unclear |
| Yang 2024[13] | Unclear | Unclear | Unclear | Unclear | Low | Low | Unclear |
| Yuan 2024[99] | Low | Unclear | Unclear | Unclear | Low | Low | Unclear |
| Zhang 2023[100] | Low | Unclear | Unclear | Unclear | Low | Low | Low |
| Zhong 2023[101] | Unclear | Unclear | Unclear | Unclear | Low | Low | Unclear |
| Zhou 2024[102] | Low | Low | High | Low | Low | Low | Low |
| Zuberi 2022[103] | Low | Unclear | Unclear | Unclear | Low | Low | Low |


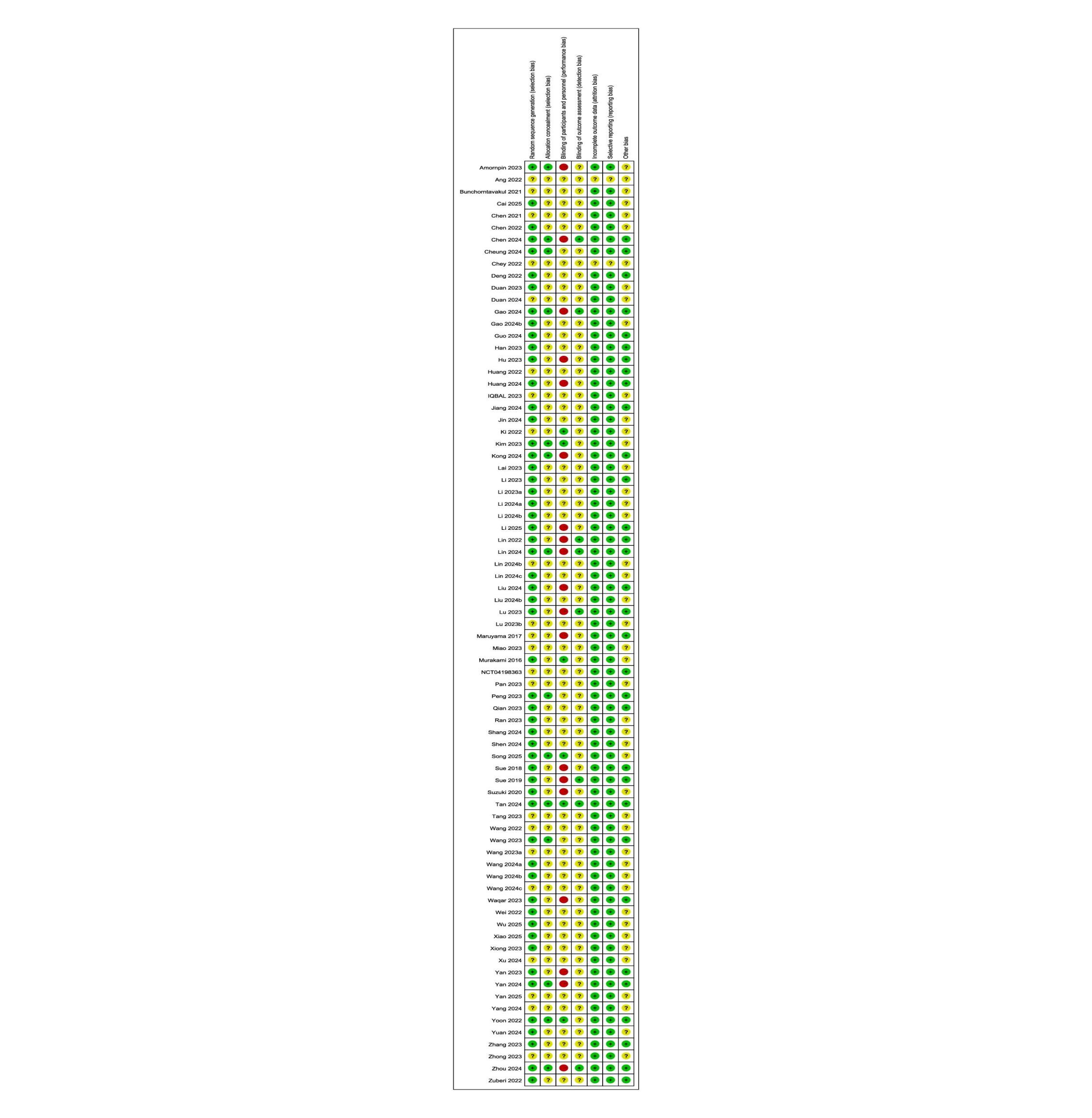


## Figure S1 Summary of risk of bias results of included studies

# Appendix 3 Proportional meta-analysis results-Random


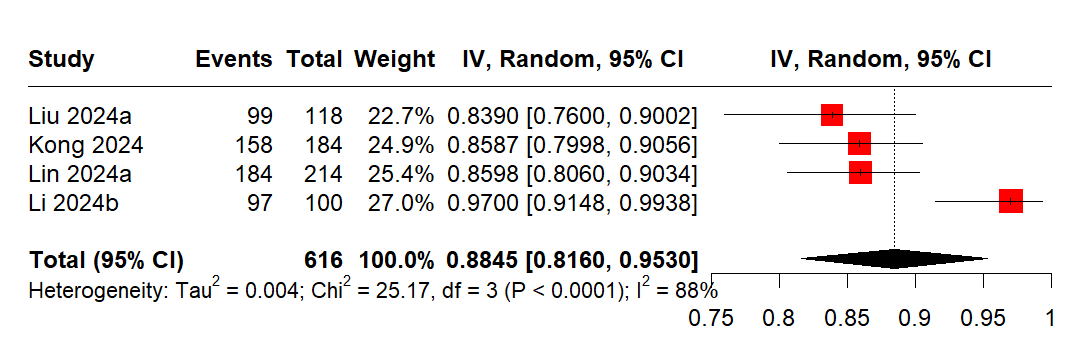


## Figure S 2 Pooled eradication rate for tegoprazan dual regimen


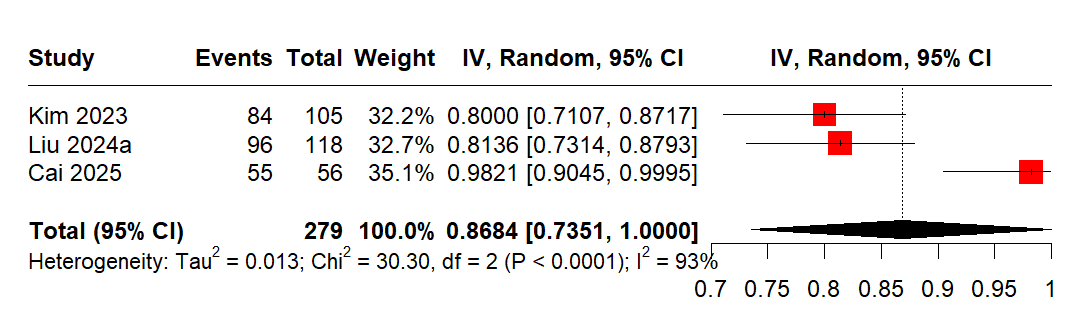


## Figure S 3 Pooled eradication rate for tegoprazan quadruple regimen


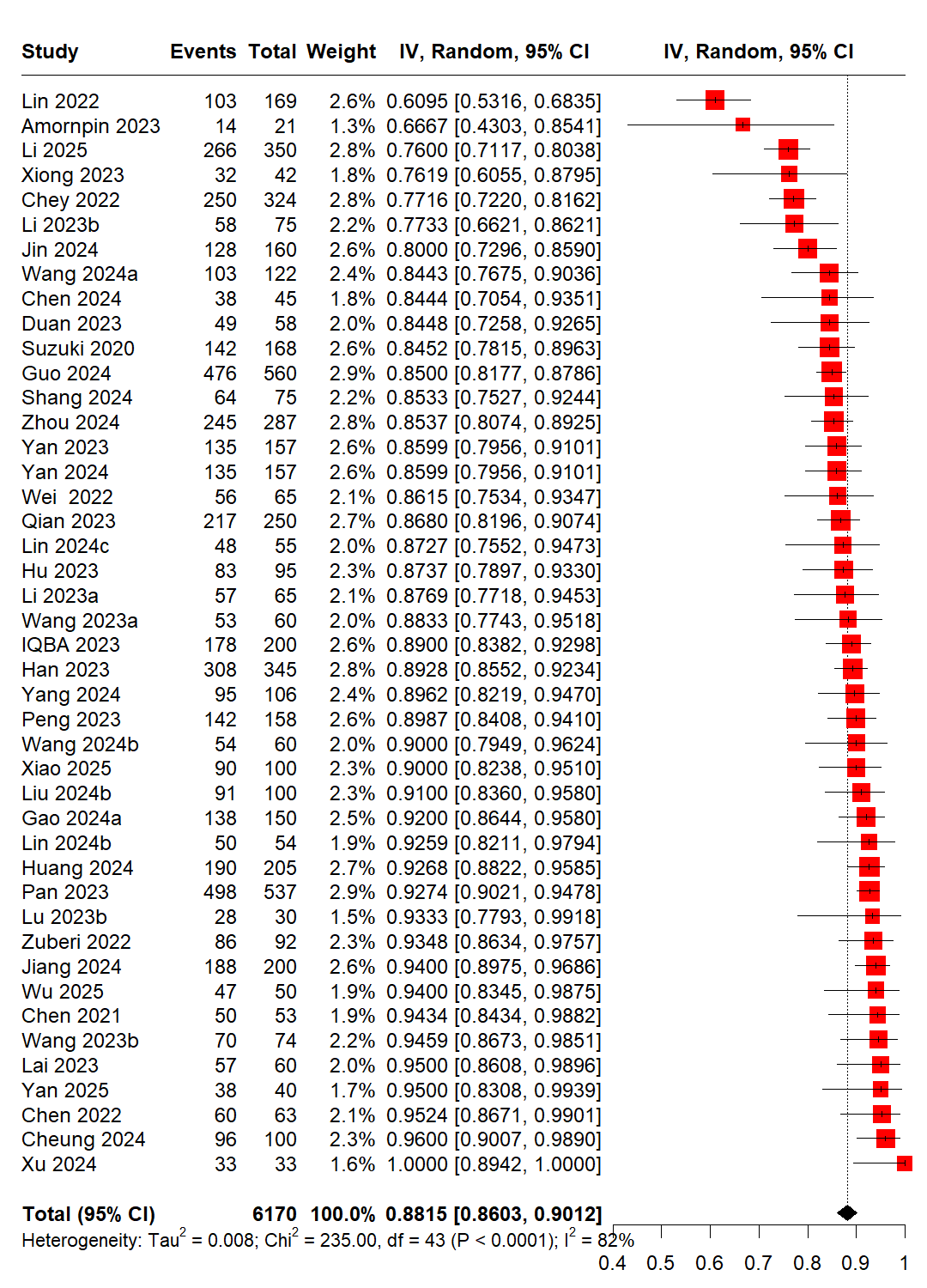


## Figure S 4 Pooled eradication rate for vonoprazan dual regimen


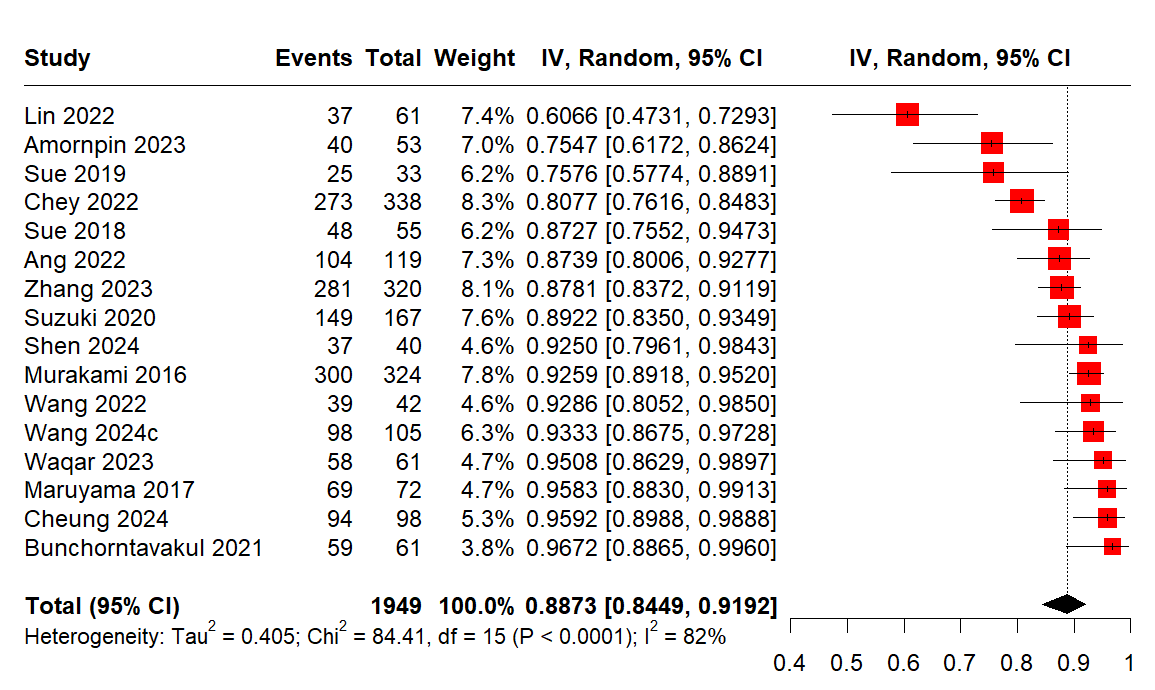


## Figure S 5 Pooled eradication rate for vonoprazan triple regimen


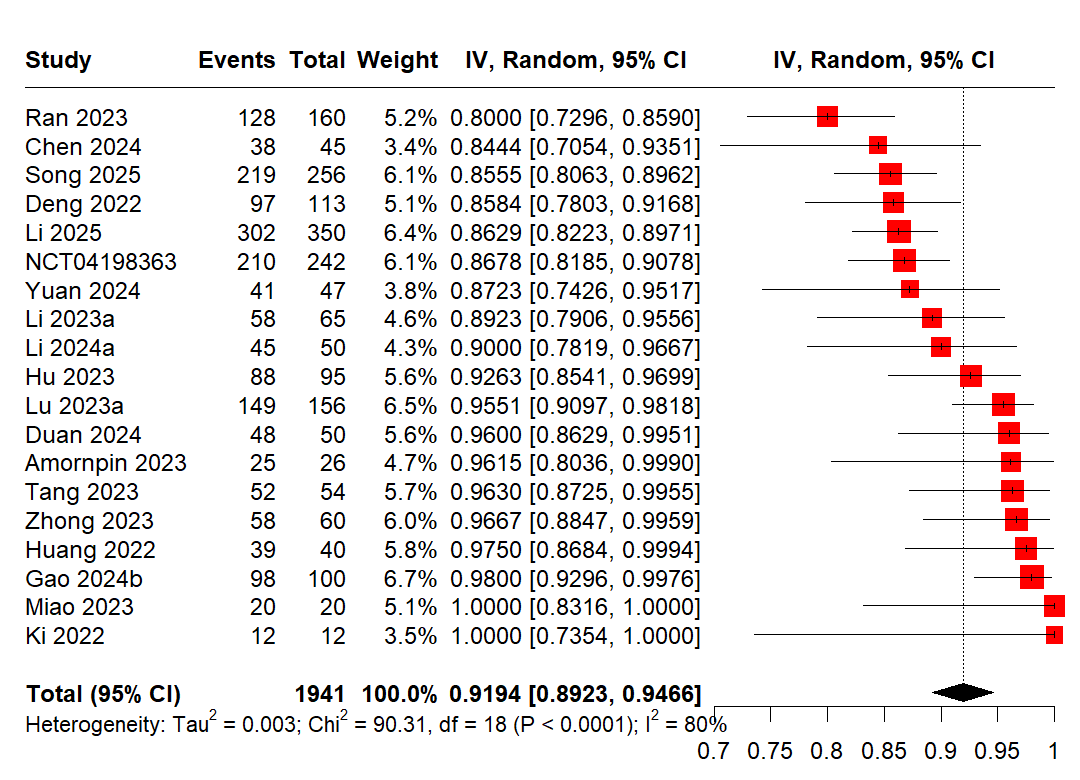


## Figure S 6 Pooled eradication rate for vonoprazan quadruple regimen


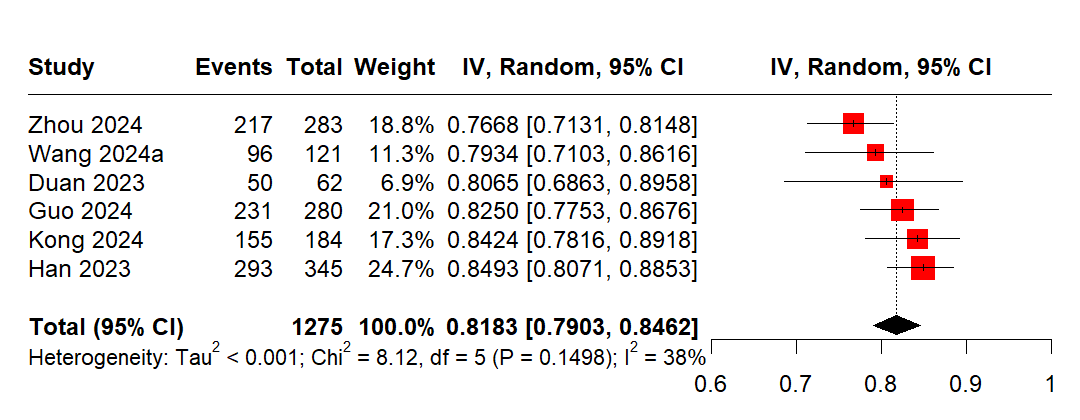


## Figure S 7 Pooled eradication rate for proton pump inhibitor-based dual regimen


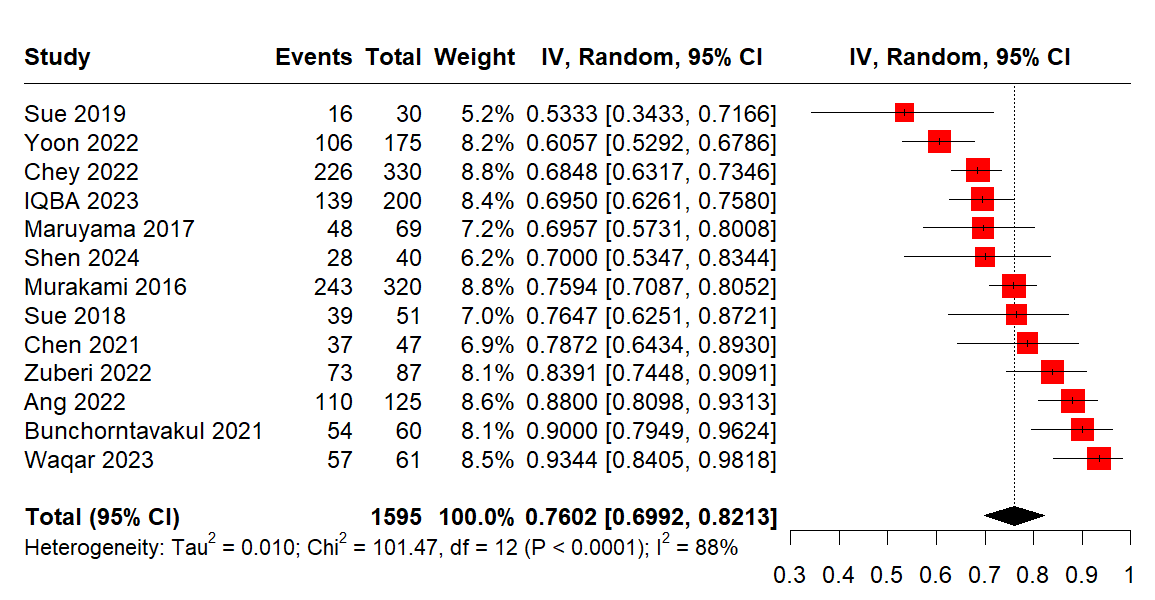


## Figure S 8 Pooled eradication rate for proton pump inhibitor-based triple regimen


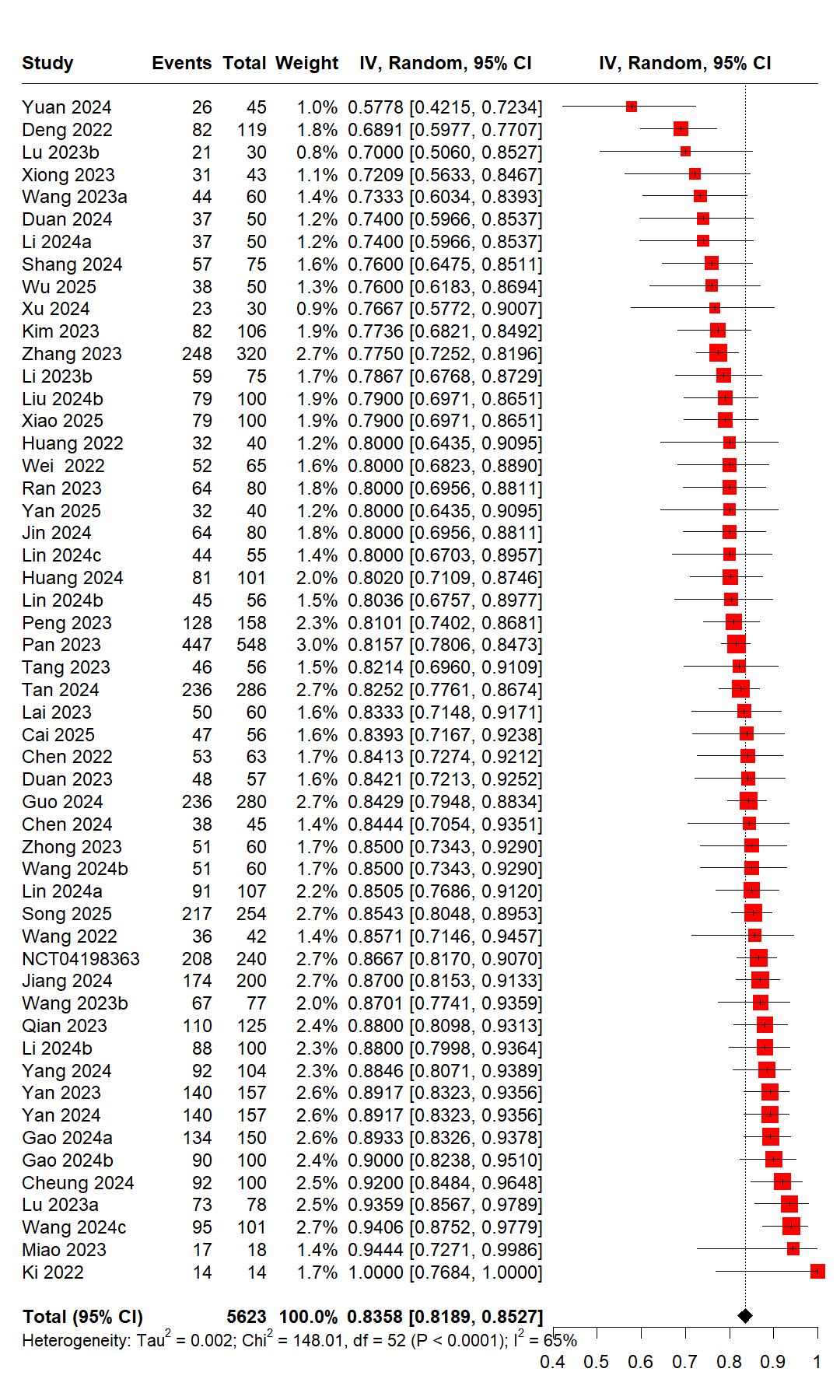


## Figure S 9 Pooled eradication rate for proton pump inhibitor-based quadruple regimen

# Appendix 4 Proportional meta-analysis results-Fix


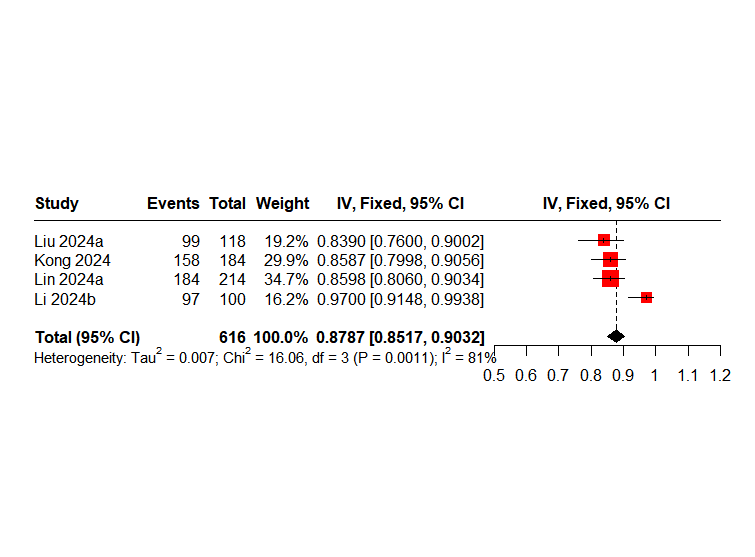


## Figure S 10 Pooled eradication rate for tegoprazan dual regimen


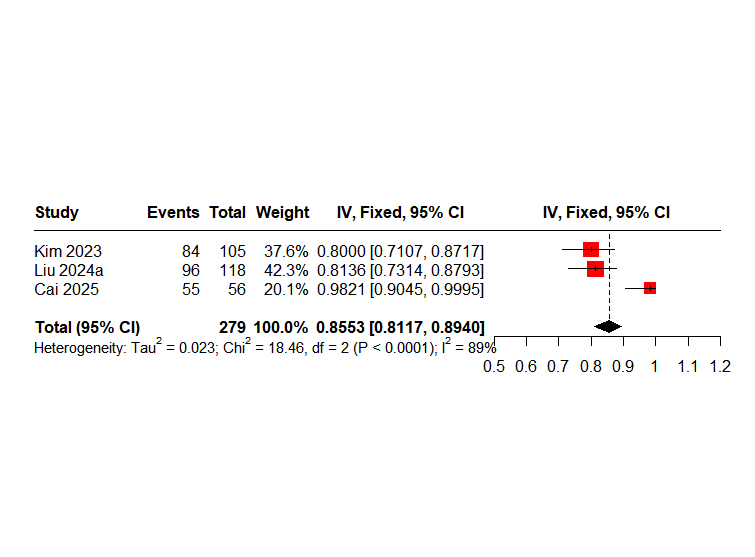


## Figure S 11 Pooled eradication rate for tegoprazan quadruple regimen


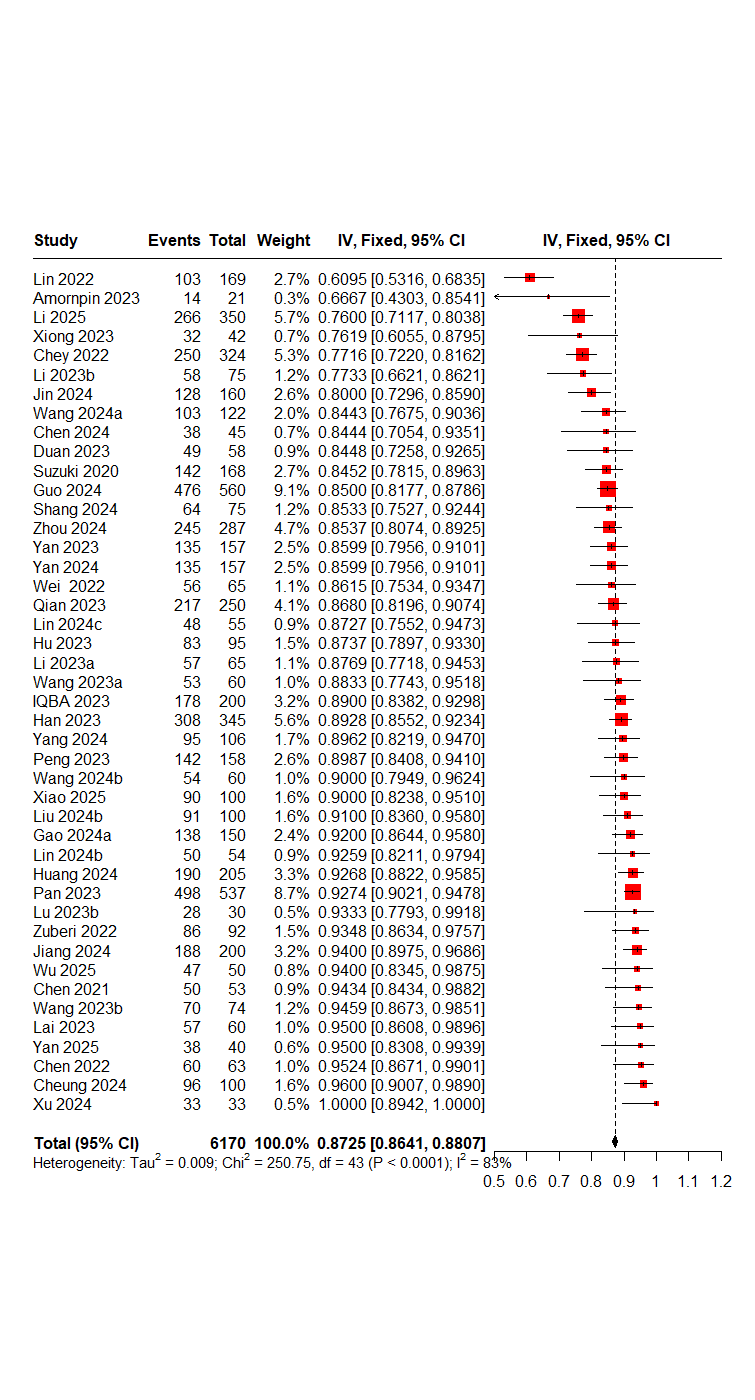


## Figure S 12 Pooled eradication rate for vonoprazan dual regimen


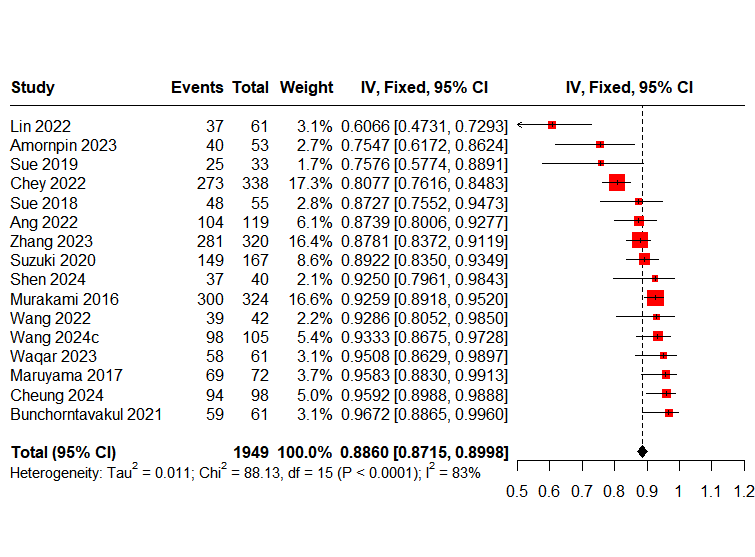


## Figure S 13 Pooled eradication rate for vonoprazan triple regimen


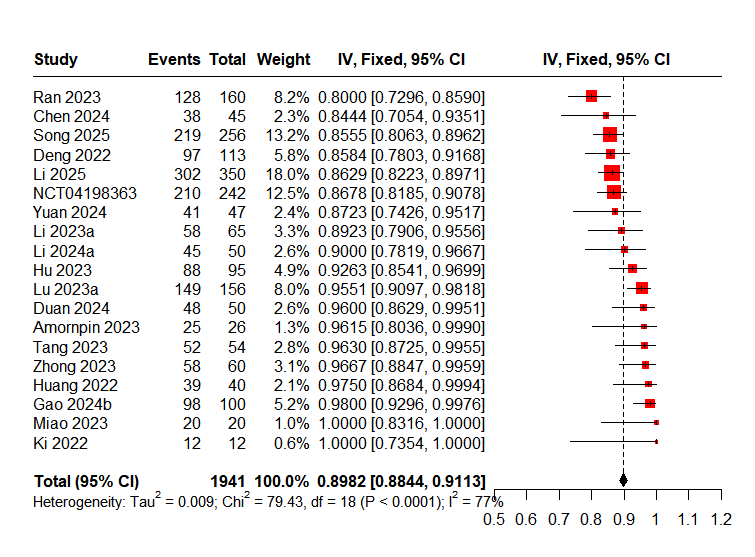


## Figure S 14 Pooled eradication rate for vonoprazan quadruple regimen


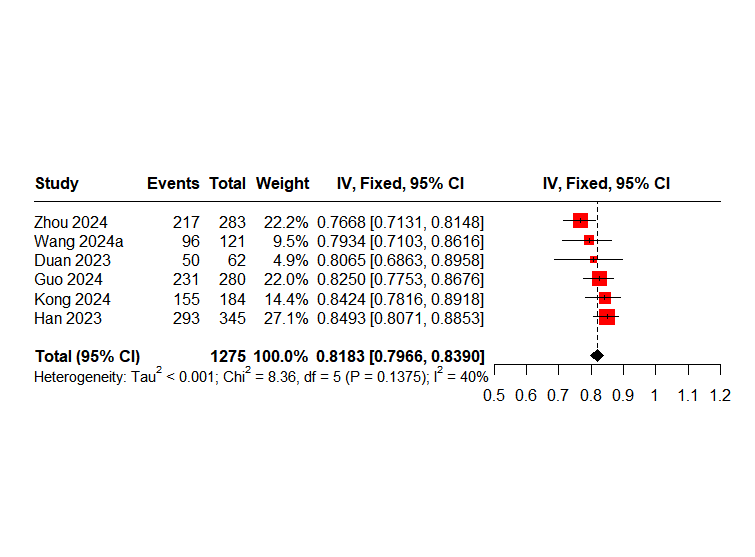


## Figure S 15 Pooled eradication rate for proton pump inhibitor-based dual regimen


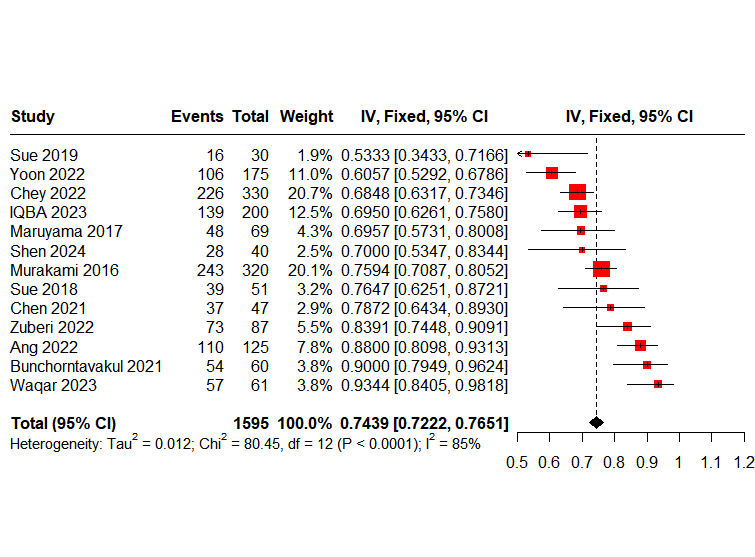


## Figure S 16 Pooled eradication rate for proton pump inhibitor-based triple regimen


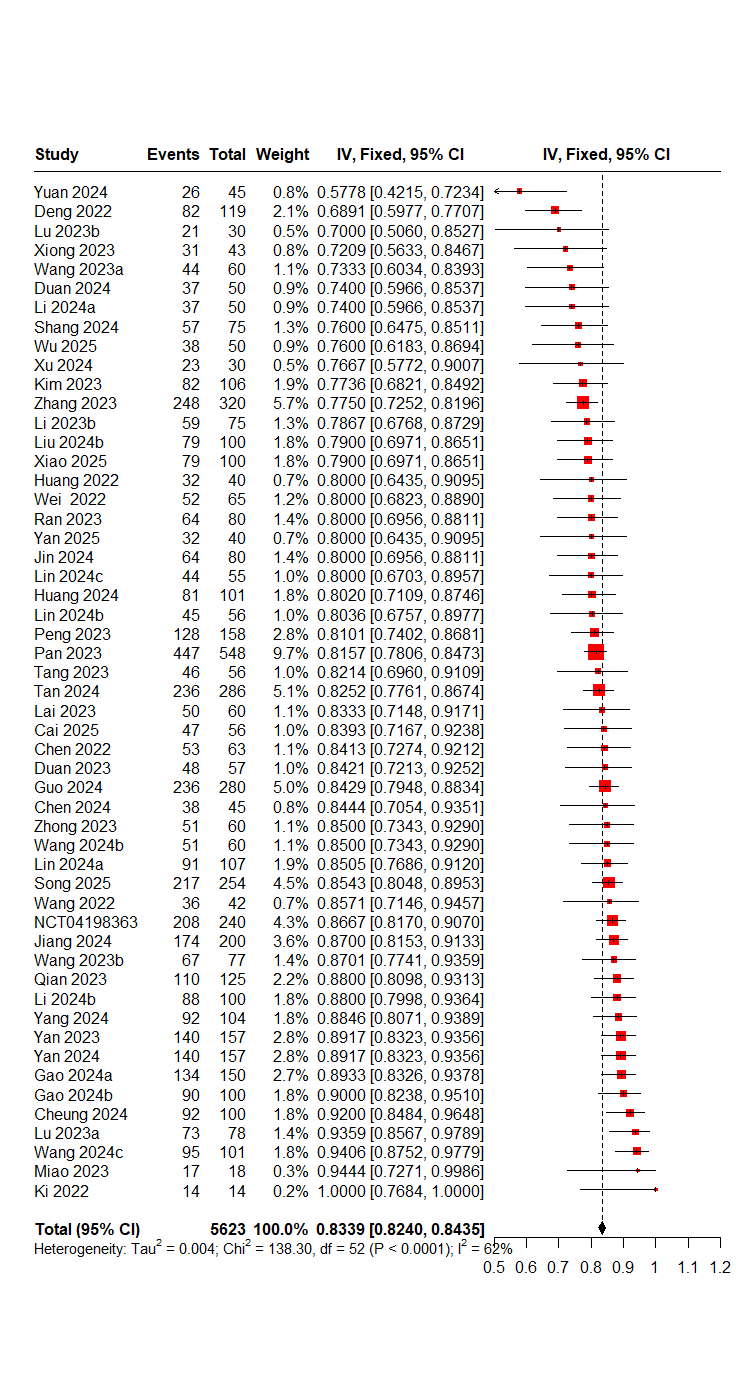


## Figure S 17 Pooled eradication rate for proton pump inhibitor-based quadruple regimen

# Appendix 5 Eradication rates for various PCAB-based regimens-Fixed


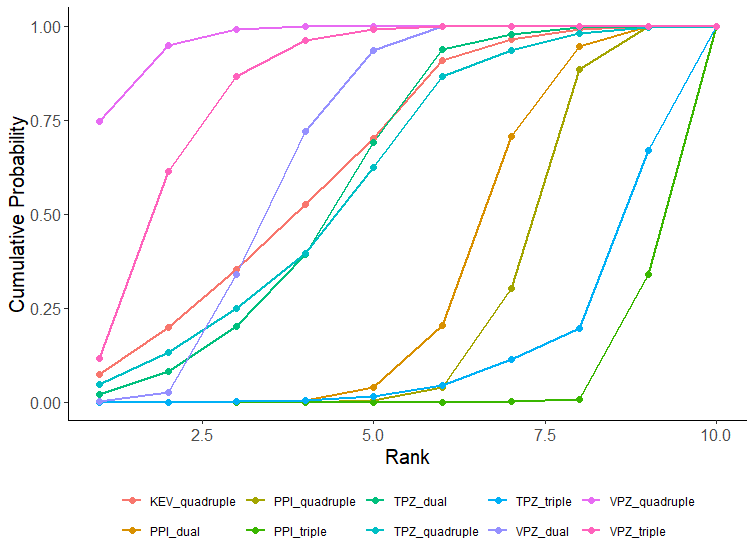


## Figure S 18 Ranking with SUCRA for PCAB-based therapies based on eradication rate

## Table S 3 League matrix of comparative efficacies of PCAB-based eradication therapy

| **TPZ_dual** | 1.10 (0.76, 1.62) | 0.74 (0.51, 1.10) | 0.54 (0.29, 1.00) | 1.29 (0.84, 1.98) | 0.49 (0.32, 0.76) | 1.06 (0.58, 1.93) | 0.99 (0.61, 1.63) | 1.50 (0.99, 2.29) | 0.69 (0.48, 1.01) |
| --- | --- | --- | --- | --- | --- | --- | --- | --- | --- |
| 0.91 (0.62, 1.32) | **VPZ_dual** | **0.67 (0.55, 0.82)** | **0.49 (0.30, 0.79)** | 1.16 (0.95, 1.43) | **0.44 (0.35, 0.55)** | 0.96 (0.59, 1.57) | 0.90 (0.55, 1.46) | **1.36 (1.11, 1.67)** | **0.62 (0.55, 0.71)** |
| 1.35 (0.91, 1.98) | **1.49 (1.21, 1.83)** | **PPI_dual** | 0.73 (0.43, 1.24) | 1.74 (1.30, 2.31) | **0.66 (0.49, 0.89)** | 1.43 (0.85, 2.42) | 1.34 (0.80, 2.22) | **2.02 (1.53, 2.69)** | 0.93 (0.74, 1.17) |
| **1.86 (1.00, 3.45)** | **2.06 (1.26, 3.36)** | 1.38 (0.81, 2.35) | **TPZ_triple** | **2.39 (1.48, 3.88)** | 0.91 (0.59, 1.40) | **1.96 (1.00, 3.92)** | 1.84 (0.93, 3.68) | **2.79 (1.65, 4.73)** | 1.28 (0.78, 2.12) |
| 0.78 (0.51, 1.19) | 0.86 (0.70, 1.05) | **0.58 (0.43, 0.77)** | **0.42 (0.26, 0.68)** | **VPZ_triple** | **0.38 (0.31, 0.47)** | 0.82 (0.49, 1.39) | 0.77 (0.46, 1.30) | 1.17 (0.88, 1.54) | **0.54 (0.43, 0.67)** |
| **2.05 (1.32, 3.17)** | **2.26 (1.81, 2.84)** | **1.52 (1.12, 2.06)** | 1.10 (0.71, 1.71) | **2.64 (2.14, 3.26)** | **PPI_triple** | **2.17 (1.27, 3.70)** | **2.03 (1.19, 3.47)** | **3.07 (2.29, 4.15)** | **1.41 (1.10, 1.81)** |
| 0.94 (0.52, 1.71) | 1.05 (0.64, 1.69) | 0.70 (0.41, 1.18) | 0.51 (0.25, 1.00) | 1.22 (0.72, 2.05) | **0.46 (0.27, 0.78)** | **KEV_quadruple** | 0.94 (0.48, 1.82) | 1.42 (0.85, 2.36) | 0.65 (0.40, 1.04) |
| 1.01 (0.61, 1.65) | 1.11 (0.68, 1.82) | 0.75 (0.45, 1.25) | 0.54 (0.27, 1.07) | 1.30 (0.77, 2.20) | **0.49 (0.29, 0.84)** | 1.07 (0.55, 2.09) | **TPZ_quadruple** | 1.51 (0.90, 2.54) | 0.70 (0.43, 1.12) |
| 0.67 (0.44, 1.01) | **0.74 (0.60, 0.90)** | **0.49 (0.37, 0.66)** | **0.36 (0.21, 0.61)** | 0.86 (0.65, 1.13) | **0.33 (0.24, 0.44)** | 0.71 (0.42, 1.18) | 0.66 (0.39, 1.11) | **VPZ_quadruple** | **0.46 (0.38, 0.56)** |
| 1.45 (0.99, 2.10) | **1.60 (1.42, 1.81)** | 1.07 (0.86, 1.35) | 0.78 (0.47, 1.29) | **1.86 (1.50, 2.33)** | **0.71 (0.55, 0.91)** | 1.53 (0.96, 2.47) | 1.44 (0.89, 2.31) | **2.17 (1.79, 2.64)** | **PPI_quadruple** |
| Odds ratio (95% confidence interval) reported. Statistically significant data presented in bold.  *TPZ_dual* Tegoprazan combined with one antibiotic; *VPZ_dual* Vonoprazan combined with one antibiotic; *PPI_dual* Proton pump inhibitor combined with one antibiotic; *TPZ_triple* Tegoprazan combined with two antibiotics; *VPZ_triple* Vonoprazan combined with two antibiotics; *PPI_triple* Proton pump inhibitor combined with two antibiotics; *KEV_quadruple* Keverprazan combined with two antibiotics and bismuth; *TPZ_quadruple* Tegoprazan combined with two antibiotics and bismuth; *VPZ_quadruple* Vonoprazan combined with two antibiotics and bismuth; *PPI_quadruple* Proton pump inhibitor combined with two antibiotics and bismuthver. | | | | | | | | | |

# Appendix 6 Subgroup analysis of eradication rates for different PCAB-based regimens-Random

a
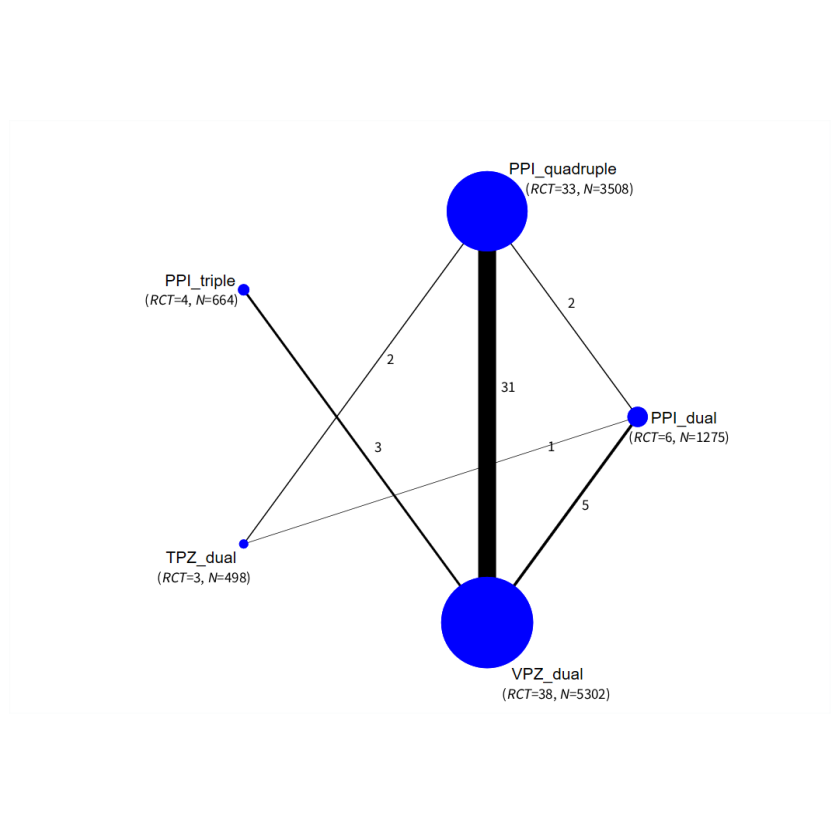


b
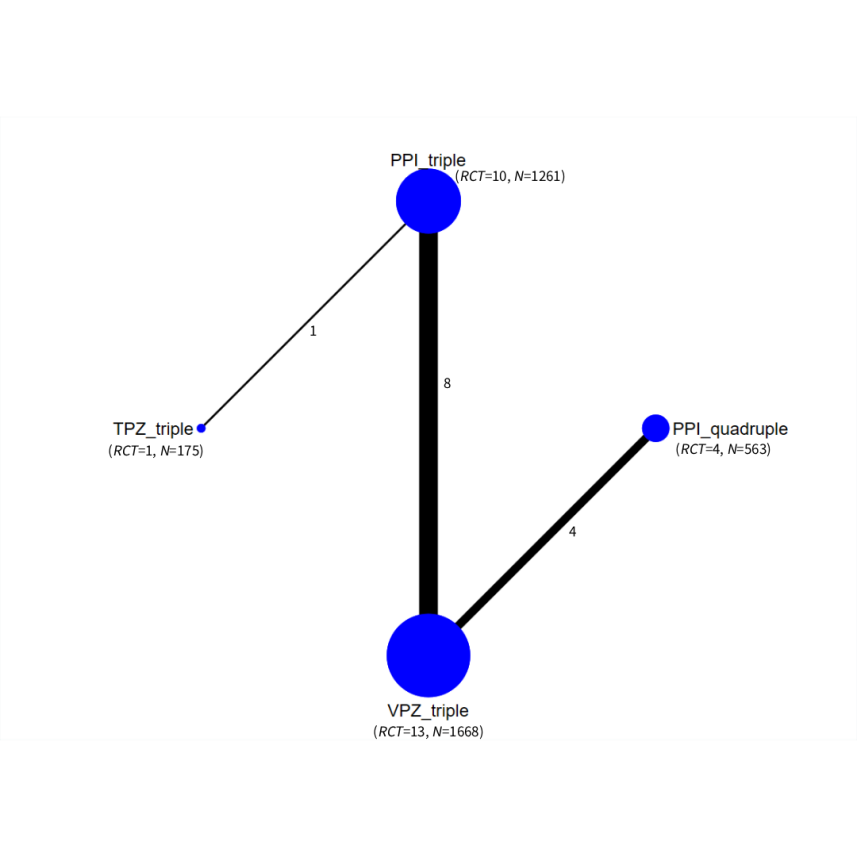


c
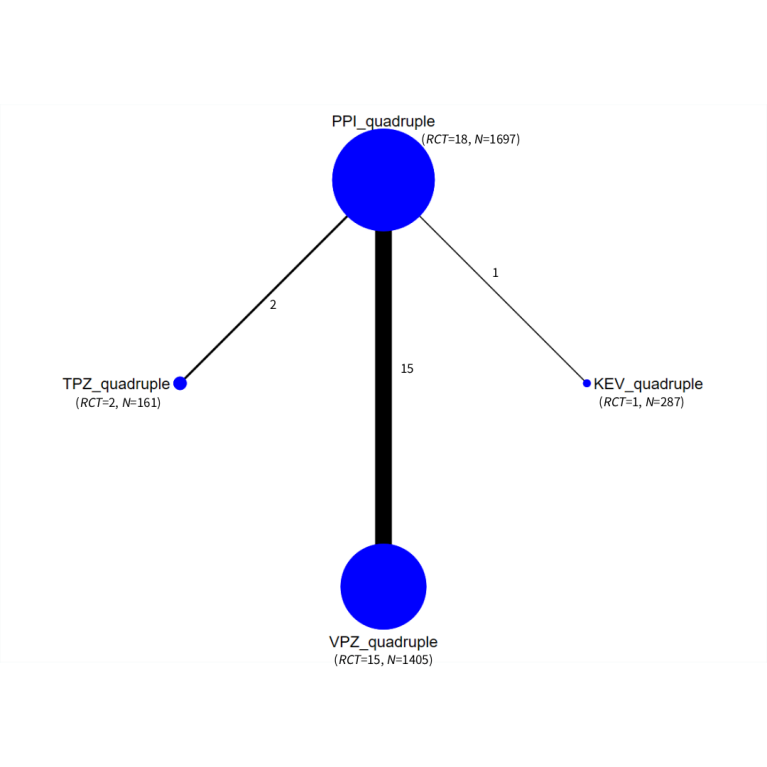


## Figure S 19 Network map of PCAB-based eradication treatment on eradication rates. a) dual therapy, b) triple therapy, and c) quadruple therapy


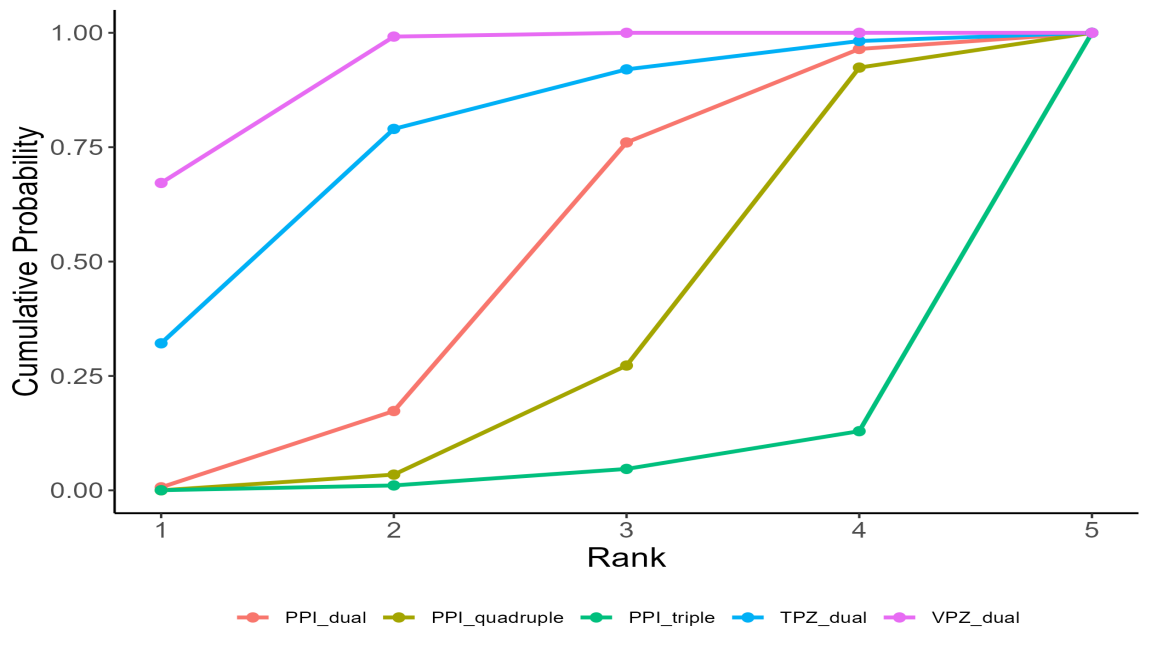


a


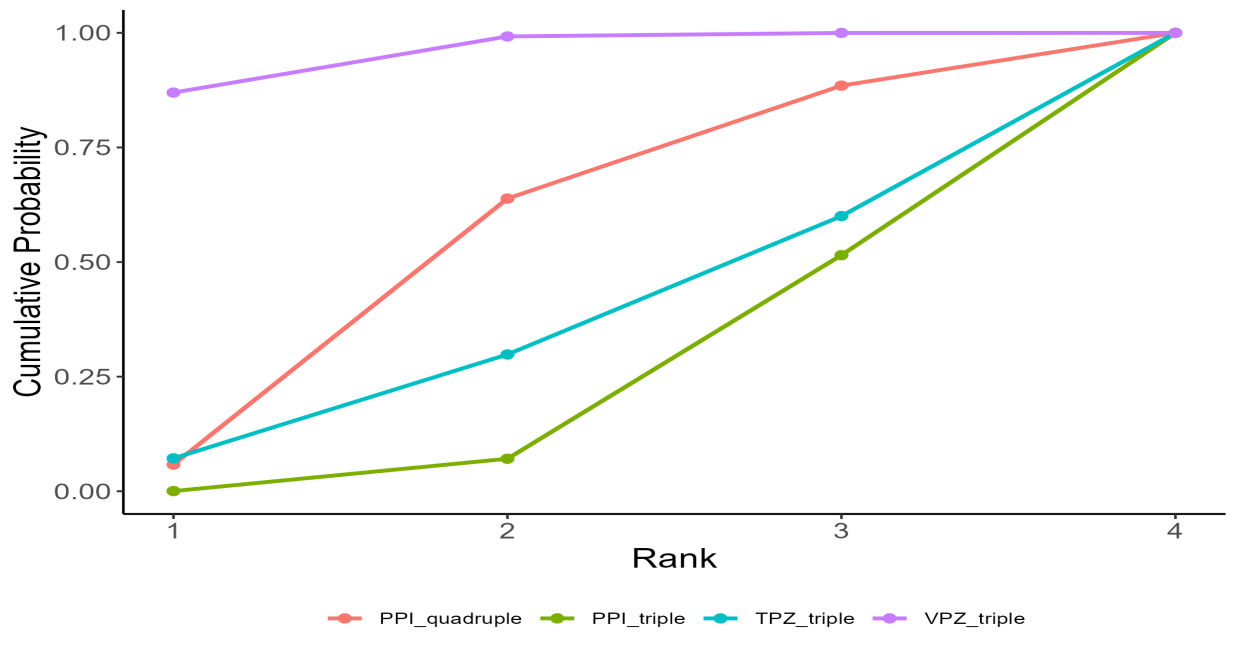


b


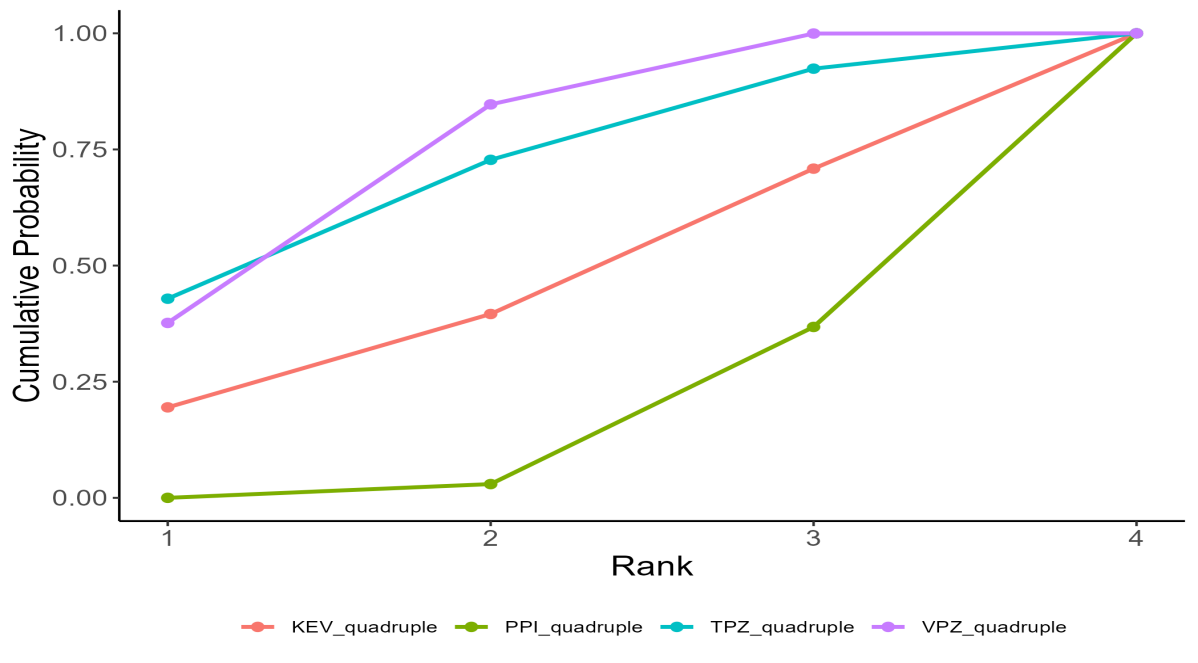


C

## Figure S 20 Ranking with SUCRA for PCAB-based therapies based on eradication rate evaluated in the included RCTs. a) dual therapy, b) triple therapy, and c) quadruple therapy

## Table S 4 League matrix of comparative efficacies of PCAB-based dual eradication therapy

| **TPZ_dual** | 1.16 (0.60, 2.19) | 0.75 (0.39, 1.44) | 0.46 (0.19, 1.03) | 0.65 (0.34, 1.22) |
| --- | --- | --- | --- | --- |
| 0.86 (0.46, 1.66) | VPZ_dual | 0.65 (0.44, 0.95) | 0.4 (0.23, 0.65) | 0.56 (0.45, 0.69) |
| 1.33 (0.69, 2.57) | **1.54 (1.06, 2.27)** | PPI_dual | 0.61 (0.31, 1.13) | 0.87 (0.57, 1.30) |
| 2.17 (0.97, 5.15) | **2.51 (1.53, 4.33)** | 1.63 (0.88, 3.18) | PPI_triple | 1.42 (0.83, 2.52) |
| 1.53 (0.82, 2.95) | **1.77 (1.45, 2.21)** | 1.15 (0.77, 1.74) | 0.7 (0.4, 1.21) | PPI_quadruple |
| Odd ratio (95% confidence interval) reported. Statistically significant data presented in bold.  *VPZ_dual* Vonoprazan combined with one antibiotic, *PPI_quadruple* Proton pump inhibitor combined with two antibiotics and Bismuth, *PPI_triple* Proton pump inhibitor combined with two antibiotics, *PPI_dual* Proton pump inhibitor combined with one antibiotic; *TPZ_dual* Tegoprazan combined with one antibiotic. | | | | |

## Table S 5 League matrix of comparative efficacies of PCAB-based triple eradication therapy

| TPZ_triple | 2.44 (0.64, 9.98) | 0.91 (0.26, 3.23) | 1.36 (0.29, 6.86) |
| --- | --- | --- | --- |
| 0.41 (0.1, 1.56) | VPZ_triple | 0.37 (0.22, 0.6) | 0.56 (0.26, 1.23) |
| 1.10 (0.31, 3.91) | **2.69 (1.66, 4.58)** | PPI_triple | 1.5 (0.62, 3.94) |
| 0.74 (0.15, 3.4) | 1.79 (0.81, 3.84) | 0.67 (0.25, 1.61) | PPI_quadruple |
| Odd ratio (95% confidence interval) reported. Statistically significant data presented in bold.  *VPZ_triple* Vonoprazan combined with two antibiotics, *PPI_quadruple* Proton pump inhibitor combined with two antibiotics and Bismuth, *PPI_triple* Proton pump inhibitor combined with two antibiotics, *TPZ_triple* Tegoprazan combined with two antibiotics. | | | |

## Table S 6 League matrix of comparative efficacies of PCAB-based quadruple eradication therapy

| KEV_quadruple | 1.66 (0.16, 21.58) | 1.66 (0.25, 12.78) | 0.65 (0.10, 4.21) |
| --- | --- | --- | --- |
| 0.60 (0.05, 6.12) | TPZ_quadruple | 1.01 (0.19, 4.93) | 0.39 (0.07, 1.64) |
| 0.60 (0.08, 3.94) | 0.99 (0.20, 5.36) | VPZ_quadruple | 0.39 (0.20, 0.67) |
| 1.53 (0.24, 9.77) | 2.53 (0.61, 13.48) | **2.55 (1.50, 4.94)** | PPI_quadruple |
| Odd ratio (95% confidence interval) reported. Statistically significant data presented in bold.  *VPZ_quadruple* Vonoprazan combined with two antibiotics and Bismuth, *PPI_quadruple* Proton pump inhibitor combined with two antibiotics and Bismuth, *TPZ_quadruple* Tegoprazan combined with two antibiotics and Bismuth; *KEV_quadruple* Keboprazan combined with two antibiotics and Bismuth. | | | |

# Appendix 7 Subgroup analysis of eradication rates for different PCAB-based regimens-Fix


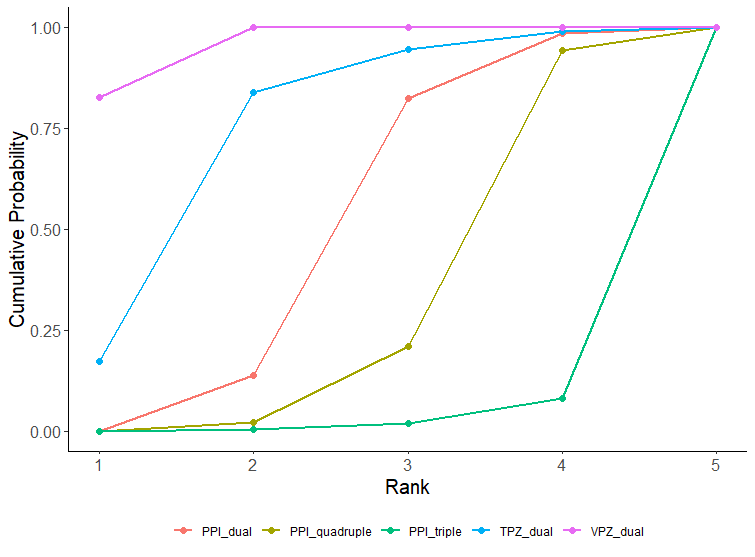


a


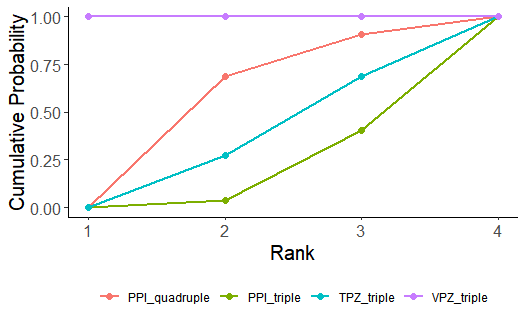


b


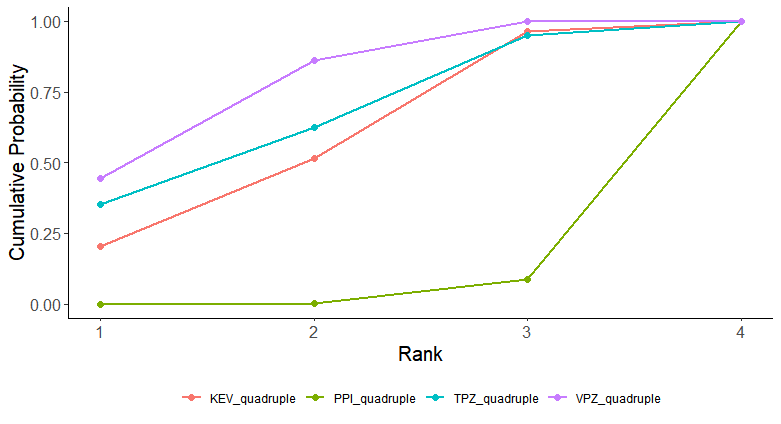


c

## Figure S 21 Ranking with SUCRA for PCAB-based therapies based on eradication rate evaluated in the included RCTs. a) dual therapy, b) triple therapy, and c) quadruple therapy

## Table S 7 League matrix of comparative efficacies of PCAB-based dual eradication therapy

| **TPZ_dual** | 1.22 (0.81, 1.86) | 0.80 (0.53, 1.22) | **0.57 (0.35, 0.94)** | 0.72 (0.48, 1.09) |
| --- | --- | --- | --- | --- |
| 0.82 (0.54, 1.24) | **VPZ_dual** | **0.66 (0.53, 0.81)** | **0.46 (0.35, 0.61)** | **0.59 (0.51, 0.67)** |
| 1.25 (0.82, 1.88) | **1.53 (1.24, 1.88)** | **PPI_dual** | 0.71 (0.50, 1.00) | 0.90 (0.71, 1.13) |
| **1.76 (1.07, 2.88)** | **2.15 (1.64, 2.83)** | **1.41 (1.00, 1.99)** | **PPI_triple** | 1.26 (0.93, 1.72) |
| 1.39 (0.92, 2.09) | **1.70 (1.48, 1.95)** | 1.12 (0.89, 1.40) | 0.79 (0.58, 1.07) | **PPI_quadruple** |
| Odd ratio (95% confidence interval) reported. Statistically significant data presented in bold.  *VPZ_dual* Vonoprazan combined with one antibiotic, *PPI_quadruple* Proton pump inhibitor combined with two antibiotics and Bismuth, *PPI_triple* Proton pump inhibitor combined with two antibiotics, *PPI_dual* Proton pump inhibitor combined with one antibiotic; *TPZ_dual* Tegoprazan combined with one antibiotic. | | | | |

## Table S 8 League matrix of comparative efficacies of PCAB-based triple eradication therapy

| **TPZ_triple** | **2.28 (1.39, 3.73)** | 0.91 (0.59, 1.40) | 1.19 (0.64, 2.18) |
| --- | --- | --- | --- |
| **0.44 (0.27, 0.72)** | **VPZ_triple** | **0.40 (0.31, 0.50)** | **0.52 (0.36, 0.74)** |
| 1.10 (0.71, 1.70) | **2.52 (1.99, 3.19)** | **PPI_triple** | 1.31 (0.85, 2.02) |
| 0.84 (0.46, 1.55) | **1.92 (1.34, 2.78)** | 0.76 (0.50, 1.18) | **PPI_quadruple** |
| Odd ratio (95% confidence interval) reported. Statistically significant data presented in bold.  *VPZ_triple* Vonoprazan combined with two antibiotics, *PPI_quadruple* Proton pump inhibitor combined with two antibiotics and Bismuth, *PPI_triple* Proton pump inhibitor combined with two antibiotics, *TPZ_triple* Tegoprazan combined with two antibiotics. | | | |

## Table S 9 League matrix of comparative efficacies of PCAB-based quadruple eradication therapy

| **KEV_quadruple** | 1.07 (0.50, 2.30) | 1.16 (0.68, 1.94) | 0.65 (0.41, 1.04) |
| --- | --- | --- | --- |
| 0.93 (0.44, 1.98) | **TPZ_quadruple** | 1.08 (0.56, 2.05) | 0.61 (0.33, 1.11) |
| 0.86 (0.52, 1.46) | 0.93 (0.49, 1.79) | **VPZ_quadruple** | **0.56 (0.45, 0.71)** |
| 1.53 (0.96, 2.45) | 1.65 (0.9, 3.03) | **1.77 (1.41, 2.23)** | **PPI_quadruple** |
| Odd ratio (95% confidence interval) reported. Statistically significant data presented in bold.  *VPZ_quadruple* Vonoprazan combined with two antibiotics and Bismuth, *PPI_quadruple* Proton pump inhibitor combined with two antibiotics and Bismuth, *TPZ_quadruple* Tegoprazan combined with two antibiotics and Bismuth; *KEV_quadruple* Keboprazan combined with two antibiotics and Bismuth. | | | |

# Appendix 8 Subgroup analysis of eradication rates in patients with different treatment histories-Random

a
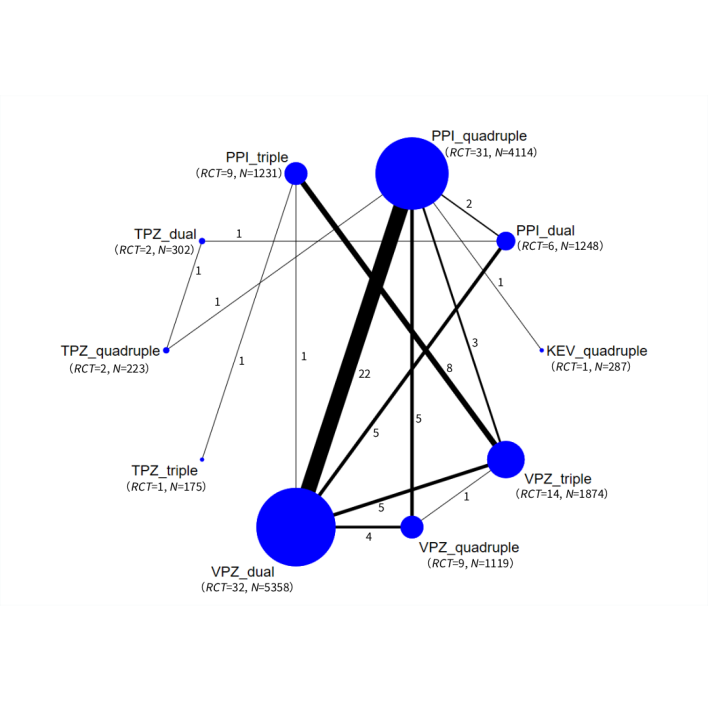


b
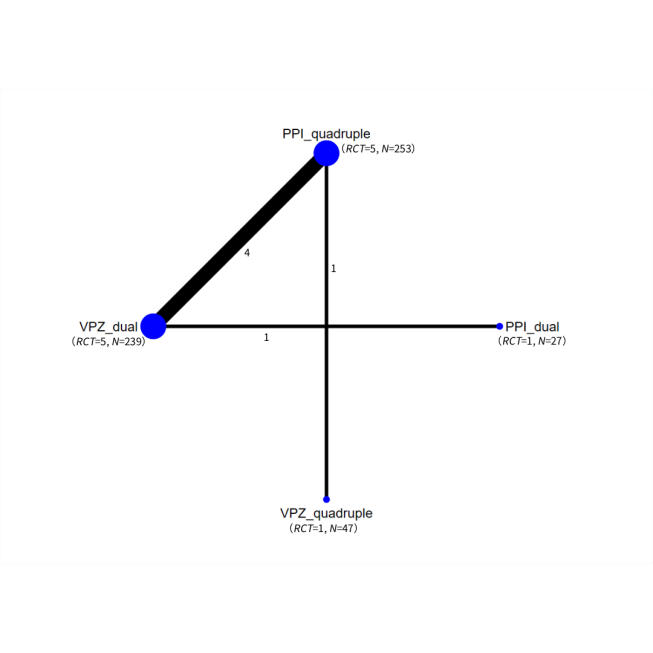


## Figure S 22 Network maps of comparisons regarding different treatment history. a) treatment-naïve patients, b) previously treated patients


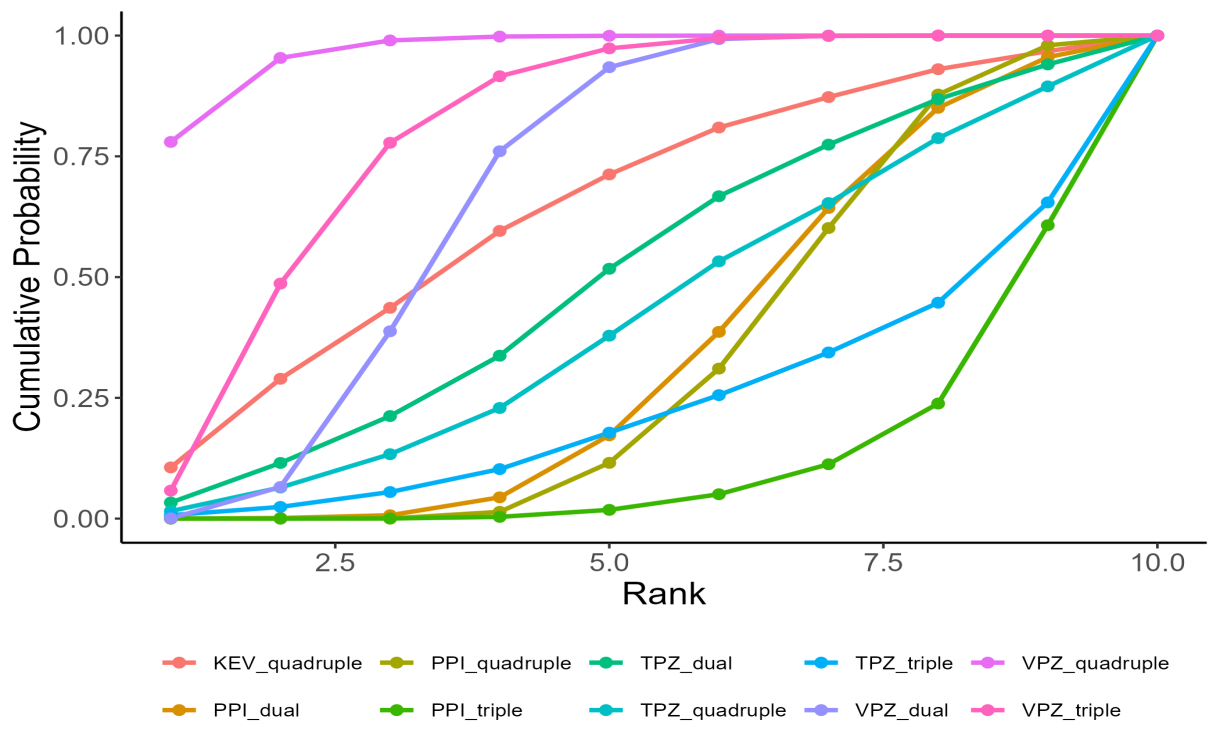


a


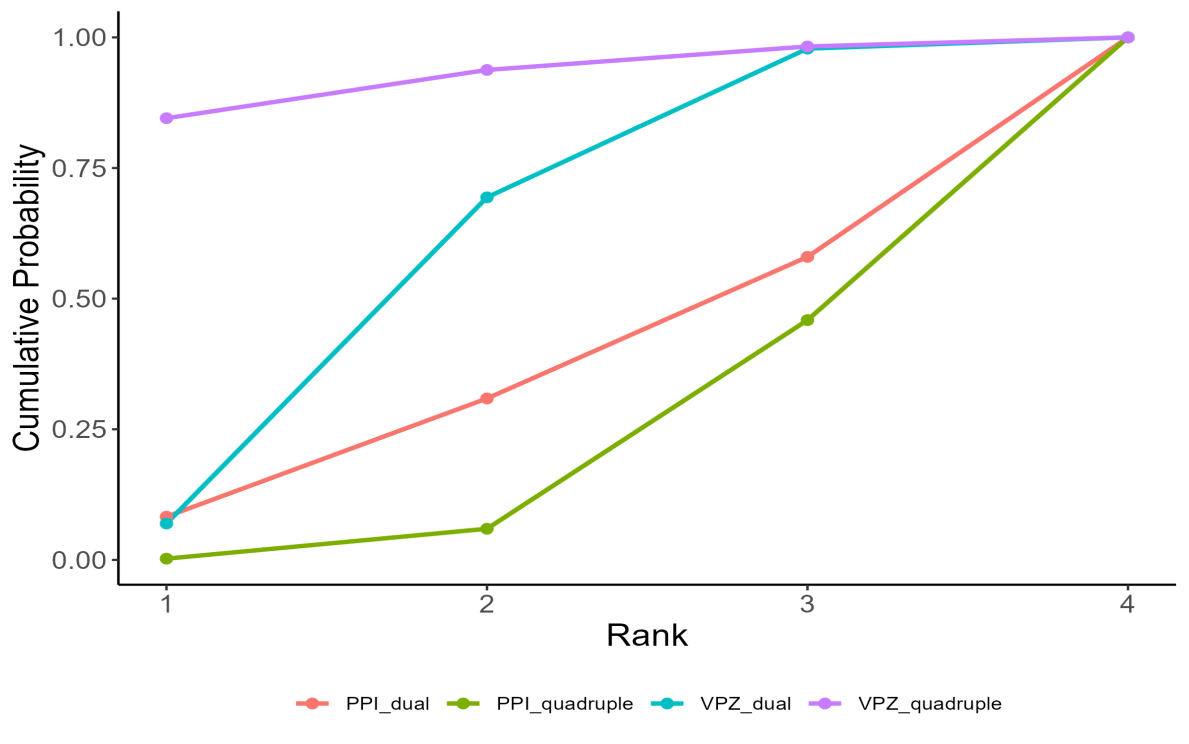


b

## Figure S 23 SUCRA ranking of eradication treatments in patients with different treatment history. a) treatment-naïve patients, b) previously treated patients

## Table S 10 League matrix of comparative efficacies of eradication treatment in treatment-naïve patient

| TPZ_dual | 1.27 (0.56, 2.89) | 0.83 (0.38, 1.82) | 0.65 (0.18, 2.27) | 1.51 (0.61, 3.64) | 0.59 (0.23, 1.52) | 1.24 (0.38, 4.08) | 0.89 (0.4, 2) | 2.17 (0.89, 5.37) | 0.81 (0.36, 1.85) |
| --- | --- | --- | --- | --- | --- | --- | --- | --- | --- |
| 0.79 (0.35, 1.79) | VPZ_dual | 0.65 (0.45, 0.96) | 0.51 (0.19, 1.33) | 1.19 (0.81, 1.7) | 0.47 (0.28, 0.75) | 0.98 (0.41, 2.35) | 0.7 (0.31, 1.59) | 1.71 (1.16, 2.55) | 0.64 (0.52, 0.79) |
| 1.2 (0.55, 2.64) | **1.53 (1.04, 2.25)** | PPI_dual | 0.79 (0.27, 2.18) | 1.81 (1.06, 3.06) | 0.71 (0.38, 1.31) | 1.49 (0.58, 3.81) | 1.07 (0.47, 2.48) | 2.61 (1.54, 4.5) | 0.98 (0.65, 1.47) |
| 1.53 (0.44, 5.5) | 1.94 (0.75, 5.29) | 1.27 (0.46, 3.7) | TPZ_triple | 2.31 (0.93, 5.86) | 0.91 (0.39, 2.1) | 1.91 (0.54, 7.02) | 1.37 (0.4, 4.93) | 3.31 (1.22, 9.75) | 1.24 (0.48, 3.41) |
| 0.66 (0.27, 1.63) | 0.84 (0.59, 1.24) | **0.55 (0.33, 0.94)** | 0.43 (0.17, 1.08) | VPZ_triple | 0.39 (0.26, 0.57) | 0.82 (0.32, 2.14) | 0.59 (0.25, 1.45) | 1.44 (0.87, 2.47) | 0.54 (0.37, 0.81) |
| 1.69 (0.66, 4.44) | **2.15 (1.33, 3.59)** | 1.40 (0.76, 2.65) | 1.10 (0.48, 2.55) | **2.55 (1.74, 3.78)** | PPI_triple | 2.1 (0.78, 5.76) | 1.51 (0.6, 3.94) | 3.67 (2.01, 7.01) | 1.37 (0.83, 2.33) |
| 0.81 (0.25, 2.62) | 1.02 (0.42, 2.47) | 0.67 (0.26, 1.73) | 0.52 (0.14, 1.86) | 1.21 (0.47, 3.09) | 0.48 (0.17, 1.27) | KEV_quadruple | 0.72 (0.22, 2.34) | 1.74 (0.69, 4.51) | 0.66 (0.28, 1.54) |
| 1.12 (0.50, 2.50) | 1.42 (0.63, 3.20) | 0.93 (0.40, 2.12) | 0.73 (0.20, 2.52) | 1.69 (0.69, 4.05) | 0.66 (0.25, 1.68) | 1.39 (0.43, 4.51) | TPZ_quadruple | 2.43 (1.01, 5.94) | 0.91 (0.41, 2.01) |
| 0.46 (0.19, 1.13) | **0.59 (0.39, 0.86)** | **0.38 (0.22, 0.65)** | **0.30 (0.10, 0.82)** | 0.7 (0.40, 1.15) | **0.27 (0.14, 0.50)** | 0.57 (0.22, 1.45) | **0.41 (0.17, 0.99)** | VPZ_quadruple | 0.38 (0.25, 0.55) |
| 1.23 (0.54, 2.80) | **1.56 (1.27, 1.93)** | 1.02 (0.68, 1.54) | 0.80 (0.29, 2.1) | **1.86 (1.24, 2.73)** | 0.73 (0.43, 1.20) | 1.53 (0.65, 3.61) | 1.10 (0.50, 2.45) | **2.66 (1.81, 3.99)** | PPI_quadruple |
| Odd ratio (95% confidence interval) reported. Statistically significant data presented in bold.  *VPZ_quadruple* Vonoprazan combined with two antibiotics and Bismuth, *VPZ_triple* Vonoprazan combined with two antibiotics, *VPZ_dual* Vonoprazan combined with one antibiotic, *PPI_quadruple* Proton pump inhibitor combined with two antibiotics and Bismuth, *PPI_triple* Proton pump inhibitor combined with two antibiotics, *PPI_dual* Proton pump inhibitor combined with one antibiotic; *TPZ_dual* Tegoprazan combined with one antibiotic; *TPZ_triple* Tegoprazan combined with two antibiotics; *TPZ_quadruple* Tegoprazan combined with two antibiotics and Bismuth; *KEV_quadruple* Keboprazan combined with two antibiotics and Bismuth | | | | | | | | | |

## Table S 11 League matrix of comparative efficacies of eradication treatment in previously treated patients

| VPZ_dual | 0.66 (0.11, 3.86) | 2.96 (0.43, 19.19) | 0.56 (0.22, 1.26) |
| --- | --- | --- | --- |
| 1.52 (0.26, 9.04) | PPI_dual | 4.5 (0.32, 59.83) | 0.85 (0.11, 5.83) |
| 0.34 (0.05, 2.31) | 0.22 (0.02, 3.10) | VPZ_quadruple | 0.19 (0.03, 1.02) |
| 1.78 (0.79, 4.51) | 1.17 (0.17, 8.83) | 5.27 (0.98, 29.31) | PPI_quadruple |
| Odd ratio (95% confidence interval) reported. Statistically significant data presented in bold.  *VPZ_quadruple* Vonoprazan combined with two antibiotics and Bismuth, *VPZ_dual* Vonoprazan combined with one antibiotic, *PPI_quadruple* Proton pump inhibitor combined with two antibiotics and Bismuth, *PPI_dual* Proton pump inhibitor combined with one antibiotic. | | | |

# Appendix 9 Subgroup analysis of eradication rates in patients with different treatment histories-Fix


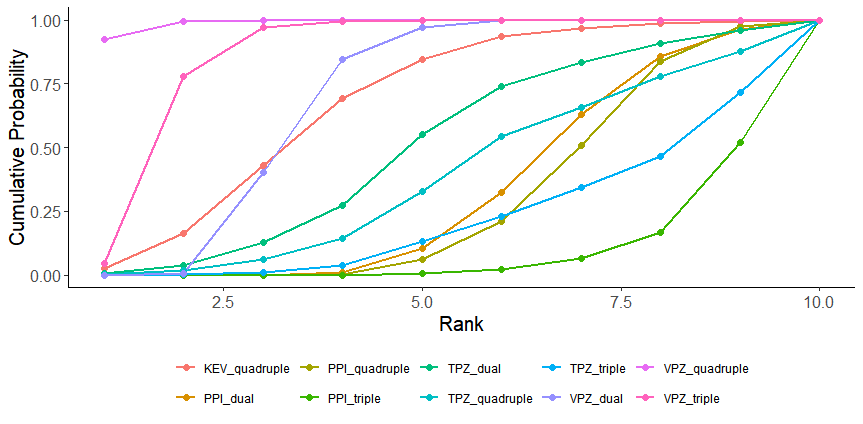
a

b
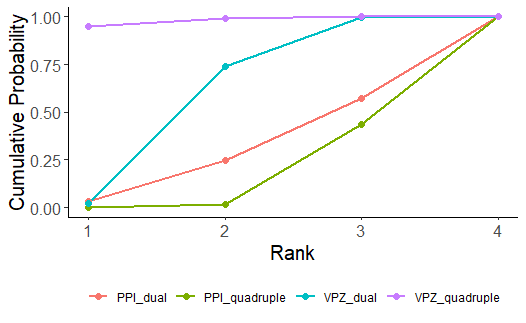


## Figure S 24 SUCRA ranking of eradication treatments in patients with different treatment history. a) treatment-naïve patients, b) previously treated patients

## Table S 12 League matrix of comparative efficacies of eradication treatment in treatment-naïve patients

| **TPZ_dual** | 1.27 (0.75, 2.16) | 0.85 (0.51, 1.39) | 0.73 (0.36, 1.56) | 1.61 (0.91, 2.88) | 0.67 (0.37, 1.19) | 1.26 (0.63, 2.49) | 0.89 (0.52, 1.58) | **2.13 (1.19, 3.83)** | 0.82 (0.49, 1.40) |
| --- | --- | --- | --- | --- | --- | --- | --- | --- | --- |
| 0.79 (0.46, 1.33) | **VPZ_dual** | **0.66 (0.53, 0.82)** | **0.58 (0.34, 0.99)** | **1.26 (1.02, 1.58)** | **0.52 (0.40, 0.68)** | 0.98 (0.61, 1.59) | 0.70 (0.41, 1.22) | **1.68 (1.30, 2.15)** | **0.65 (0.56, 0.73)** |
| 1.18 (0.72, 1.97) | **1.51 (1.22, 1.88)** | **PPI_dual** | 0.87 (0.50, 1.52) | **1.90 (1.43, 2.61)** | 0.79 (0.56, 1.13) | 1.48 (0.88, 2.47) | 1.06 (0.62, 1.84) | **2.54 (1.85, 3.52)** | 0.97 (0.77, 1.23) |
| 1.37 (0.64, 2.80) | **1.72 (1.01, 2.90)** | 1.15 (0.66, 2.00) | **TPZ_triple** | **2.17 (1.33, 3.59)** | 0.91 (0.59, 1.41) | 1.70 (0.83, 3.41) | 1.21 (0.57, 2.61) | **2.89 (1.64, 5.06)** | 1.12 (0.66, 1.89) |
| 0.62 (0.35, 1.10) | **0.79 (0.63, 0.98)** | **0.53 (0.38, 0.70)** | **0.46 (0.28, 0.75)** | **VPZ_triple** | **0.41 (0.33, 0.52)** | 0.77 (0.46, 1.32) | **0.56 (0.31, 0.98)** | 1.33 (0.95, 1.83) | **0.51 (0.41, 0.65)** |
| 1.5 (0.84, 2.69) | **1.91 (1.47, 2.53)** | 1.27 (0.89, 1.78) | 1.1 (0.71, 1.69) | **2.42 (1.93, 3.06)** | **PPI_triple** | **1.88 (1.09, 3.21)** | 1.35 (0.73, 2.44) | **3.20 (2.24, 4.61)** | 1.23 (0.92, 1.65) |
| 0.79 (0.40, 1.59) | 1.02 (0.63, 1.64) | 0.67 (0.41, 1.14) | 0.59 (0.29, 1.20) | 1.29 (0.76, 2.18) | **0.53 (0.31, 0.92)** | **KEV_quadruple** | 0.71 (0.36, 1.46) | **1.71 (1.01, 2.98)** | 0.66 (0.42, 1.06) |
| 1.12 (0.63, 1.92) | 1.42 (0.82, 2.46) | 0.94 (0.54, 1.62) | 0.83 (0.38, 1.75) | **1.79 (1.02, 3.21)** | 0.74 (0.41, 1.38) | 1.40 (0.69, 2.77) | **TPZ_quadruple** | **2.39 (1.35, 4.37)** | 0.92 (0.53, 1.56) |
| **0.47 (0.26, 0.84)** | **0.59 (0.46, 0.77)** | **0.39 (0.28, 0.54)** | 0.35 (0.20, 0.61) | 0.75 (0.55, 1.05) | **0.31 (0.22, 0.45)** | **0.58 (0.34, 0.99)** | **0.42 (0.23, 0.74)** | **VPZ_quadruple** | **0.38 (0.30, 0.49)** |
| 1.22 (0.72, 2.06) | **1.55 (1.36, 1.77)** | 1.03 (0.81, 1.30) | 0.90 (0.53, 1.52) | **1.96 (1.53, 2.47)** | 0.81 (0.61, 1.08) | 1.52 (0.94, 2.41) | 1.09 (0.64, 1.89) | **2.60 (2.03, 3.39)** | **PPI_quadruple** |
| Odd ratio (95% confidence interval) reported. Statistically significant data presented in bold.  *VPZ_quadruple* Vonoprazan combined with two antibiotics and Bismuth, *VPZ_triple* Vonoprazan combined with two antibiotics, *VPZ_dual* Vonoprazan combined with one antibiotic, *PPI_quadruple* Proton pump inhibitor combined with two antibiotics and Bismuth, *PPI_triple* Proton pump inhibitor combined with two antibiotics, *PPI_dual* Proton pump inhibitor combined with one antibiotic; *TPZ_dual* Tegoprazan combined with one antibiotic; *TPZ_triple* Tegoprazan combined with two antibiotics; *TPZ_quadruple* Tegoprazan combined with two antibiotics and Bismuth; *KEV_quadruple* Keboprazan combined with two antibiotics and Bismuth. | | | | | | | | | |

## Table S 13 League matrix of comparative efficacies of eradication treatment in previously treated patients

| **VPZ_dual** | 0.66 (0.19, 2.19) | 3.09 (0.97, 10.72) | **0.58 (0.34, 0.99)** |
| --- | --- | --- | --- |
| 1.52 (0.46, 5.16) | **PPI_dual** | 4.73 (0.89, 26.39) | 0.89 (0.24, 3.35) |
| 0.32 (0.09, 1.03) | 0.21 (0.04, 1.12) | **VPZ_quadruple** | **0.19 (0.06, 0.52)** |
| **1.71 (1.01, 2.96)** | 1.12 (0.30, 4.21) | **5.28 (1.93, 16.41)** | **PPI_quadruple** |
| Odd ratio (95% confidence interval) reported. Statistically significant data presented in bold.  *VPZ_quadruple* Vonoprazan combined with two antibiotics and Bismuth, *VPZ_dual* Vonoprazan combined with one antibiotic, *PPI_quadruple* Proton pump inhibitor combined with two antibiotics and Bismuth, *PPI_dual* Proton pump inhibitor combined with one antibiotic. | | | |

# Appendix 10 Subgroup analysis of eradication rates for various treatment durations-Random

a
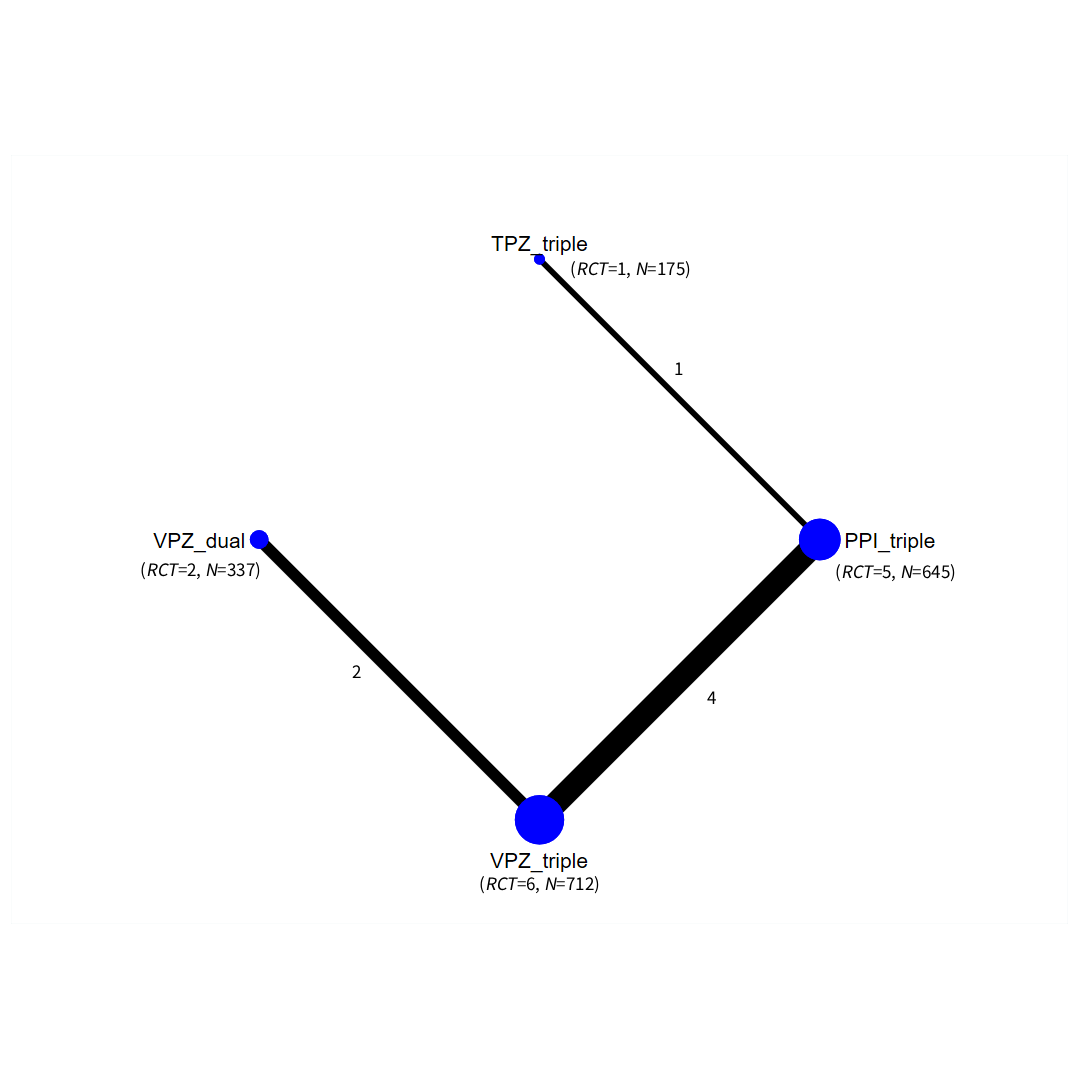


b
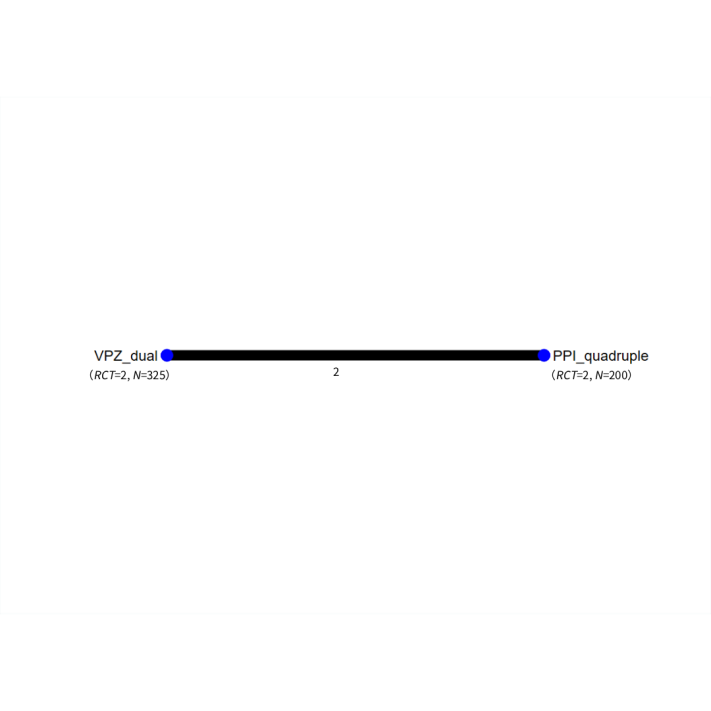


c
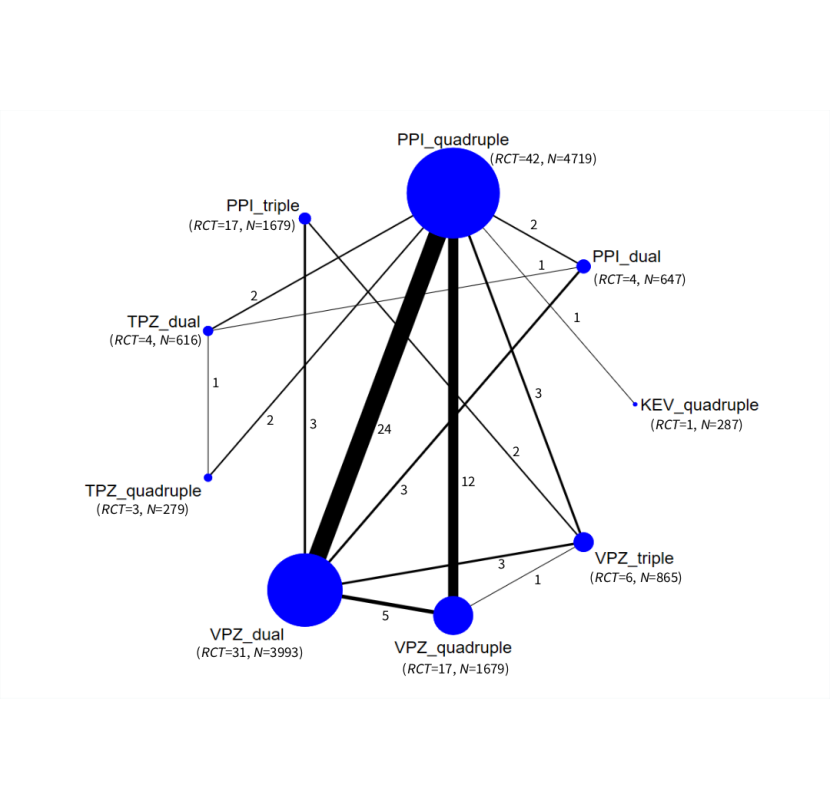


## Figure S 25 Network maps of comparisons regarding different treatment durations. a) 7-day, b) 10-day, and c) 14-day


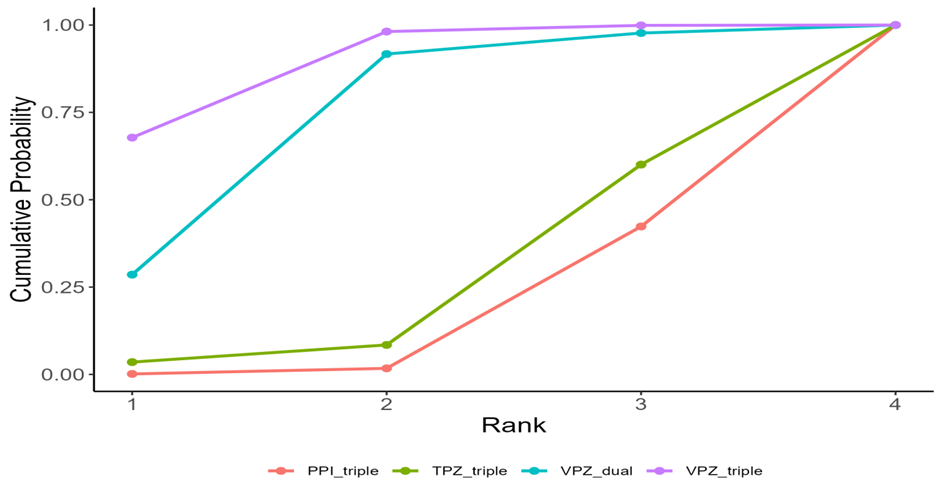


a


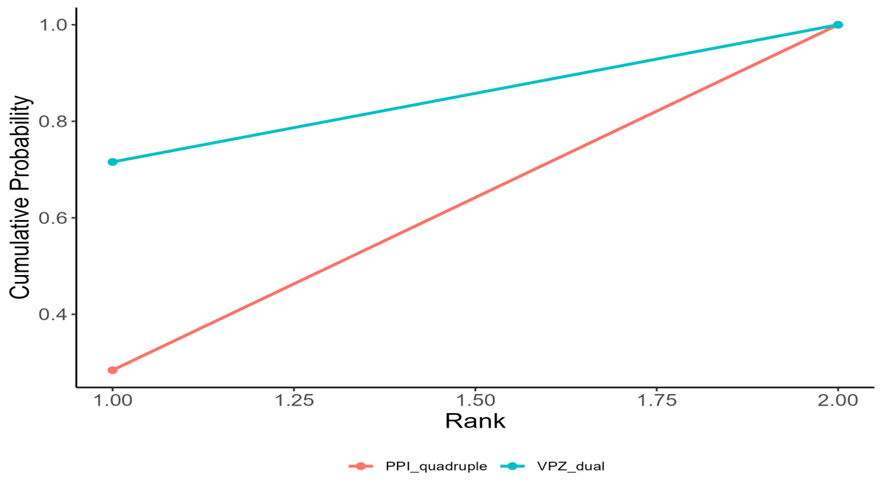


b


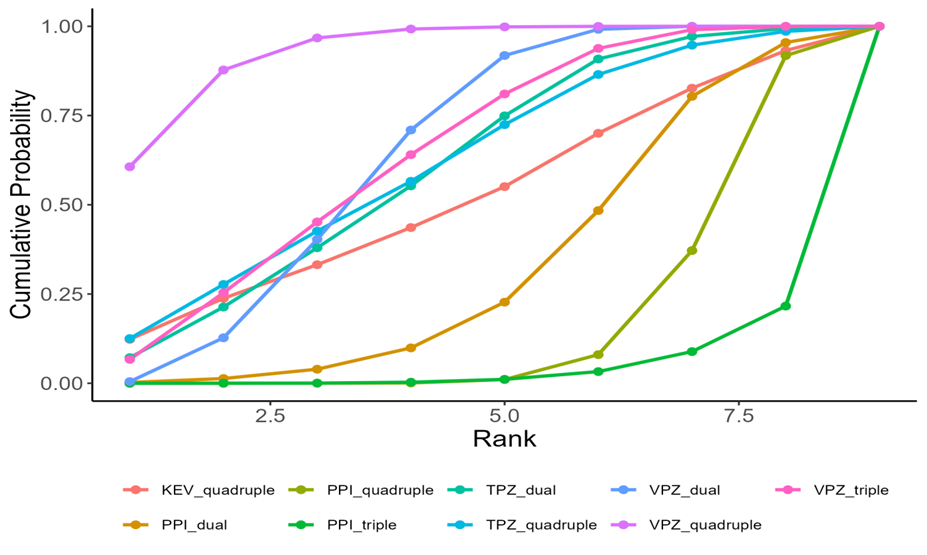


c

## Figure S 26 Ranking with SUCRA for treatments with different durations based on eradication rates. a) 7-day, b) 10-day, and c) 14-day

## Table S 14 League matrix of comparative efficacies of 14-day treatments based on eradication rate

| TPZ_dual | 1.05 (0.55, 1.95) | 0.72 (0.36, 1.39) | 1.05 (0.47, 2.24) | 0.42 (0.17, 0.96) | 0.9 (0.29, 2.74) | 1.02 (0.48, 2.22) | 1.5 (0.76, 2.97) | 0.59 (0.31, 1.06) |
| --- | --- | --- | --- | --- | --- | --- | --- | --- |
| 0.96 (0.51, 1.82) | VPZ_dual | 0.69 (0.41, 1.14) | 1 (0.6, 1.63) | 0.4 (0.21, 0.71) | 0.86 (0.32, 2.26) | 0.97 (0.47, 2.12) | 1.43 (1.02, 2.08) | 0.56 (0.44, 0.7) |
| 1.40 (0.72, 2.78) | 1.46 (0.88, 2.44) | PPI_dual | 1.46 (0.72, 2.88) | 0.59 (0.26, 1.25) | 1.25 (0.43, 3.69) | 1.42 (0.62, 3.39) | 2.09 (1.17, 3.86) | 0.82 (0.49, 1.37) |
| 0.96 (0.45, 2.14) | 1.00 (0.61, 1.67) | 0.68 (0.35, 1.39) | VPZ_triple | 0.4 (0.21, 0.74) | 0.86 (0.29, 2.53) | 0.97 (0.41, 2.44) | 1.43 (0.83, 2.62) | 0.56 (0.34, 0.93) |
| **2.39 (1.04, 5.97)** | **2.49 (1.40, 4.72)** | 1.71 (0.80, 3.89) | **2.49 (1.35, 4.79)** | PPI_triple | 2.14 (0.71, 6.88) | 2.42 (0.98, 6.67) | 3.57 (1.87, 7.54) | 1.4 (0.77, 2.7) |
| 1.11 (0.36, 3.49) | 1.16 (0.44, 3.09) | 0.80 (0.27, 2.34) | 1.17 (0.40, 3.39) | 0.47 (0.15, 1.41) | KEV_quadruple | 1.13 (0.35, 3.84) | 1.67 (0.62, 4.65) | 0.65 (0.25, 1.68) |
| 0.98 (0.45, 2.10) | 1.03 (0.47, 2.14) | 0.70 (0.29, 1.62) | 1.03 (0.41, 2.41) | 0.41 (0.15, 1.02) | 0.89 (0.26, 2.85) | TPZ_quadruple | 1.48 (0.66, 3.24) | 0.58 (0.27, 1.17) |
| 0.67 (0.34, 1.31) | **0.70 (0.48, 0.98)** | **0.48 (0.26, 0.86)** | 0.70 (0.38, 1.21) | 0.28 (0.13, 0.54) | 0.60 (0.21, 1.60) | 0.68 (0.31, 1.51) | VPZ_quadruple | 0.39 (0.27, 0.54) |
| 1.70 (0.95, 3.19) | **1.78 (1.42, 2.26)** | 1.22 (0.73, 2.05) | **1.78 (1.08, 2.92)** | 0.71 (0.37, 1.30) | 1.53 (0.60, 3.97) | 1.73 (0.86, 3.69) | **2.55 (1.86, 3.64)** | PPI_quadruple |
| Odd ratio (95% confidence interval) reported. Statistically significant data presented in bold.  *VPZ_quadruple* Vonoprazan combined with two antibiotics and Bismuth, *VPZ_triple* Vonoprazan combined with two antibiotics, *VPZ_dual* Vonoprazan combined with one antibiotic, *PPI_quadruple* Proton pump inhibitor combined with two antibiotics and Bismuth, *PPI_triple* Proton pump inhibitor combined with two antibiotics, *PPI_dual* Proton pump inhibitor combined with one antibiotic; *TPZ_dual* Tegoprazan combined with one antibiotic; *TPZ_quadruple* Tegoprazan combined with two antibiotics and Bismuth; *KEV_quadruple* Keboprazan combined with two antibiotics and Bismuth. | | | | | | | | |

## Table S 15 League matrix of comparative efficacies of 7-day treatments based on eradication rate

| VPZ_dual | 0.34 (0.05, 2.39) | 1.23 (0.43, 3.45) | 0.31 (0.08, 1.12) |
| --- | --- | --- | --- |
| 2.93 (0.42, 20.16) | TPZ_triple | 3.59 (0.72, 18.3) | 0.9 (0.22, 3.77) |
| 0.82 (0.29, 2.31) | 0.28 (0.05, 1.39) | VPZ_triple | 0.25 (0.11, 0.56) |
| 3.22 (0.89, 12.35) | 1.11 (0.27, 4.45) | **3.94 (1.79, 8.96)** | PPI_triple |
| Odd ratio (95% confidence interval) reported. Statistically significant data presented in bold.  *VPZ_dual* Vonoprazan combined with one antibiotic, *VPZ_triple* Vonoprazan combined with two antibiotics, *PPI_triple* Proton pump inhibitor combined with two antibiotics, *TPZ_triple* Tegoprazan combined with two antibiotics. | | | |

## Table S 16 League matrix of comparative efficacies of 10-day treatments based on eradication rate

| VPZ_dual | 0.82 (0.40, 1.64) |
| --- | --- |
| 1.22 (0.61, 2.51) | PPI_quadruple |
| Odd ratio (95% confidence interval) reported. Statistically significant data presented in bold.  *VPZ_dual* Vonoprazan combined with one antibiotic, *PPI_quadruple* Proton pump inhibitor combined with two antibiotics and Bismuth. | |

# Appendix 11 Subgroup analysis of eradication rates for various treatment durations-Fix

a
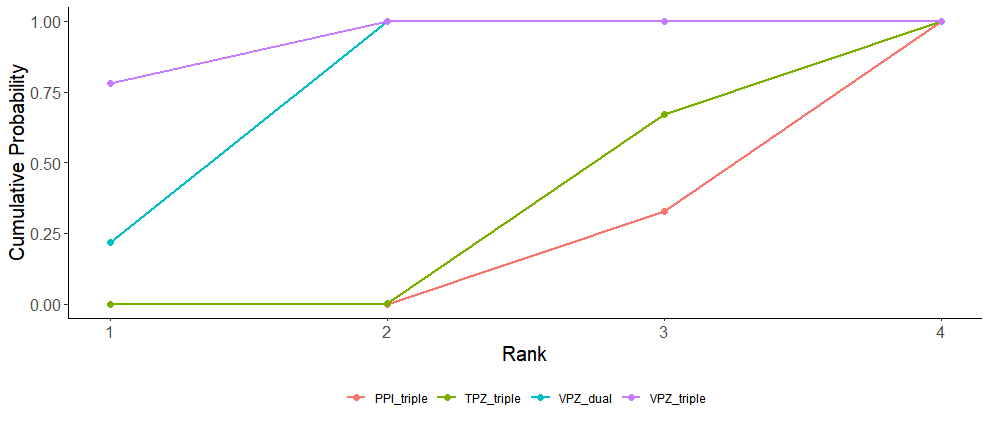


b
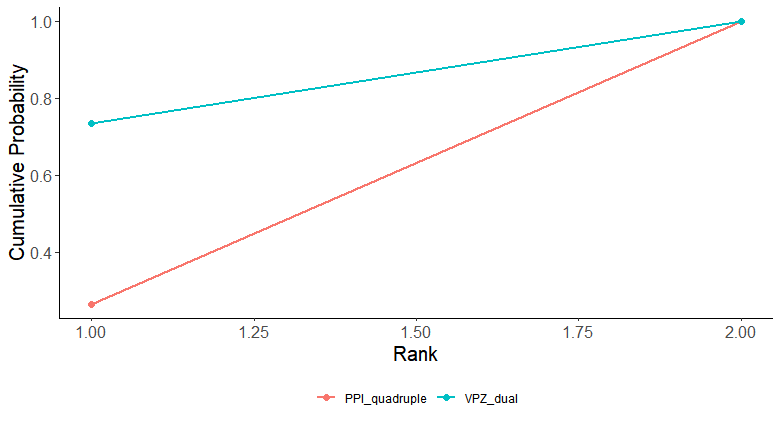


c
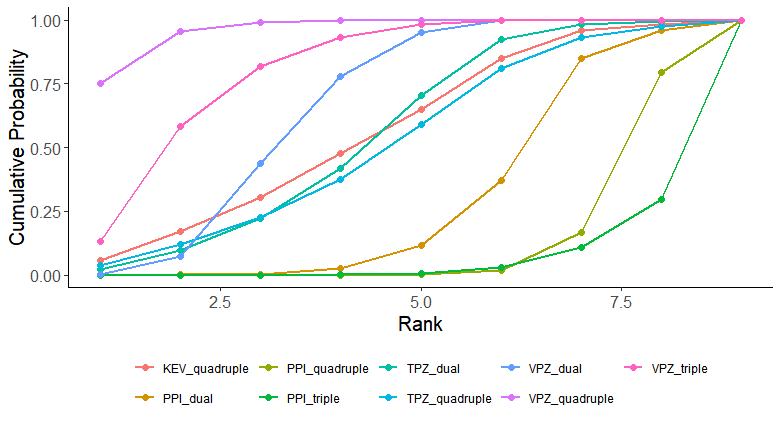


## Figure S 27 Ranking with SUCRA for treatments with different durations based on eradication rates. a) 7-day, b) 10-day, and c) 14-day

## Table S 17 League matrix of comparative efficacies of 14-day treatments based on eradication rate

| **TPZ_dual** | 1.12 (0.75, 1.66) | 0.79 (0.52, 1.18) | 1.27 (0.80, 2.02) | 0.6 (0.37, 0.98) | 1.02 (0.55, 1.88) | 0.97 (0.59, 1.58) | 1.5 (0.97, 2.32) | **0.66 (0.45, 0.97)** |
| --- | --- | --- | --- | --- | --- | --- | --- | --- |
| 0.89 (0.60, 1.34) | **VPZ_dual** | **0.7 (0.53, 0.94)** | 1.14 (0.87, 1.49) | **0.54 (0.40, 0.72)** | 0.91 (0.56, 1.50) | 0.87 (0.53, 1.43) | **1.34 (1.08, 1.68)** | **0.59 (0.52, 0.69)** |
| 1.27 (0.85, 1.91) | **1.42 (1.06, 1.89)** | **PPI_dual** | **1.61 (1.11, 2.36)** | 0.76 (0.50, 1.14) | 1.29 (0.75, 2.25) | 1.23 (0.73, 2.10) | **1.91 (1.36, 2.69)** | 0.84 (0.63, 1.12) |
| 0.79 (0.49, 1.25) | 0.88 (0.67, 1.14) | **0.62 (0.42, 0.90)** | **VPZ_triple** | **0.47 (0.35, 0.64)** | 0.8 (0.47, 1.38) | 0.76 (0.44, 1.33) | 1.18 (0.86, 1.63) | **0.52 (0.40, 0.68)** |
| **1.67 (1.02, 2.73)** | **1.86 (1.39, 2.51)** | 1.31 (0.88, 1.98) | **2.12 (1.57, 2.89)** | **PPI_triple** | 1.7 (0.97, 3.02) | 1.62 (0.92, 2.87) | **2.51 (1.76, 3.58)** | 1.11 (0.81, 1.52) |
| 0.98 (0.53, 1.81) | 1.10 (0.67, 1.8) | 0.77 (0.44, 1.34) | 1.25 (0.72, 2.15) | 0.59 (0.33, 1.03) | **KEV_quadruple** | 0.95 (0.49, 1.85) | 1.48 (0.88, 2.45) | 0.65 (0.40, 1.04) |
| 1.03 (0.63, 1.69) | 1.15 (0.70, 1.9) | 0.81 (0.48, 1.38) | 1.31 (0.75, 2.26) | 0.62 (0.35, 1.09) | 1.05 (0.54, 2.06) | **TPZ_quadruple** | 1.55 (0.92, 2.61) | 0.69 (0.42, 1.10) |
| 0.67 (0.43, 1.03) | **0.74 (0.60, 0.92)** | **0.52 (0.37, 0.74)** | 0.85 (0.61, 1.17) | **0.4 (0.28, 0.57)** | 0.68 (0.41, 1.14) | 0.64 (0.38, 1.09) | **VPZ_quadruple** | **0.44 (0.36, 0.54)** |
| **1.51 (1.03, 2.22)** | **1.68 (1.46, 1.94)** | 1.19 (0.89, 1.58) | **1.91 (1.47, 2.50)** | 0.90 (0.66, 1.23) | 1.53 (0.96, 2.47) | 1.46 (0.91, 2.37) | **2.26 (1.84, 2.78)** | **PPI_quadruple** |
| Odd ratio (95% confidence interval) reported. Statistically significant data presented in bold.  *VPZ_quadruple* Vonoprazan combined with two antibiotics and Bismuth, *VPZ_triple* Vonoprazan combined with two antibiotics, *VPZ_dual* Vonoprazan combined with one antibiotic, *PPI_quadruple* Proton pump inhibitor combined with two antibiotics and Bismuth, *PPI_triple* Proton pump inhibitor combined with two antibiotics, *PPI_dual* Proton pump inhibitor combined with one antibiotic; *TPZ_dual* Tegoprazan combined with one antibiotic; *TPZ_quadruple* Tegoprazan combined with two antibiotics and Bismuth; *KEV_quadruple* Keboprazan combined with two antibiotics and Bismuth. | | | | | | | | |

## Table S 18 League matrix of comparative efficacies of 7-day treatments based on eradication rate

| **VPZ_dual** | **0.34 (0.16, 0.70)** | 1.21 (0.78, 1.89) | **0.31 (0.17, 0.55)** |
| --- | --- | --- | --- |
| **2.95 (1.43, 6.12)** | **TPZ_triple** | **3.58 (2.02, 6.38)** | 0.91 (0.59, 1.40) |
| 0.82 (0.53, 1.28) | **0.28 (0.16, 0.5)** | **VPZ_triple** | **0.25 (0.17, 0.37)** |
| **3.26 (1.82, 5.83)** | 1.1 (0.72, 1.70) | **3.94 (2.72, 5.84)** | **PPI_triple** |
| Odd ratio (95% confidence interval) reported. Statistically significant data presented in bold.  *VPZ_dual* Vonoprazan combined with one antibiotic, *VPZ_triple* Vonoprazan combined with two antibiotics, *PPI_triple* Proton pump inhibitor combined with two antibiotics, *TPZ_triple* Tegoprazan combined with two antibiotics. | | | |

## Table S 19 League matrix of comparative efficacies of 10-day treatments based on eradication rate

| **VPZ_dual** | 0.85 (0.52, 1.42) |
| --- | --- |
| 1.17 (0.71, 1.93) | **PPI_quadruple** |
| Odd ratio (95% confidence interval) reported. Statistically significant data presented in bold.  *VPZ_dual* Vonoprazan combined with one antibiotic, *PPI_quadruple* Proton pump inhibitor combined with two antibiotics and Bismuth. | |

# Appendix 12 Subgroup analysis of eradication rates in different regions-Random


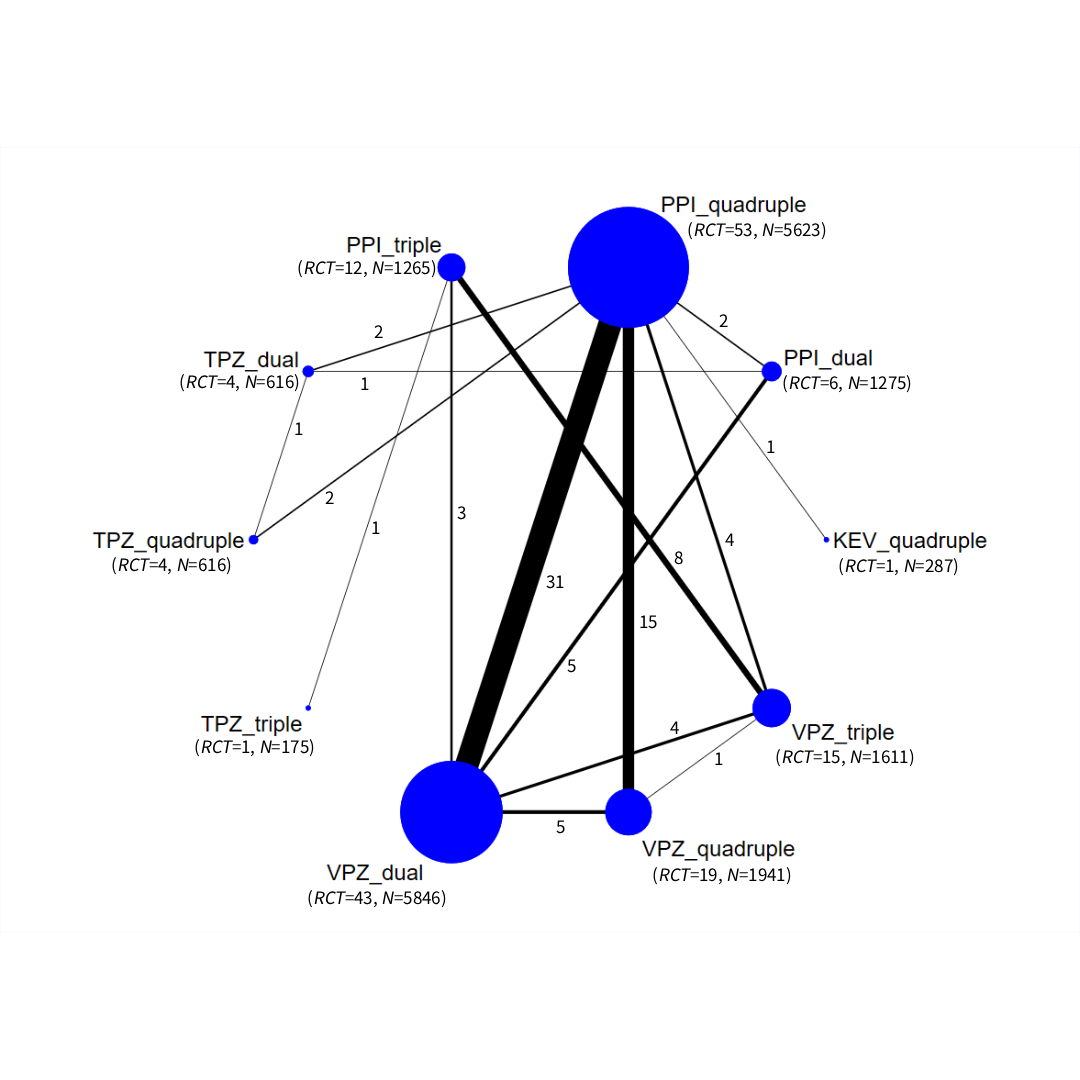


## Figure S 28 Network map of comparisons of eradication treatments in Asian population


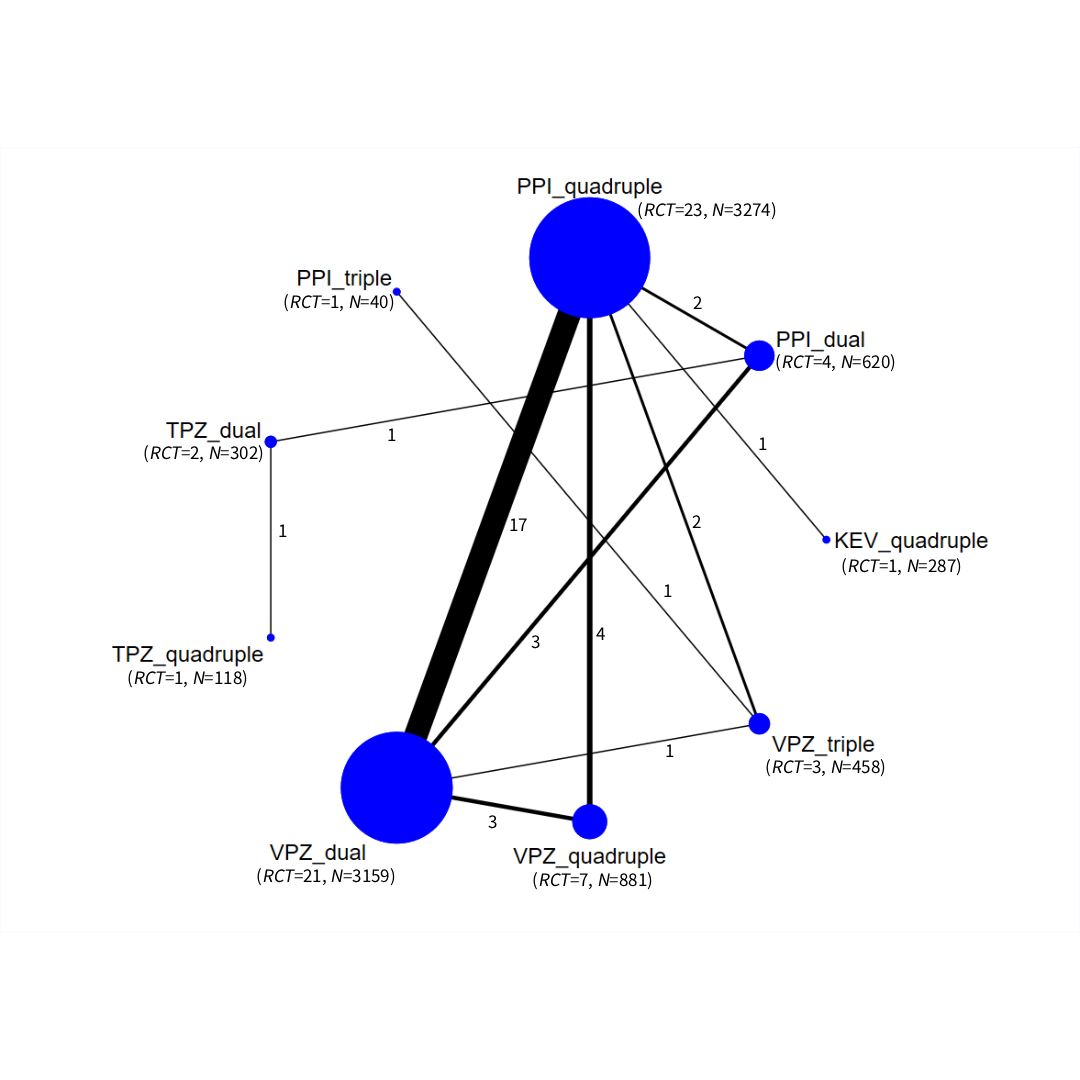


## Figure S 29 Network map of comparisons of eradication treatments in treatment-naïve Chinese patients receiving 14-day regimens


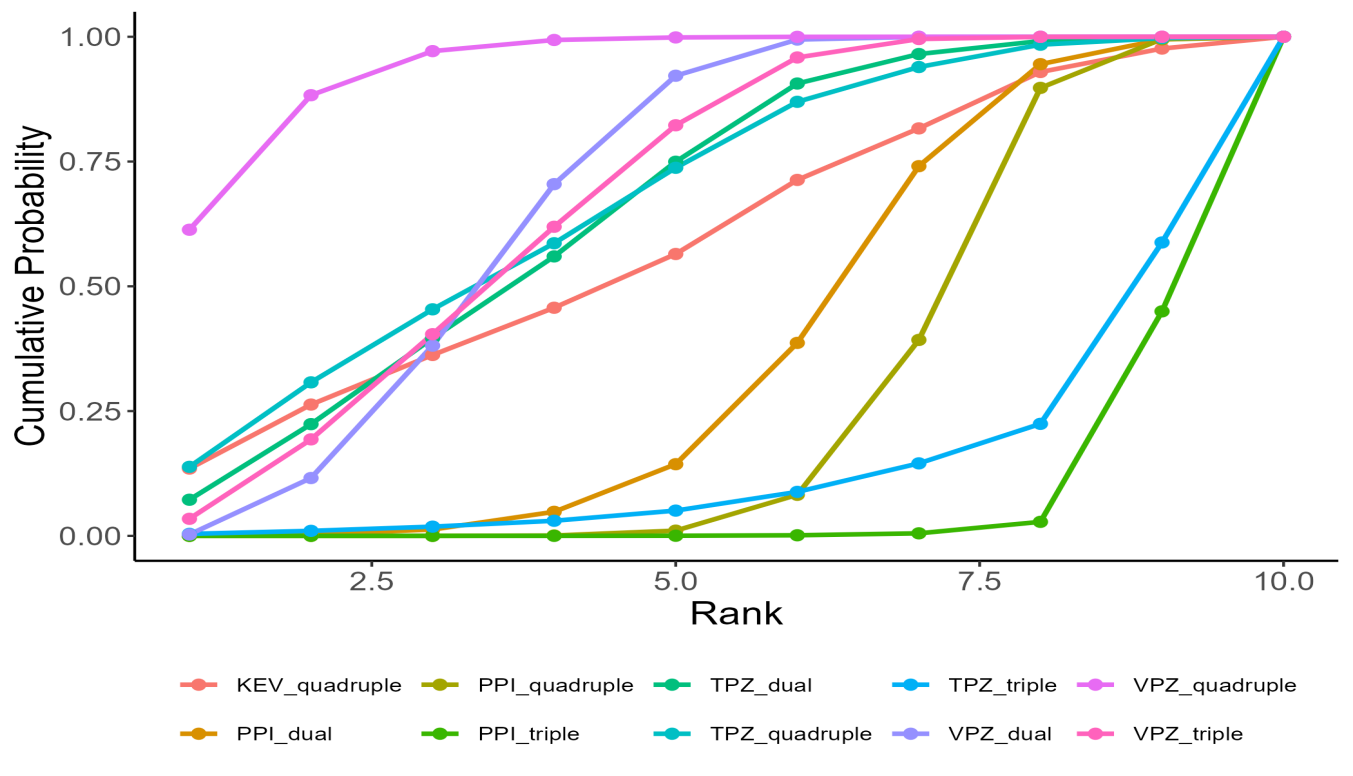


## Figure S 30 League matrix of comparative efficacies of eradication treatments in Asian patients

## Table S 20 League matrix of comparative efficacies of eradication treatments in Asian patients

| TPZ_dual | 1.03 (0.55, 1.91) | 0.68 (0.35, 1.31) | 0.36 (0.1, 1.23) | 1.02 (0.48, 2.1) | 0.33 (0.15, 0.71) | 0.91 (0.29, 2.79) | 1.03 (0.48, 2.28) | 1.5 (0.77, 2.97) | 0.6 (0.32, 1.08) |
| --- | --- | --- | --- | --- | --- | --- | --- | --- | --- |
| 0.97 (0.52, 1.83) | VPZ_dual | 0.66 (0.44, 1) | 0.35 (0.12, 1.02) | 0.99 (0.66, 1.47) | 0.32 (0.2, 0.52) | 0.88 (0.32, 2.34) | 1 (0.47, 2.17) | 1.46 (1.04, 2.06) | 0.58 (0.47, 0.7) |
| 1.46 (0.76, 2.85) | 1.51 (1.00, 2.30) | PPI_dual | 0.53 (0.17, 1.66) | 1.49 (0.85, 2.64) | 0.48 (0.26, 0.91) | 1.33 (0.46, 3.83) | 1.51 (0.67, 3.51) | 2.2 (1.32, 3.74) | 0.87 (0.56, 1.34) |
| 2.76 (0.82, 9.72) | 2.85 (0.98, 8.39) | 1.88 (0.60, 6.00) | TPZ_triple | 2.83 (0.99, 7.94) | 0.91 (0.35, 2.36) | 2.53 (0.58, 10.75) | 2.85 (0.79, 10.78) | 4.14 (1.37, 12.84) | 1.65 (0.55, 4.84) |
| 0.98 (0.48, 2.06) | 1.01 (0.68, 1.52) | 0.67 (0.38, 1.18) | 0.35 (0.13, 1.01) | VPZ_triple | 0.32 (0.21, 0.49) | 0.89 (0.31, 2.53) | 1.01 (0.44, 2.39) | 1.47 (0.9, 2.45) | 0.58 (0.39, 0.88) |
| **3.03 (1.40, 6.74)** | **3.13 (1.92, 5.11)** | **2.08 (1.10, 3.91)** | 1.10 (0.42, 2.87) | **3.10 (2.04, 4.71)** | PPI_triple | 2.77 (0.93, 8.14) | 3.13 (1.3, 7.82) | 4.56 (2.58, 8.21) | 1.81 (1.09, 3) |
| 1.10 (0.36, 3.47) | 1.13 (0.43, 3.09) | 0.75 (0.26, 2.17) | 0.39 (0.09, 1.72) | 1.12 (0.40, 3.20) | 0.36 (0.12, 1.08) | KEV_quadruple | 1.14 (0.34, 3.87) | 1.65 (0.61, 4.66) | 0.65 (0.25, 1.72) |
| 0.97 (0.44, 2.10) | 1.00 (0.46, 2.11) | 0.66 (0.28, 1.49) | 0.35 (0.09, 1.27) | 0.99 (0.42, 2.27) | **0.32 (0.13, 0.77)** | 0.88 (0.26, 2.96) | TPZ_quadruple | 1.46 (0.65, 3.24) | 0.58 (0.27, 1.19) |
| 0.67 (0.34, 1.30) | **0.69 (0.49, 0.96)** | **0.46 (0.27, 0.76)** | **0.24 (0.08, 0.73)** | 0.68 (0.41, 1.11) | **0.22 (0.12, 0.39)** | 0.61 (0.21, 1.64) | 0.69 (0.31, 1.54) | VPZ_quadruple | 0.4 (0.28, 0.54) |
| 1.68 (0.92, 3.15) | **1.73 (1.42, 2.14)** | 1.15 (0.75, 1.78) | 0.61 (0.21, 1.80) | **1.71 (1.13, 2.59)** | **0.55 (0.33, 0.92)** | 1.53 (0.58, 4.00) | 1.73 (0.84, 3.71) | **2.52 (1.86, 3.52)** | PPI_quadruple |
| Odd ratio (95% confidence interval) reported. Statistically significant data presented in bold.  *VPZ_quadruple* Vonoprazan combined with two antibiotics and Bismuth, *VPZ_triple* Vonoprazan combined with two antibiotics, *VPZ_dual* Vonoprazan combined with one antibiotic, *PPI_quadruple* Proton pump inhibitor combined with two antibiotics and Bismuth, *PPI_triple* Proton pump inhibitor combined with two antibiotics, *PPI_dual* Proton pump inhibitor combined with one antibiotic; *TPZ_dual* Tegoprazan combined with one antibiotic; *TPZ_triple* Tegoprazan combined with two antibiotics; *TPZ_quadruple* Tegoprazan combined with two antibiotics and Bismuth; *KEV_quadruple* Keboprazan combined with two antibiotics and Bismuth. | | | | | | | | | |


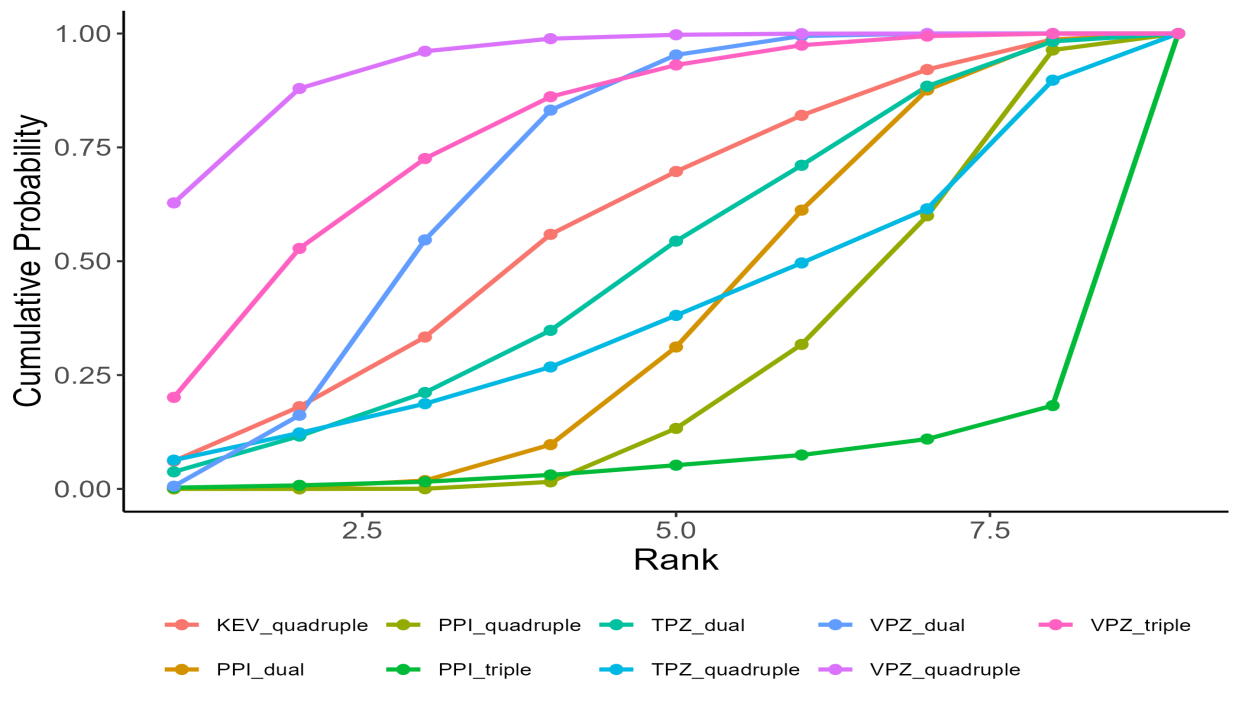


## Figure S 31 Ranking with SUCRA for eradication treatments in treatment-naïve Chinese patients receiving 14-day regimens

## Table S 21 League matrix of comparative efficacies in treatment-naïve Chinese patients receiving 14-day regimens

| TPZ_dual | 1.37 (0.54, 3.54) | 0.88 (0.39, 2) | 1.6 (0.52, 4.94) | 0.27 (0.04, 1.67) | 1.18 (0.35, 3.92) | 0.83 (0.34, 2.03) | 2.05 (0.74, 5.62) | 0.77 (0.3, 1.98) |
| --- | --- | --- | --- | --- | --- | --- | --- | --- |
| 0.73 (0.28, 1.85) | VPZ_dual | 0.64 (0.4, 1.02) | 1.17 (0.6, 2.24) | 0.2 (0.03, 0.95) | 0.86 (0.39, 1.85) | 0.61 (0.16, 2.23) | 1.5 (0.99, 2.2) | 0.56 (0.44, 0.7) |
| 1.13 (0.5, 2.57) | 1.55 (0.98, 2.5) | PPI_dual | 1.81 (0.83, 3.94) | 0.31 (0.05, 1.57) | 1.33 (0.55, 3.23) | 0.95 (0.28, 3.2) | 2.32 (1.28, 4.19) | 0.87 (0.54, 1.41) |
| 0.63 (0.2, 1.93) | 0.86 (0.45, 1.66) | 0.55 (0.25, 1.21) | VPZ_triple | 0.17 (0.03, 0.71) | 0.74 (0.28, 1.96) | 0.52 (0.13, 2.23) | 1.29 (0.61, 2.67) | 0.48 (0.26, 0.9) |
| 3.65 (0.6, 28.46) | **5.01 (1.05, 30.6)** | 3.23 (0.64, 20.48) | **5.83 (1.42, 31.68)** | PPI_triple | 4.34 (0.76, 30.21) | 3.07 (0.41, 27.51) | 7.52 (1.51, 46.97) | 2.8 (0.59, 17.1) |
| 0.85 (0.25, 2.85) | 1.16 (0.54, 2.58) | 0.75 (0.31, 1.83) | 1.36 (0.51, 3.62) | 0.23 (0.03, 1.31) | KEV_quadruple | 0.71 (0.16, 3.2) | 1.75 (0.75, 4.05) | 0.65 (0.31, 1.38) |
| 1.2 (0.49, 2.95) | 1.64 (0.45, 6.07) | 1.05 (0.31, 3.57) | 1.91 (0.45, 7.98) | 0.33 (0.04, 2.44) | 1.41 (0.31, 6.29) | TPZ_quadruple | 2.44 (0.63, 9.44) | 0.91 (0.25, 3.37) |
| 0.49 (0.18, 1.36) | 0.67 (0.45, 1.01) | **0.43 (0.24, 0.78)** | 0.78 (0.38, 1.64) | **0.13 (0.02, 0.66)** | 0.57 (0.25, 1.34) | 0.41 (0.11, 1.59) | VPZ_quadruple | 0.37 (0.25, 0.56) |
| 1.3 (0.5, 3.39) | **1.79 (1.43, 2.25)** | 1.15 (0.71, 1.86) | **2.08 (1.12, 3.9)** | 0.36 (0.06, 1.68) | 1.53 (0.72, 3.22) | 1.09 (0.3, 4.01) | **2.67 (1.77, 3.97)** | PPI_quadruple |
| Odd ratio (95% confidence interval) reported. Statistically significant data presented in bold.  *VPZ_quadruple* Vonoprazan combined with two antibiotics and Bismuth, *VPZ_triple* Vonoprazan combined with two antibiotics, *VPZ_dual* Vonoprazan combined with one antibiotic, *PPI_quadruple* Proton pump inhibitor combined with two antibiotics and Bismuth, *PPI_triple* Proton pump inhibitor combined with two antibiotics, *PPI_dual* Proton pump inhibitor combined with one antibiotic; *TPZ_dual* Tegoprazan combined with one antibiotic; *TPZ_quadruple* Tegoprazan combined with two antibiotics and Bismuth; *KEV_quadruple* Keboprazan combined with two antibiotics and Bismuth | | | | | | | | |

# Appendix 13 Subgroup analysis of eradication rates in different regions-Fix


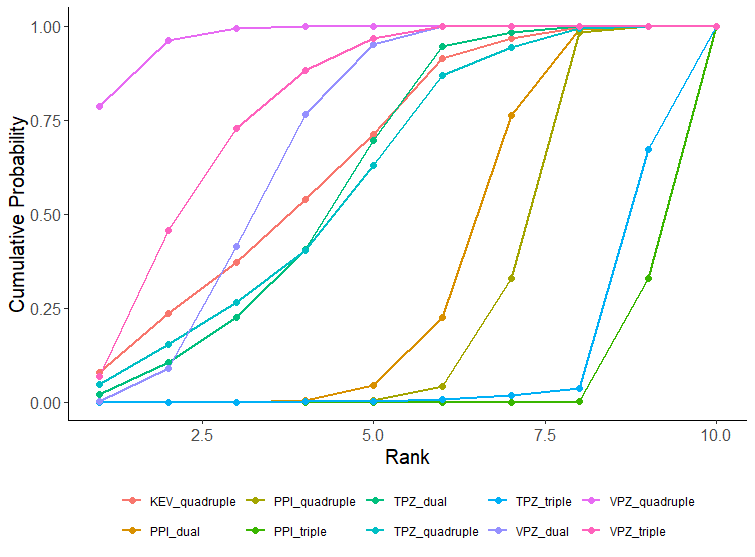


## Figure S 32 Ranking with SUCRA for eradication treatments in Asian population

## Table S 22 League matrix of comparative efficacies of eradication treatments in Asian patients

| **TPZ_dual** | 1.11 (0.76, 1.64) | 0.75 (0.51, 1.10) | 0.41 (0.21, 0.78) | 1.21 (0.78, 1.90) | **0.37 (0.23, 0.59)** | 1.05 (0.58, 1.93) | 0.99 (0.60, 1.62) | 1.5 (0.99, 2.29) | 0.69 (0.47, 1.01) |
| --- | --- | --- | --- | --- | --- | --- | --- | --- | --- |
| 0.90 (0.61, 1.32) | **VPZ_dual** | **0.67 (0.54, 0.82)** | **0.36 (0.22, 0.62)** | 1.09 (0.85, 1.39) | **0.33 (0.24, 0.44)** | 0.95 (0.59, 1.55) | 0.89 (0.55, 1.44) | **1.35 (1.10, 1.66)** | **0.62 (0.55, 0.7)** |
| 1.34 (0.91, 1.98) | **1.49 (1.22, 1.84)** | **PPI_dual** | **0.54 (0.31, 0.96)** | **1.63 (1.19, 2.24)** | **0.49 (0.34, 0.71)** | 1.42 (0.85, 2.4) | 1.33 (0.08, 2.21) | **2.02 (1.52, 2.67)** | 0.92 (0.74, 1.16) |
| **2.47 (1.28, 4.73)** | **2.74 (1.62, 4.64)** | **1.84 (1.04, 3.23)** | **TPZ_triple** | **2.98 (1.80, 4.97)** | 0.91 (0.59, 1.40) | **2.60 (1.28, 5.33)** | **2.43 (1.20, 4.99)** | **3.70 (2.12, 6.51)** | 1.70 (0.99, 2.90) |
| 0.83 (0.53, 1.29) | 0.92 (0.72, 1.17) | **0.62 (0.45, 0.84)** | **0.34 (0.20, 0.56)** | **VPZ_triple** | **0.3 (0.23, 0.40)** | 0.87 (0.51, 1.49) | 0.82 (0.48, 1.40) | 1.24 (0.92, 1.68) | **0.57 (0.44, 0.73)** |
| **2.72 (1.68, 4.42)** | **3.02 (2.25, 4.10)** | **2.03 (1.41, 2.93)** | 1.10 (0.72, 1.70) | **3.29 (2.52, 4.35)** | **PPI_triple** | **2.87 (1.64, 5.07)** | **2.69 (1.52, 4.77)** | **4.09 (2.87, 5.85)** | **1.87 (1.37, 2.57)** |
| 0.95 (0.52, 1.72) | 1.05 (0.64, 1.71) | 0.71 (0.42, 1.18) | **0.38 (0.19, 0.78)** | 1.15 (0.67, 1.94) | **0.35 (0.20, 0.61)** | **KEV_quadruple** | 0.94 (0.48, 1.82) | 1.42 (0.85, 2.35) | 0.65 (0.41, 1.04) |
| 1.01 (0.62, 1.66) | 1.13 (0.69, 1.83) | 0.75 (0.45, 1.25) | **0.41 (0.20, 0.84)** | 1.22 (0.71, 2.09) | **0.37 (0.21, 0.66)** | 1.07 (0.55, 2.09) | **TPZ_quadruple** | 1.52 (0.91, 2.53) | 0.70 (0.43, 1.12) |
| 0.67 (0.44, 1.01) | **0.74 (0.60, 0.91)** | **0.50 (0.37, 0.66)** | **0.27 (0.15, 0.47)** | 0.81 (0.60, 1.09) | **0.24 (0.17, 0.35)** | 0.70 (0.43, 1.17) | 0.66 (0.40, 1.10) | **VPZ_quadruple** | **0.46 (0.38, 0.56)** |
| 1.45 (0.99, 2.11) | **1.61 (1.43, 1.83)** | 1.08 (0.86, 1.35) | 0.59 (0.35, 1.01) | **1.75 (1.37, 2.26)** | **0.53 (0.39, 0.73)** | 1.53 (0.96, 2.46) | 1.43 (0.89, 2.31) | **2.18 (1.79, 2.65)** | **PPI_quadruple** |
| Odd ratio (95% confidence interval) reported. Statistically significant data presented in bold.  *VPZ_quadruple* Vonoprazan combined with two antibiotics and Bismuth, *VPZ_triple* Vonoprazan combined with two antibiotics, *VPZ_dual* Vonoprazan combined with one antibiotic, *PPI_quadruple* Proton pump inhibitor combined with two antibiotics and Bismuth, *PPI_triple* Proton pump inhibitor combined with two antibiotics, *PPI_dual* Proton pump inhibitor combined with one antibiotic; *TPZ_dual* Tegoprazan combined with one antibiotic; *TPZ_triple* Tegoprazan combined with two antibiotics; *TPZ_quadruple* Tegoprazan combined with two antibiotics and Bismuth; *KEV_quadruple* Keboprazan combined with two antibiotics and Bismuth. | | | | | | | | | |


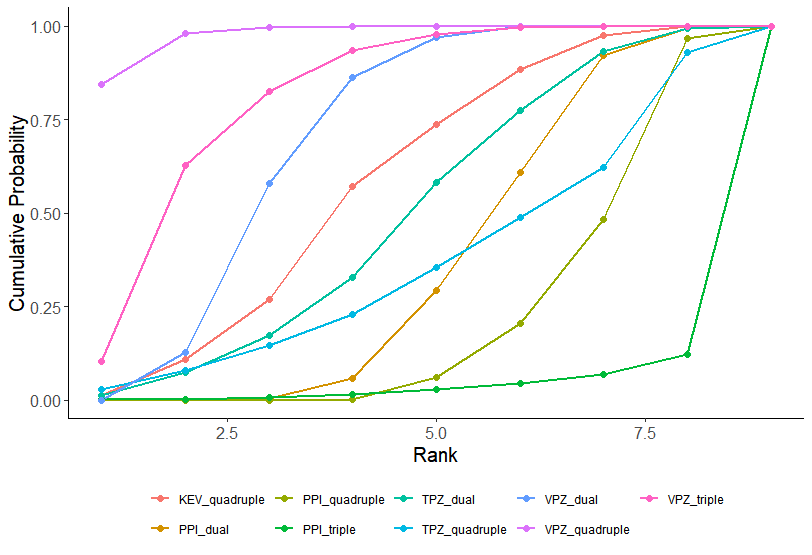


## Figure S 33 Ranking with SUCRA for eradication treatments in treatment-naïve Chinese patients receiving 14-day regimens

## Table S 23 League matrix of comparative efficacies in treatment-naïve Chinese patients receiving 14-day regimens

| **TPZ_dual** | 1.31 (0.68, 2.54) | 0.88 (0.49, 1.56) | 1.55 (0.72, 3.36) | 0.27 (0.04, 1.21) | 1.14 (0.51, 2.55) | 0.84 (0.42, 1.65) | **2.11 (1.04, 4.31)** | 0.74 (0.38, 1.45) |
| --- | --- | --- | --- | --- | --- | --- | --- | --- |
| 0.76 (0.39, 1.46) | **VPZ_dual** | **0.67 (0.49, 0.91)** | 1.18 (0.77, 1.83) | **0.20 (0.04, 0.79)** | 0.87 (0.53, 1.42) | 0.64 (0.25, 1.62) | **1.61 (1.22, 2.14)** | **0.57 (0.48, 0.67)** |
| 1.14 (0.64, 2.03) | **1.50 (1.09, 2.04)** | **PPI_dual** | **1.76 (1.06, 2.95)** | 0.30 (0.06, 1.22) | 1.30(0.74, 2.29) | 0.95 (0.39, 2.31) | **2.41 (1.59, 3.63)** | 0.85 (0.62, 1.17) |
| 0.65 (0.30, 1.40) | 0.85 (0.55, 1.31) | **0.57 (0.34, 0.95)** | **VPZ_triple** | **0.17 (0.03, 0.63)** | 0.74 (0.40, 1.37) | 0.54 (0.19, 1.51) | 1.37 (0.83, 2.24) | **0.48 (0.32, 0.72)** |
| 3.76 (0.83, 22.34) | **4.91 (1.26, 26.44)** | 3.28 (0.82, 17.97) | **5.77 (1.59, 29.20)** | **PPI_triple** | 4.26 (1.01, 23.97) | 3.17 (0.60, 20.95) | **7.91 (1.98, 42.95)** | 2.78 (0.73, 14.82) |
| 0.88 (0.39, 1.95) | 1.15 (0.70, 1.89) | 0.77 (0.44, 1.36) | 1.36 (0.73, 2.52) | **0.23 (0.04, 0.99)** | **KEV_quadruple** | 0.74 (0.26, 2.10) | **1.86 (1.07, 3.21)** | 0.65 (0.41, 1.04) |
| 1.19 (0.61, 2.39) | 1.57 (0.62, 4.08) | 1.05 (0.43, 2.58) | 1.85 (0.66, 5.23) | 0.32 (0.05, 1.67) | 1.36 (0.48, 3.92) | **TPZ_quadruple** | 2.52 (0.96, 6.77) | 0.89 (0.35, 2.32) |
| **0.47 (0.23, 0.96)** | **0.62 (0.47, 0.82)** | **0.42 (0.28, 0.63)** | 0.73 (0.45, 1.21) | **0.13 (0.02, 0.51)** | **0.54 (0.31, 0.93)** | 0.40 (0.15, 1.04) | **VPZ_quadruple** | **0.35 (0.26, 0.47)** |
| 1.35 (0.69, 2.60) | **1.77 (1.50, 2.08)** | 1.18 (0.86, 1.62) | **2.08 (1.40, 3.13)** | 0.36 (0.07, 1.38) | 1.53 (0.96, 2.45) | 1.13 (0.43, 2.88) | **2.84 (2.11, 3.83)** | **PPI_quadruple** |
| Odd ratio (95% confidence interval) reported. Statistically significant data presented in bold.  *VPZ_quadruple* Vonoprazan combined with two antibiotics and Bismuth, *VPZ_triple* Vonoprazan combined with two antibiotics, *VPZ_dual* Vonoprazan combined with one antibiotic, *PPI_quadruple* Proton pump inhibitor combined with two antibiotics and Bismuth, *PPI_triple* Proton pump inhibitor combined with two antibiotics, *PPI_dual* Proton pump inhibitor combined with one antibiotic; *TPZ_dual* Tegoprazan combined with one antibiotic; *TPZ_quadruple* Tegoprazan combined with two antibiotics and Bismuth; *KEV_quadruple* Keboprazan combined with two antibiotics and Bismuth. | | | | | | | | |

# Appendix 14 Compliance rate of various regimens-Random

## Table S 24 League matrix of compliance rate in eradication treatments

| TPZ_dual | 1.04 (0.25, 4.3) | 0.54 (0.12, 2.33) | 0.87 (0.1, 5.52) | 0.5 (0.1, 2.49) | 0.79 (0.14, 5.37) | 0.54 (0.13, 2.07) |
| --- | --- | --- | --- | --- | --- | --- |
| 0.96 (0.23, 4.01) | **VPZ_dual** | 0.52 (0.2, 1.31) | 0.84 (0.16, 3.15) | 0.48 (0.06, 4.05) | 0.76 (0.26, 2.58) | 0.52 (0.28, 0.83) |
| 1.84 (0.43, 8.14) | 1.91 (0.76, 4.94) | **PPI_dual** | 1.6 (0.24, 7.84) | 0.92 (0.11, 8) | 1.46 (0.36, 7.04) | 0.99 (0.33, 2.73) |
| 1.14 (0.18, 9.84) | 1.2 (0.32, 6.18) | 0.62 (0.13, 4.16) | **VPZ_triple** | 0.57 (0.05, 8.57) | 0.91 (0.17, 7.84) | 0.62 (0.16, 2.91) |
| 2 (0.4, 10.17) | 2.08 (0.25, 17.75) | 1.09 (0.13, 9.5) | 1.76 (0.12, 19.06) | **TPZ_quadruple** | 1.57 (0.16, 19.97) | 1.08 (0.12, 8.67) |
| 1.27 (0.19, 7.3) | 1.32 (0.39, 3.82) | 0.69 (0.14, 2.75) | 1.1 (0.13, 5.82) | 0.64 (0.05, 6.28) | **VPZ_quadruple** | 0.69 (0.16, 2.08) |
| 1.86 (0.48, 7.87) | **1.93 (1.2, 3.51)** | 1.01 (0.37, 3.01) | 1.61 (0.34, 6.18) | 0.93 (0.12, 8.05) | 1.46 (0.48, 6.17) | **PPI_quadruple** |
| Odd ratio (95% confidence interval) reported. Statistically significant data presented in bold.  *VPZ_quadruple* Vonoprazan combined with two antibiotics and Bismuth, *VPZ_triple* Vonoprazan combined with two antibiotics, *VPZ_dual* Vonoprazan combined with one antibiotic, *PPI_quadruple* Proton pump inhibitor combined with two antibiotics and Bismuth, *PPI_dual* Proton pump inhibitor combined with one antibiotic; *TPZ_dual* Tegoprazan combined with one antibiotic; *TPZ_quadruple* Tegoprazan combined with two antibiotics and Bismuth. | | | | | | |

# Appendix 15 Compliance rate of various regimens-Fix


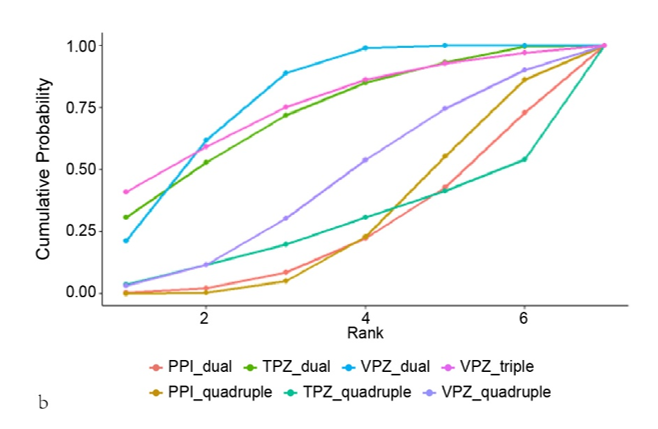


## Figure S 34 Ranking with SUCRA for eradication treatments regarding compliance

## Table S 25 League matrix of compliance rate in eradication treatments

| **TPZ_dual** | 1.03 (0.38, 2.81) | 0.54 (0.19, 1.53) | 1.11 (0.28, 4.65) | 0.50 (0.18, 1.28) | 0.69 (0.22, 2.24) | 0.58 (0.22, 1.57) |
| --- | --- | --- | --- | --- | --- | --- |
| 0.97 (0.36, 2.64) | **VPZ_dual** | **0.53 (0.28, 0.97)** | 1.07 (0.41, 3.13) | 0.48 (0.11, 1.91) | 0.67 (0.37, 1.21) | **0.56 (0.41, 0.77)** |
| 1.85 (0.65, 5.40) | **1.90 (1.03, 3.62)** | **PPI_dual** | 2.05 (0.66, 7.04) | 0.91 (0.21, 3.79) | 1.28 (0.55, 3.06) | 1.07 (0.55, 2.16) |
| 0.90 (0.22, 3.51) | 0.94 (0.32, 2.42) | 0.49 (0.14, 1.52) | **VPZ_triple** | 0.44 (0.08, 2.33) | 0.63 (0.18, 1.93) | 0.53 (0.18, 1.34) |
| 2.00 (0.78, 5.61) | 2.08 (0.52, 8.74) | 1.09 (0.26, 4.74) | 2.25 (0.43, 13.15) | **TPZ_quadruple** | 1.40 (0.31, 6.71) | 1.17 (0.30, 4.85) |
| 1.44 (0.45, 4.62) | 1.48 (0.83, 2.70) | 0.78 (0.33, 1.83) | 1.58 (0.52, 5.45) | 0.71 (0.15, 3.21) | **VPZ_quadruple** | 0.84 (0.43, 1.64) |
| 1.72 (0.64, 4.59) | **1.77 (1.30, 2.43)** | 0.93 (0.46, 1.83) | 1.90 (0.75, 5.51) | 0.85 (0.21, 3.36) | 1.20 (0.61, 2.31) | **PPI_quadruple** |
| Odd ratio (95% confidence interval) reported. Statistically significant data presented in bold.  *VPZ_quadruple* Vonoprazan combined with two antibiotics and Bismuth, *VPZ_triple* Vonoprazan combined with two antibiotics, *VPZ_dual* Vonoprazan combined with one antibiotic, *PPI_quadruple* Proton pump inhibitor combined with two antibiotics and Bismuth, *PPI_dual* Proton pump inhibitor combined with one antibiotic; *TPZ_dual* Tegoprazan combined with one antibiotic; *TPZ_quadruple* Tegoprazan combined with two antibiotics and Bismuth. | | | | | | |

# Appendix 16 Adverse events of various regimens-Random

## Table S 26 League matrix of adverse events in eradication treatments

| **TPZ_dual** | 0.96 (0.59, 1.54) | 1.14 (0.68, 1.86) | 1.75 (0.98, 3.11) | 2.07 (0.82, 4.96) | 2.46 (1.28, 4.8) | 2.36 (1.34, 4.11) | 2.61 (1.63, 4.13) |
| --- | --- | --- | --- | --- | --- | --- | --- |
| 1.04 (0.65, 1.69) | **VPZ_dual** | 1.19 (0.85, 1.66) | 1.82 (1.29, 2.61) | 2.15 (0.99, 4.59) | 2.57 (1.43, 4.7) | 2.46 (1.77, 3.43) | 2.72 (2.3, 3.23) |
| 0.88 (0.54, 1.46) | 0.84 (0.6, 1.18) | **PPI_dual** | 1.53 (0.96, 2.5) | 1.81 (0.78, 4.12) | 2.16 (1.13, 4.19) | 2.07 (1.32, 3.3) | 2.28 (1.62, 3.28) |
| 0.57 (0.32, 1.02) | **0.55 (0.38, 0.78)** | 0.65 (0.4, 1.05) | **VPZ_triple** | 1.18 (0.58, 2.36) | 1.4 (0.73, 2.76) | 1.35 (0.86, 2.11) | 1.49 (1.04, 2.11) |
| 0.48 (0.2, 1.22) | 0.46 (0.22, 1.01) | 0.55 (0.24, 1.28) | 0.85 (0.42, 1.73) | **PPI_triple** | 1.19 (0.47, 3.14) | 1.14 (0.51, 2.62) | 1.26 (0.59, 2.76) |
| **0.41 (0.21, 0.78)** | **0.39 (0.21, 0.7)** | **0.46 (0.24, 0.89)** | 0.71 (0.36, 1.38) | 0.84 (0.32, 2.15) | **TPZ_quadruple** | 0.96 (0.5, 1.85) | 1.06 (0.59, 1.87) |
| **0.42 (0.24, 0.75)** | **0.41 (0.29, 0.56)** | **0.48 (0.3, 0.76)** | 0.74 (0.47, 1.16) | 0.88 (0.38, 1.96) | 1.04 (0.54, 2.01) | **VPZ_quadruple** | 1.1 (0.8, 1.52) |
| **0.38 (0.24, 0.61)** | **0.37 (0.31, 0.43)** | **0.44 (0.31, 0.62)** | **0.67 (0.47, 0.96)** | 0.79 (0.36, 1.69) | 0.95 (0.54, 1.68) | 0.91 (0.66, 1.26) | **PPI_quadruple** |
| Odd ratio (95% confidence interval) reported. Statistically significant data presented in bold.  *VPZ_quadruple* Vonoprazan combined with two antibiotics and Bismuth, *VPZ_triple* Vonoprazan combined with two antibiotics, *VPZ_dual* Vonoprazan combined with one antibiotic, *PPI_quadruple* Proton pump inhibitor combined with two antibiotics and Bismuth, *PPI_triple* Proton pump inhibitor combined with two antibiotics, *PPI_dual* Proton pump inhibitor combined with one antibiotic; *TPZ_dual* Tegoprazan combined with one antibiotic; *TPZ_quadruple* Tegoprazan combined with two antibiotics and Bismuth. | | | | | | | |

# Appendix 17 Adverse events of various regimens-Fix

**
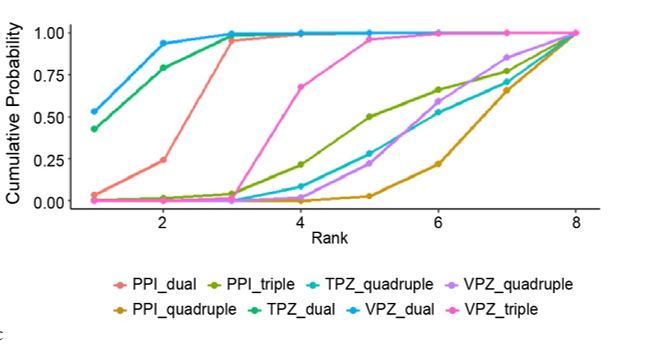
**

## Figure S 35 Ranking with SUCRA for eradication treatments regarding adverse events

## Table S 27 League matrix of adverse events in eradication treatments

| **TPZ_dual** | 0.97 (0.68, 1.41) | 1.17 (0.81, 1.71) | **1.74 (1.12, 2.69)** | **2.19 (1.06, 4.53)** | **2.43 (1.45, 4.14)** | **2.40 (1.56, 3.71)** | **2.62 (1.84, 3.76)** |
| --- | --- | --- | --- | --- | --- | --- | --- |
| 1.03 (0.71, 1.48) | **VPZ_dual** | 1.2 0(0.93, 1.55) | **1.79 (1.38, 2.32)** | **2.24 (1.18, 4.25)** | **2.5 (1.57, 4.00)** | **2.46 (1.92, 3.18)** | **2.70 (2.37, 3.07)** |
| 0.85 (0.59, 1.24) | 0.83 (0.64, 1.08) | **PPI_dual** | **1.49 (1.04, 2.13)** | 1.86 (0.94, 3.71) | **2.08 (1.25, 3.48)** | **2.05 (1.44, 2.93)** | **2.24 (1.72, 2.94)** |
| **0.57 (0.37, 0.89)** | **0.56 (0.43, 0.73)** | **0.67 (0.47, 0.96)** | **VPZ_triple** | 1.25 (0.69, 2.28) | 1.40 (0.83, 2.36) | 1.38 (0.98, 1.94) | **1.51 (1.16, 1.96)** |
| **0.46 (0.22, 0.94)** | **0.45 (0.24, 0.85)** | 0.54 (0.27, 1.06) | 0.80 (0.44, 1.44) | **PPI_triple** | 1.12 (0.51, 2.44) | 1.10 (0.56, 2.18) | 1.20 (0.63, 2.28) |
| **0.41 (0.24, 0.69)** | **0.40 (0.25, 0.64)** | **0.48 (0.29, 0.80)** | 0.72 (0.42, 1.20) | 0.90 (0.41, 1.95) | **TPZ_quadruple** | 0.99 (0.58, 1.65) | 1.08 (0.68, 1.69) |
| **0.42 (0.27, 0.64)** | **0.41 (0.31, 0.52)** | **0.49 (0.34, 0.69)** | 0.72 (0.51, 1.02) | 0.91 (0.46, 1.80) | 1.01 (0.61, 1.71) | **VPZ_quadruple** | 1.09 (0.84, 1.41) |
| **0.38 (0.27, 0.54)** | **0.37 (0.33, 0.42)** | **0.45 (0.34, 0.58)** | **0.66 (0.51, 0.86)** | 0.83 (0.44, 1.58) | 0.93 (0.59, 1.46) | 0.91 (0.71, 1.19) | **PPI_quadruple** |
| Odd ratio (95% confidence interval) reported. Statistically significant data presented in bold.  *VPZ_quadruple* Vonoprazan combined with two antibiotics and Bismuth, *VPZ_triple* Vonoprazan combined with two antibiotics, *VPZ_dual* Vonoprazan combined with one antibiotic, *PPI_quadruple* Proton pump inhibitor combined with two antibiotics and Bismuth, *PPI_triple* Proton pump inhibitor combined with two antibiotics, *PPI_dual* Proton pump inhibitor combined with one antibiotic; *TPZ_dual* Tegoprazan combined with one antibiotic; *TPZ_quadruple* Tegoprazan combined with two antibiotics and Bismuth. | | | | | | | |

# Appendix 18 Treatment discontinuation due to adverse events of various regimens - Random


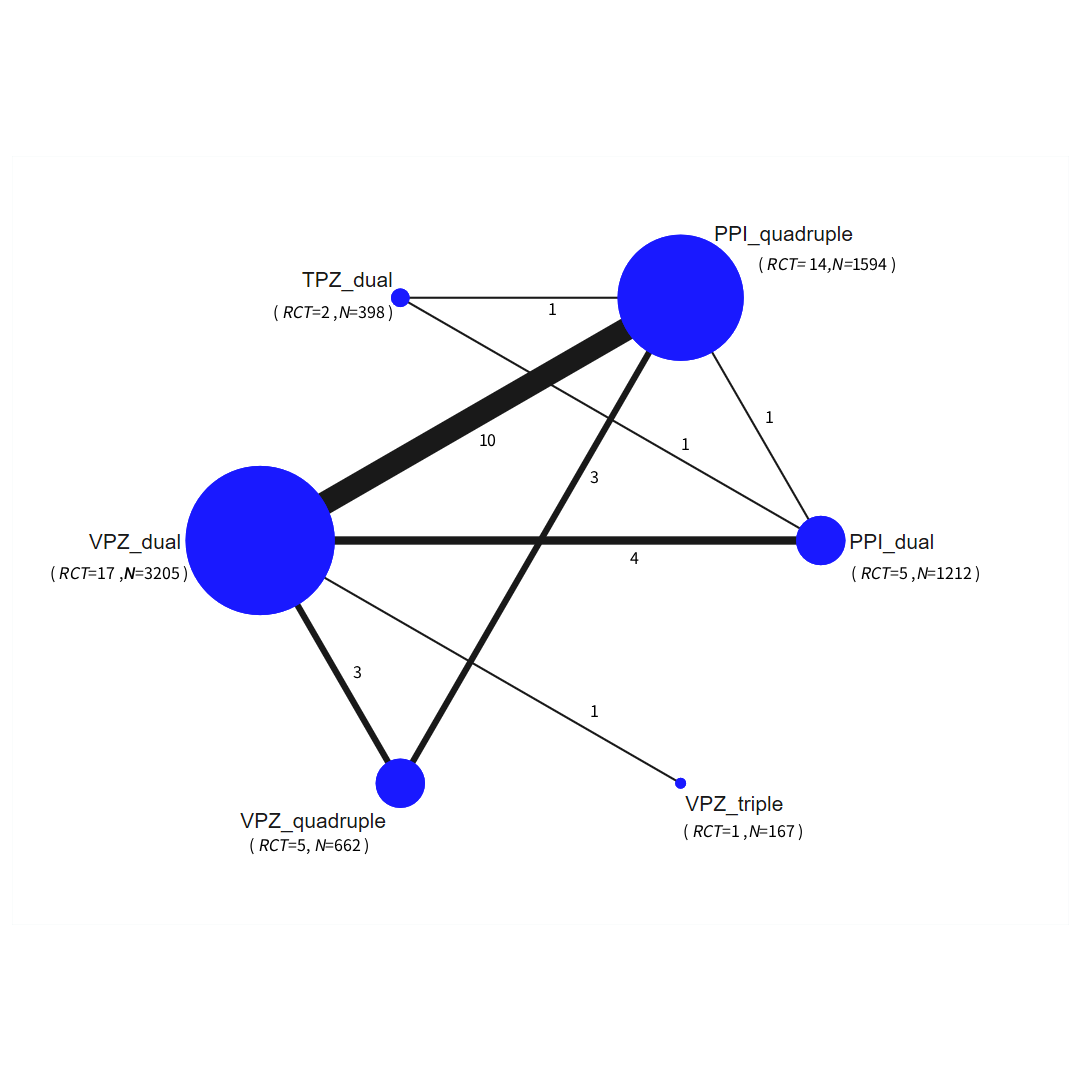


## Figure S 36 Network map of PCAB-based eradication treatment on treatment discontinuation due to adverse events


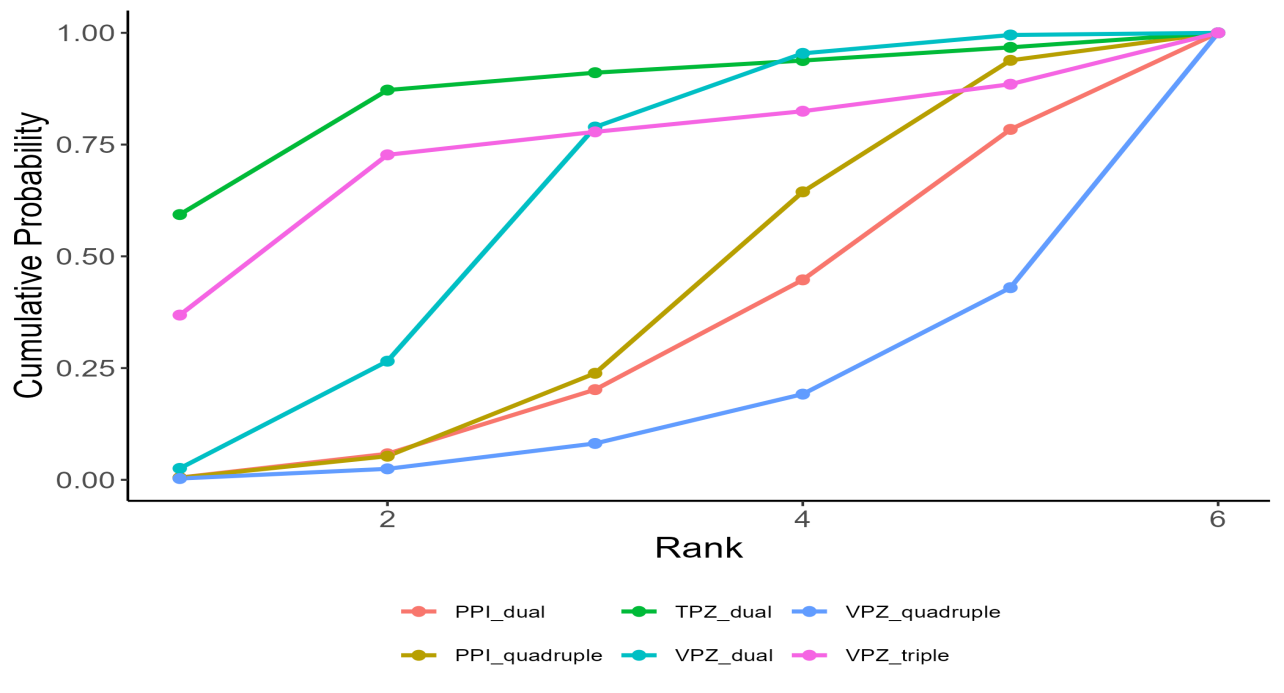


## Figure S 37 Ranking with SUCRA for eradication treatments regarding treatment discontinuation due to adverse events

## Table S 28 League matrix of treatment discontinuation rate due to adverse events in eradication treatments

| **TPZ_dual** | 4.32 (0.36, 174.58) | 6.49 (0.55, 261.9) | 1.79 (0.03, 163.14) | 9.02 (0.66, 371.79) | 5.77 (0.48, 223.15) |
| --- | --- | --- | --- | --- | --- |
| 0.23 (0.01, 2.77) | **VPZ_dual** | 1.52 (0.65, 3.27) | 0.41 (0.01, 6.4) | 2.05 (0.77, 5.31) | 1.34 (0.73, 2.31) |
| 0.15 (0, 1.8) | 0.66 (0.31, 1.54) | **PPI_dual** | 0.27 (0.01, 4.88) | 1.36 (0.41, 4.67) | 0.89 (0.36, 2.16) |
| 0.56 (0.01, 38.79) | 2.41 (0.16, 79.48) | 3.65 (0.2, 125.43) | **VPZ_triple** | 5 (0.27, 191.1) | 3.25 (0.19, 107.68) |
| 0.11 (0, 1.51) | 0.49 (0.19, 1.3) | 0.74 (0.21, 2.45) | 0.2 (0.01, 3.68) | **VPZ_quadruple** | 0.65 (0.25, 1.66) |
| 0.17 (0, 2.09) | 0.74 (0.43, 1.37) | 1.12 (0.46, 2.77) | 0.31 (0.01, 5.2) | 1.53 (0.6, 3.97) | **PPI_quadruple** |
| Odd ratio (95% confidence interval) reported. Statistically significant data presented in bold.  *VPZ_quadruple* Vonoprazan combined with two antibiotics and Bismuth, *VPZ_triple* Vonoprazan combined with two antibiotics, *VPZ_dual* Vonoprazan combined with one antibiotic, *PPI_quadruple* Proton pump inhibitor combined with two antibiotics and Bismuth, *PPI_dual* Proton pump inhibitor combined with one antibiotic; *TPZ_dual* Tegoprazan combined with one antibiotic | | | | | |

# Appendix 19 Treatment discontinuation due to adverse events of various regimens -Fix


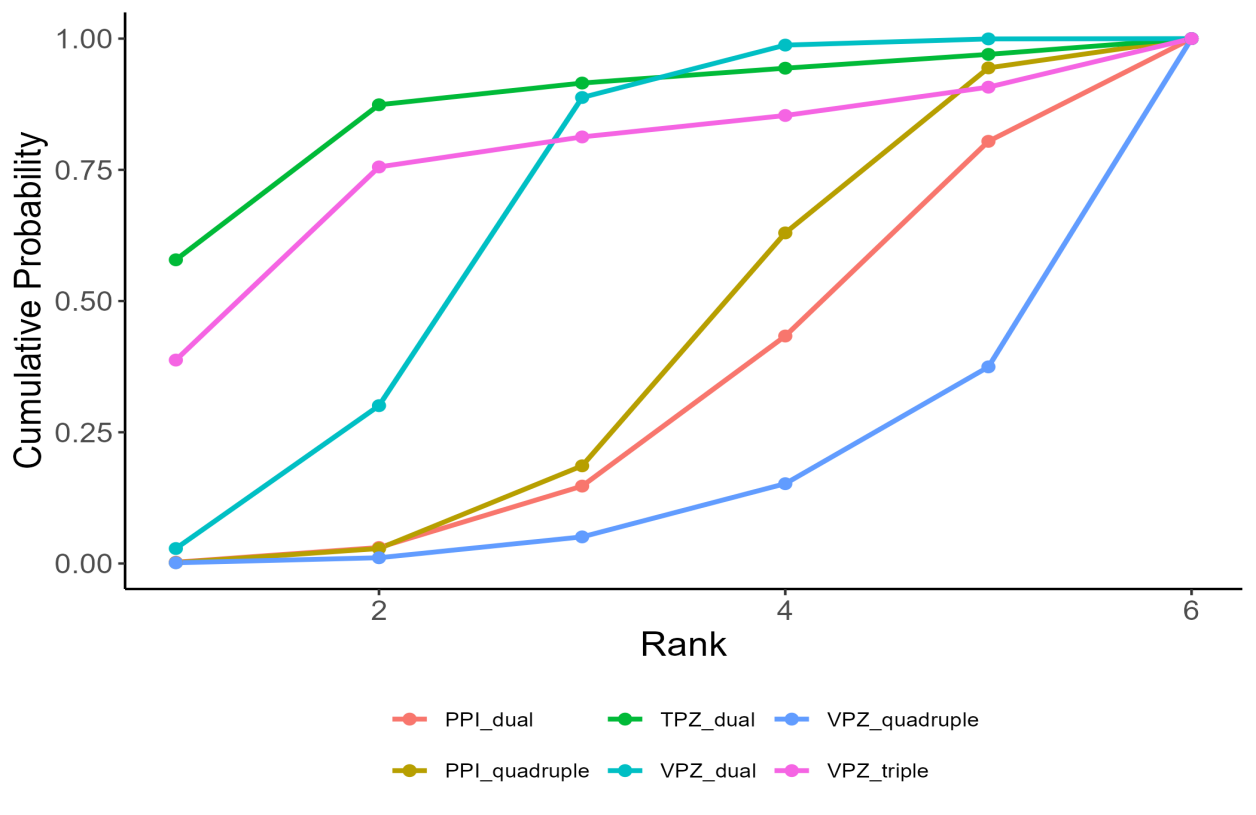


## Figure S 38 Ranking with SUCRA for eradication treatments regarding treatment discontinuation due to adverse events

## Table S 29 League matrix of treatment discontinuation rate due to adverse events in eradication treatments

| **TPZ_dual** | 3.96 (0.39, 121.58) | 6.17 (0.61, 188.26) | 1.64 (0.03, 114.43) | 8.58 (0.74, 285.04) | 5.54 (0.56, 165.27) |
| --- | --- | --- | --- | --- | --- |
| 0.25 (0.01, 2.58) | **VPZ_dual** | 1.56 (0.84, 2.82) | 0.41 (0.01, 5.28) | 2.13 (0.98, 4.94) | 1.4 (0.9, 2.19) |
| 0.16 (0.01, 1.65) | 0.64 (0.36, 1.19) | **PPI_dual** | 0.26 (0.01, 3.65) | 1.38 (0.53, 3.75) | 0.9 (0.47, 1.76) |
| 0.61 (0.01, 36.39) | 2.43 (0.19, 78.1) | 3.81 (0.27, 127.23) | **VPZ_triple** | 5.29 (0.36, 177.7) | 3.4 (0.25, 111.83) |
| 0.12 (0, 1.35) | 0.47 (0.2, 1.02) | 0.73 (0.27, 1.88) | 0.19 (0.01, 2.8) | **VPZ_quadruple** | 0.66 (0.29, 1.43) |
| 0.18 (0.01, 1.8) | 0.71 (0.46, 1.12) | 1.11 (0.57, 2.15) | 0.29 (0.01, 3.96) | 1.52 (0.7, 3.48) | **PPI_quadruple** |
| Odd ratio (95% confidence interval) reported. Statistically significant data presented in bold.  *VPZ_quadruple* Vonoprazan combined with two antibiotics and Bismuth, *VPZ_triple* Vonoprazan combined with two antibiotics, *VPZ_dual* Vonoprazan combined with one antibiotic, *PPI_quadruple* Proton pump inhibitor combined with two antibiotics and Bismuth, *PPI_dual* Proton pump inhibitor combined with one antibiotic; *TPZ_dual* Tegoprazan combined with one antibiotic | | | | | |

# Appendix 20 Funnel plots-Random


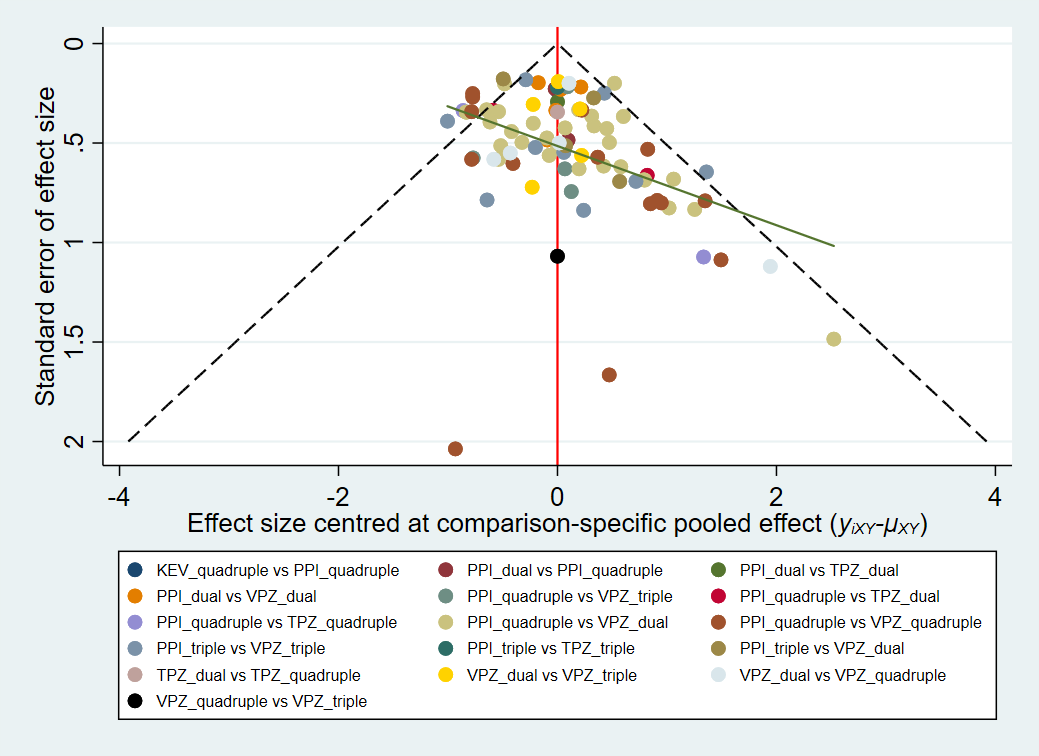


## Figure S 39 Funnel plot for eradication treatment in the overall analysis


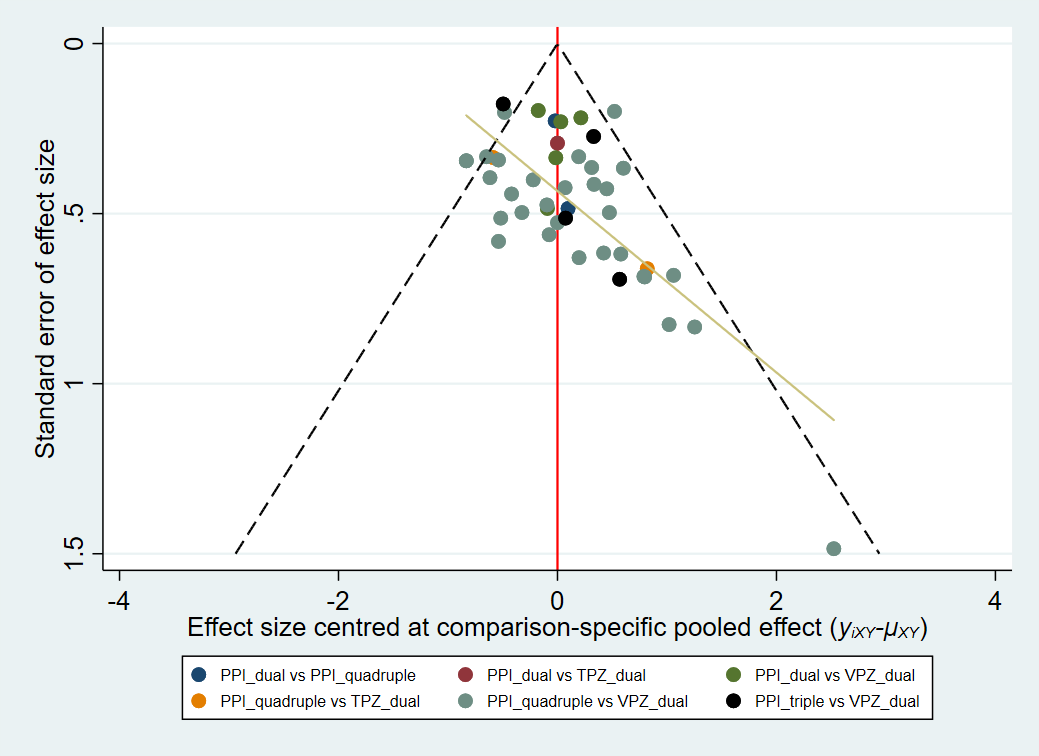


## Figure S 40 Funnel plot for PCAB-based dual therapy for eradication treatment


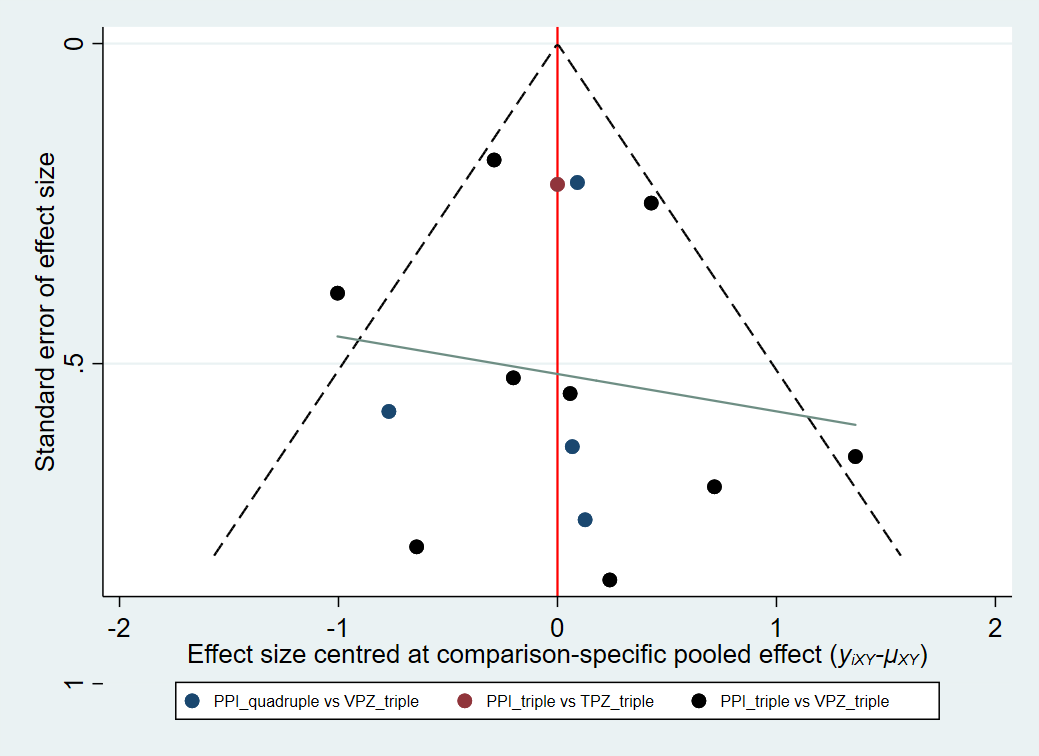


## Figure S 41 Funnel plot for PCAB-based triple therapy for eradication treatment


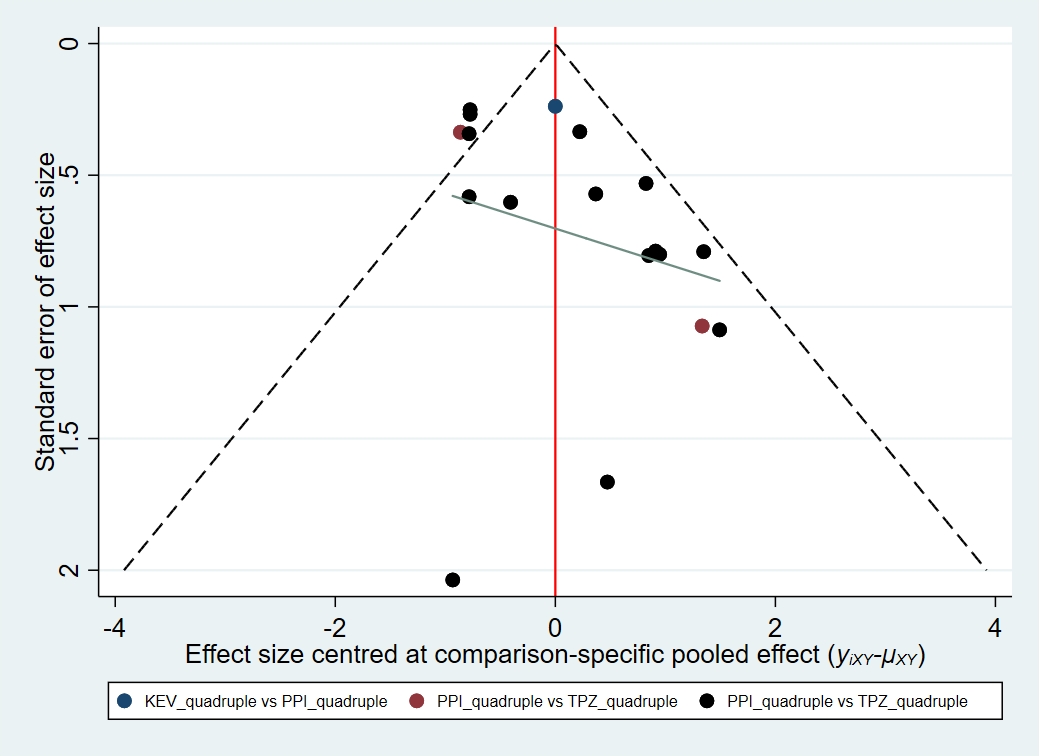


## Figure S 42 Funnel plot for PCAB-based quadruple therapy for eradication treatment


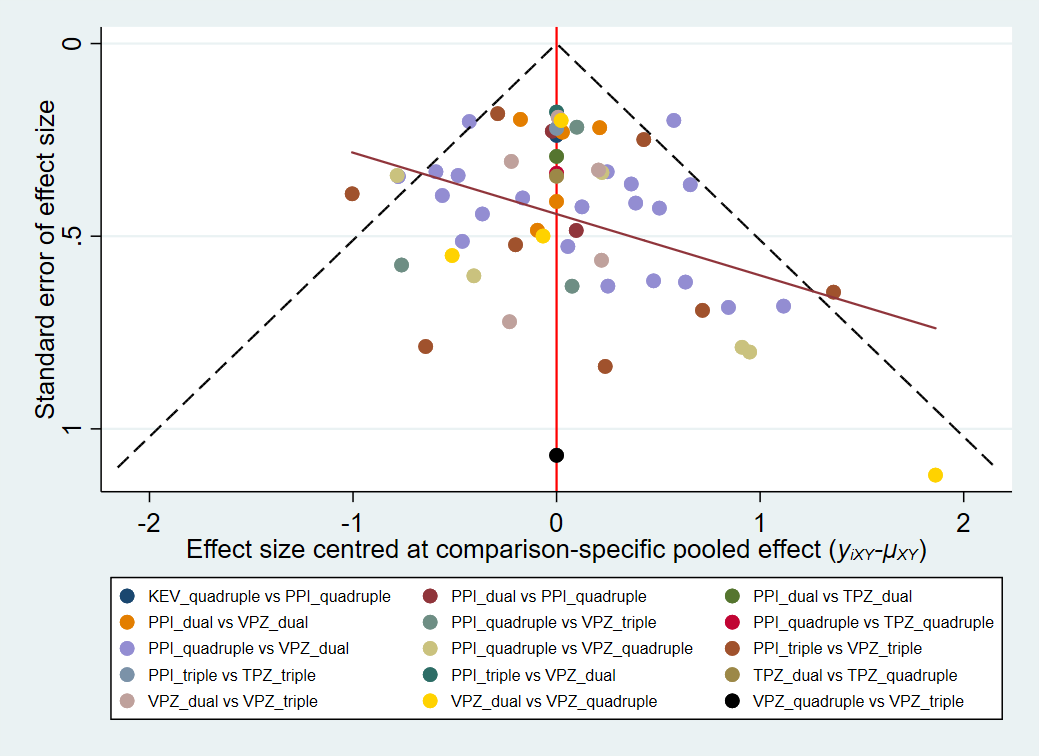


## Figure S 43 Funnel plot for eradication treatment in treatment-naïve patients


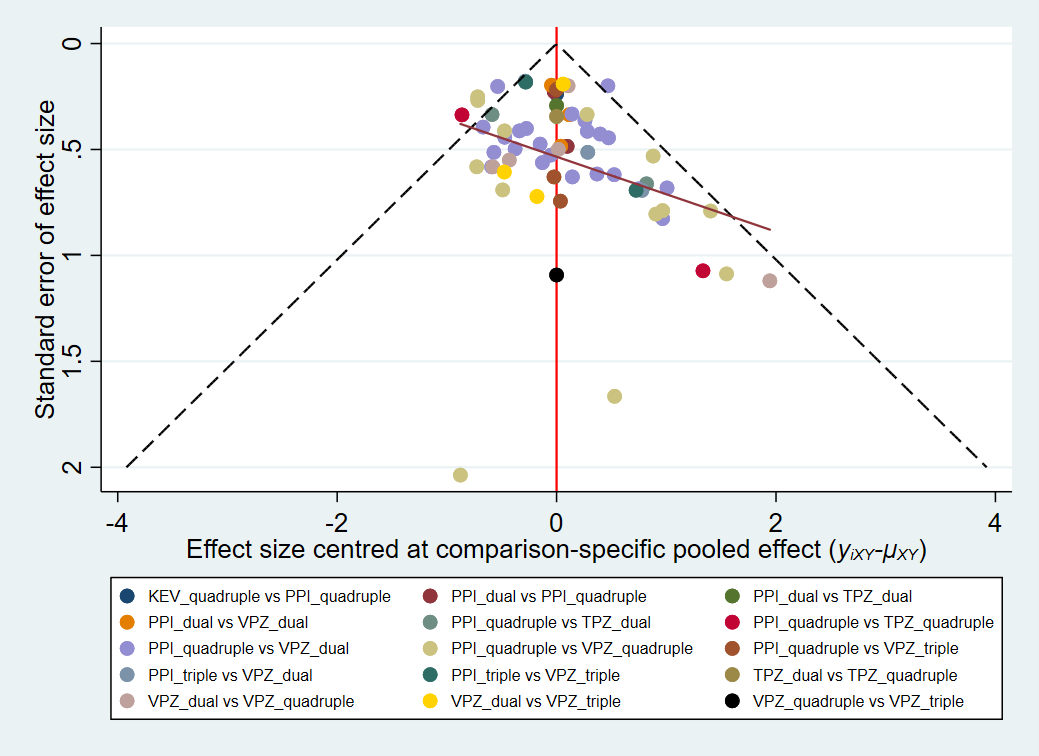


## Figure S 44 Funnel plot for 14-day eradication treatment


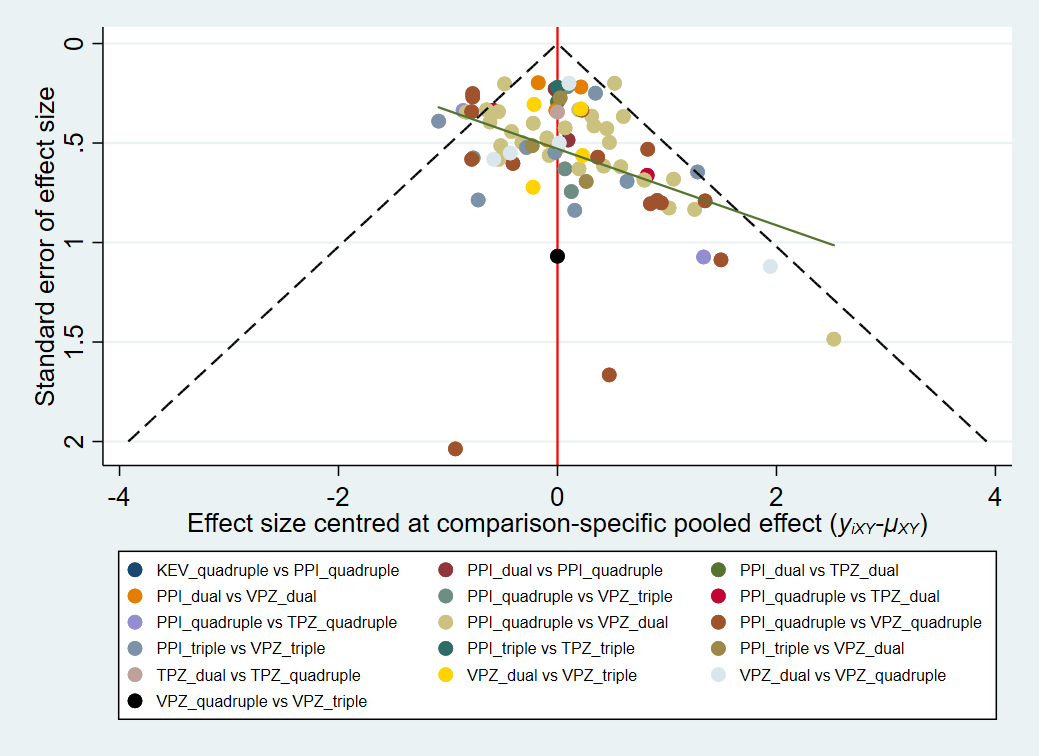


## Figure S 45 Funnel plot for eradication treatment in Asian patients


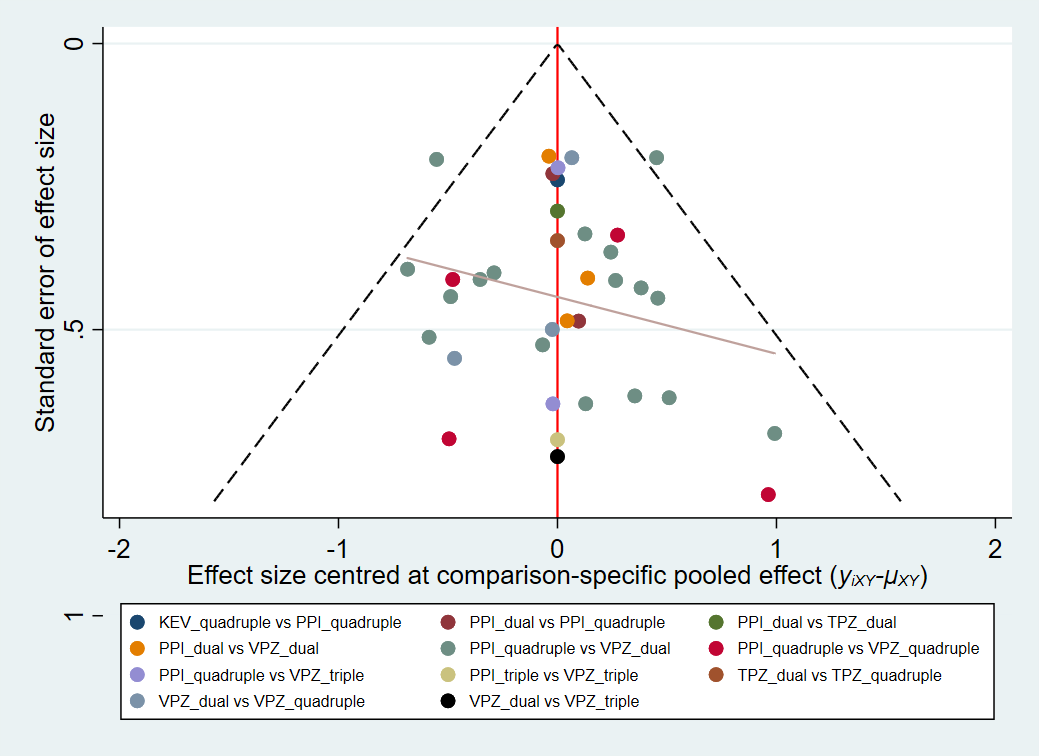


## Figure S 46 Funnel plot for 14-day eradication treatment in treatment-naïve patients in China


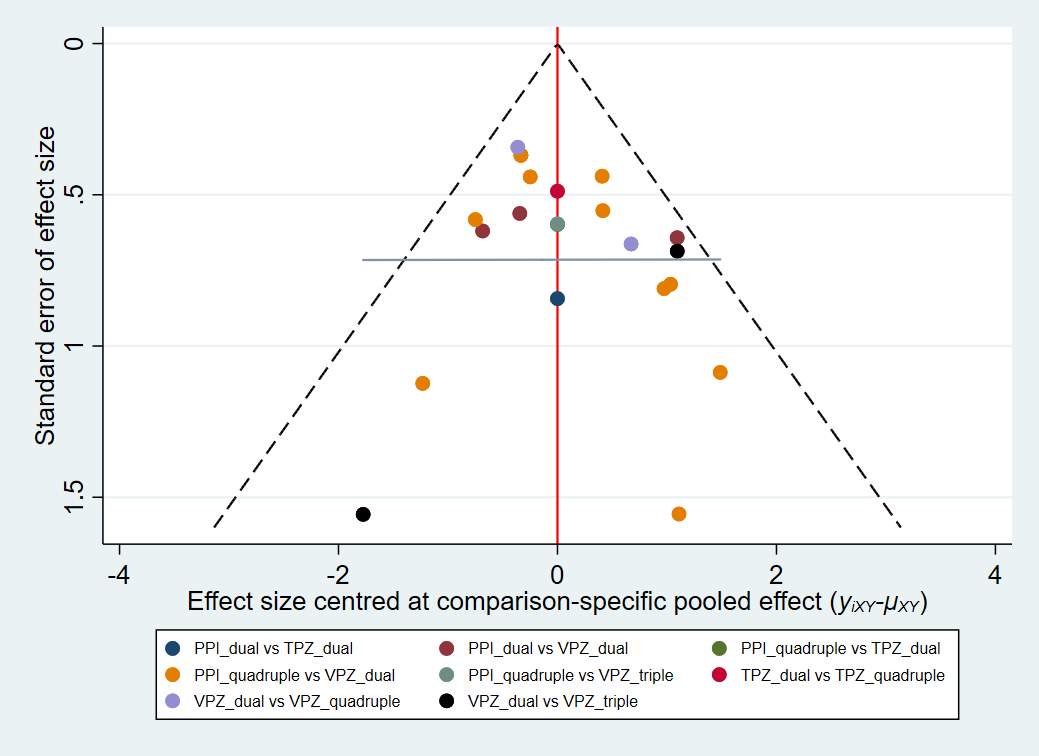


## Figure S 47 Funnel plot for treatments based on compliance


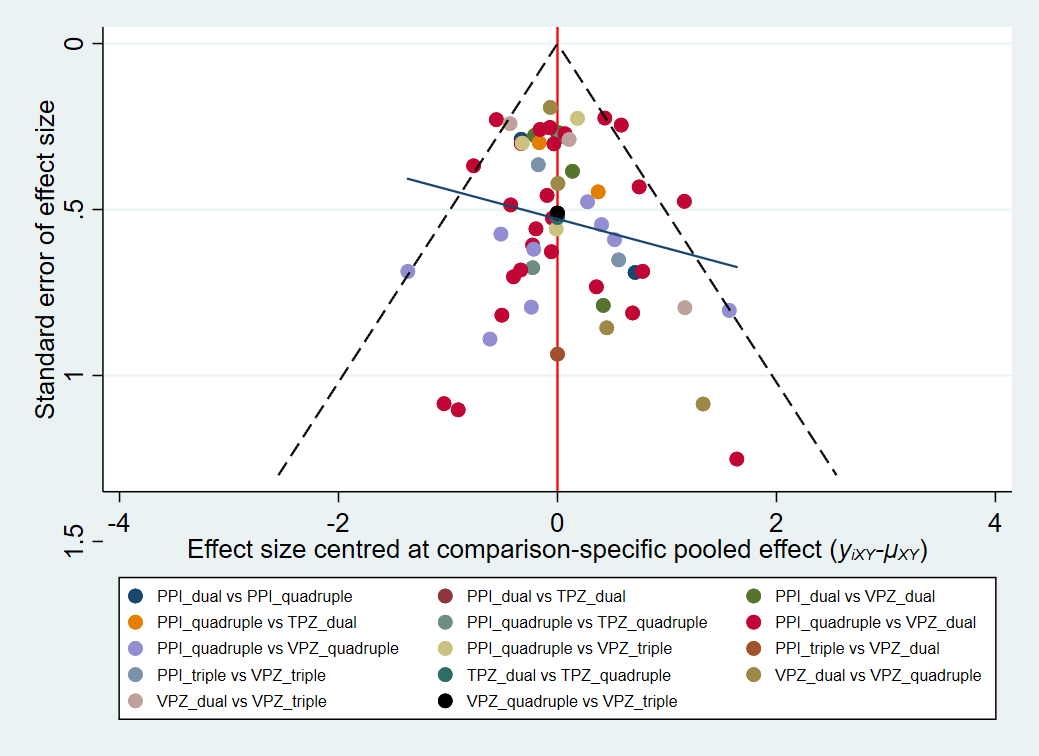


## Figure S 48 Funnel plot for treatment based on adverse events


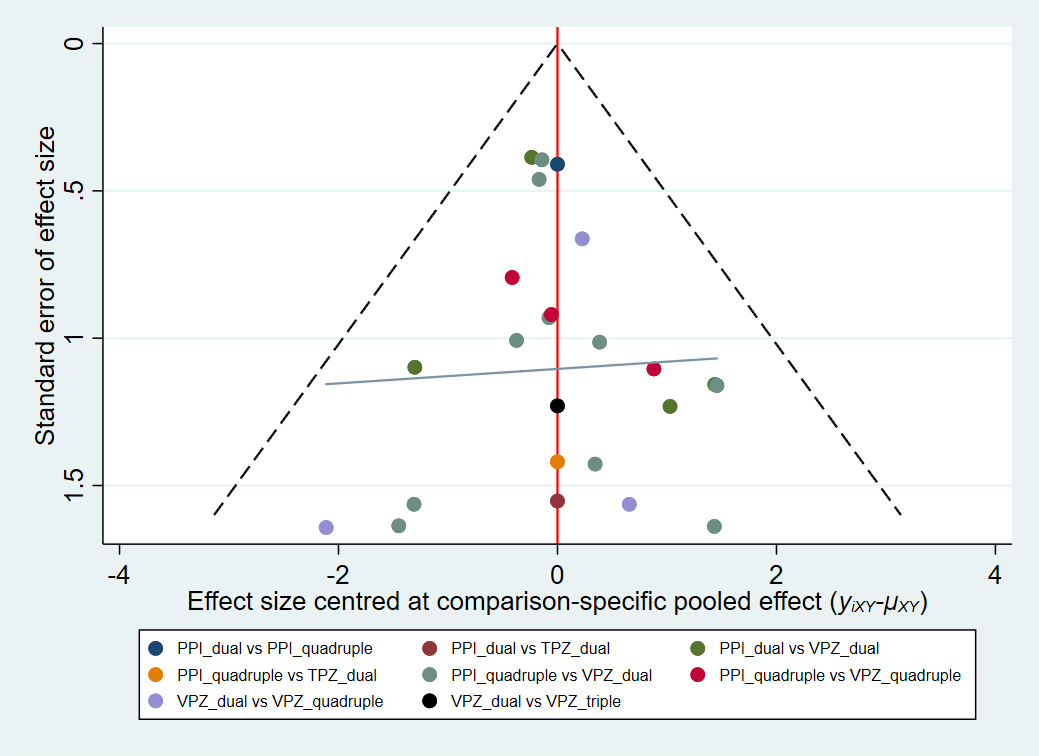


## Figure S 49 Funnel plot for treatment based on treatment discontinuation due to adverse events

# Appendix 21 Inconsistency (Node-splitting) results-Random


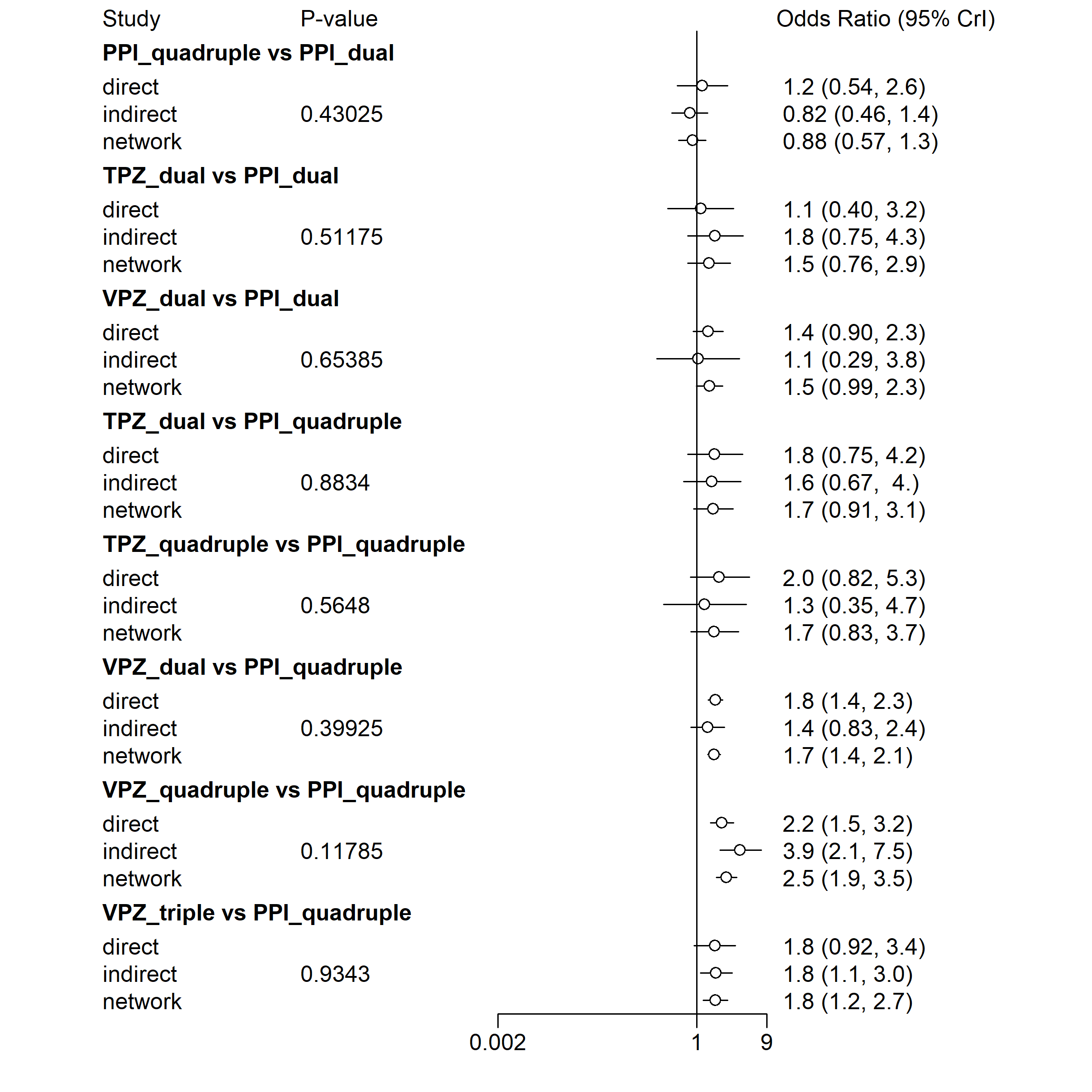


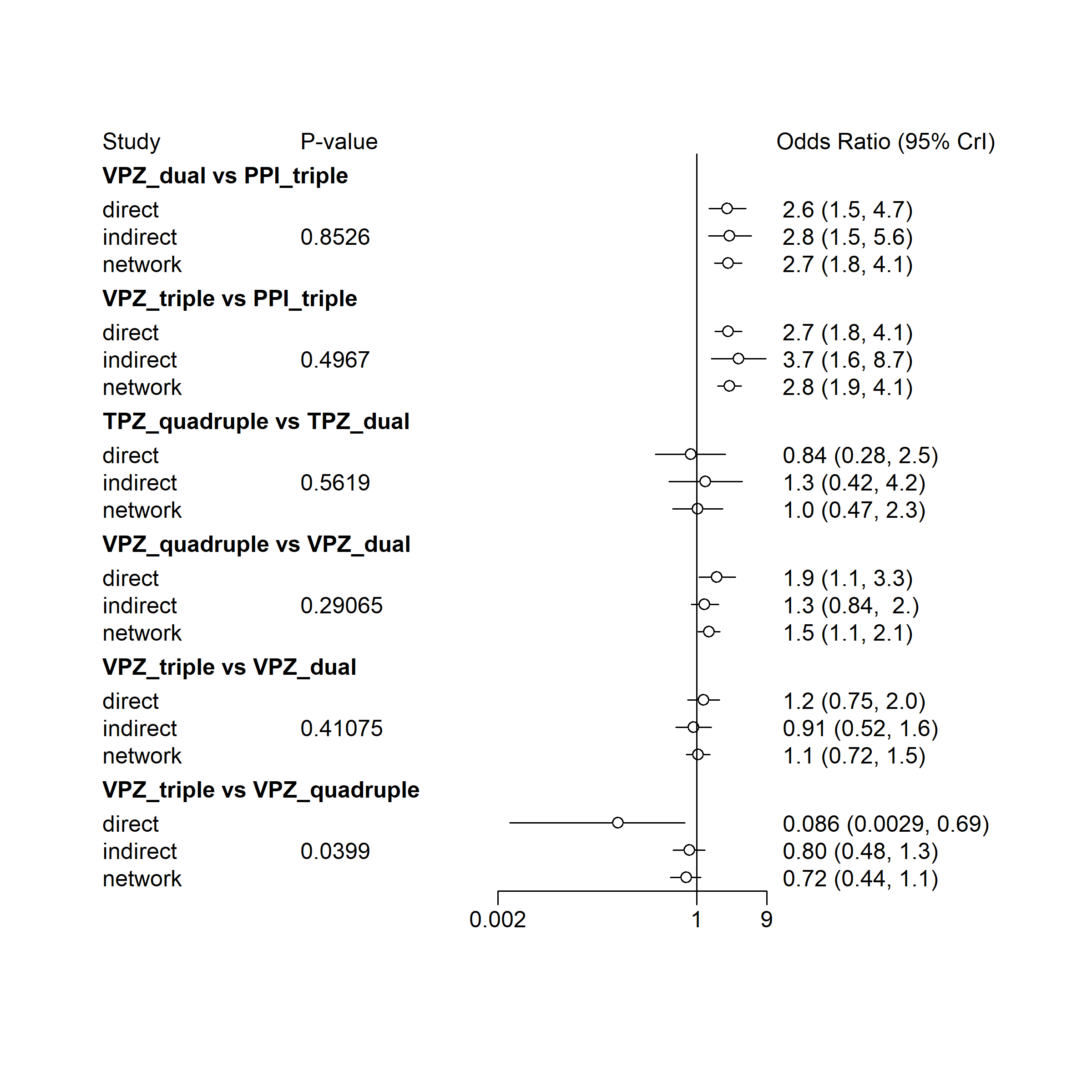


## Figure S 50 Detection of local inconsistencies in eradication rates


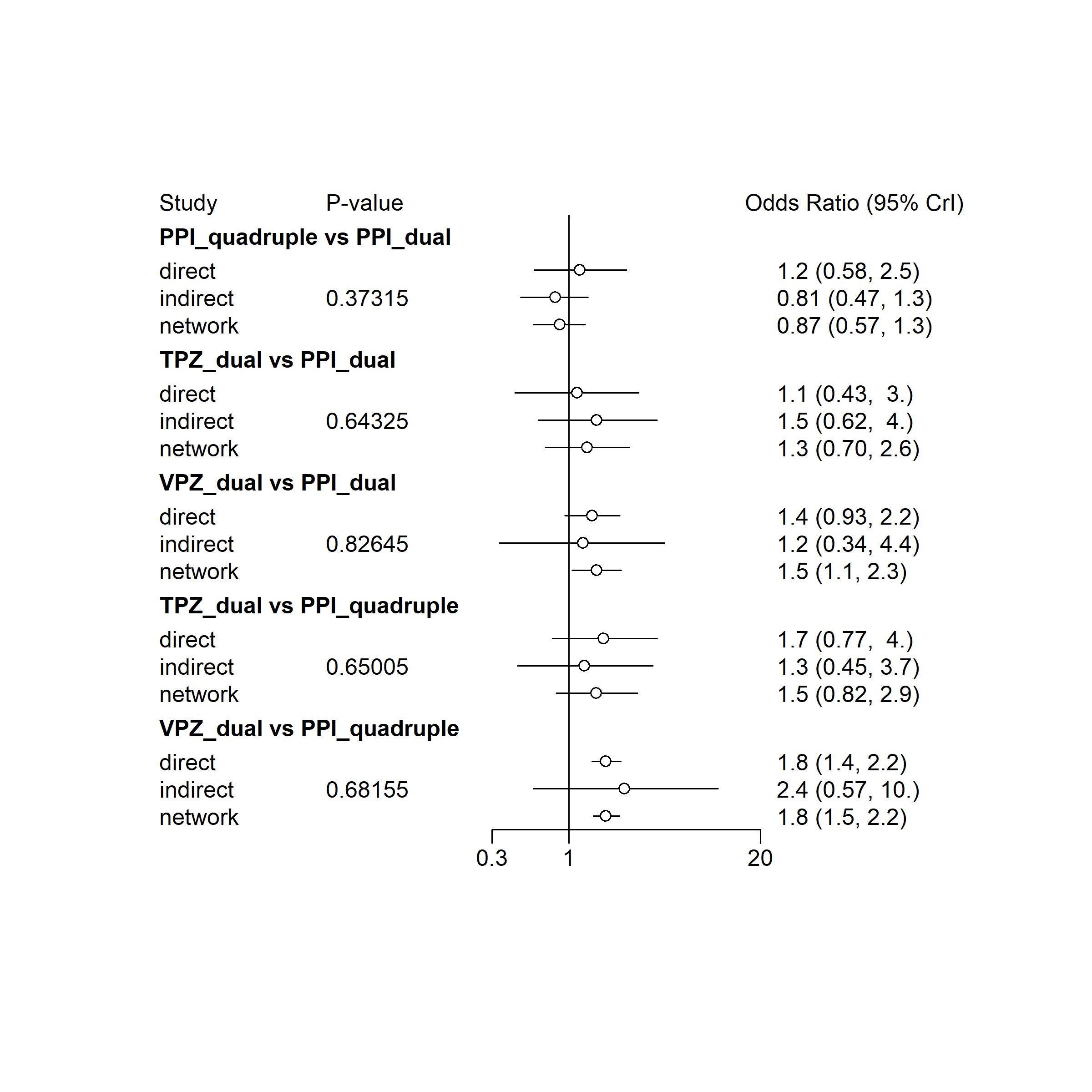


## Figure S 51 Detection of local inconsistencies in PCAB-based dual therapy treatment groups on eradication rate


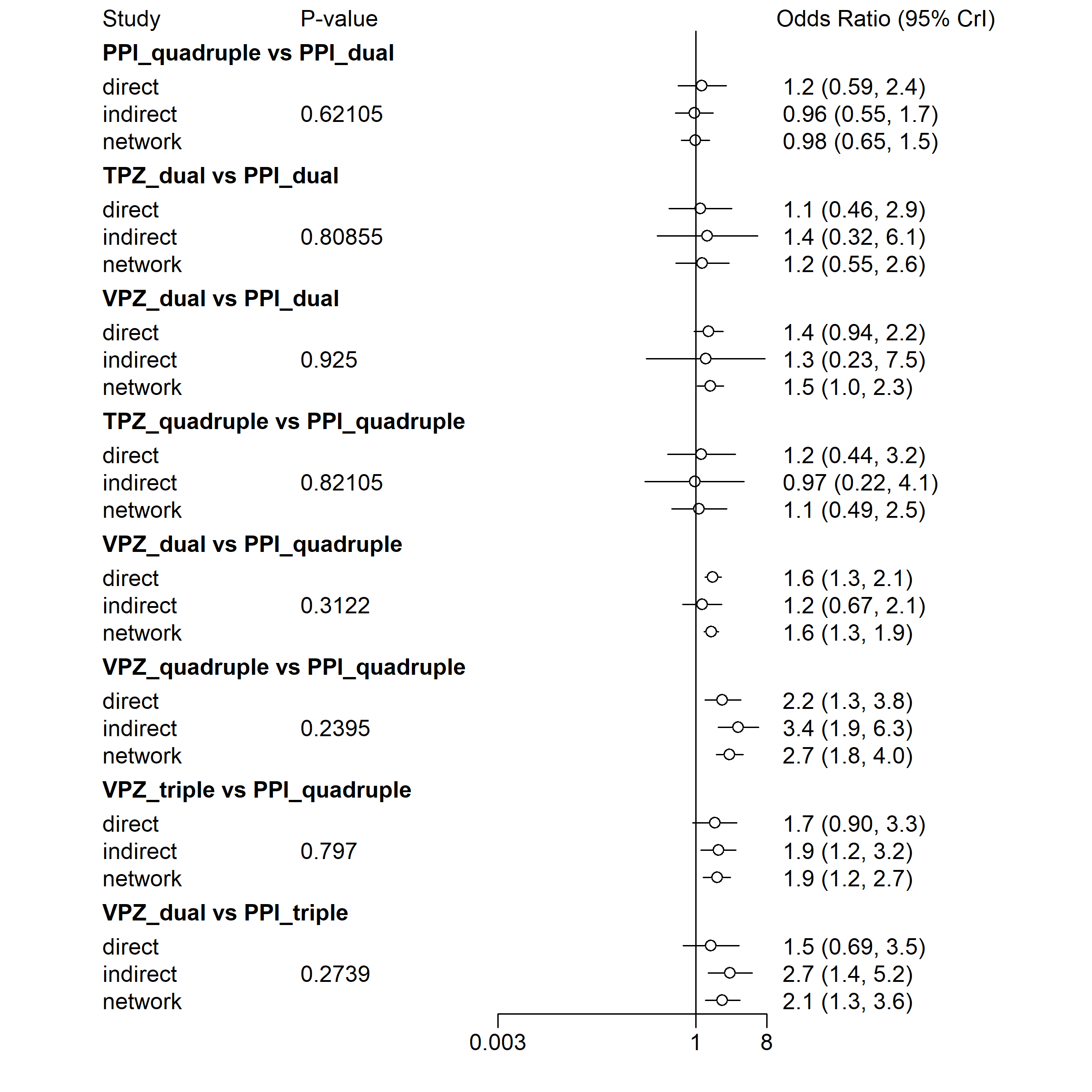


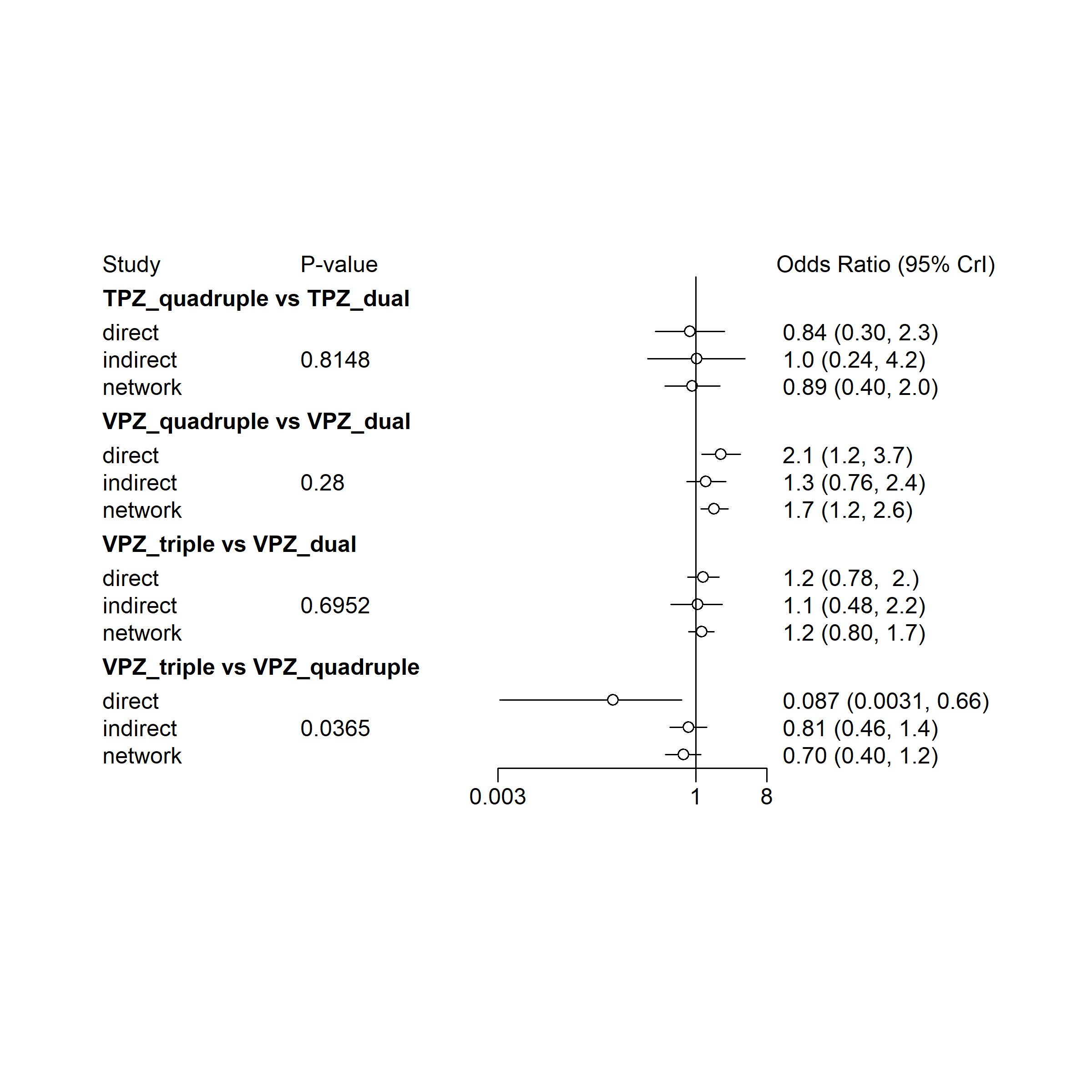


## Figure S 52 Detection of local inconsistencies of eradication treatments in treatment-naive patients


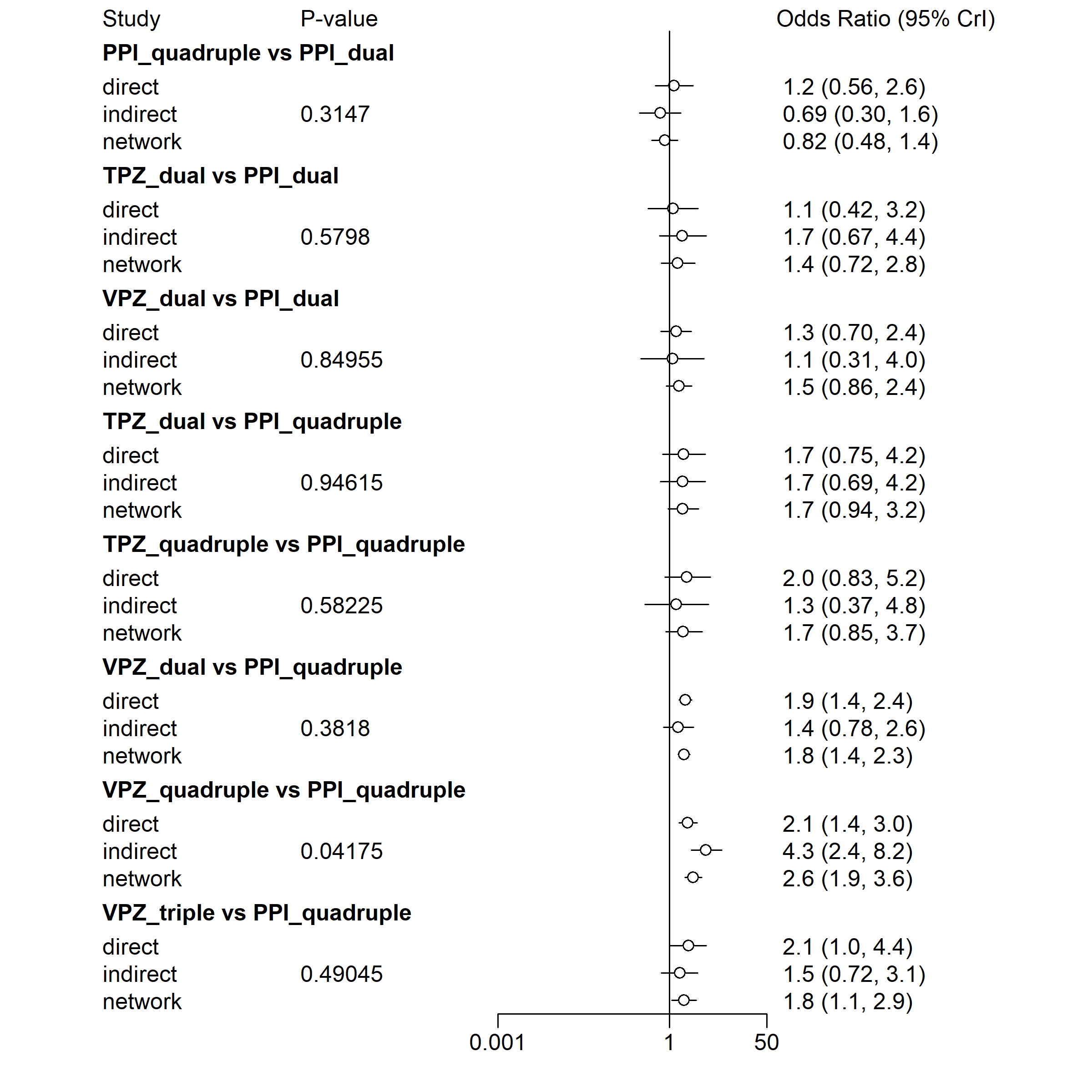


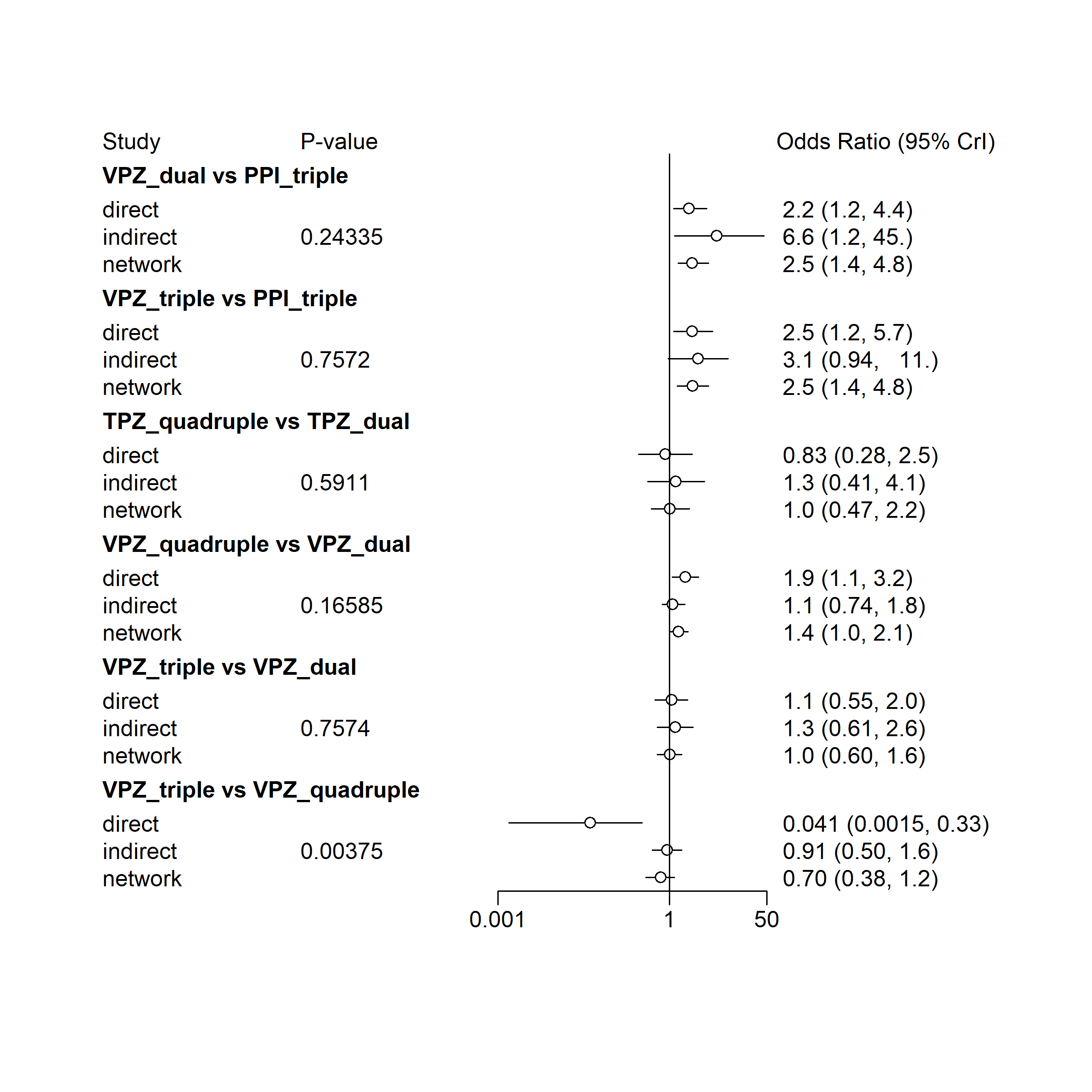


## Figure S 53 Detection of local inconsistencies of 14-day treatment groups on eradication rate


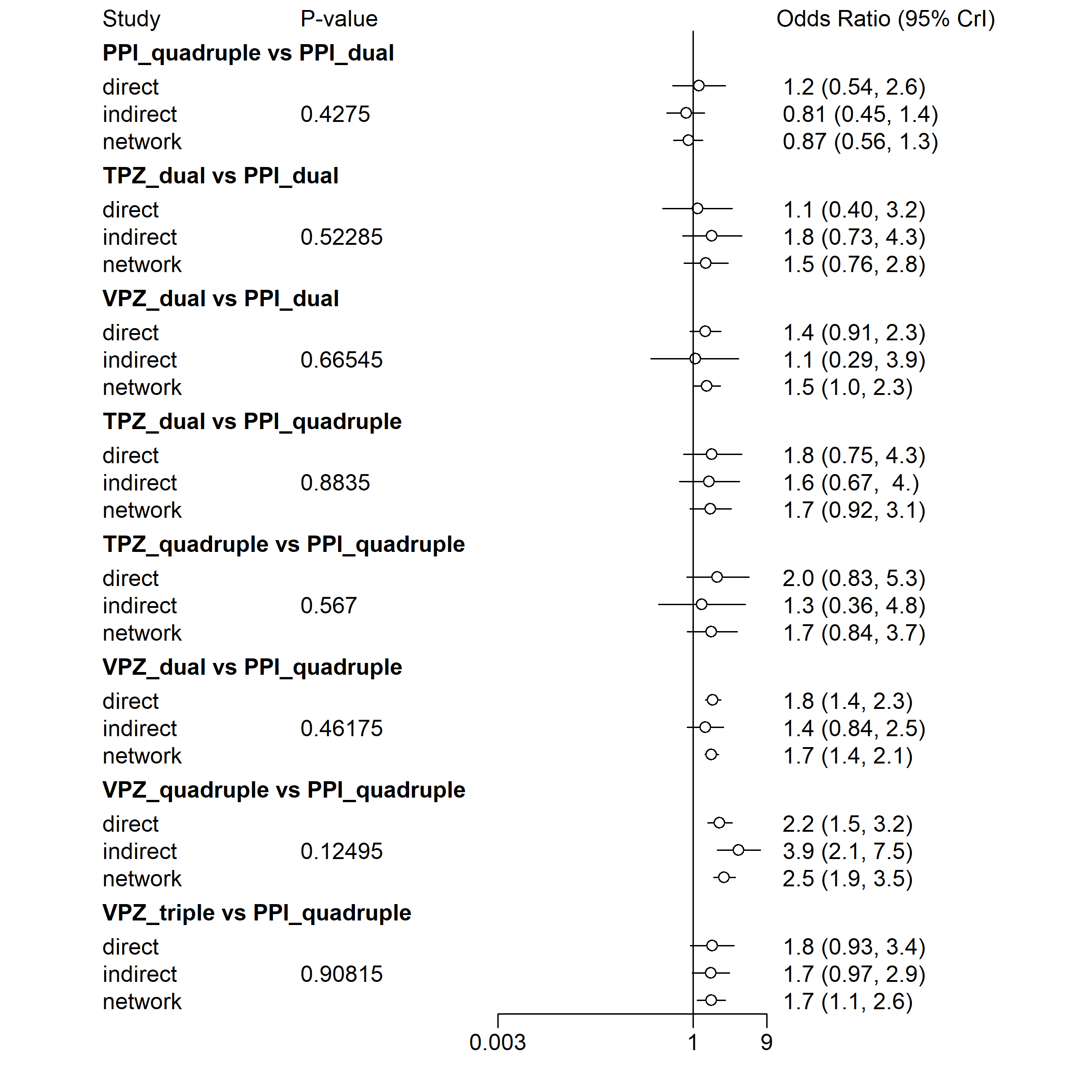


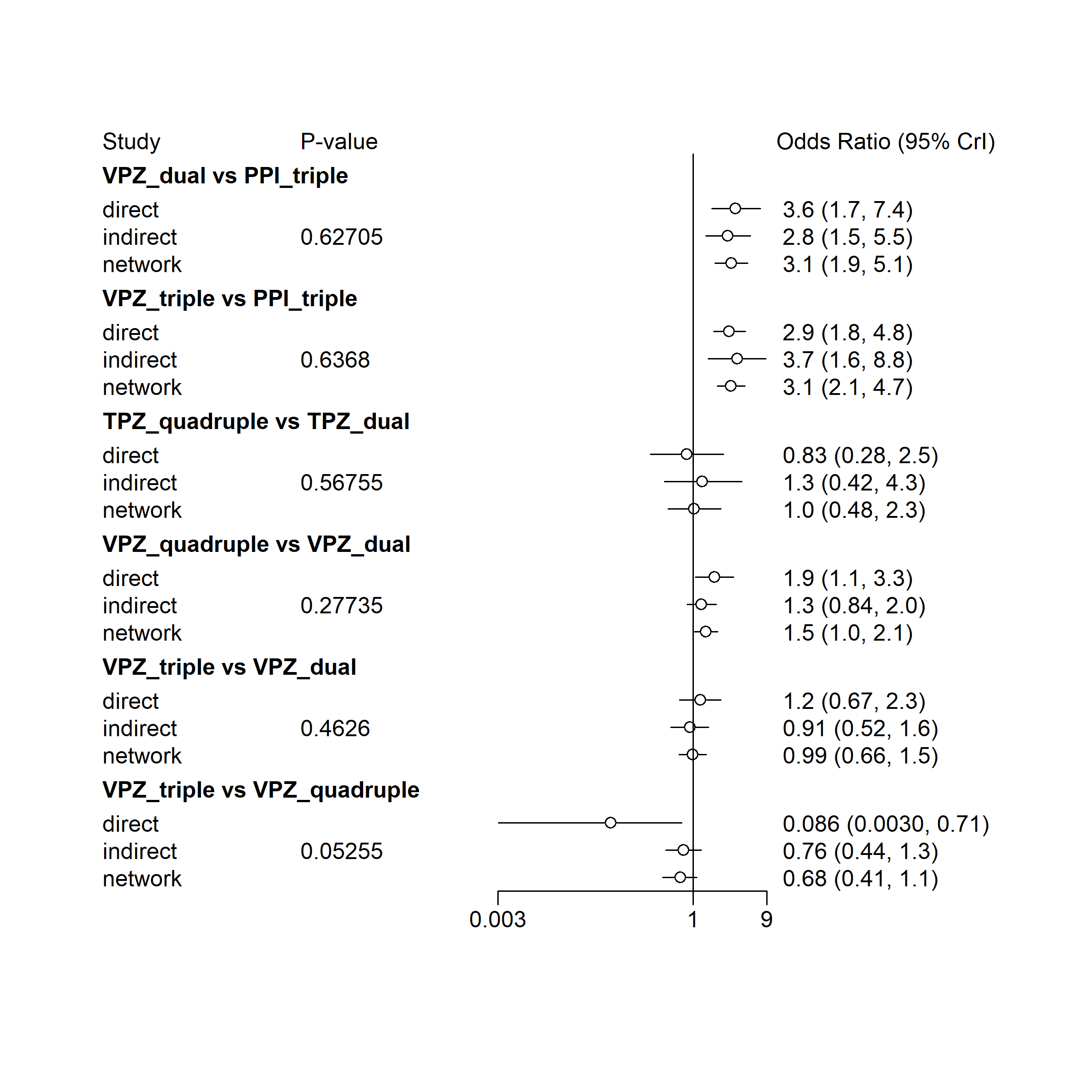


## Figure S 54 Detection of local inconsistencies for eradication treatment in Asian patients


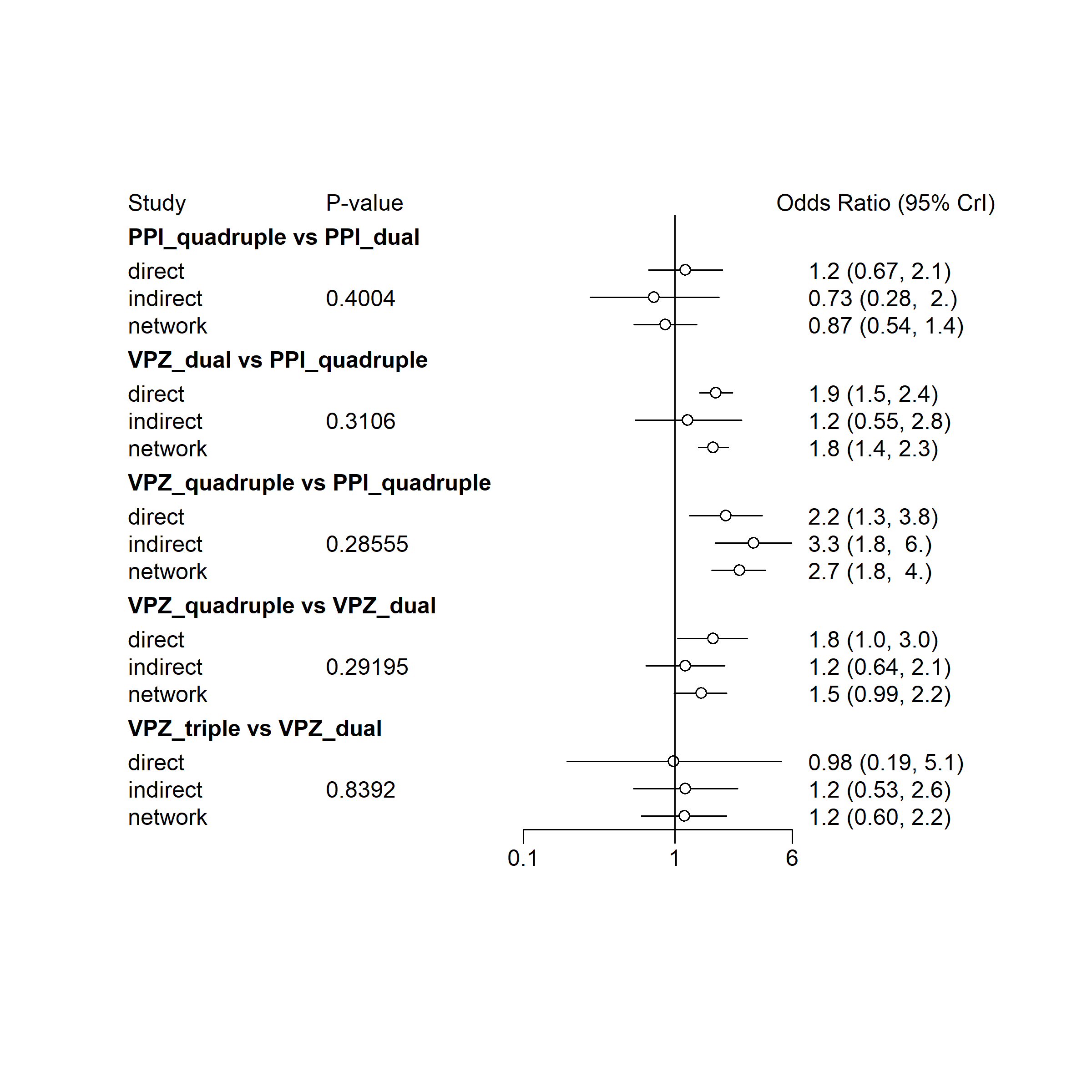


## Figure S 55 Detection of local inconsistencies of 14-day eradication treatment in treatment-naive patients in China


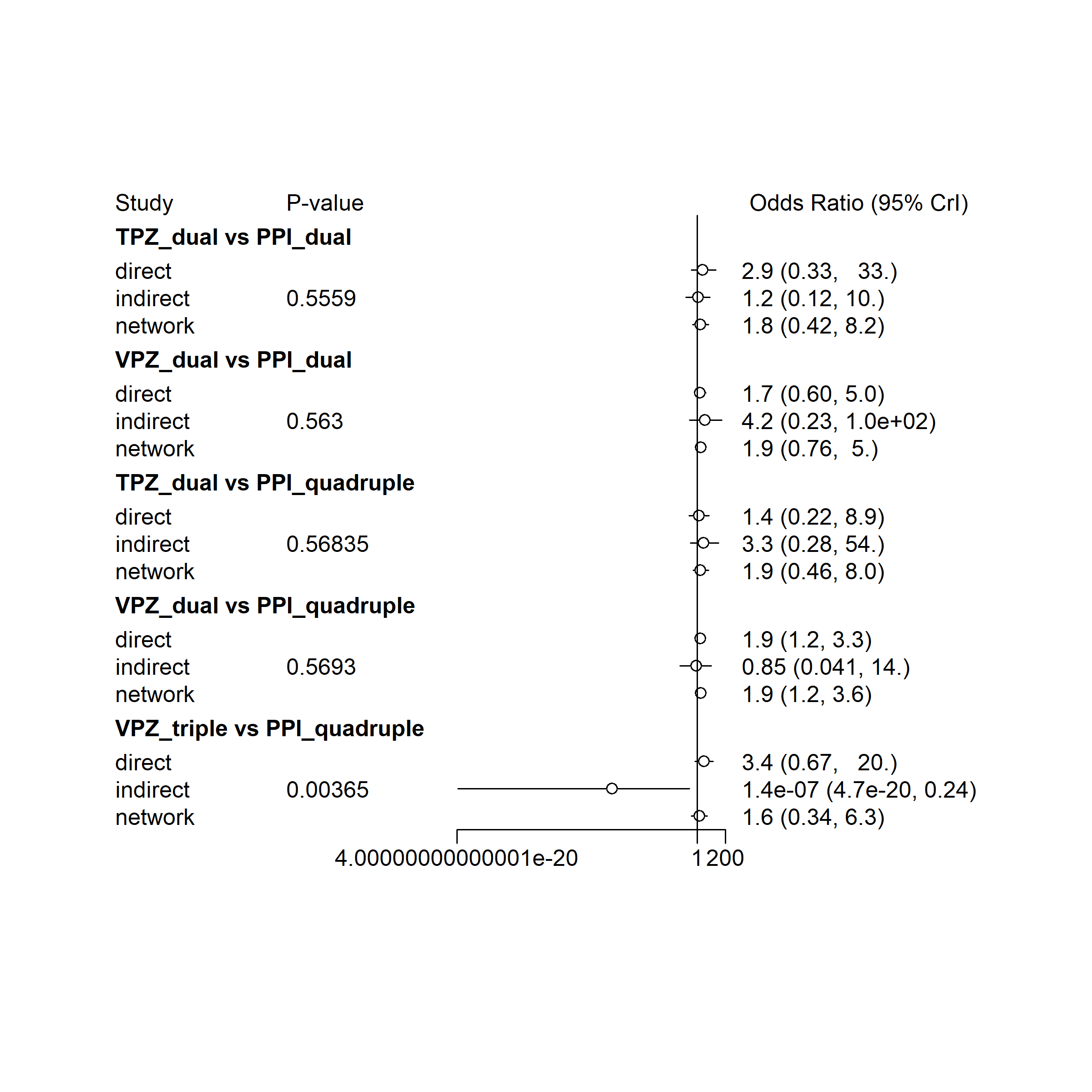


## Figure S 56 Detection of local inconsistencies of treatment groups on compliance


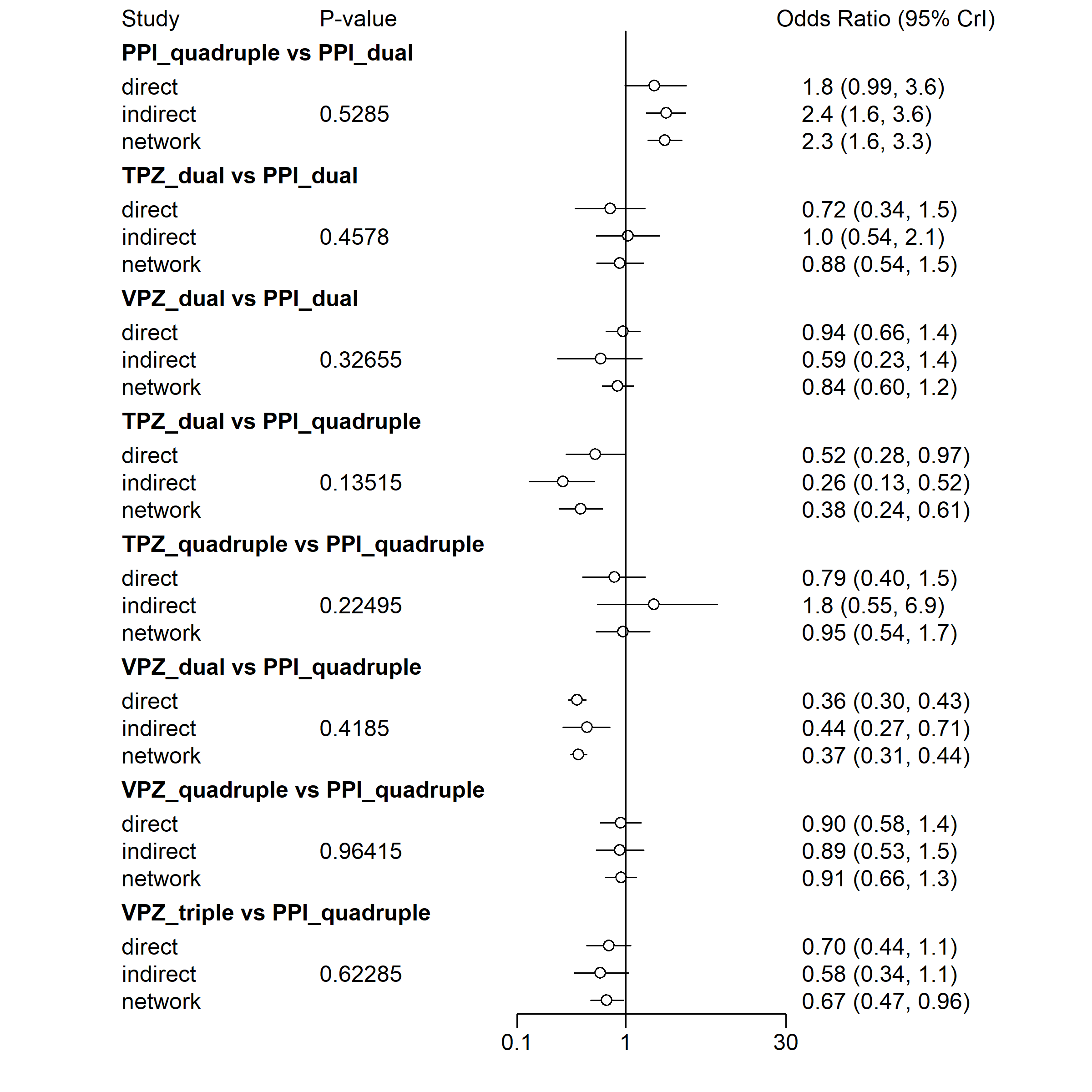


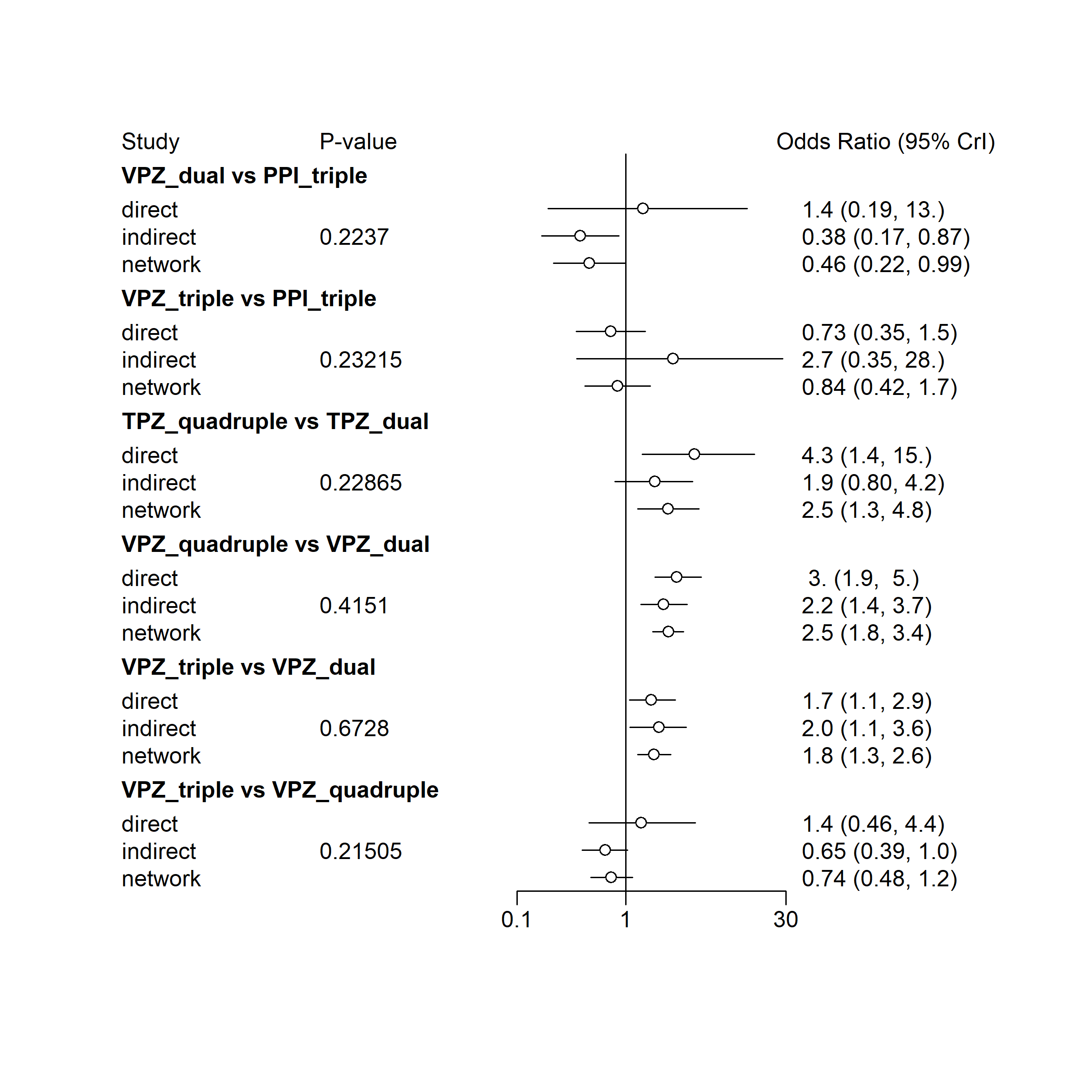


## Figure S 57 Detection of local inconsistencies of treatment groups on adverse events


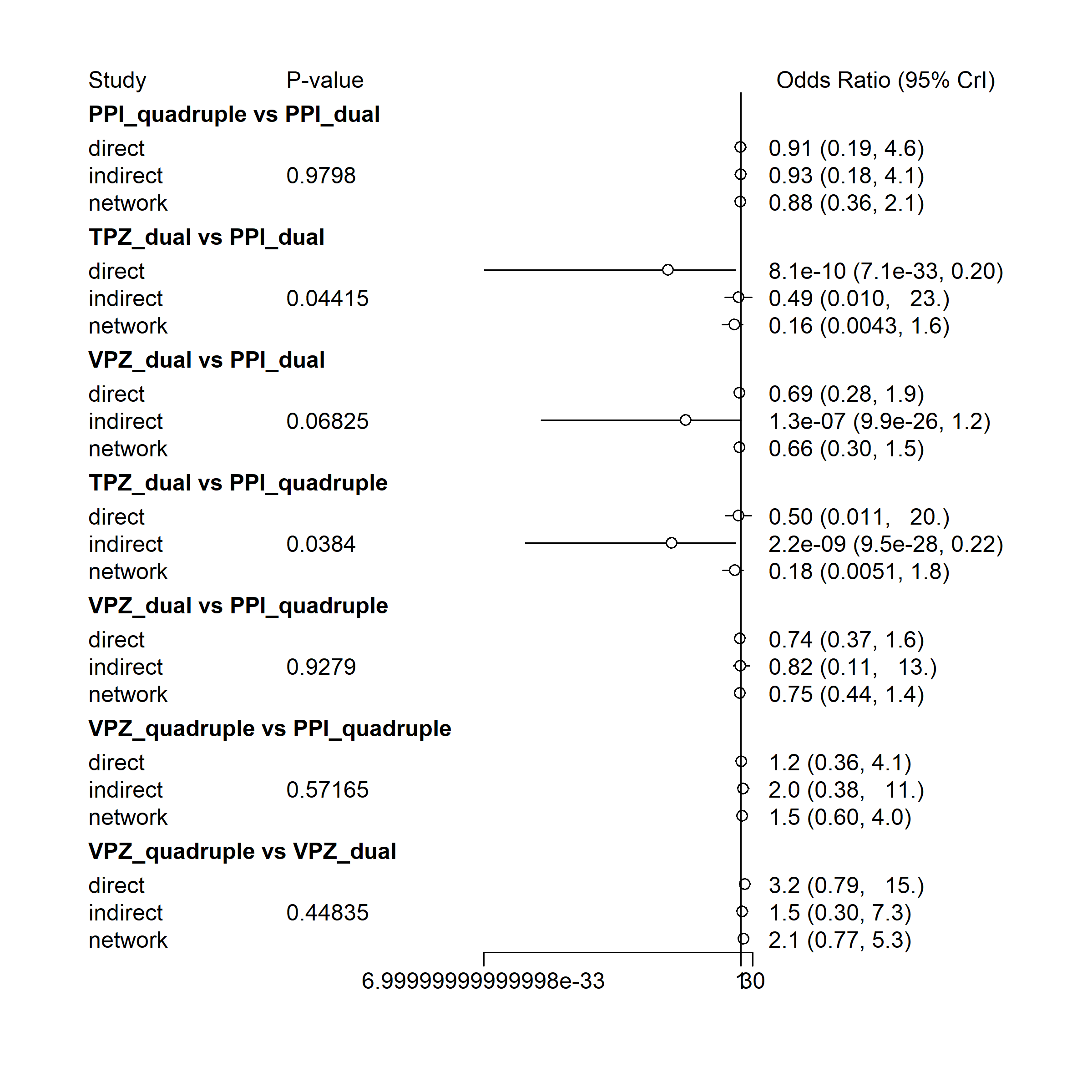


## Figure S 58 Detection of local inconsistencies of treatment groups on treatment discontinuation due to adverse events

Appendix 22 Heterogeneity test-Random


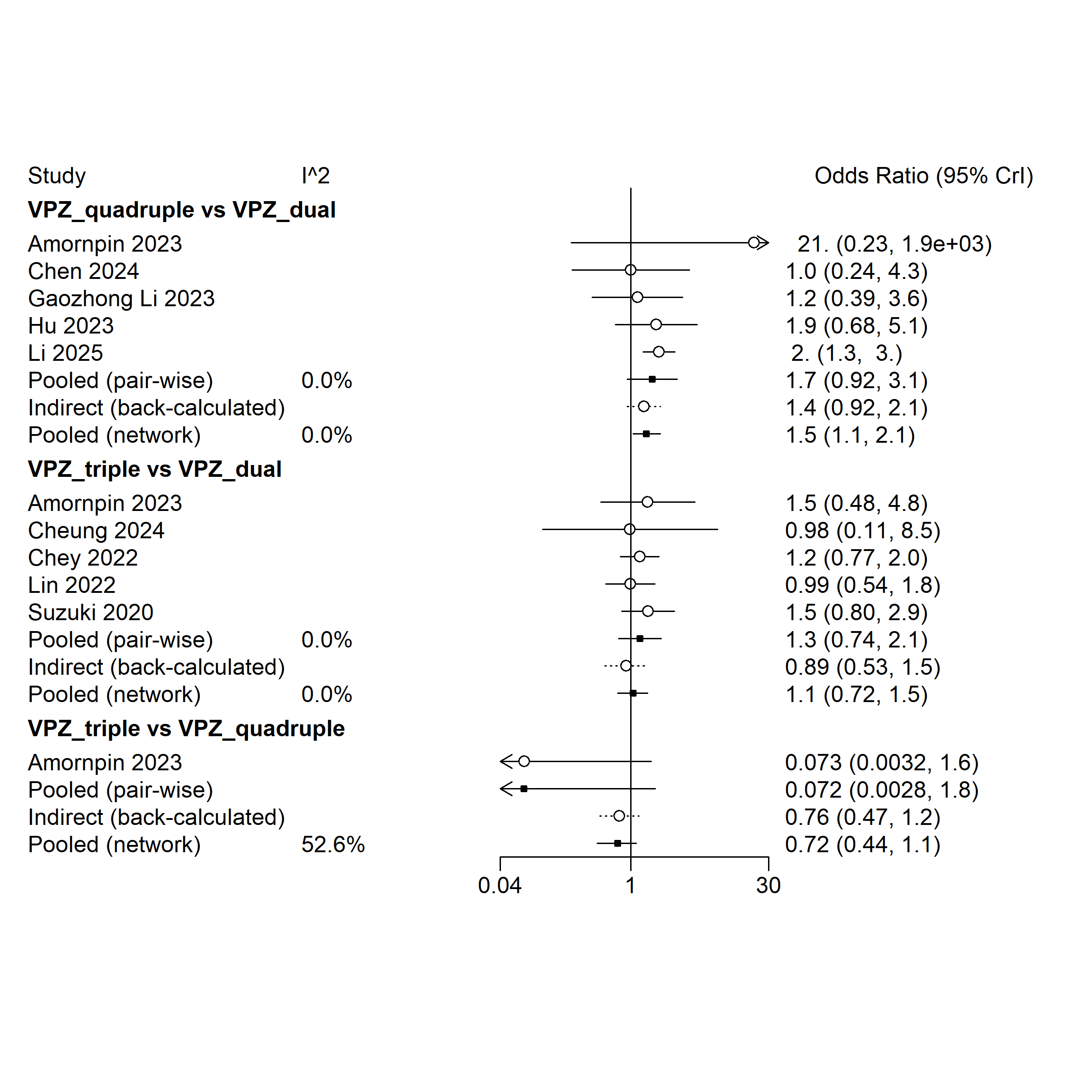

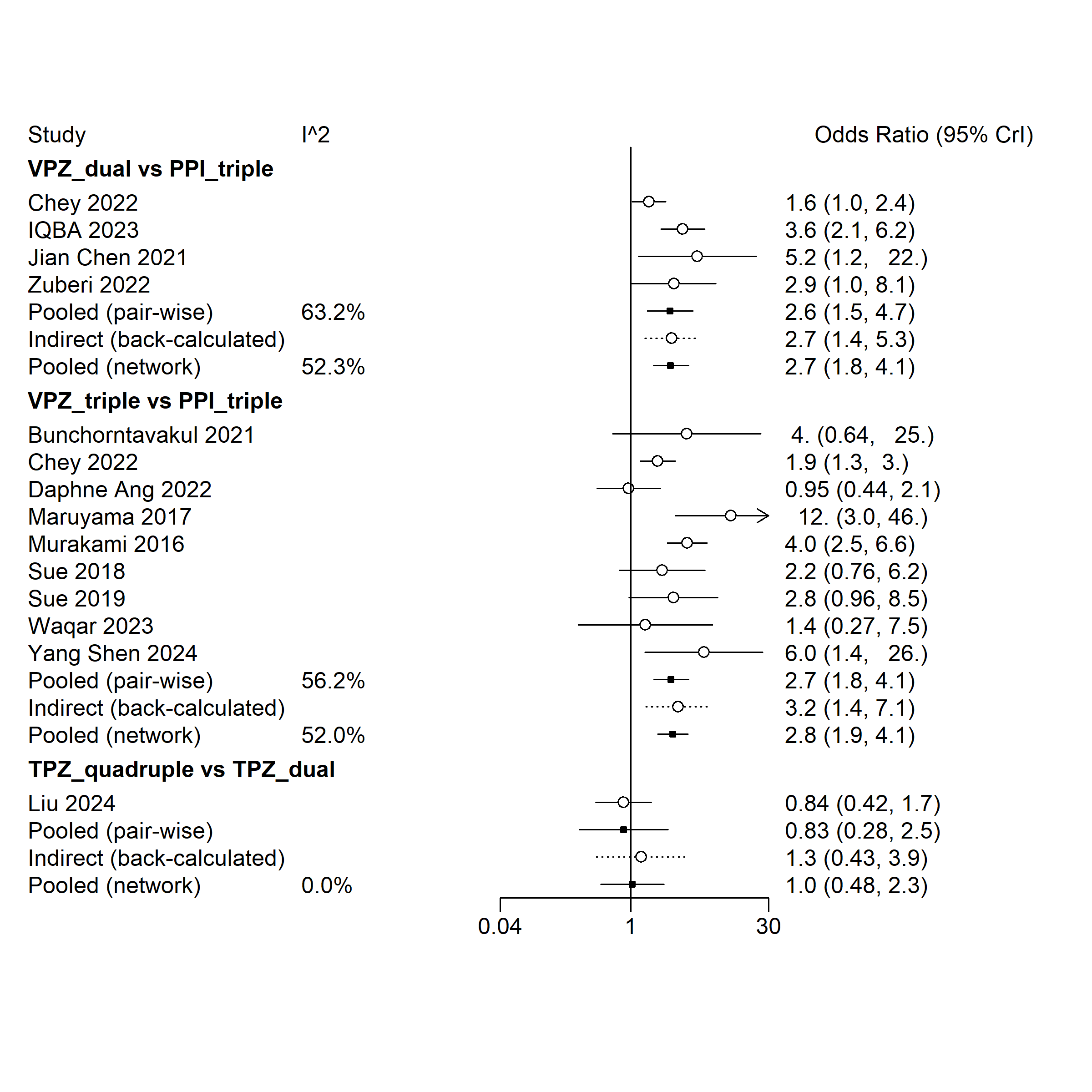

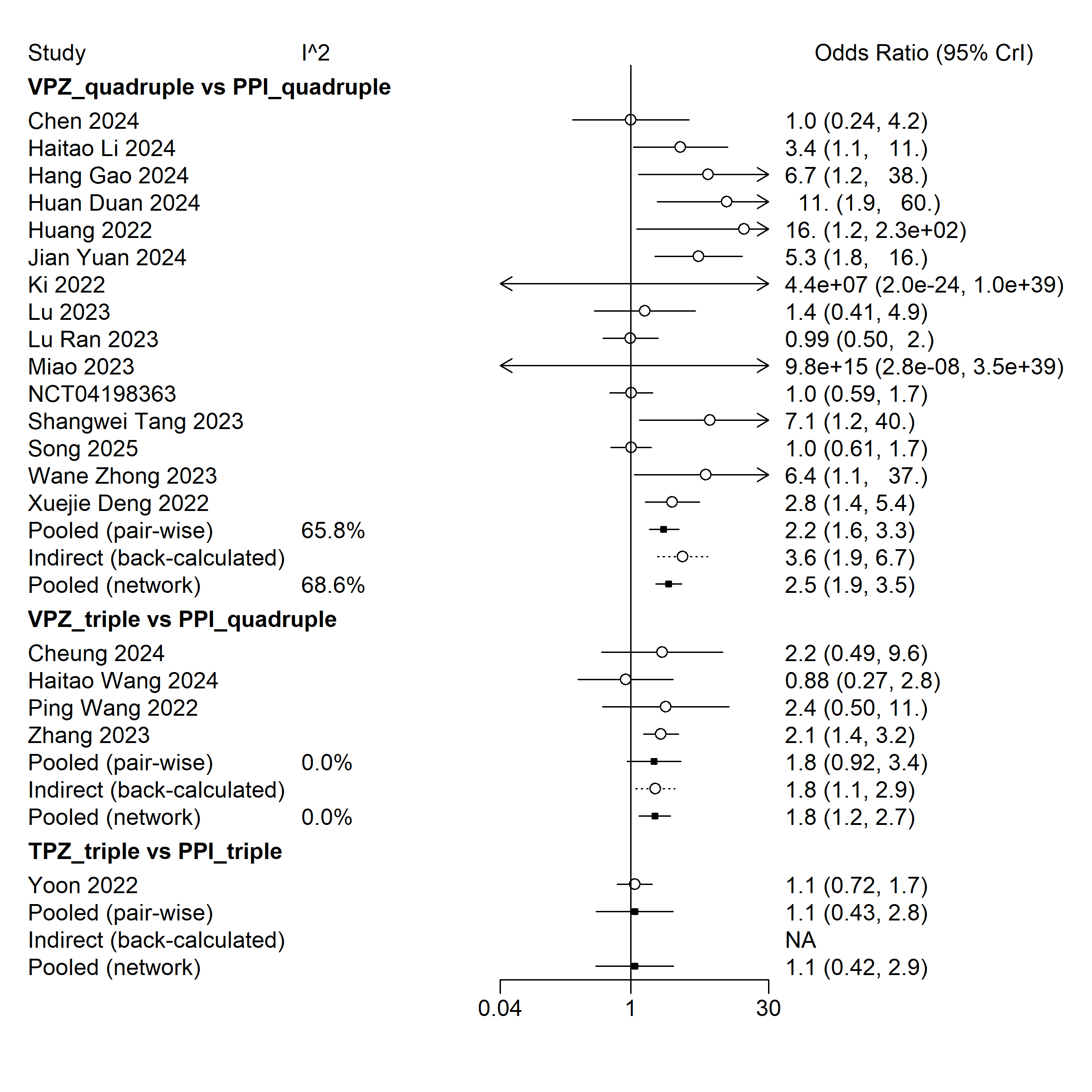

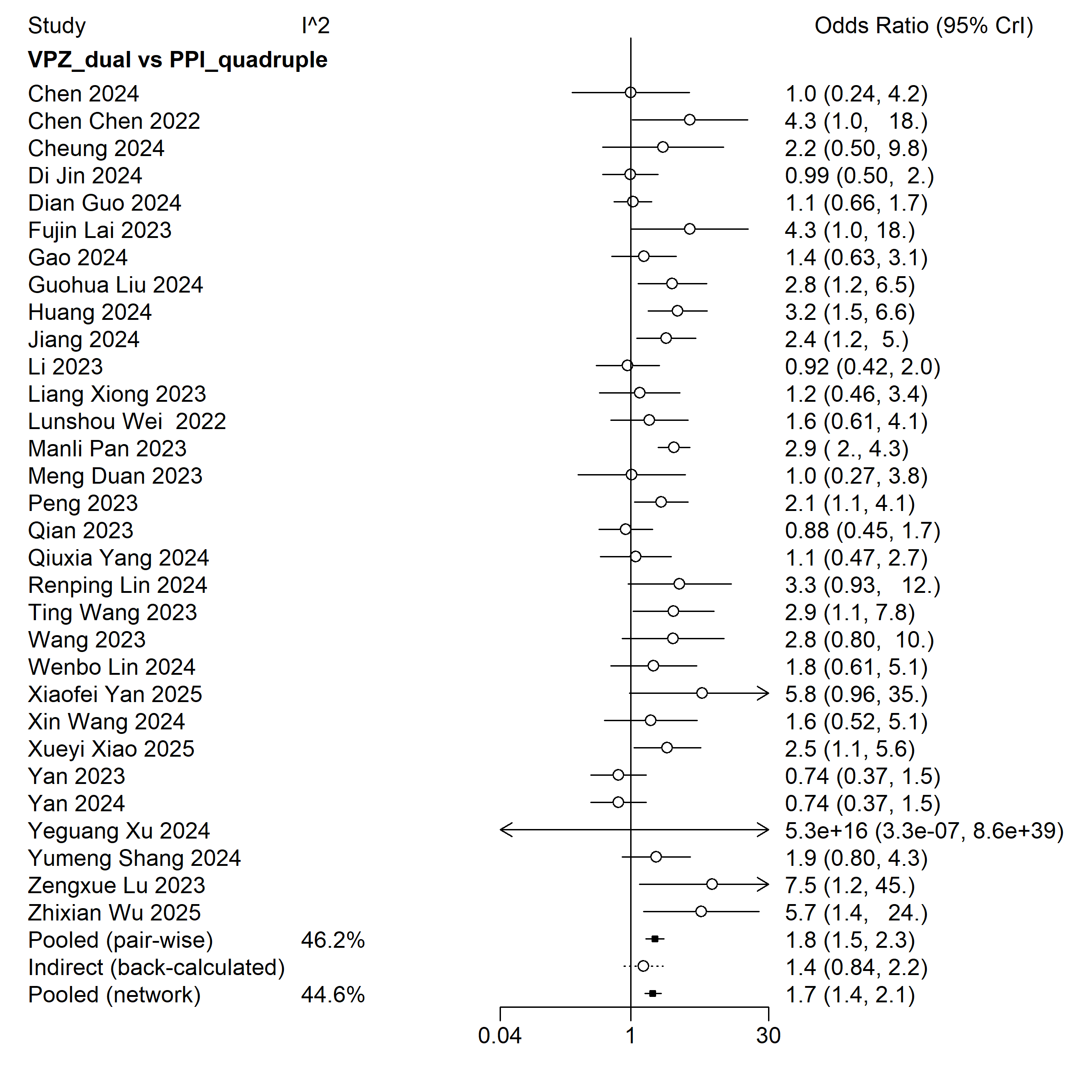

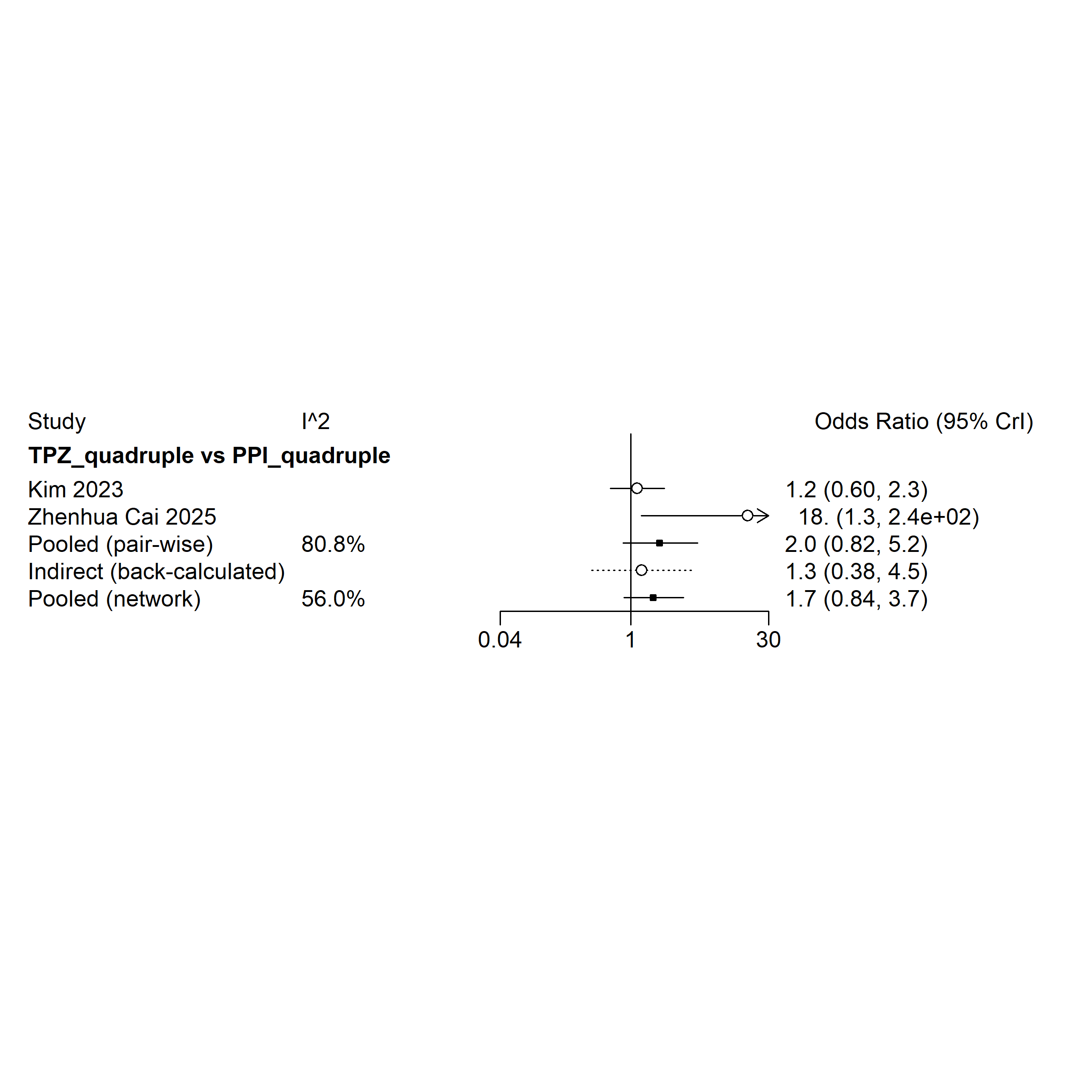

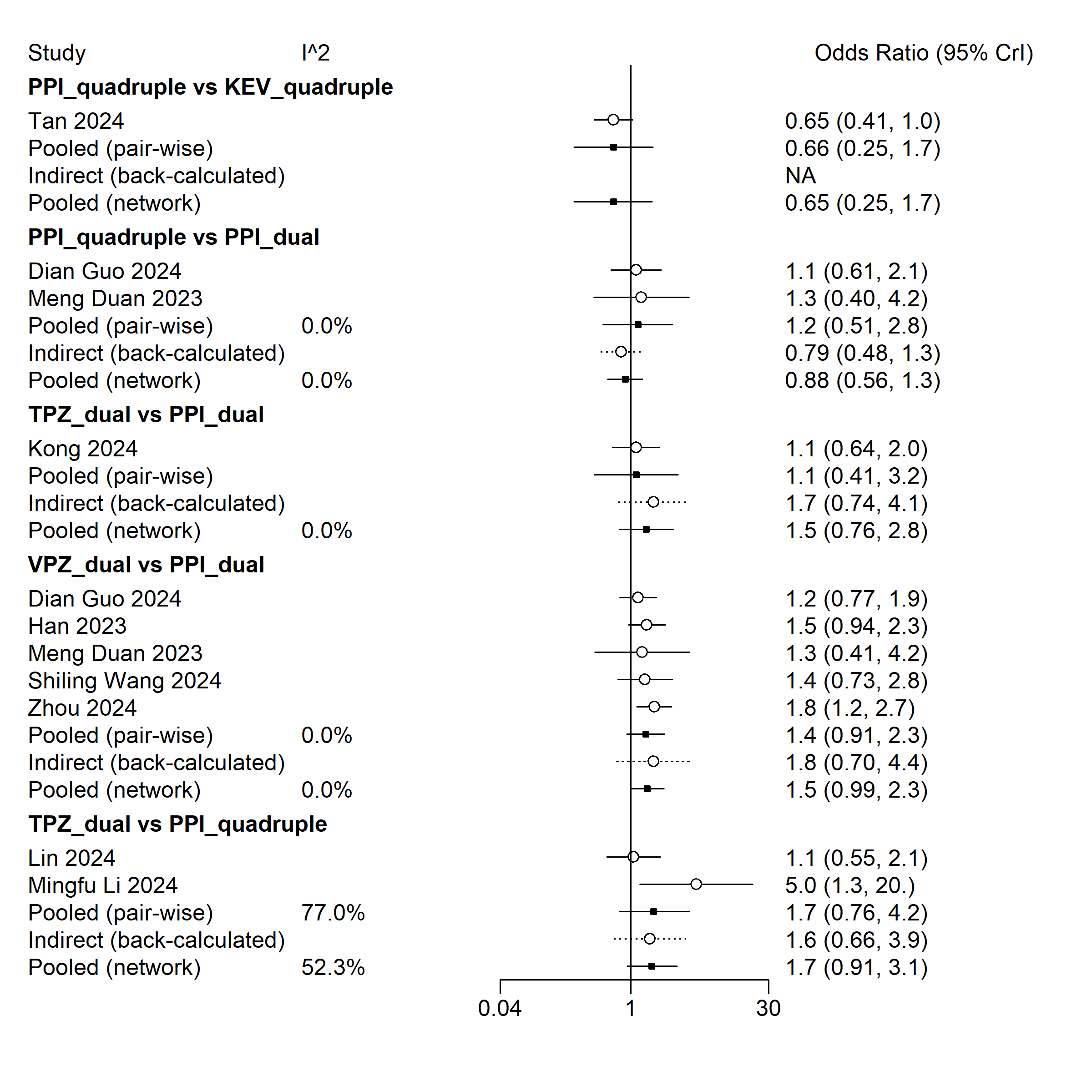


## Figure S 59 Heterogeneity among eradication treatments in overall analysis


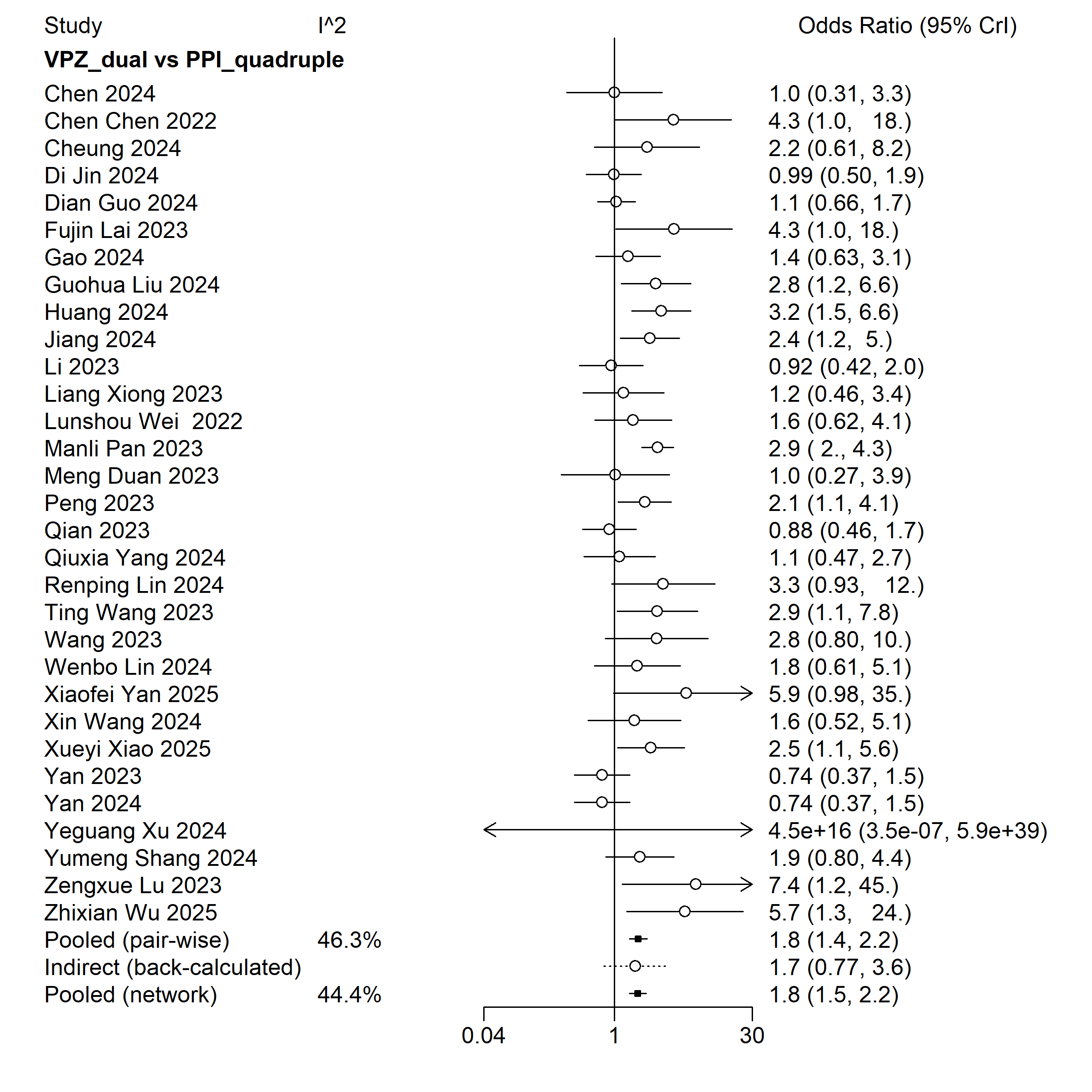

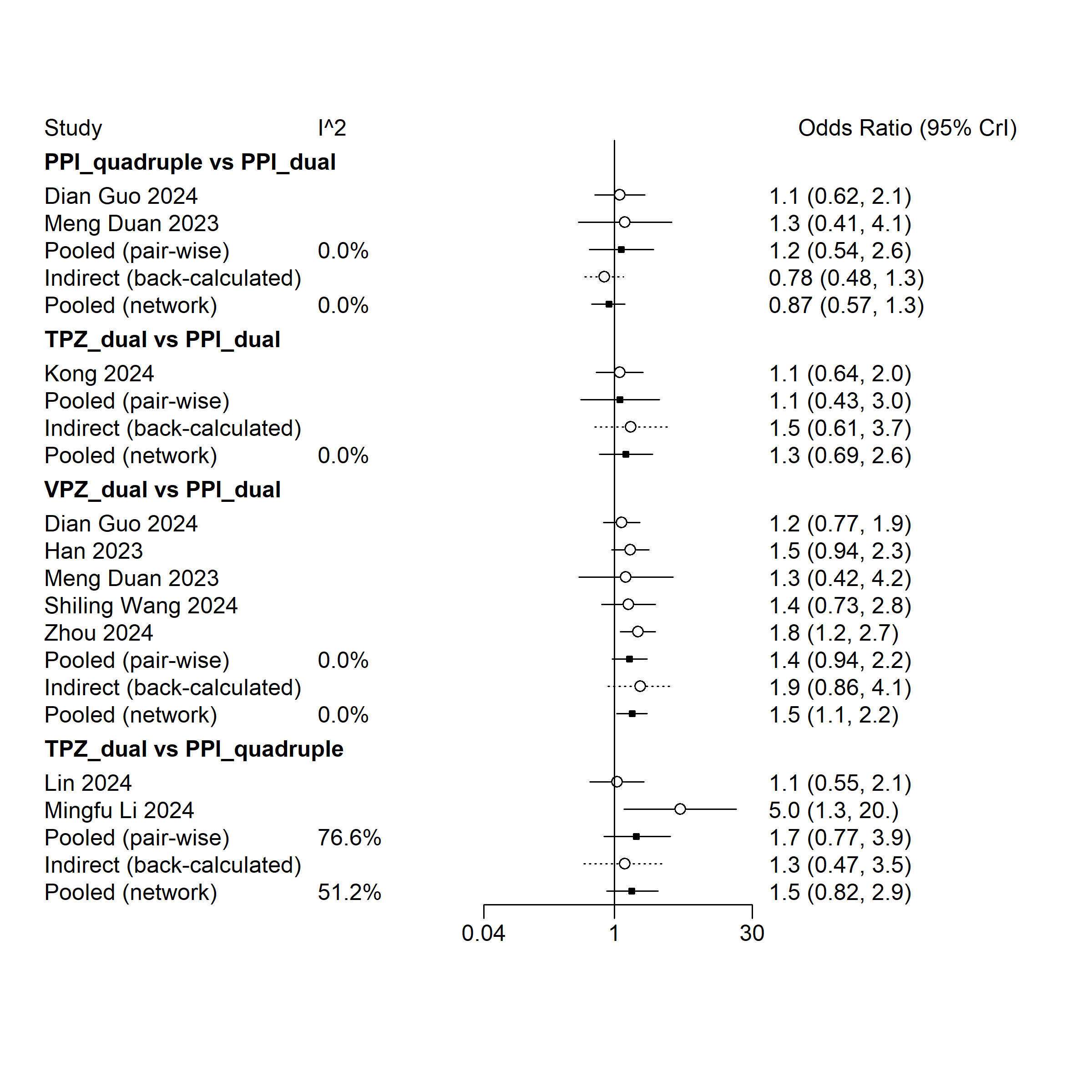

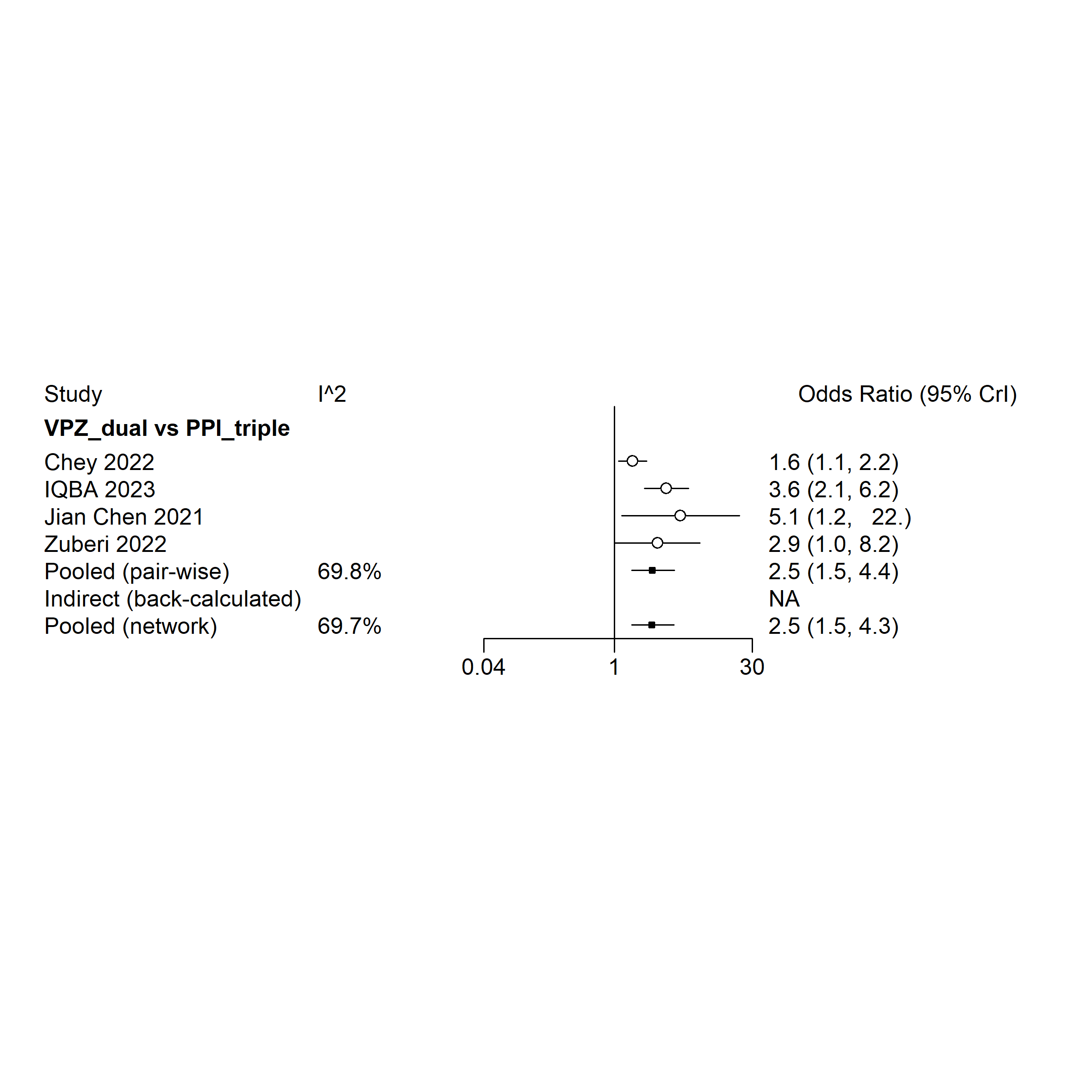


## Figure S 60 Heterogeneity among PCAB-based dual therapy for eradication treatment


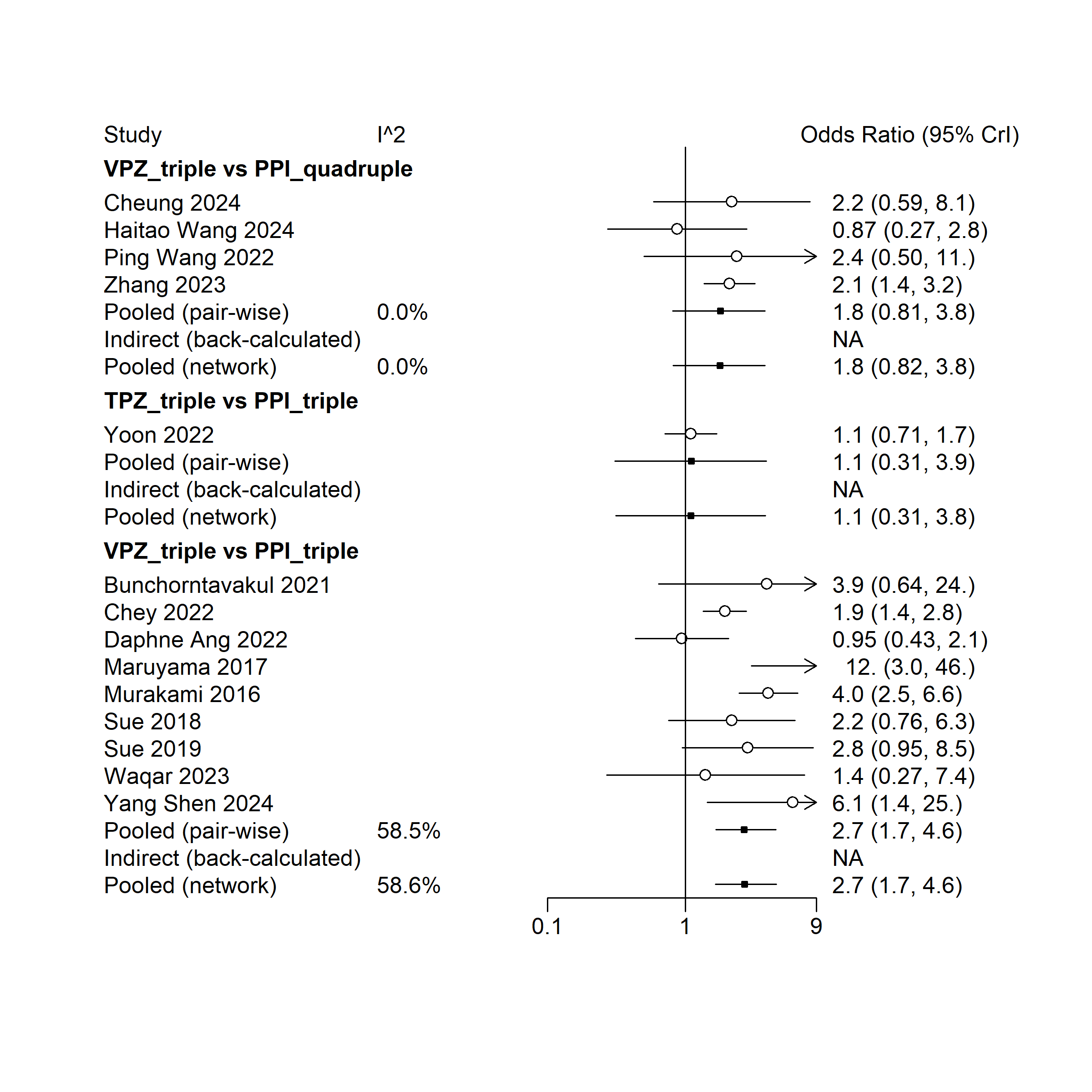


## Figure S 61 Heterogeneity among PCAB-based triple therapy for eradication treatment


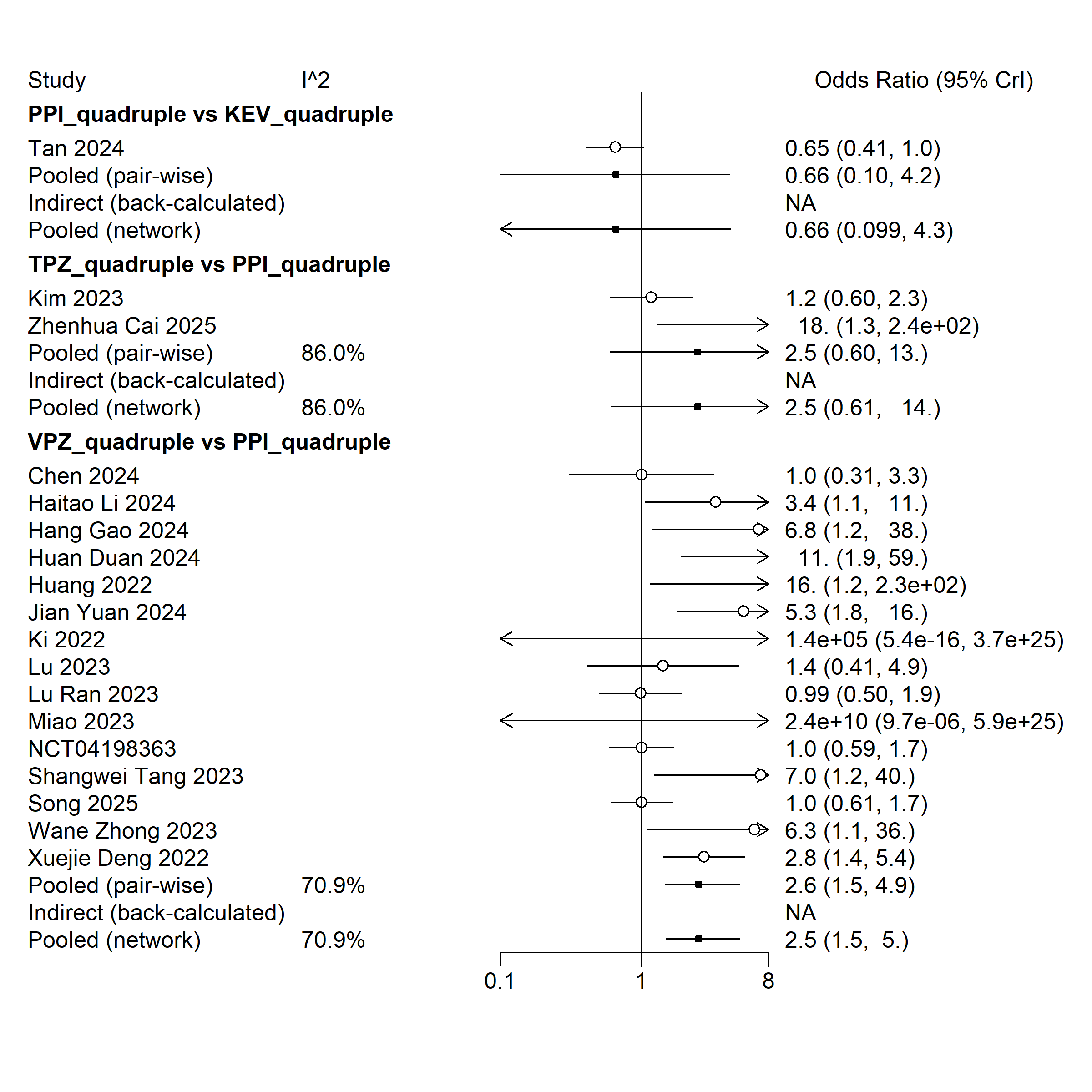


## Figure S 62 Heterogeneity among PCAB-based quadruple therapy for eradication treatment


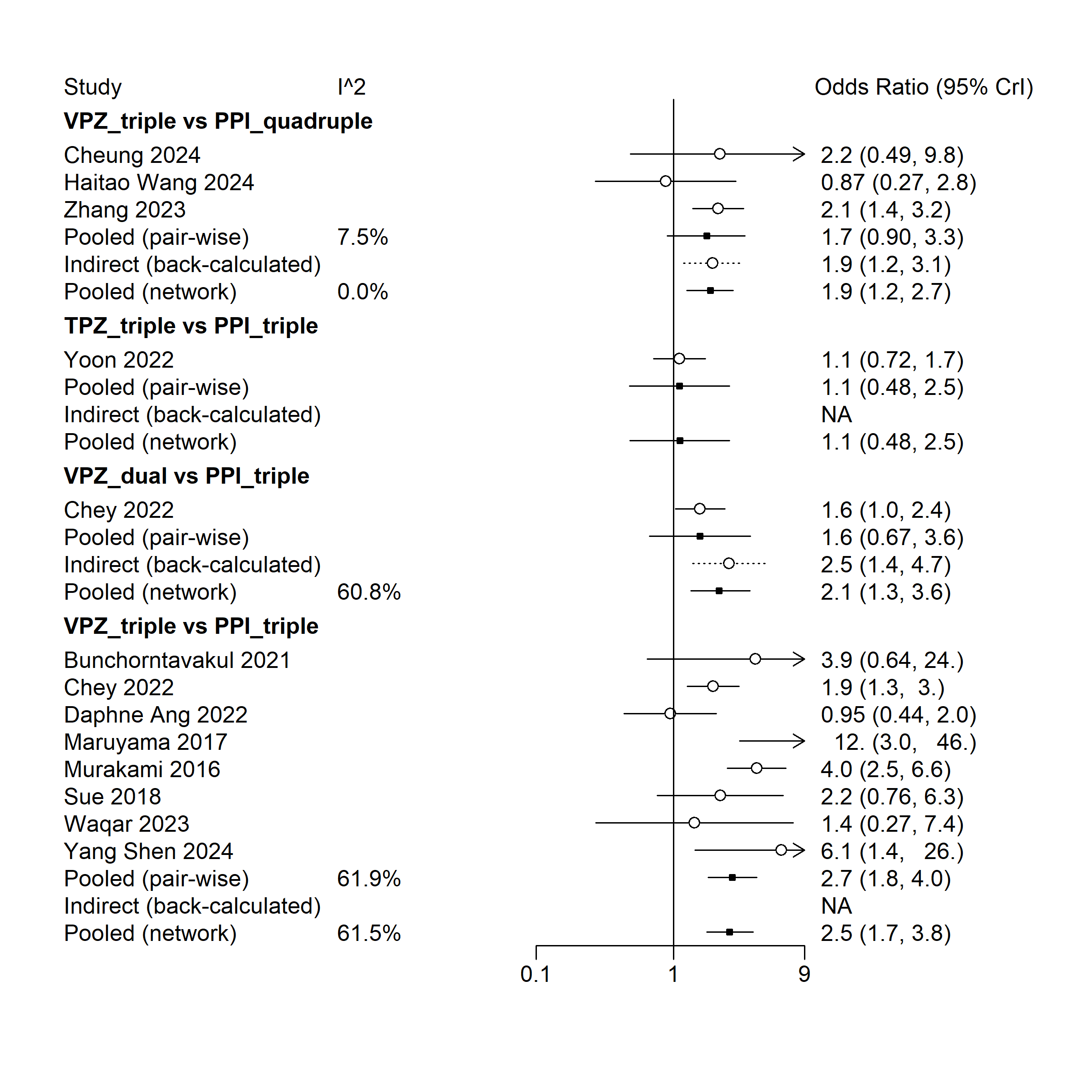

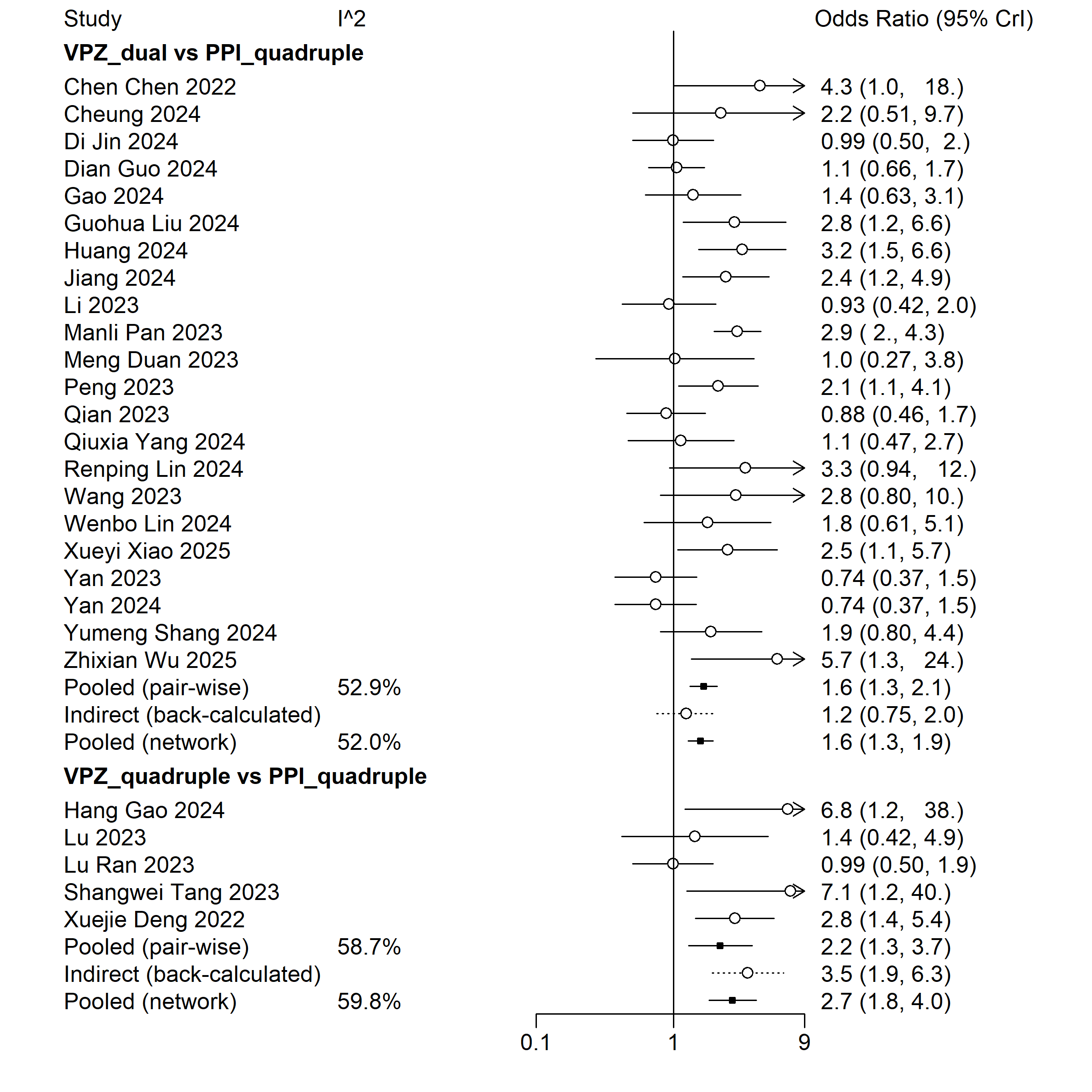

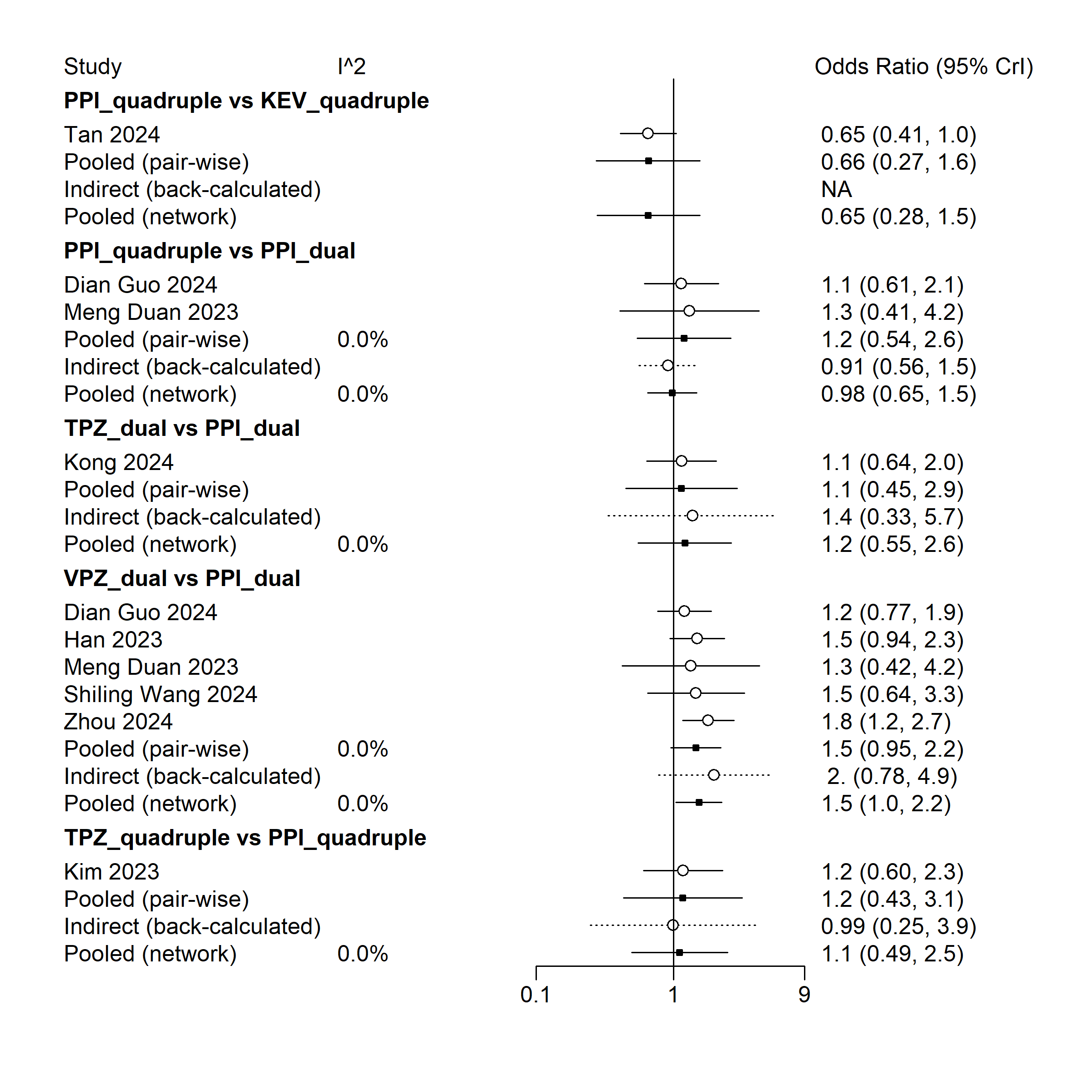

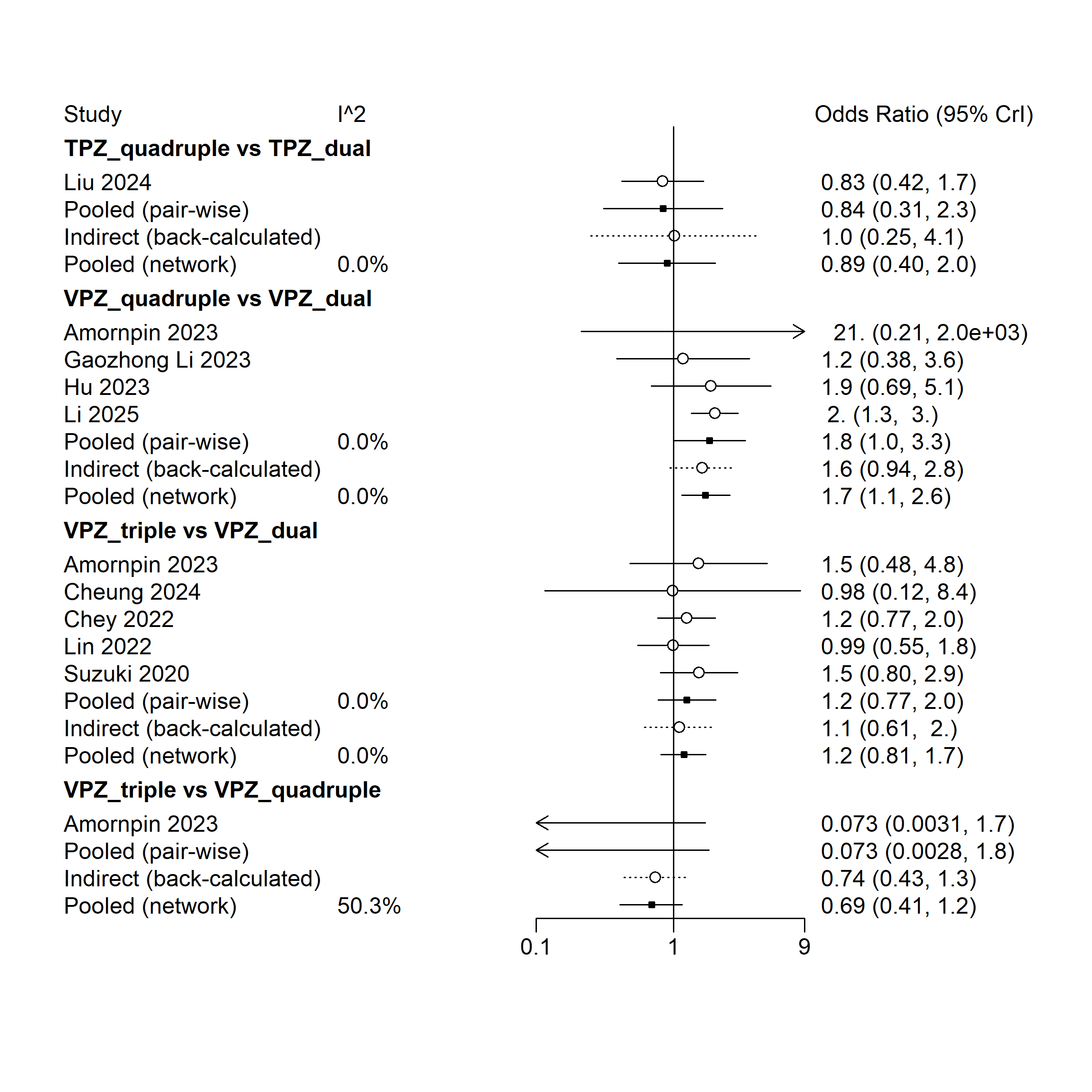


## Figure S 63 Heterogeneity among eradication treatment in treatment-naive patients


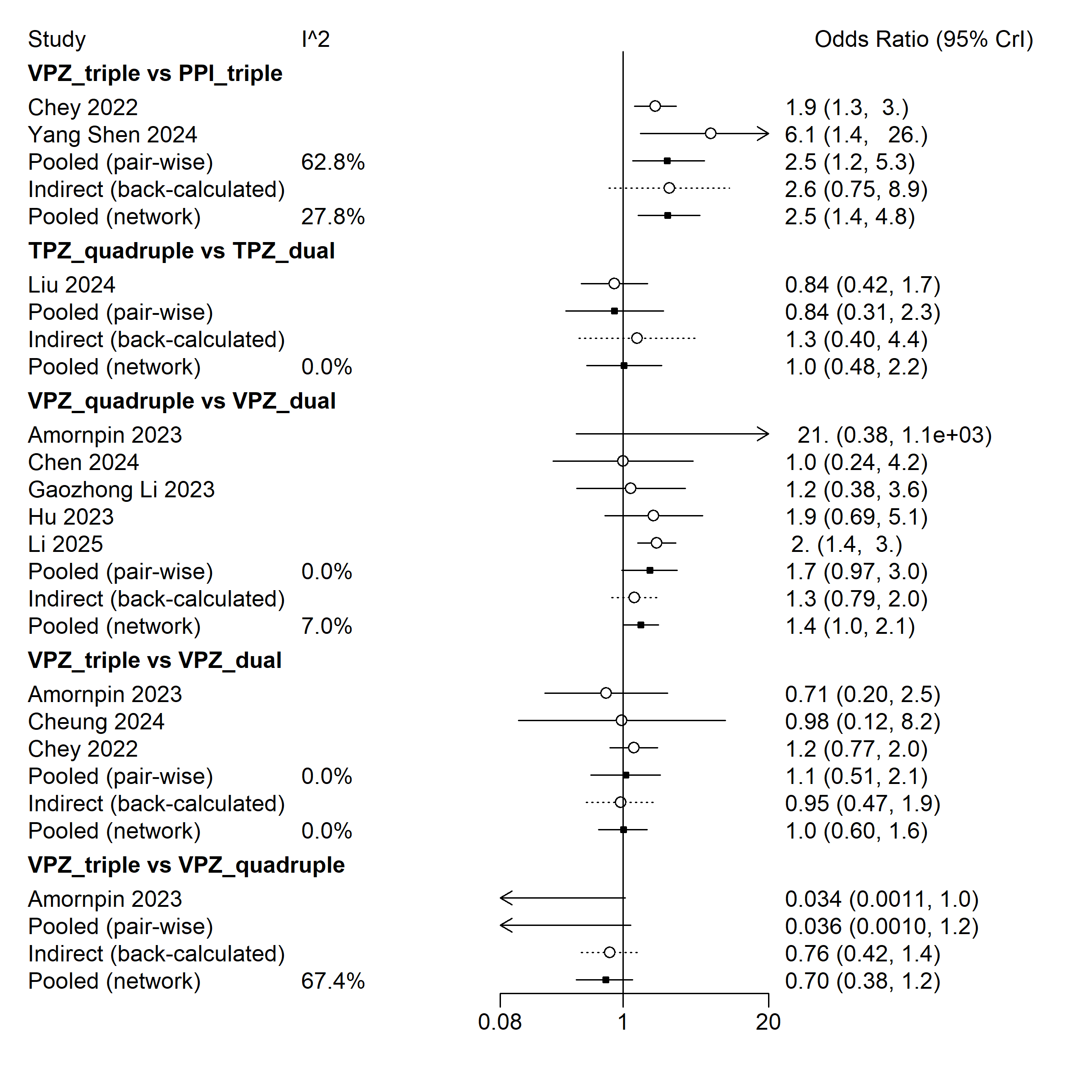

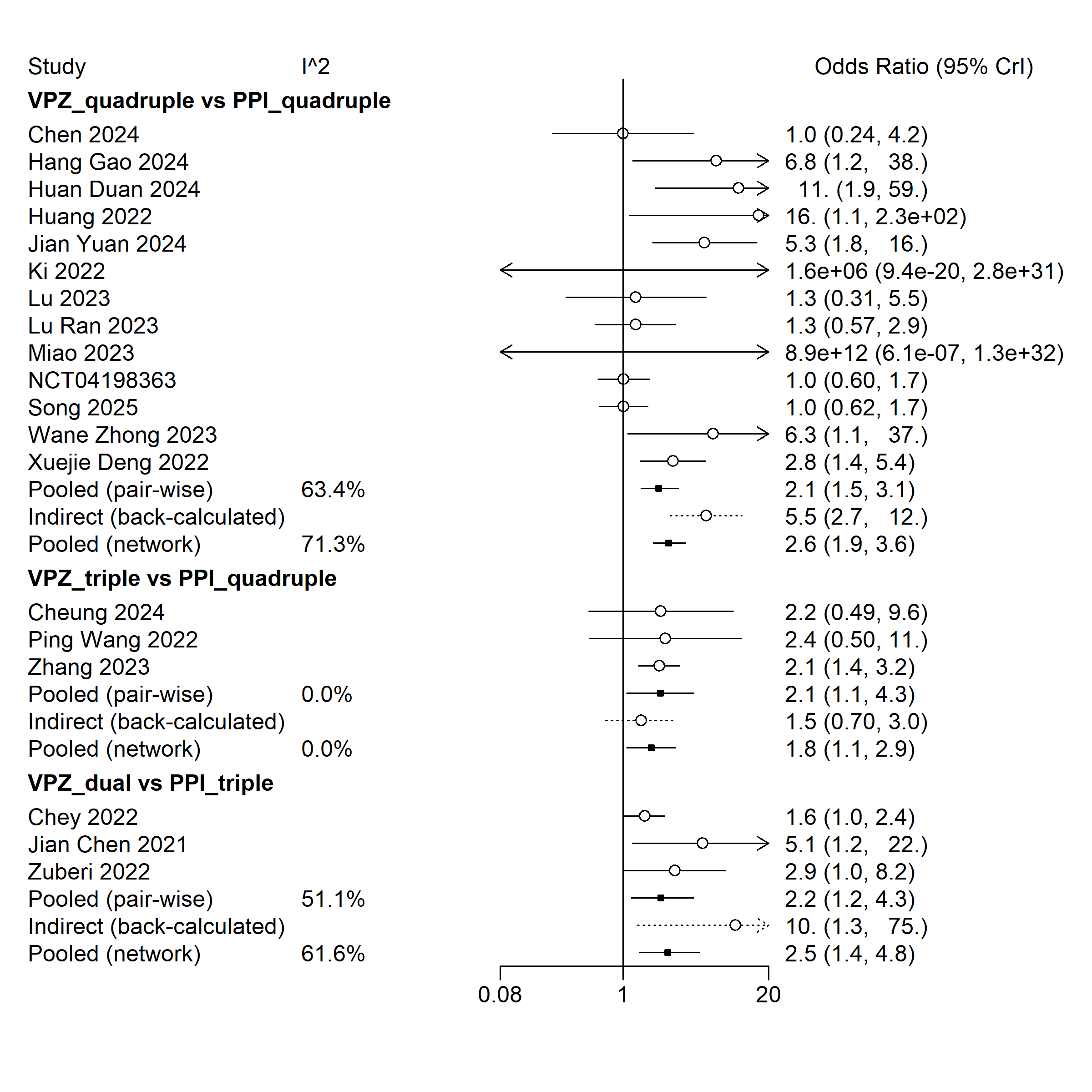

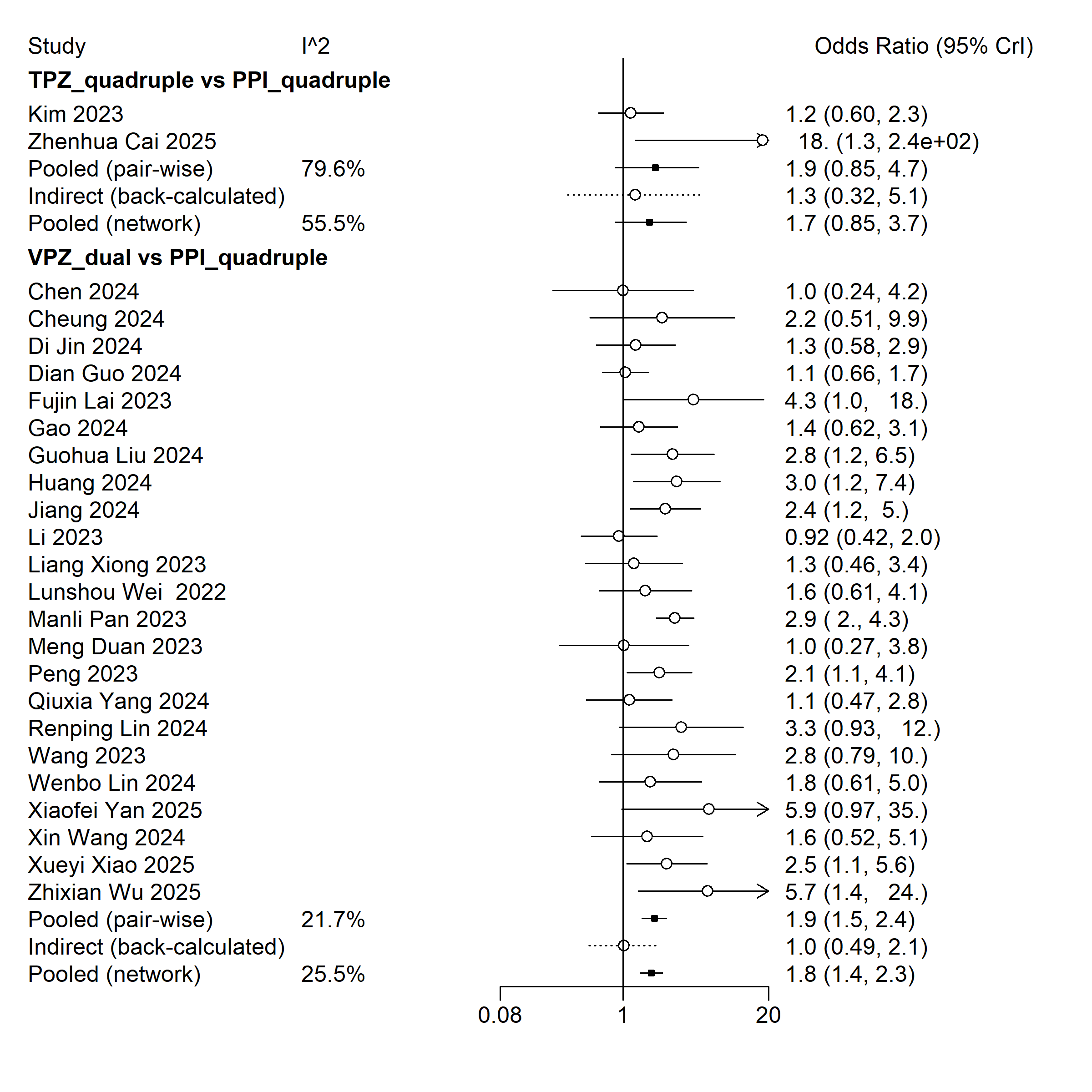


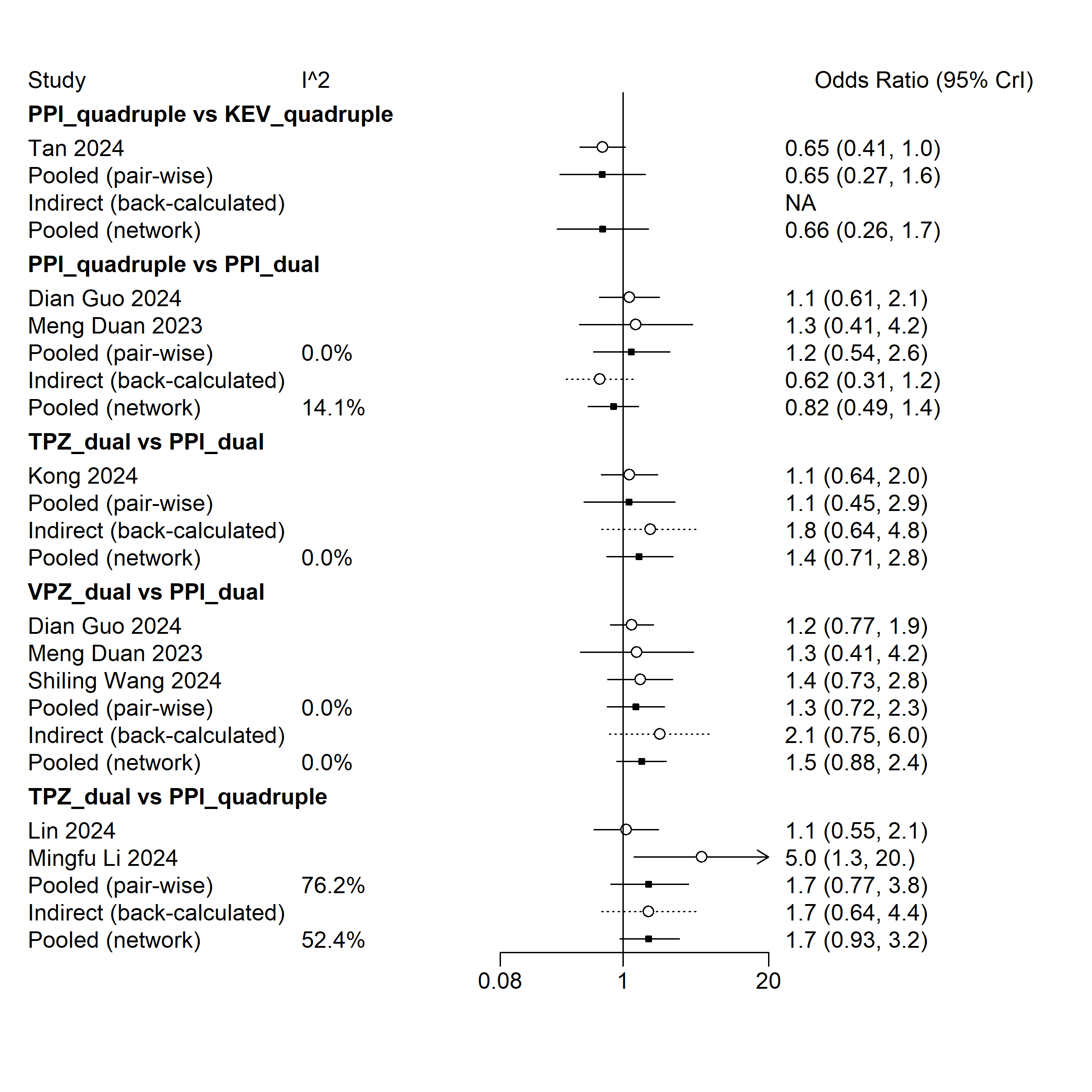


## Figure S 64 Heterogeneity among 14-day eradication treatment


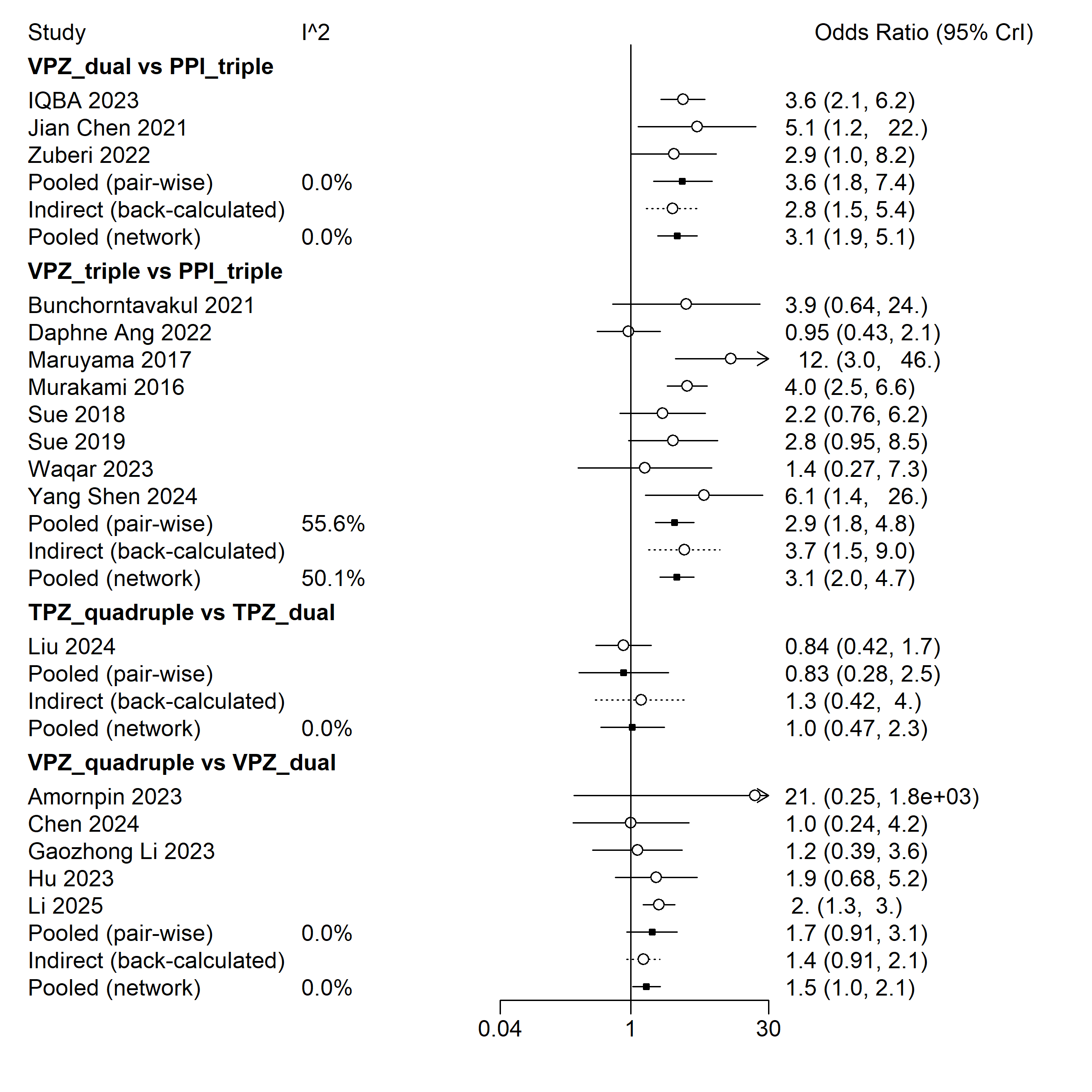

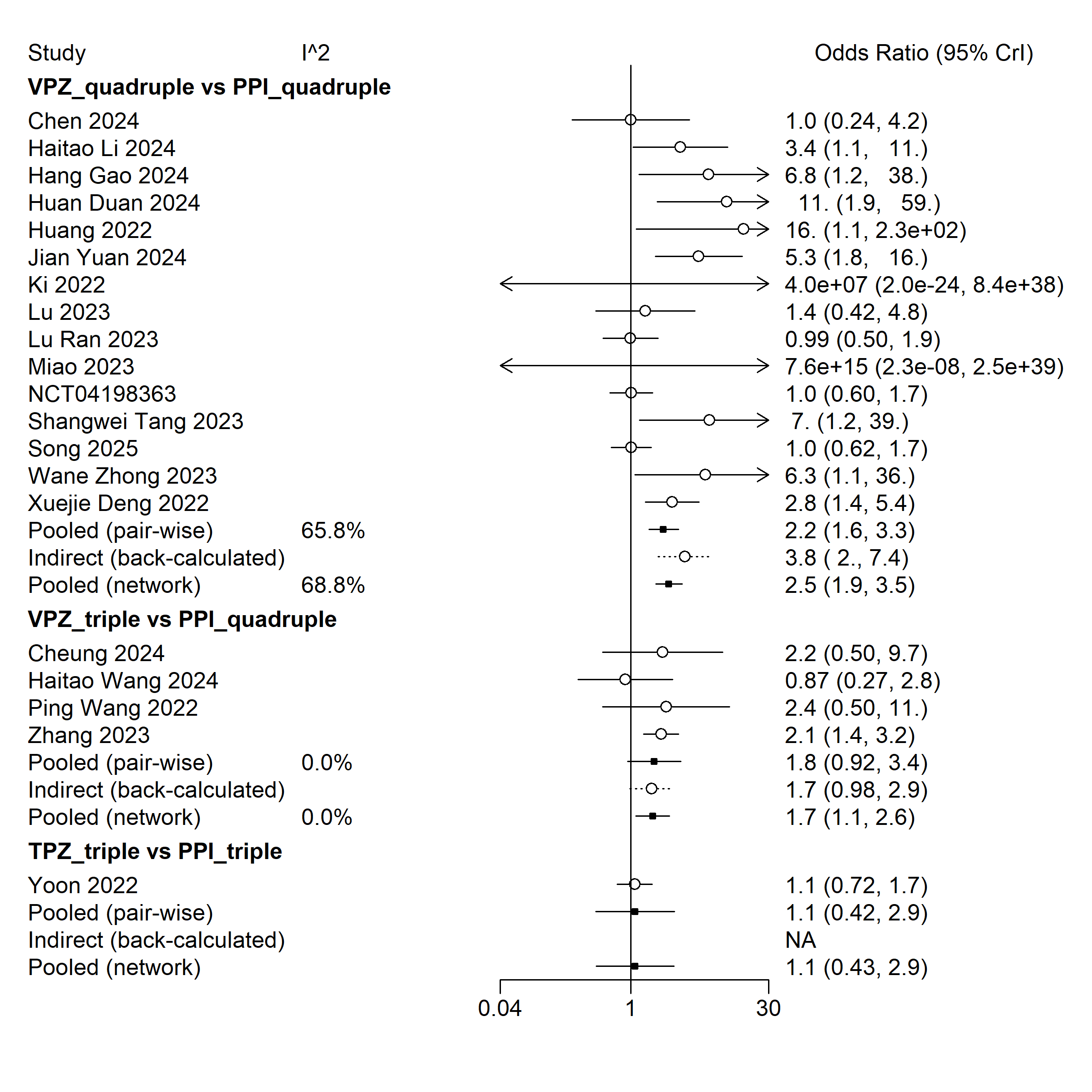

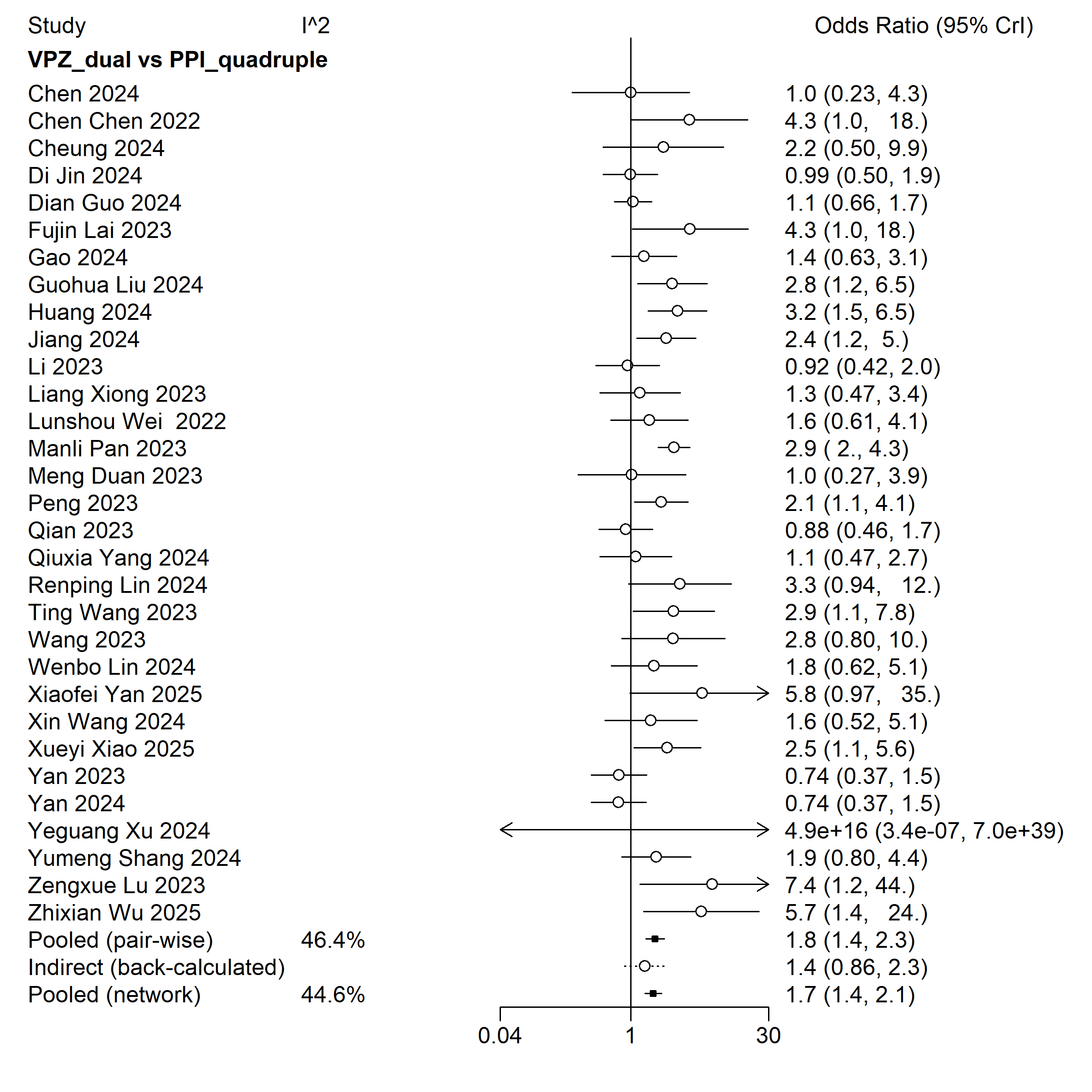


## Figure S 65 Heterogeneity among eradication treatment in Asian patients

## Figure S 66 Heterogeneity among 14-day eradication treatment in treatment-naive patients in China

## Figure S 67 Heterogeneity among eradication treatments based on compliance

## Figure S 68 Heterogeneity among eradication treatments based on adverse events

## Figure S 69 Heterogeneity among eradication treatments based on treatment discontinuation due to adverse events

Appendix 23 Sensitivity analysis in the overall population

## Figure S 70 Ranking with SUCRA for PCAB-based therapies based on eradication rate excluding high-risk studies

| **TPZ_dual** | 0.39 (0.07, 1.81) | 0.25 (0.04, 1.25) | 0.15 (0.02, 0.94) | 0.41 (0.07, 1.99) | 0.13 (0.02, 0.66) | 0.31 (0.04, 1.88) | 0.41 (0.06, 2.46) | 0.49 (0.08, 2.32) | 0.2 (0.04, 0.91) |
| --- | --- | --- | --- | --- | --- | --- | --- | --- | --- |
| 2.56 (0.55, 14.88) | **VPZ_dual** | 0.64 (0.39, 1.05) | 0.38 (0.13, 1.09) | 1.05 (0.64, 1.69) | 0.34 (0.2, 0.55) | 0.8 (0.29, 2.16) | 1.06 (0.42, 2.85) | 1.24 (0.82, 1.94) | 0.52 (0.41, 0.66) |
| 4 (0.8, 24.33) | 1.56 (0.96, 2.56) | **PPI_dual** | 0.59 (0.18, 1.89) | 1.64 (0.82, 3.19) | 0.53 (0.26, 1.05) | 1.25 (0.41, 3.78) | 1.65 (0.6, 4.92) | 1.92 (1.05, 3.73) | 0.81 (0.48, 1.36) |
| **6.83 (1.07, 52.85)** | 2.65 (0.92, 7.93) | 1.7 (0.53, 5.65) | **TPZ_triple** | 2.78 (0.95, 8.23) | 0.9 (0.35, 2.35) | 2.12 (0.49, 9.27) | 2.79 (0.69, 12.35) | 3.27 (1.08, 10.86) | 1.39 (0.47, 4.15) |
| 2.44 (0.5, 14.86) | 0.95 (0.59, 1.56) | 0.61 (0.31, 1.22) | 0.36 (0.12, 1.05) | **VPZ_triple** | 0.33 (0.19, 0.53) | 0.76 (0.25, 2.27) | 1.01 (0.37, 2.98) | 1.18 (0.66, 2.24) | 0.5 (0.31, 0.81) |
| **7.55 (1.52, 46.66)** | **2.93 (1.82, 4.91)** | 1.88 (0.95, 3.84) | 1.11 (0.43, 2.89) | **3.07 (1.88, 5.13)** | **PPI_triple** | 2.35 (0.78, 7.14) | 3.11 (1.11, 9.53) | 3.63 (1.97, 7.22) | 1.53 (0.92, 2.6) |
| 3.23 (0.53, 23.17) | 1.25 (0.46, 3.45) | 0.8 (0.26, 2.46) | 0.47 (0.11, 2.03) | 1.31 (0.44, 3.95) | 0.43 (0.14, 1.28) | **KEV_quadruple** | 1.33 (0.36, 5.21) | 1.54 (0.55, 4.57) | 0.65 (0.25, 1.73) |
| 2.43 (0.41, 17.4) | 0.95 (0.35, 2.36) | 0.61 (0.2, 1.68) | 0.36 (0.08, 1.44) | 0.99 (0.34, 2.73) | **0.32 (0.1, 0.9)** | 0.75 (0.19, 2.81) | **TPZ_quadruple** | 1.17 (0.43, 3.1) | 0.49 (0.19, 1.2) |
| 2.06 (0.43, 12.29) | 0.81 (0.52, 1.22) | **0.52 (0.27, 0.96)** | **0.31 (0.09, 0.93)** | 0.85 (0.45, 1.53) | **0.28 (0.14, 0.51)** | 0.65 (0.22, 1.81) | 0.85 (0.32, 2.33) | **VPZ_quadruple** | 0.42 (0.28, 0.61) |
| **4.88 (1.09, 28.09)** | **1.91 (1.52, 2.44)** | 1.23 (0.74, 2.08) | 0.72 (0.24, 2.14) | **2.01 (1.24, 3.26)** | 0.65 (0.38, 1.09) | 1.53 (0.58, 4.07) | 2.02 (0.83, 5.33) | 2.37 (1.65, 3.57) | **PPI_quadruple** |
| Odds ratio (95% confidence interval) reported. Statistically significant data presented in bold.  *TPZ_dual* Tegoprazan combined with one antibiotic; *VPZ_dual* Vonoprazan combined with one antibiotic; *PPI_dual* Proton pump inhibitor combined with one antibiotic; *TPZ_triple* Tegoprazan combined with two antibiotics; *VPZ_triple* Vonoprazan combined with two antibiotics; *PPI_triple* Proton pump inhibitor combined with two antibiotics; *KEV_quadruple* Keverprazan combined with two antibiotics and bismuth; *TPZ_quadruple* Tegoprazan combined with two antibiotics and bismuth; *VPZ_quadruple* Vonoprazan combined with two antibiotics and bismuth; *PPI_quadruple* Proton pump inhibitor combined with two antibiotics and bismuthver. | | | | | | | | | |

## Table S 30 League matrix of comparative efficacies of PCAB-based eradication therapy excluding high-risk studies
